# A symbiotic supramolecular approach to the design of novel amphiphiles with antibacterial properties against MSRA†
†Electronic supplementary information (ESI) available: Supporting experimental data, methods and computational details. CCDC 1866274 and 1866275. For ESI and crystallographic data in CIF or other electronic format see DOI: 10.1039/c8cc08485h


**DOI:** 10.1039/c8cc08485h

**Published:** 2018-12-04

**Authors:** Stilyana N. Tyuleva, Nyasha Allen, Lisa J. White, Antigoni Pépés, Helena J. Shepherd, Paul J. Saines, Rebecca J. Ellaby, Daniel P. Mulvihill, Jennifer R. Hiscock

**Affiliations:** a School of Physical Sciences , University of Kent , Canterbury , CT2 7NH , UK . Email: J.R.Hiscock@Kent.ac.uk; b School of Biosciences , University of Kent , Canterbury , CT2 7NJ , UK . Email: D.P.Mulvihill@Kent.ac.uk

## Abstract

Herein, we identify supramolecular self-associating amphiphiles (SSAs) as a novel class of antibacterials with activity towards methicillin-resistant *Staphylococcus aureus*. Structure–activity relationships have been identified in the solid, solution and gas phases. Finally, we show that when supplied in combination, SSAs exhibit increased antibacterial efficacy against these clinically relevant microbes.

## Abstract 2

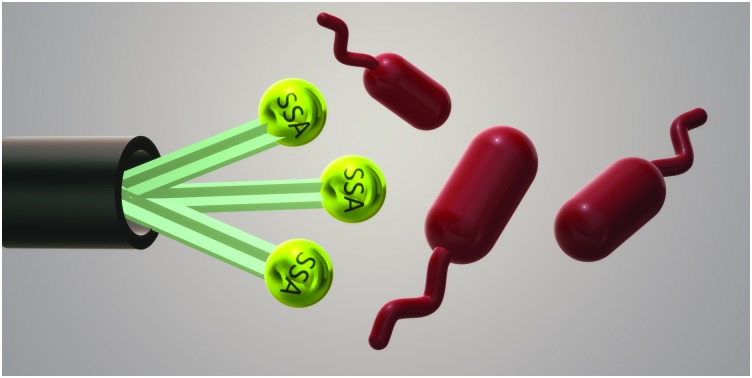
The co-formulation of supramolecular self-associating amphiphiles (SSAs) enhances solution state physicochemical properties and increases efficacy against methicillin-resistant *Staphylococcus aureus*.

## 

The introduction of hydrogen bond donating (HBD) and accepting (HBA) functionalities into the structure of amphiphiles is known to enhance the solution state physical properties observed.^1^ It is generally accepted that this is due to the formation of intramolecular, non-covalent interactions which stabilise molecular self-association processes.^2^ Supramolecular self-associating amphiphilic salts (SSAs) represent a family of such compounds. The self-associative and physicochemical properties for >30 structurally related SSAs have been studied in the solid state, gas phase and solution state, leading towards the prediction of global solution state properties using low level computational modelling.^3^ Here, we discuss the self-associative and physicochemical properties of three such SSAs, both as independent species and when co-formulated in 1 : 1 mixtures. We also show the antibacterial properties of these SSAs against methicillin-resistant *Staphylococcus aureus* (MRSA) and identify symbiotic SSA relationships, which enhance both physicochemical and antibacterial properties.

Since Alexander Fleming discovered penicillin^4^ society has come to rely on an arsenal of compounds to combat (or prevent) bacterial diseases and infections. However, bacterial isolates have now been identified that are resistant to all classes of antimicrobial currently marketed,^5^ including the antiseptic octenidine^6^ and antibiotic of last resort – Colistin.^7^ Left unchecked, it is predicted that by 2050 antimicrobial resistant bacteria will be the cause of more deaths globally per year than cancer.^8^ To focus the worldwide development of novel antimicrobials, the World Health Organization have published a priority list of those bacteria for which new antibiotics are urgently needed, which includes MRSA.^9^ Here, we showcase a supramolecular approach to antibacterial design, with an initial focus on increasing antibacterial activity against clinically relevant MRSA, and highlight the potential of SSAs to contribute to this area of global need.

Compound **1** was synthesized by analogous methods to those of **2**3*c* in a yield of 23%. Compound **3** was obtained through the reaction of 2-aminoanthracene with triphosgene, followed by the addition of *tert*-butyl-aminoacetate and subsequent deprotection before tetrabutylammonium (TBA) hydroxide was added to give **4** in a yield of 34%.
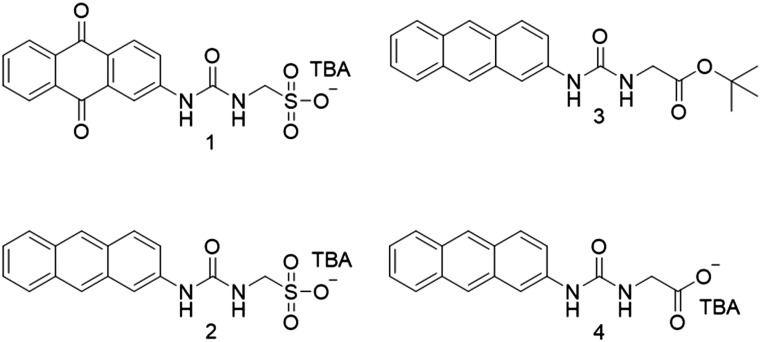


A single crystal X-ray structure obtained of **1** shows the anionic monomeric unit to form a urea-sulfonate dimer, stabilized through the formation of four hydrogen bonds (Fig. 1a). A second single crystal isolated from a mixture of **1** (27.78 mM) and **2** (27.78 mM) in DMSO-*d*_6_, Fig. 1b, was shown to contain both the analogous hydrated homogeneous dimer of **1** (15% occupancy) and the hydrated heterogeneous dimer of **1** and **2** (85% occupancy). However, powder X-ray diffraction analysis of the remaining sample bulk was found to exhibit the greatest similarity to a previously isolated, non-solvated dimer of **1** and **2** (CCDC 1562758†).3*c* It is hypothesized that this preference for heterogeneous urea-sulfonate dimer formation is due to further complex stabilization, obtained from the pairing of the electron rich anthracene with the electron poor anthraquinone ring systems.

**Fig. 1:**
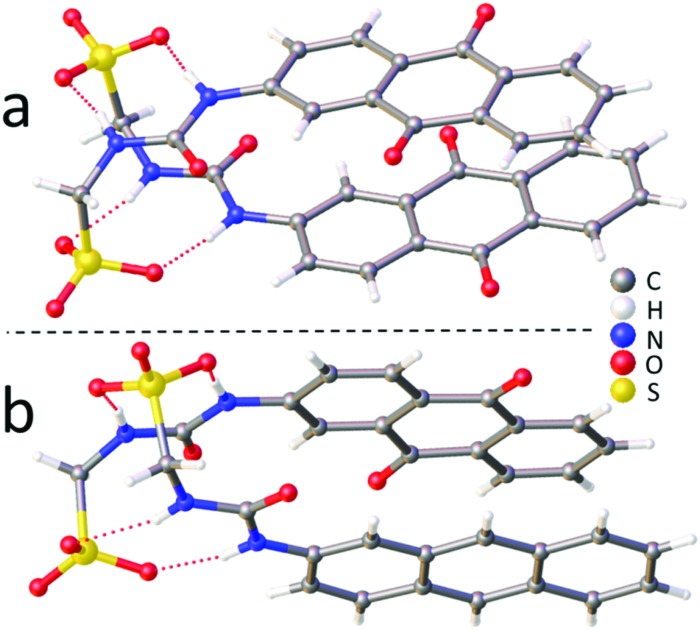
(a) Single crystal X-ray structure of **1**. (b) Single crystal X-ray structure of **1** and **2**. The TBA counter cations, solvent molecules and disorder have been omitted for clarity.

High resolution mass spectrometry analysis of **1**, **2** and **4** also shows evidence of homogenous anionic monomer dimerization, as observed in the solid state (Fig. 1a). However, analysis of those data produced from 1 : 1 mixtures of **1**, **2** and **4** shows evidence not only of homogenous but also heterogeneous dimerization processes (Table 1). Again, this supports those solid-state observations depicted in Fig. 1b.

**Table 1:** An overview of species observed by high resolution ESI –ve mass spectrometry for mixtures containing **1**, **2** and **4** in a 1 : 1 ratio. Ma and Mb represent the anionic component of a SSA

Complex	**1**(Ma) + **2**(Mb) (*m*/*z*)	**1**(Ma) + **4**(Mb) (*m*/*z*)	**2**(Ma) + **4**(Mb) (*m*/*z*)
[Ma + Mb + H]^–^	689.1358	653.1676	623.1981
[Ma + Mb + Na]^–^	711.1001	675.1469	^*a*^
[Ma + Mb + K]^–^	^*a*^	^*a*^	^*a*^
[Ma + Ma + H]^–^	719.1045	719.1113	659.1632
[Ma + Ma + Na]^–^	741.0737	741.0974	681.1400
[Ma + Ma + K]^–^	^*a*^	^*a*^	^*a*^
[Mb + Mb + H]^–^	^*a*^	^*a*^	587.2260
[Mb + Mb + Na]^–^	681.1333	^*a*^	^*a*^
[Mb + Mb + K]^–^	^*a*^	^*a*^	^*a*^

^*a*^SSA solubility prevented experiment.

Both the solid state and gas phase studies allow the observation of those molecular association processes related to **1**, **2**, **4** and the 1 : 1 mixtures thereof in the absence of comparative solvent interactions. Moving into the solution phase, these SSAs were studied in both DMSO and EtOH : H_2_O (1 : 19) environments to enable the observation of simple dimerization processes and complex extended aggregate formation respectively. Quantitative ^1^H NMR studies in both DMSO-*d*_6_ and EtOH : D_2_O (1 : 19) were performed initially to determine the proportion of SSA visible by NMR at a specific concentration. An apparent ‘loss’ of SSA indicates the presence of larger self-associated aggregates in solution that cannot be observed by conventional solution state techniques. In DMSO-*d*_6_ an apparent SSA ‘loss’ was observed for all solutions containing **4** (total concentration = 56 mM) indicating the presence extended aggregated species. Comparative dynamic light scattering (DLS) studies performed with homogenous solutions of **1**, **2**, **4** and 1 : 1 heterogeneous solutions of those same SSAs at 56 mM in DMSO support the results of those quantitative ^1^H NMR studies. Only those solutions containing **4** gave reproducible correlation functions supporting the presence of stable extended aggregates. This is attributed to the difference in preferential self-associative binding modes adopted by the anionic monomeric units of different geometries (Fig. 2), and the strength of those intermolecular hydrogen bonds formed during complexation processes. This hypothesis is further supported by the results of low level computational modelling, Fig. 3.

**Fig. 2:**
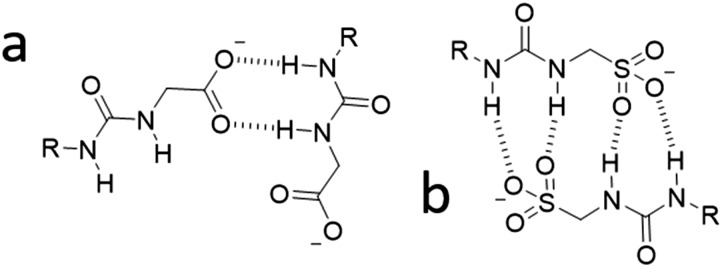
Hypothesised preferential hydrogen bonding modes adopted by (a) the carboxylate-urea tape and (b) the sulfonate-urea dimer motif, influenced by anion geometry.

**Fig. 3:**
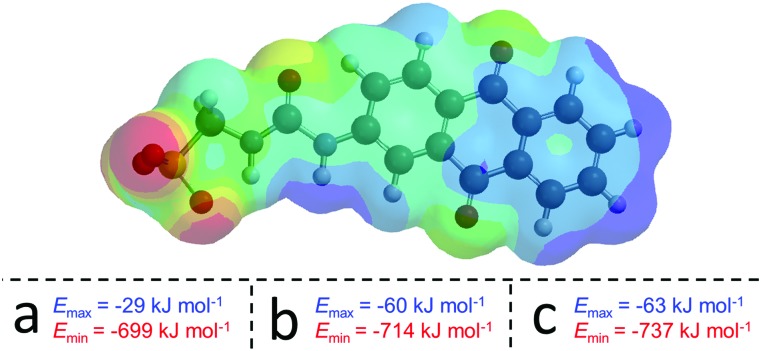
Example electrostatic potential map calculated for the anionic component of **1** and the *E*_max_ and *E*_min_ values calculated for (a) **1**, (b) **2** and (c) **4**, using semi-empirical PM6 modelling methods.

In line with the work of Hunter^10^ and Stewart,^11^ electrostatic potential maps produced by semi-empirical PM6 modelling of energy minimised structures, are used to identify and comparatively quantify those properties associated with the principle HBD site (the most positively charge point on a molecular surface (*E*_max_) in this case the urea NHs) and, the principle HBA site (the most negatively charged point on the molecular surface (*E*_min_) in this case the anionic moiety). A comparative decrease in *E*_max_ and *E*_min_ values indicate the deactivation of the principle HBD moieties and activation of the principle HBA sites respectively.

DLS data show that the solvated diameter of those self-associated structures of **4** that form in DMSO vary from 436 nm, to 496 nm and 404 nm, for solutions containing **4** only, a 1 : 1 mixture of **1** and **4** and a 1 : 1 mixture of **2** and **4** respectively at a total concentration of 56 mM. The sizes of those structures existing in a DMSO solution containing **1** and/or **2** were obtained using ^1^H NMR DOSY. The diameters of these species were shown to be ≤1.41 nm, suggesting the presence monomeric or hydrogen bonded dimeric anionic species only, as observed in the solid state and gas phase. Proton NMR dilution studies in DMSO-*d*_6_ enabled the elucidation of homogeneous, hydrogen bonded dimerization constants (*K*_dim_) for the anionic components of **1** (*K*_dim_ = 17 M^–1^) and **2** (*K*_dim_ = 2 M^–1^).3c,^12^ Although both self-association processes are comparatively weak, the dimer formed by **1** is comparatively stronger than that of **2**. It is hypothesised that this is due to the enhanced electron withdrawing properties of the anthraquinone over the anthracene group. This results in the increased acidity of the NH HBD groups of **1** in contrast to **2** (Fig. 3), which causes an increase in the strength of the hydrogen bonded complex formed. Due to evidence of extended aggregate formation for **4** in DMSO self-association constants were not determined, however an analogous ^1^H NMR dilution study did show evidence of hydrogen bonded self-association. Separate ^1^H NMR titration studies performed with **1** and **2** also showed evidence of heterogeneous anionic hydrogen bonded complex formation, supporting those results obtained for 1 : 1 mixtures of **1** and **2** in the solid state and gas phase.

Moving from a DMSO environment into aqueous solutions, quantitative ^1^H NMR experiments in a 1 : 19 EtOH : D_2_O mixture at 5.56 mM, showed an apparent ‘loss’ of approximately 34%, 77% and 92% for SSAs **1**, **2** and **4** respectively. For those 1 : 1 mixtures of **1**, **2** and **4** an apparent ‘loss’ of approximately 95%, 68% and 94% was observed for **1** and **2**, **1** and **4** and **2** and **4** respectively. This suggests a symbiotic process occurring between these SSAs, resulting in a greater incorporation of amphiphilic species into extended aggregate structures at comparable total molecular concentrations. These symbiotic relationships appear most effective for those 1 : 1 mixtures tested containing **2**. Zeta potential experiments preformed at total concentrations of 5.56 mM and 0.56 mM for 1 : 19 EtOH : H_2_O solutions of **1**, **2**, **4** and 1 : 1 mixtures of those three SSAs, show stable self-associated aggregate formation in all solutions tested, Table 2. With the exception of those solutions containing **1** only, a decrease in zeta potential was observed with decreasing SSA concentration, which indicates the stability of those extended aggregates formed is concentration dependent. This is particularly noticeable for those structures containing a carboxylate moiety (**4**). It is again hypothesised that this is due to the anionic geometry influenced hydrogen bonding mode adopted, strength of hydrogen bonded complexation and/or hydrophilicity of the anionic units of **1**, **2** and **4**.

**Table 2:** Summary of zeta potential, CMC, surface tension at CMC and average intensity particle size distribution DLS data obtained for SSAs **1**, **2**, **4** and mixtures of SSA **1** and **2**, **1** and **4**, **2** and **4** in an EtOH : H_2_O (1 : 19) solution

SSA	Zeta potential (mV)		CMC (mM)	Surface tension at CMC (mN m^–1^)	Peak maxima (nm)	
	5.56 mM	0.56 mM			5.56 mM	0.56 mM
**1**	–64	–78	8.17	52.75	142	124
**2**	–88	–78	2.523*c*	43.153*c*	209	198
**4**	–57	–38	2.75	52.65	217	324
**1** and **2**	^*a*^	–83	1.09	52.49	^*a*^	224
**1** and **4**	–65	–48	5.12	51.60	288	275
**2** and **4**	–60	–41	2.48	45.74	155	193

^*a*^SSA solubility prevented experiment.

The critical micelle concentrations (CMC), Table 2, were calculated for **1**, **2**, **4** and 1 : 1 mixtures of these salts in a 1 : 19 (EtOH : H_2_O) solution, *via* the pendant drop method. Here CMC is defined as the concentration at which surface tension was no longer found to decrease with increasing SSA concentration. Stable extended aggregate formation was observed by DLS, zeta potential and microscopy at concentrations below the experimentally derived CMC value. However, it is possible for stable aggregates to exist in solution at concentrations below the CMC.^13^ Those values obtained for both anthracene containing SSAs were found to be similar, 2.52 mM and 2.75 mM for solutions of **2** and **4** respectively. However, the substitution of the anthracene group (**2**) for the anthraquinone unit (**1**) resulted in an increased CMC of 8.17 mM. When combining **1**, **2** and **4** in 1 : 1 mixtures the presence of **2** was found to lower the CMC value when compared to that calculated for homogenous solutions of **1**, **2** and **4**. This again indicates a symbiotic relationship between these SSAs.

The size of those self-associated, extended aggregate structures produced by **1**, **2**, **4** and the 1 : 1 mixtures thereof, in a 1 : 19 EtOH : H_2_O solution were established indirectly by DLS studies (Table 2) and directly by solution state microscopy. The sizes of structures observed by these two techniques were found to be in agreement (see ESI†). The size of those structures observed by DLS at both 5.56 mM and 0.56 mM were found to remain stable with **1**, **2** and **1** and **4** however, those solutions containing **4** only or mixtures of **2** and **4** were less so. Again, this alteration of self-associated aggregate size is more prevalent in those systems containing a carboxylate ion, supporting the findings of CMC and quantitative ^1^H NMR studies. The intrinsic fluorescence properties of the anionic components contained in **1**, **2** and **4** means that we can directly visualise those extended aggregates formed through combination of both transmission and fluorescence microscopy. To reduce the movement of those extended aggregate structures in solution, the samples were stabilised using agar pads in line with methods reported by Levin.^14^ These microscopy studies showed the formation of mainly spherical structures, as illustrated in Fig. 4, for the 1 : 1 mixture of **1** and **2** in a 1 : 19 EtOH : H_2_O solution.

**Fig. 4:**
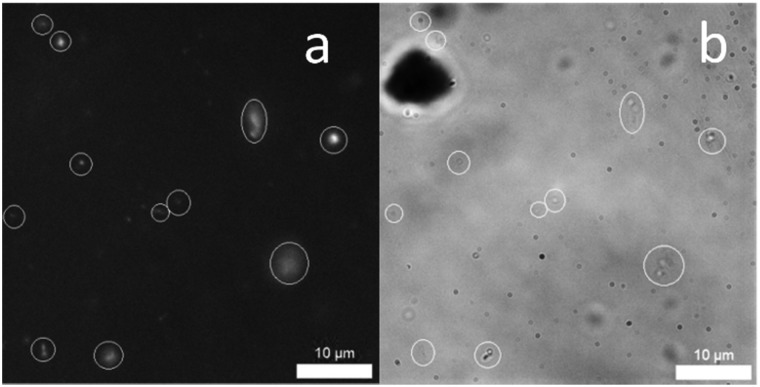
(a) A DAPI filtered fluorescence microscopy image of **1** and **2** (1 : 1 mix) (total concentration = 1 mM) in an EtOH : H_2_O (1 : 19) solution. (b) An analogous transmitted light microscopy image. Evidence of aggregated spherical structures are circled for clarity. Photo bleaching during the imaging process resulted in loss of fluorescence emission intensity.

SSAs **1**, **2** and **4** all show antibacterial activity against clinical isolates of MRSA USA300. The minimum inhibitory concentration (MIC_50_) of compound(s) needed to prevent 50% of cell growth was determined using growth curve analysis against cells in log phase, Fig. 5. Considering those MIC_50_ values obtained for homogenous solutions of **1**, **2** and **4**, SSA **2** was shown to be the most effective antibacterial agent (MIC_50_ = 0.46 mM). Exchanging the anthracene for the anthraquinone group (**1**) was found to decrease antibacterial efficacy as was the substitution of the sulfonate anion for the carboxylate ion (**4**). However, it was also observed that the presence of **2** in 1 : 1 SSA mixtures dramatically increased the antibacterial efficacy for these examples. This provides evidence not only of a novel class of antibacterial (SSAs) but also a synergistic relationship between multicomponent SSA systems. Therefore, it is evidenced here that the SSA mode of antibacterial action associated with **1**, **2** and **4** is aided by the presence of **2** in 1 : 1 mixtures containing **1** or **4**.

**Fig. 5:**
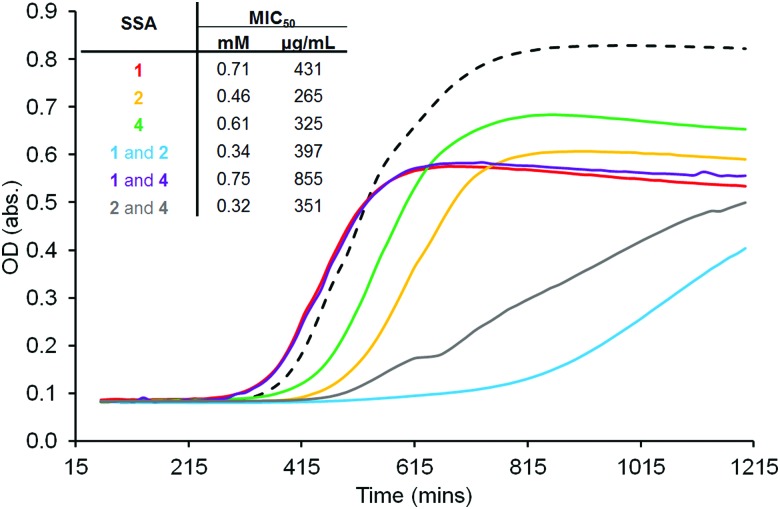
Growth curves obtained at a total concentration of 0.375 mM and, MIC_50_ values calculated for **1**, **2**, **4** and 1 : 1 mixtures of those three SSAs against MRSA USA300. Dashed line = solvent addition only.

In conclusion we have explored the self-associative properties of **1**, **2**, **4** and the 1 : 1 mixtures thereof in the solid, solution and gas phase. In a 1 : 19 EtOH : H_2_O solution the size and stability of those aggregates formed were determined indirectly by DLS and zeta potential measurements however, self-associated spherical structures were directly visualized for **1**, **2**, **4** and a 1 : 1 mixture of **1** and **2** using a combination of fluorescence and transmission microscopy. All three SSAs were shown to illicit an antibacterial response against MRSA USA300 with the trend in CMC echoing that observed for MIC_50_. In both cases the presence of **2**, when supplied as a 1 : 1 mixture with **1** and **4**, was found to lower CMC and increase antibacterial efficacy. This indicates that the formation of those self-associated structures is imperative for effective SSA delivery to the bacterial cell. Expanding on these initial observations, our current work continues to establish the full potential of SSAs as antibacterial agents against a broader spectrum of clinically relevant microbes, developing ever more effective next generation systems through the identification of molecular structure – physicochemical property – antibacterial efficacy relationships.

We acknowledge the University of Kent and the BBSRC (BB/P011934/1) for funding. We also thank T. A. Eastwood (microscopy) and K. Howland (mass spectrometry).

## Conflicts of interest

There are no conflicts to declare.

## Supplementary Material

Supplementary information

Crystal structure data
