# Supplementary material for: A symbiotic supramolecular approach to the design of novel amphiphiles with antibacterial properties against MSRA
Source: Chem Commun (Camb). 2018 Dec 4;55(1):95–8. doi: 10.1039/c8cc08485h (PMC6336146; doi:10.1039/c8cc08485h)
Supplement: Supplementary file 1 [file CC-055-C8CC08485H-s001.pdf]

Electronic Supplementary Material (ESI) for Chemical Communications.
This journal is © The Royal Society of Chemistry 2018

A Symbiotic Supramolecular Approach to the Design of Novel Amphiphiles with Antibacterial Properties Against MSRA

Stilyana N. Tyuleva,[a] Nyasha Allen,[b] Lisa J. White,[a] Antigoni Pépés,[a] Helena J. Shepherd,[a] Paul Saines,[a] Rebecca J. Ellaby,[a] and Daniel P. Mulvihill*[b] and Jennifer R. Hiscock*[a]

[a] School of Physical Sciences, University of Kent, Canterbury, UK, CT2 7NH, E-mail: J.R.Hiscock@Kent.ac.uk.
[b] School of Biosciences, University of Kent, Canterbury, UK, CT2 7NJ, E-mail: D.P.Mulvihill@Kent.ac.uk.

Electronic Supplementary Information

Abstract: Herein, we identify Supramolecular Self-associating Amphiphiles (SSAs) as a novel class of antimicrobials, with activity towards Methicillin-resistant *Staphylococcus aureus*. Structure-activity relationships are identified in the solid, solution and gas phases. Finally, we show that when supplied in combination, SSAs exhibit increased antimicrobial efficacy against these clinically relevant microbes.

DOI: 10.1002/anie.2016XXXXX

Table of Contents

Table of Contents 2
Experimental Procedures 3
Chemical Structures 4
Chemical Synthesis 5
NMR Characterization 6
Mass Spectrometry Results 16
^{1}H NMR DOSY Data 27
^{1}H NMR Titration Study Data 30
^{1}H NMR Dilution Study Data 31
Quantitative ^{1}H NMR Data 33
Fluorescence Data 39
Surface Tension Measurements and Critical Micelle Concentration (CMC) Determination 42
Dynamic Light Scattering (H_2O:EtOH 19:1) 45
    Particle Size Distribution 45
    Correlation Function 50
Dynamic Light Scattering (DMSO) 56
    Particle Size Distribution 56
    Correlation Function 59
Dynamic Light Scattering Overview 62
Zeta Potential Data 63
Single Crystal X-Ray Diffraction Structures 69
Powder X-Ray Diffraction Study Data 711
Microscopy 72
MIC_{50} Determination 95
References 107

Experimental Procedures

General Experimental: A positive pressure of nitrogen and oven dried glassware were used for all reactions. All solvents and starting materials were purchased from known chemical suppliers or available stores and used without any further purification unless specifically stipulated. The NMR spectra were obtained using a Burker AV2 400 MHz or AVNEO 400 MHz spectrometer. The data was processed using ACD Labs or Topspin software. NMR Chemical shift values are reported in parts per million (ppm) and calibrated to the centre of the residual solvent peak set (s = singlet, br = broad, d = doublet, t = triplet, q = quartet, m = multiplet). Tensiometry measurements were undertaken using the Biolin Scientific Theta Attension optical tensiometer. The data was processed using Biolin OneAttension software. A Hamilton (309) syringe was used for the measurements. The melting point for each compound was measured using Stuart SMP10 melting point apparatus. High resolution mass spectrometry was performed using a Bruker microTOF-Q mass spectrometer and spectra recorded and processed using Bruker's Compass Data Analysis software. Infrared spectra were obtained using a Shimadzu IR-Affinity 1 model Infrared spectrometer. The data are analysed in wavenumbers (cm^{-1}) using IRsolution software. Fluorescence emission and excitation spectra were obtained using Agilent Technology Cary Eclipse Fluorescence Spectrophotometer and processed using Eclipse ADL (Advanced Reads) software, the results were reported in nm. DLS and Zeta Potential studies were carried out using Anton Paar Litesizer™ 500 and processed using Kalliope™ Professional. Cellular growth curve measurements obtained using Thermo Scientific Multiscan Go 1510-0318C plate reader and recorded using the SkanIt Software 4.0.

Mass Spectrometry: Approximately 1 mg of each compound or mixture of compounds was dissolved in 1 mL of methanol. This solution was further diluted 100-fold before undergoing analysis. 10 µL of each sample was then injected directly into a flow of 10mM ammonium acetate in 95% water (flow rate = 0.02 mL/min).

Self-association constant calculation: Self-association constants were determined using Bindfit v0.5 (http://app.supramolecular.org/bindfit/). All the data can be accessed online using the hyperlinks provided.

Fluorometry studies: All samples were prepared by serial dilution from an initial stock solution.

Tensiometry Studies: All the samples were prepared in a EtOH:H_2O (1:19) solution. All samples underwent an annealing process in which the various solutions were heated to approximately 40 °C before being allowed to cool to room temperature, allowing each sample to reach a thermodynamic minimum. All samples were prepared through serial dilution of the most concentrated sample. Three surface tension measurements were obtained for each sample at a given concentration, using the pendant drop method. The average values were then used to calculate the critical micelle concentration (CMC).

DLS Studies: All vials used for preparing the samples were clean dry. All solvents used were filtered to remove any particulates that may interfere with the results obtained. Samples of differing concentrations were obtained through serial dilution of a concentrated solution. All samples underwent an annealing process, in which they were heated to 40 ^{0}C before being allowed to cool to 25 ^{0}C. A series of 5 runs were recorded at 40 ^{0}C to check for sample stability before a series of 10 runs were recorded at 25 ^{0}C.

Zeta Potential Studies: All vials used for preparing the samples were clean dry. All solvents used were filtered to remove any particulates that may interfere with the results obtained. All samples underwent an annealing process in which the various solutions were heated to approximately 40 °C before cooling to room temperature for 30 mins, allowing each sample to reach a thermodynamic minimum. The final zeta potential value given is an average of the number of experiments conducted at 25 °C. Samples of differing concentrations were obtained through serial dilution of an initial stock solution.

Single Crystal X-ray Studies: A suitable crystal of each amphiphile was selected and mounted on a Rigaku Oxford Diffraction Supernova diffractometer. Data were collected using Cu Kα radiation at 100 K or 293 K as necessary due to crystal instability at lower temperatures. Structures were solved with the ShelXT[1] or ShelXS structure solution programs via Direct Methods and refined with ShelXL[2] by Least Squares minimisation. Olex2[3] was used as an interface to all ShelX programs (CCDC 1866274-1866275).

X-ray Powder Diffraction Studies: Bulk solid-state phase purity was examined using powder X-ray diffraction carried out on a PANalytical Empyrean diffractometer (40.0 kV, 30.0 mA) operating in θ-2θ reflection geometry and equipped with monochromated Cu $K\alpha_1$, λ = 1.5406 Å, X-rays, and a X'Celerator 1D detector. The sample was held on a glass plate at room temperature with data collected in intervals of 0.017° over the course of 14hrs. The weakly diffracting nature of the sample together with the small quantity available results in a powder diffraction pattern with only modest signal to noise on top of an undulating background, which is consistent with that observed from a pattern of the glass plate. Powder diffraction patterns were calculated from the structures of Structures 1, 2 and 3 using Crystal Diffract 6.5.5 (Figure S117).[4] The observed diffraction pattern was very similar to that of Structure 3, with all observed peaks and peak splitting being consistent with this being the dominant phase in the solid state. In contrast there were significant differences between the diffraction pattern of Structure 1 and the experimentally observed diffraction pattern suggesting this phase is not present. The moderate quality of the data and similarity of the powder diffraction pattern of Structure 1 and 2 prevents us from concluding that the sample is completely anhydrous but if present Structure 1 is likely a minority phase as several of the diffraction peaks expected from this phase are not observed.

Fluorescence and Transmission Microscopy Studies: All samples were visualized using an Olympus XI71 microscope with a PlanApo 100x OTIRFM-SP 1.49 NA lens attached to a PIFOC z-axis focus drive (Physik Instrumente, Karlsruhe, Germany), which was placed onto a ASI motorised stage (ASI, Eugene, OR). The objective lens, the environmental chamber along with the sample holder sustained the required temperature. The samples were illuminated using LED light sources (Cairn Research Ltd, Faversham, UK) using filters suitable for each sample (Chroma, Bellows Falls, VT). Metamorph software (Molecular Devices) software was used to control the settings and analyze the images, visualized using Zyla 5.5 (Andor) CMOS camera. 10 µl of each of the appropriate samples was pipetted onto the centre of an agarose pad. Coverslip was used to cover the pipetted sample and was secured in place. Each of the agarose pads was labelled. Filters used in the studies: GFP excitation 480 nm and emission 510 nm, DAPI excitation 360 and emission 460nm.

MIC_{50} studies[5]: *Preparation of luria broth media (LB):* Yeast extract (5 g), tryptone (10 g) and sodium chloride (10 g) were dissolved in milli-q H_2O (1000 mL) then divided into 400 mL bottles and autoclaved.
Preparation of luria broth (LB) Agar plates: Agar (6 g) was added to LB (400 mL) and autoclaved. Once cool, the LB agar was poured into sterile petri dishes under sterile conditions and allowed to set. LB plates were stored at 4 °C until use.
Preparation of bacterial plates: Sterile LB agar plates were streaked using the desired bacteria (MRSA USA300) then incubated in the 25 °C incubator overnight.
Preparation of antimicrobial compounds for MIC_{50} studies: Stock solutions of compounds **1**, **2** and **4** were prepared in a 1:19 EtOH:milli-q H_2O mixture the day of experiment. Eight concentrations of each compound/mixture were then prepared from the stock solution in the same solvent mixture.
Preparation of inoculum: A starter culture was produced through the inoculation of LB media (5 mL) with ≥ 4 single colonies of the desired bacteria under sterile conditions and incubated at 37 °C overnight. The following day, a subculture was made using LB (5 mL) and the starter culture (100 µL), then incubated at 37 °C until the culture had reached an optical density of 0.4 at 600 nm. Cellular density was then adjusted using sterile milli-q H_2O to equal a 0.5 McFarland Standard (10^7 – 10^8 cfu/mL), then a 1:10 dilution was carried out using sterile milli-q H_2O (900 µL) and the McFarland adjusted suspension (100 µL). A final dilution (1:100) was carried out on the 1:10 suspension (150 µL) using LB (14.85 mL) before use (10^5 cfu/mL).
Preparation of 96 well Microplate: The 1:100 suspension (90 µL) was pipetted into the desired wells under sterile conditions, then solutions containing **1**, **2**, **4** or 1:1 mixtures of **1**, **2** and **4** (90 µL) were added to the wells to equal a total volume of 180 µL. The plates were sealed using Parafilm, then incubated at 37 °C in a microplate reader for 18-25 hours. An absorbance reading was taken at 600nm every 15 minutes. Each experiment was repeated three times on two different days giving six repetitions in total.
Calculation of MIC_{50}: Growth curves were plotted using the average of the six comparative absorbance readings in Microsoft Excel. The MIC_{50} value was determined by plotting the average absorbance reading obtained at 900 minutes for each compound concentration in Origin. The resulting curve was [normalized] and fitted using the Boltzmann fit, and the equation from this fit was used to calculate the MIC_{50}.

Chemical Structures

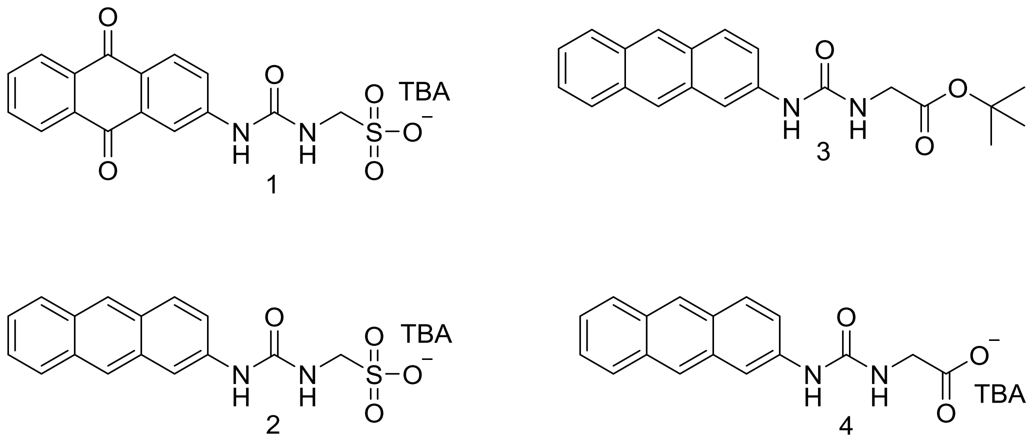

Figure S1. Chemical structures of compounds 1-5. TBA = Tetrabutylammonium.

Chemical Synthesis

Tetrabutylammonium (TBA) aminomethanesulfonate: TBA hydroxide (1N) in methanol (1.71 mL) was added to aminomethanesulfonic acid (0.19 g, 1.71 mM) at room temperature and taken to dryness. Assumed Yield 100 % (0.60 g, 1.71 mM).

Compound 1: A solution of 2-aminoanthraquinone (0.67 g, 3.01 mM) and triphosgene (0.445 g, 1.50 mM) in ethyl acetate (30 mL) was heated at reflux for four hours. TBA aminomethanesulfonate (1.06 g, 3.01 mM) in ethyl acetate (10 mL) was then added to the reaction mixture, which was then heated at reflux overnight. The resultant mixture was filtered and the solid isolated was dissolved in methanol (15 mL). Any remaining solid was removed by filtration and the filtrate taken to dryness. The resultant solid was re-dissolved in chloroform (20 mL) and washed with water (1 x 10 mL). The organic layer was then taken to dryness to give the final product as an orange solid (0.411 g, 0.68 mM). Yield: 23 %; mp: 145 °C; ^{1}H NMR (400 MHz, DMSO-d_6): δ:9.68 (s,1H,NH), 8.25 (d, J = 2.12 Hz, 1H), 7.97 – 7.86 (m, 3H), 7.76 – 7.73 (m, 2H), 7.59 (d, J = 8.48 Hz, 1H), 7.51 (br t, 1H, NH), 4.03 (d, J = 5.68 Hz, 2H), 3.17 – 3.13 (m, 8H), 1.59 – 1.51 (m, 8H), 1.34 – 1.25 (m, 8H), 0.92 (t, J = 7.28 Hz, 12H), ^{13}C{^{1}H} NMR (100 MHz, DMSO-d_6): δ:182.8 (C=O), 181.3 (C=O), 154.4 (C=O), 147.0 (ArC), 134.6 (ArCH), 134.2 (ArCH), 134.1 (ArC), 133.3 (ArC), 133.3 (ArC), 128.3 (ArCH), 126.8 (ArCH), 126.7 (ArCH), 126.1 (ArC), 122.8 (ArCH), 114.3 (ArCH), 58.0 (CH_2), 56.4 (CH_2), 23.5 (CH_2), 19.7 (CH_2), 13.9 (CH_3); IR (film): v (cm^{-1}) = 3269 (NH stretch), 1670, 1209, 1177, 853; HRMS for the sulfonate-urea ion ($C_{16}H_{13}N_2O_6S$) (ESI$^-$): m/z: act: 359.0339 [M]$^-$ cal: 359.0343 [M]$^-$.

Compound 2: This compound was synthesised in line with previously published methods.[6] The proton spectrum matches previously reported data. ^{1}H NMR (400 MHz, DMSO-d_6): δ:9.14 (s,1H,NH), 8.40 (s,1H), 8.29 (s,1H), 8.22 (s,1H), 7.99 – 7.93 (m,3H), 7.45 – 7.37 (m,3H), 6.88 (t, J = 5.88 Hz, 1H), 4.00 (d, J = 5.92 Hz, 2H), 3.14 – 3.10 (m, 8H), 1.57 – 1.49 (m, 8H), 1.33 – 1.23 (m, 8H), 0.92 (t, J = 7.28 Hz, 12H).

Compound 3: A solution of 2-aminoanthracene (0.50 g, 2.58 mM) and triphosgene (0.38 g, 1.29 mM) in ethyl acetate (35 mL) was heated at reflux for 4 hours. *Tert*-butyl 2-aminoacetate (0.34 mL, 2.58 mM) was then added to the reaction mixture. This mixture was then heated at reflux overnight. The resultant mixture was then filtered ant the final product isolated as a grey solid (0.55 g, 1.57mM). Yield: 61.0%; mp: > 200 °C; ^{1}H NMR (400 MHz, DMSO-d_6): δ:9.09 (s,1H, NH), 8.44 (s, 1H), 8.34 (s, 1H), 8.22 (s,1H), 8.01 – 7.97 (m, 3H), 7.47 – 7.39 (m, 3H), 6.53 (t, J = 5.88 Hz, 1H), 3.83 (d, J = 5.88 Hz, 2H), 1.44 (s, 9H);^{13}C{^{1}H} NMR (100 MHz, DMSO-d_6): δ:170.4 (C=O), 155.8 (C=O), 137.8 (ArC), 132.6 (ArC), 132.2 (ArC), 130.4 (ArC), 129.2 (ArCH), 128.5 (ArCH), 128.5 (ArC), 128.0 (ArCH), 126.2 (ArCH), 126.0 (ArCH), 125.0 (ArCH), 124.6 (ArCH), 121.3 (ArCH), 111.5 (ArCH), 81.1(C) , 42.5 (CH_2), 28.3 (CH_3); IR (film): v (cm^{-1}) = 3308 (NH stretch), 1653, 1244, 1161, 889; HRMS ($C_{21}H_{22}N_2O_3$) (ESI$^-$): m/z: act: 349.1628 [M]$^-$ cal: 349.1557 [M]$^-$.

Compound 4: Compound **3** (0.90 g, 2.59 mM) was dissolved in dichloromethane (50 mL) and trifluoroacetic acid (8 mL) and stirred for 1 hour. The mixture was then washed with sodium hydroxide (50 mL, 6 M) and the resultant green solid (0.70 g) isolated by filtration and suspended in methanol (20 mL). To this solution was added TBA hydroxide (1N) in methanol (2.32 mL). This solution was passed through a Biotage SCX(II) column with methanol and the TBA:carboxylate ratio equilibrated through the further addition of TBA hydroxide (1N) in methanol. The pure product was isolated as a yellow-green solid (0.78 g, 1.46 mM). Yield: 56 %; mp: > 200 °C; ^{1}H NMR (400 MHz, DMSO-d_6): δ:9.88 (s, 1H, NH), 8.40 (s, 1H), 8.29 (s, 2H), 8.00 – 7.93 (m, 3H), 7.55 (d, J = 9.08 Hz, 1H), 7.45 – 7.37 (m, 2H), 6.98 (s, 1H, NH), 3.64 (d, J = 4.36 Hz, 2H), 3.15 – 3.11 (m, 8H), 1.57 – 1.50 (m, 8H), 1.33 – 1.24 (m, 8H), 0.92 (t, J = 7.24 Hz, 12H);^{13}C{^{1}H} NMR (100 MHz, DMSO-d_6): δ:172.6 (CO), 155.8 (CO), 138.8 (ArC), 132.9 (ArC), 132.2 (ArC), 130.2 (ArC), 129.0 (ArCH), 128.5 (ArCH), 128.4 (ArC), 128.0 (ArCH), 126.2 (ArCH), 125.8 (ArCH), 124.7 (ArCH), 124.3 (ArCH), 121.7 (ArCH), 110.6 (ArCH), 58.0 (CH_2), 44.2 (CH_2), 23.5 (CH_2), 19.7 (CH_2), 14.0 (CH_3);IR (film): v (cm^{-1}) = 3332.99 (NH stretch), 1684, 1225, 1163, 889; HRMS for the carboxylate-urea ion ($C_{17}H_{13}N_2O_3^-$) (ESI$^-$): m/z: act: 293.0932 [M]$^-$ cal: 293.0923 [M]$^-$.

NMR Characterization

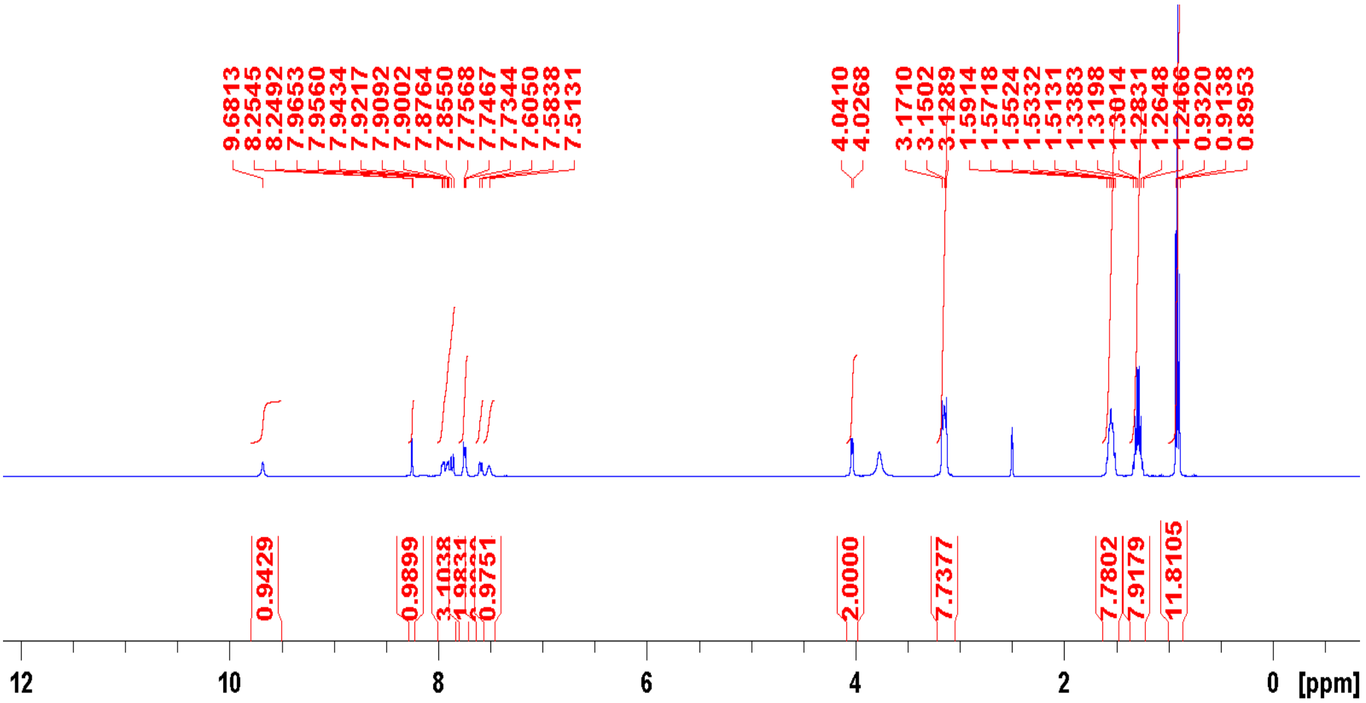

Figure S2. A ^{1}H NMR spectrum of compound **1** in DMSO-d_6 at 298 K.

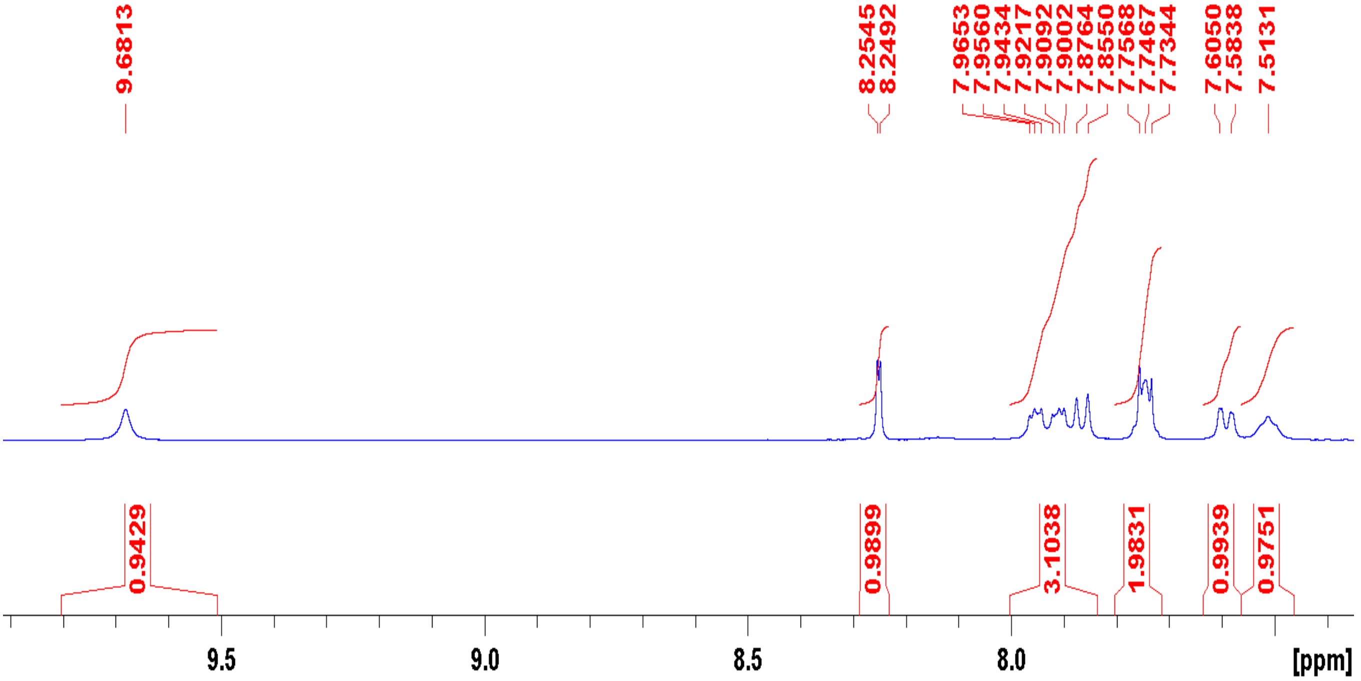

Figure S3. A zoomed in ^{1}H NMR spectrum of compound **1** in DMSO-d_6 at 298 K.

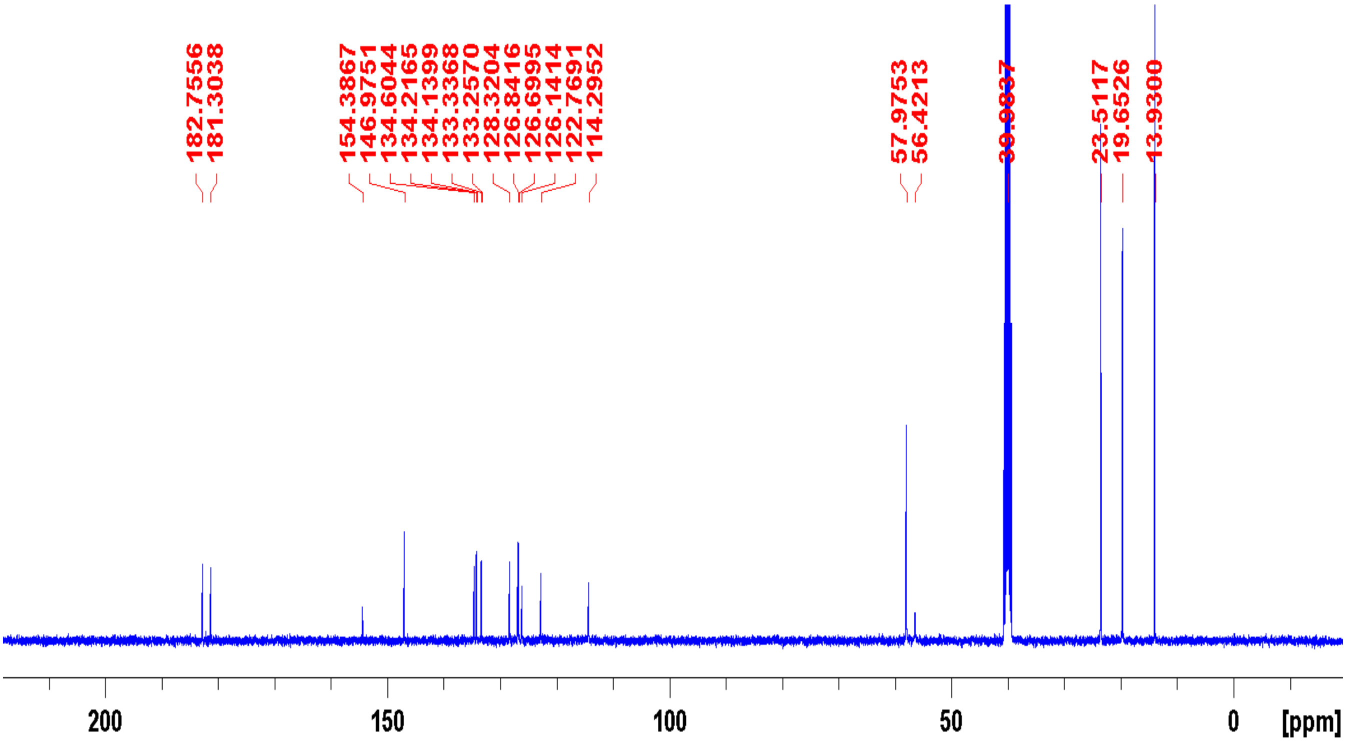

Figure S4. A ^{13}C NMR spectrum of compound **1** in DMSO-d_6 at 298 K.

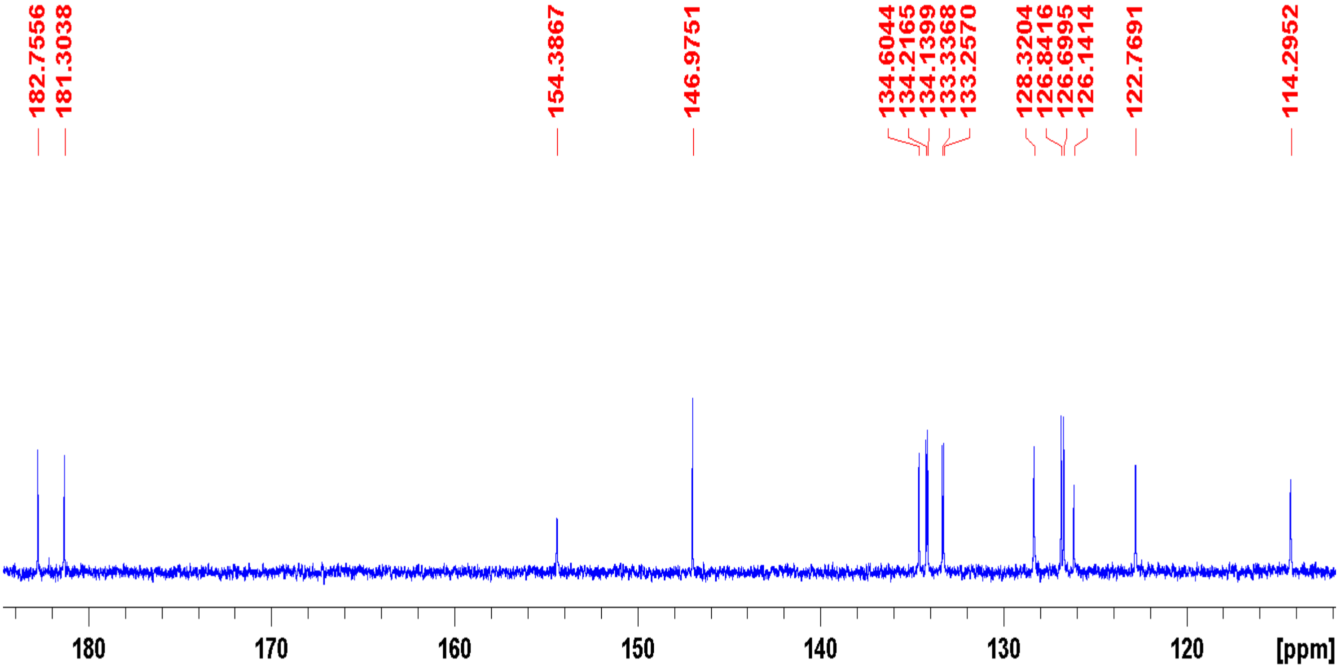

Figure S5. A zoomed in ^{13}C NMR spectrum of compound **1** in DMSO-d_6 at 298 K.

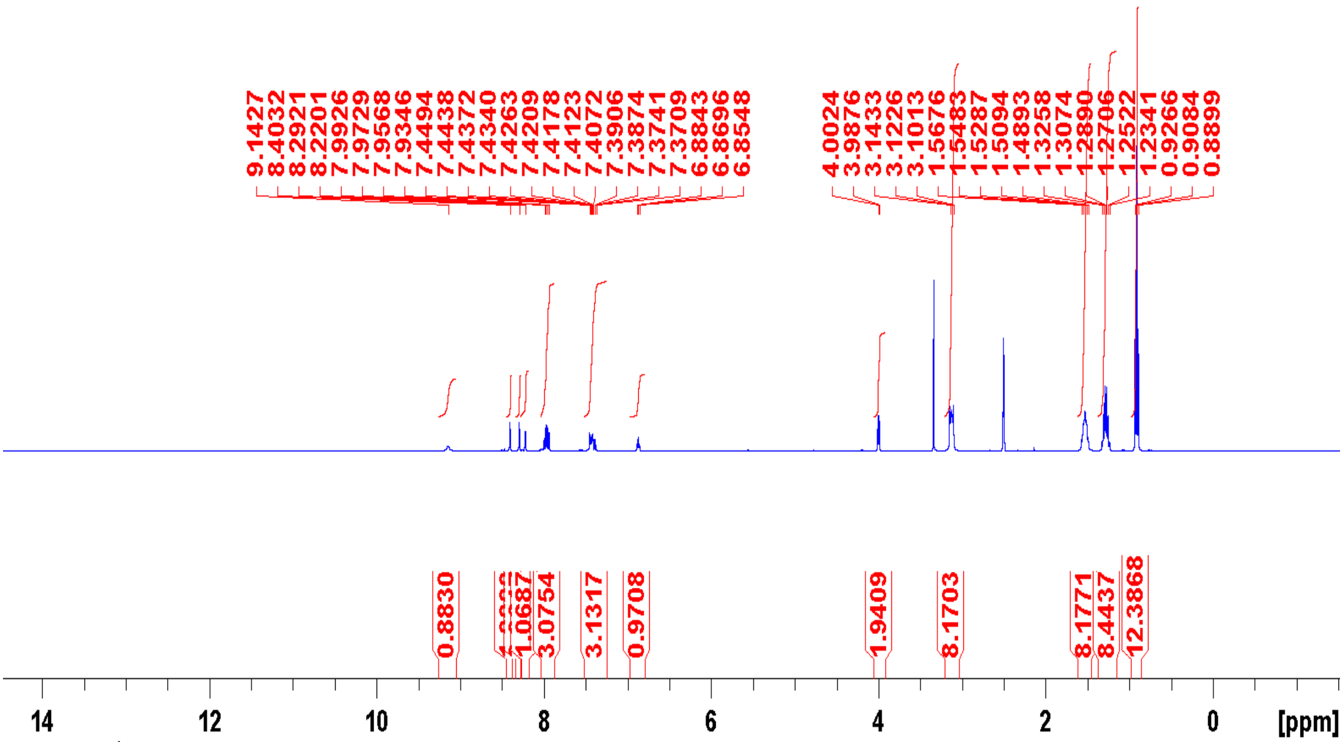

Figure S6. A ^{1}H NMR spectrum of compound **2** in DMSO-d_6 at 298 K.

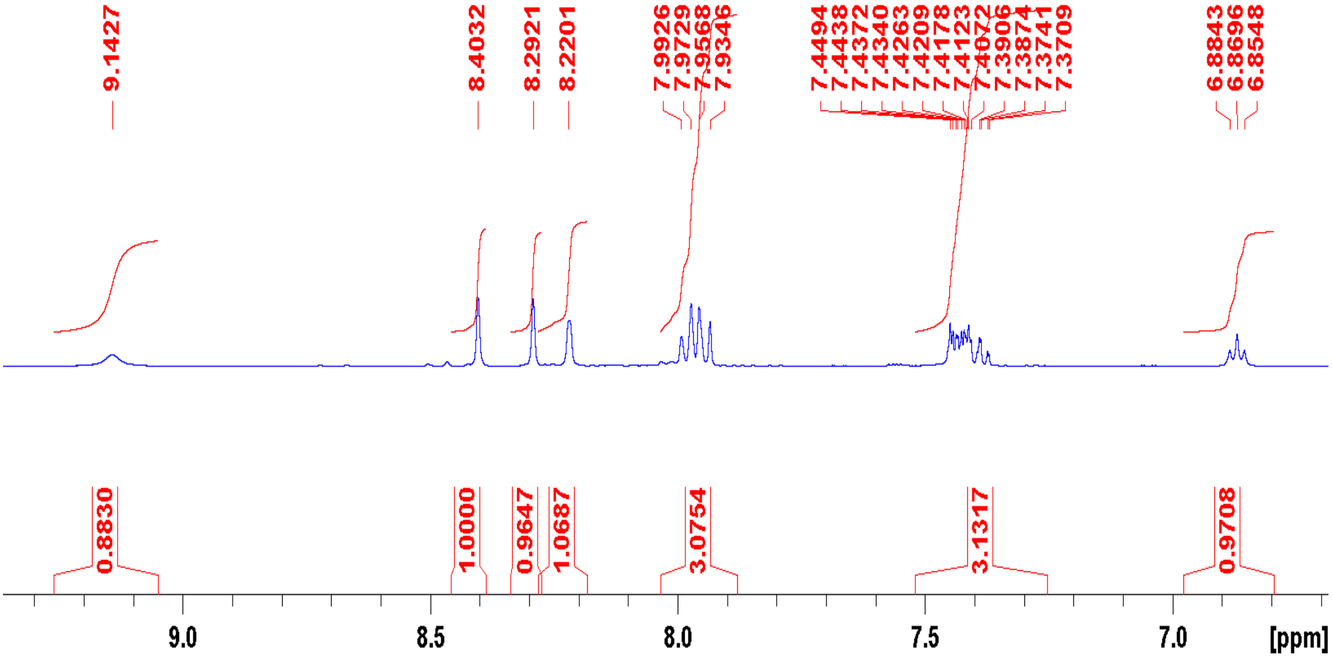

Figure S7. A zoomed in ^{1}H NMR spectrum of compound **2** in DMSO-d_6 at 298 K.

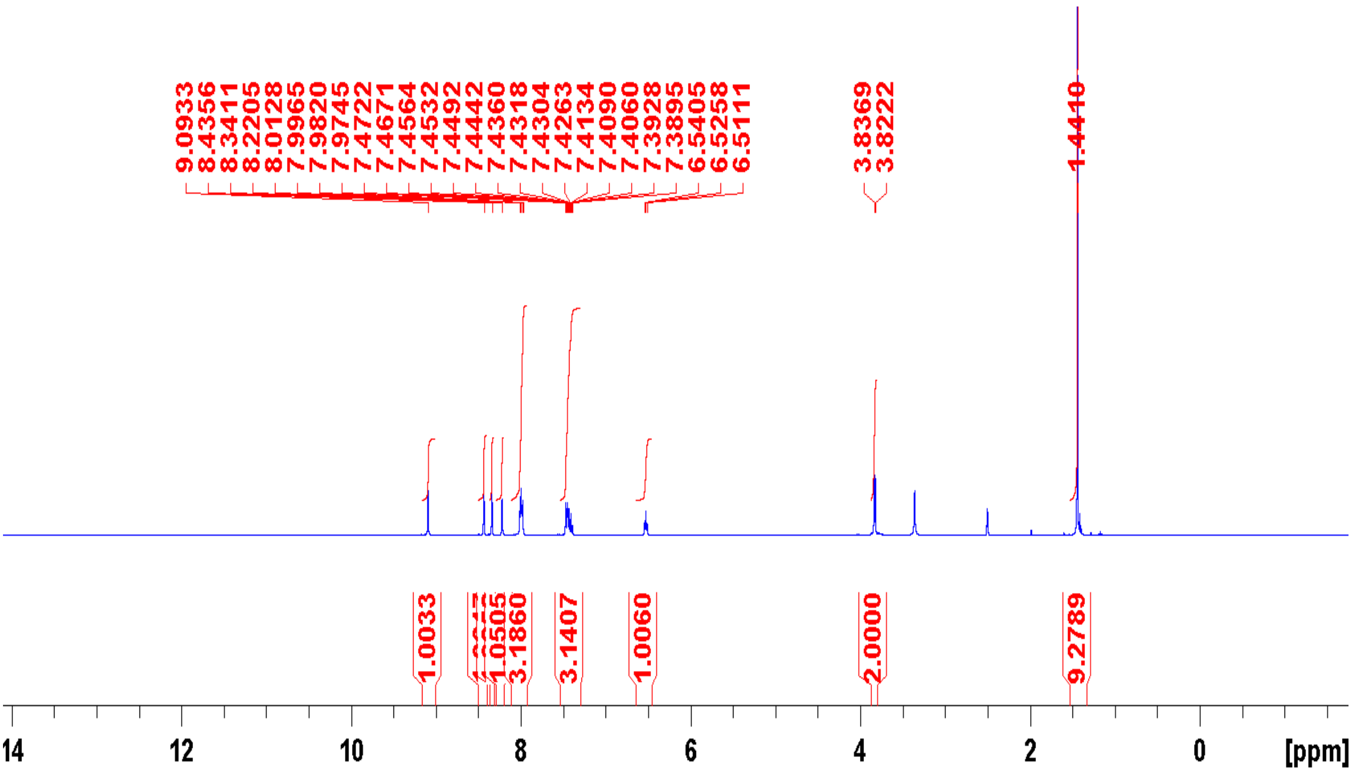

Figure S8. A ^{1}H NMR spectrum of compound **3** in DMSO-d_6 at 298 K.

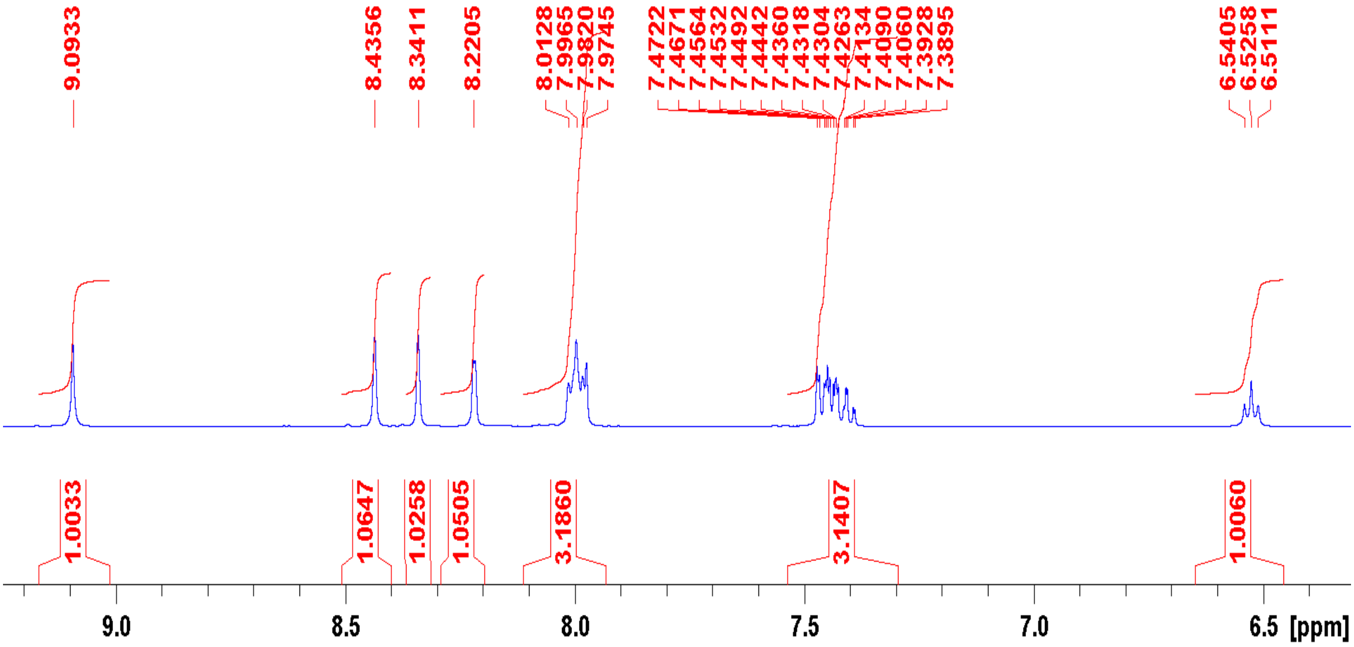

Figure S9. A zoomed in ^{1}H NMR spectrum of compound **3** in DMSO-d_6 at 298 K.

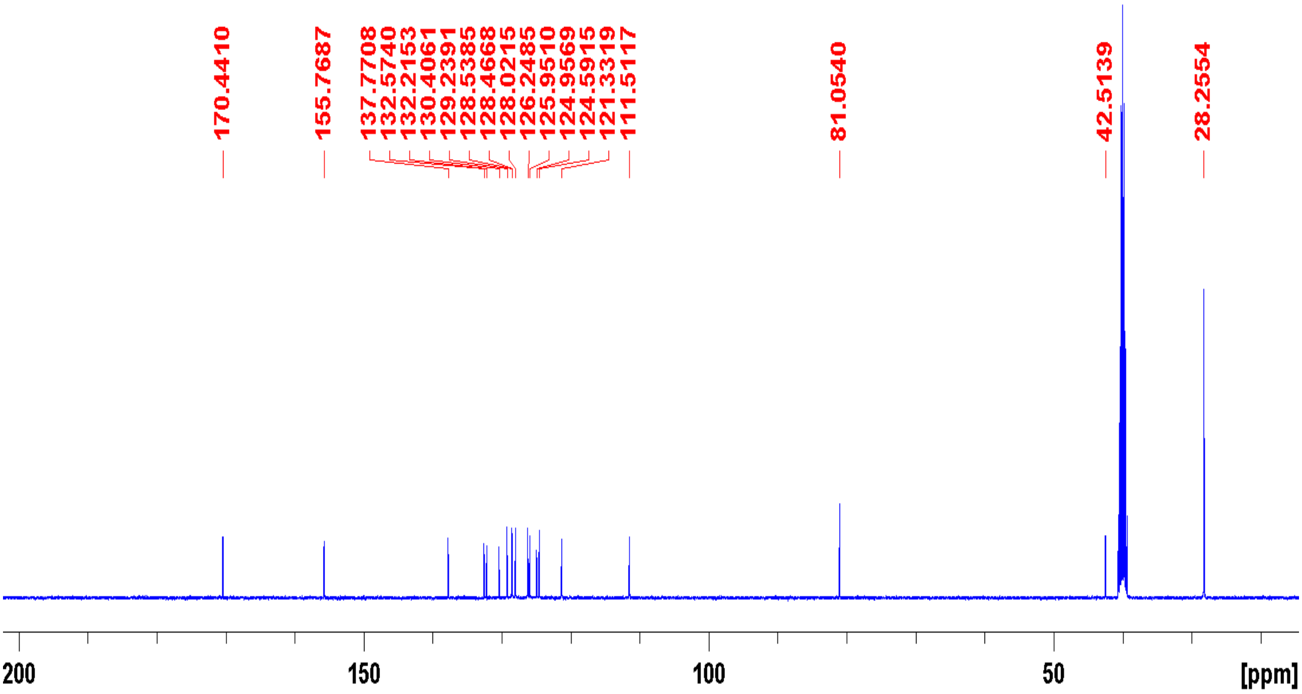

Figure S10. A ^{13}C NMR spectrum of compound **3** in DMSO-d_6 at 298 K.

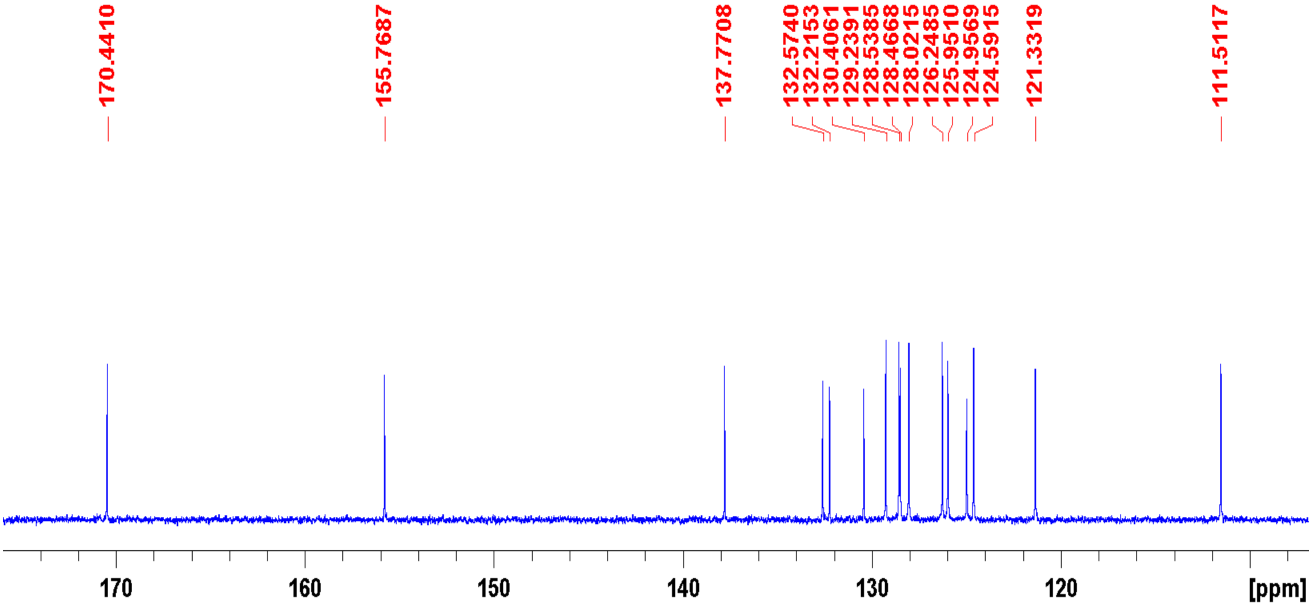

Figure S11. A zoomed in ^{13}C NMR spectrum of compound **3** in DMSO-d_6 at 298 K.

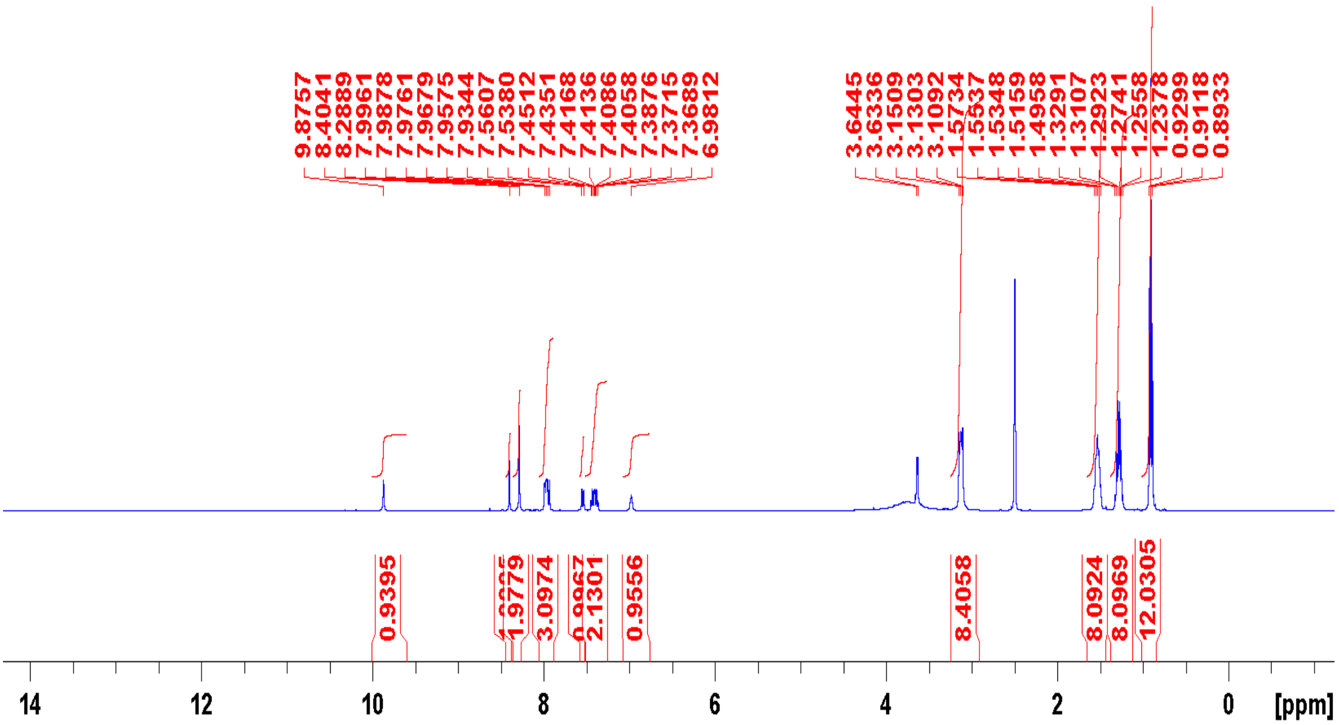

Figure S12. A ^{1}H NMR spectrum of compound **4** in DMSO-d_6 at 298 K.

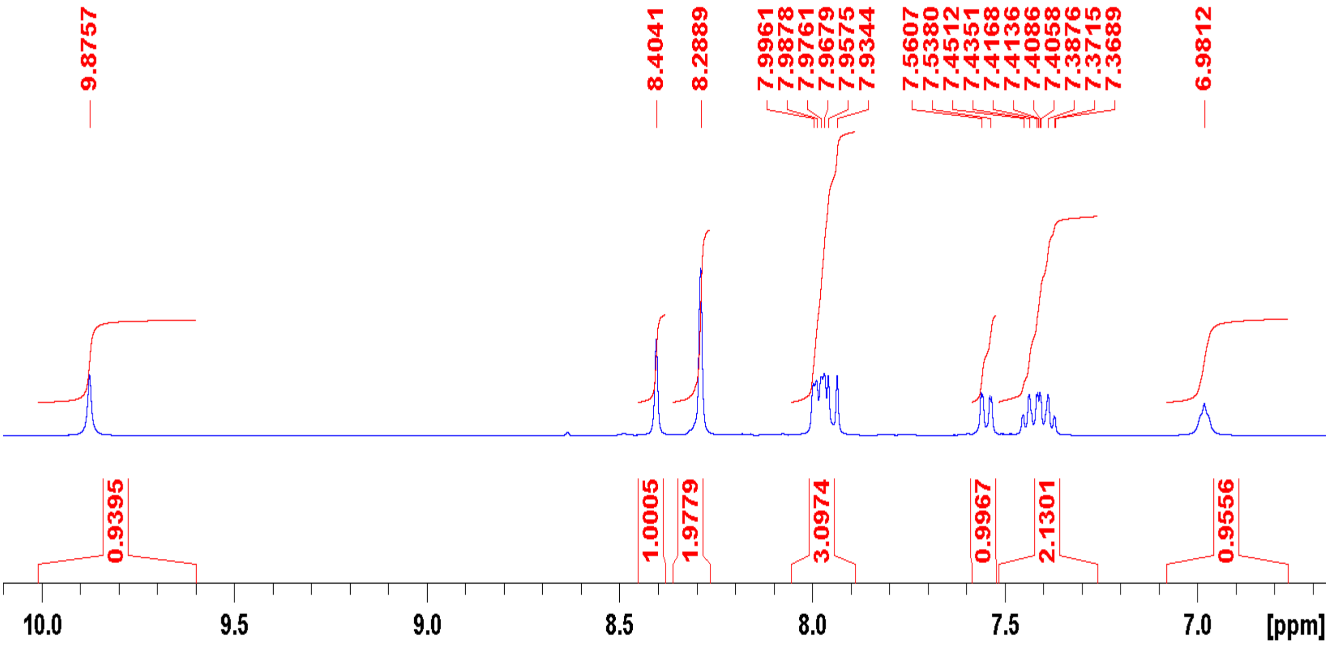

Figure S13. A zoomed in ^{1}H NMR spectrum of compound **4** in DMSO-d_6 at 298 K.

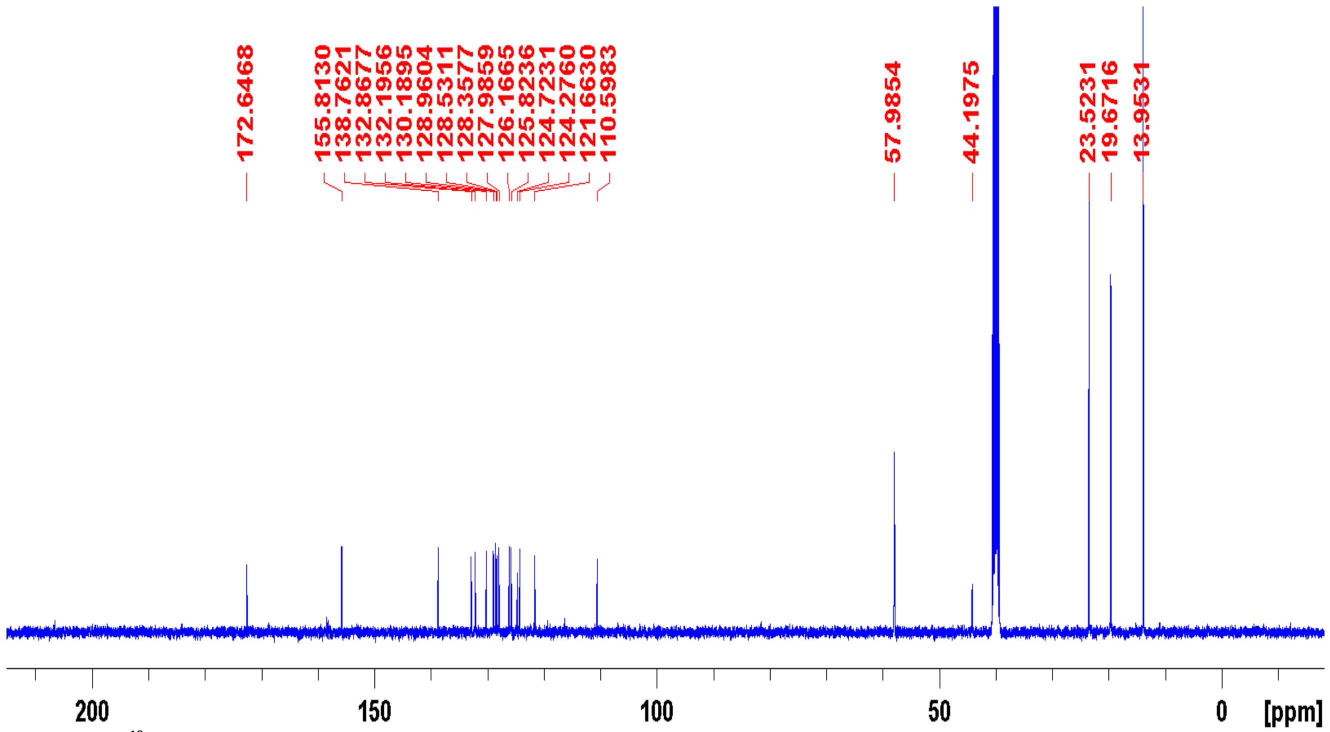

Figure S14. A ^{13}C NMR spectrum of compound **4** in DMSO-d_6 at 298 K.

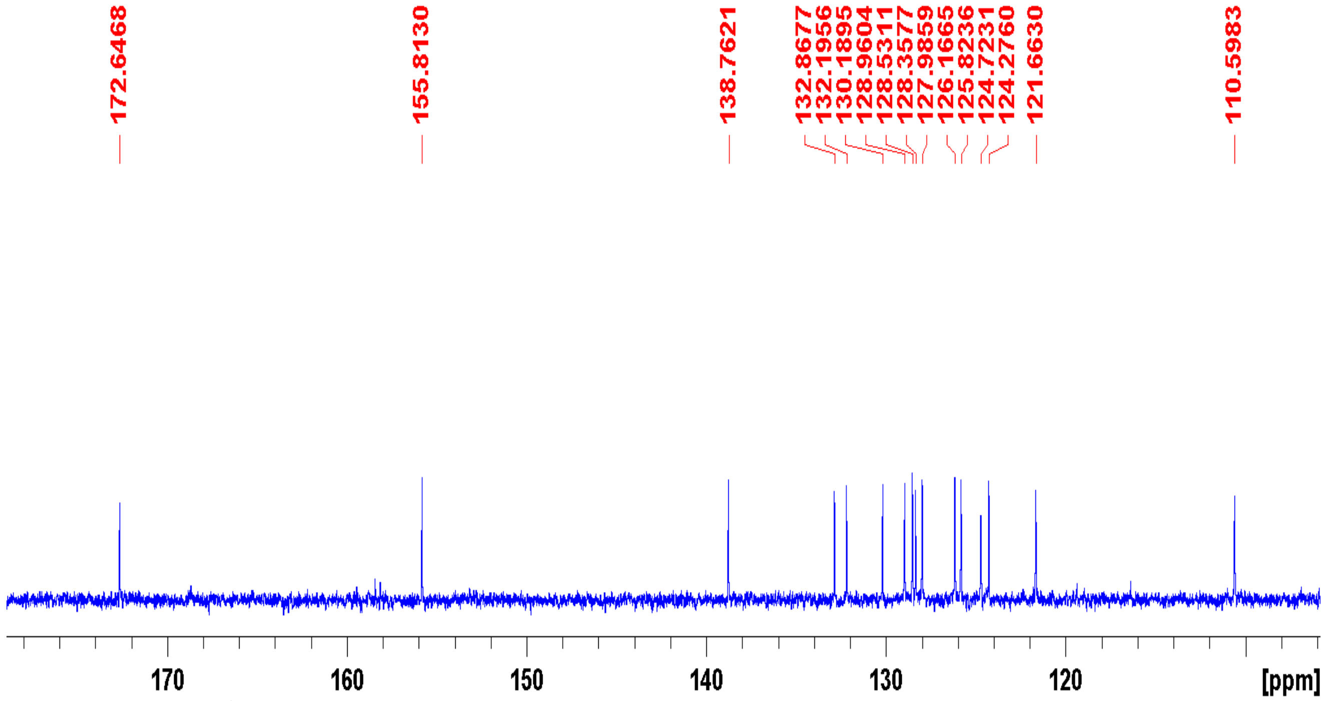

Figure S15. A zoomed in ^{13}C NMR spectrum of compound **4** in DMSO-d_6 at 298 K.

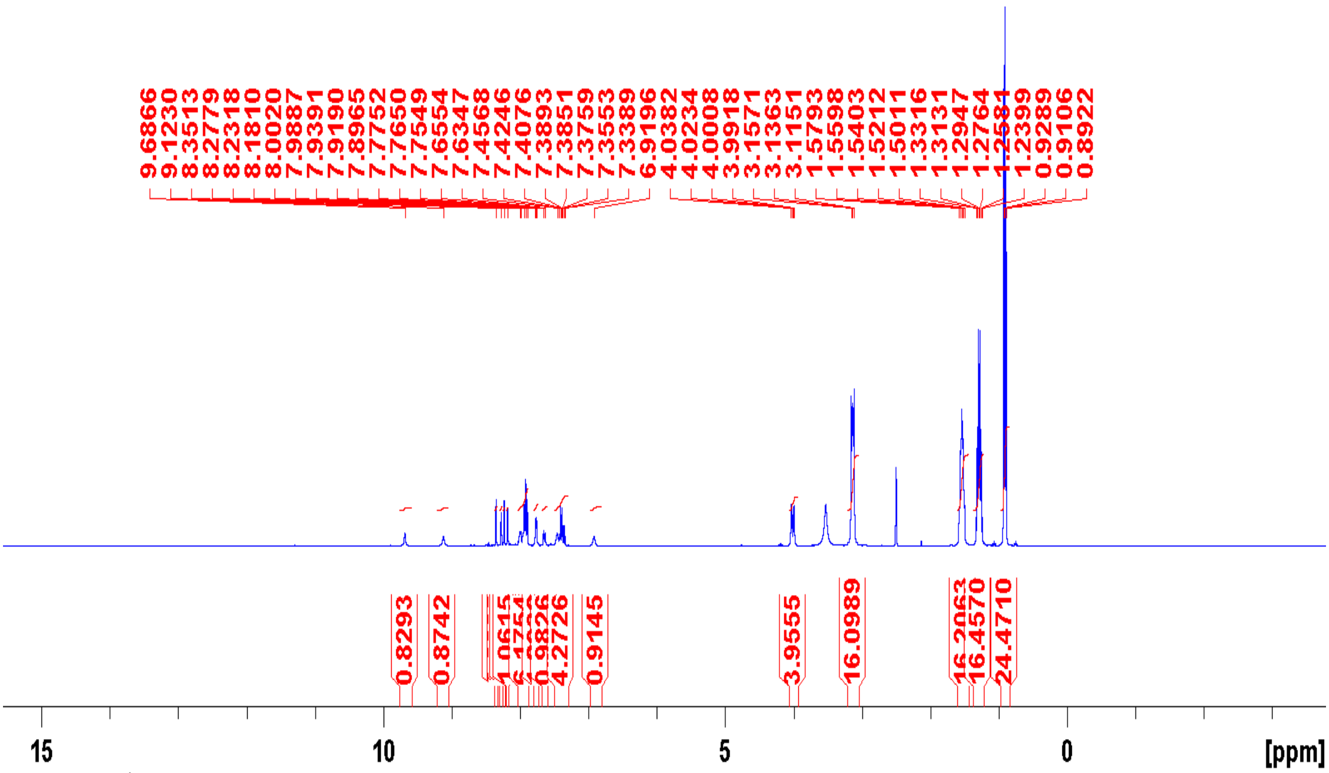

Figure S16. A ^{1}H NMR spectrum of compounds **1** and **2** (1:1) in DMSO-d_6 at 298 K.

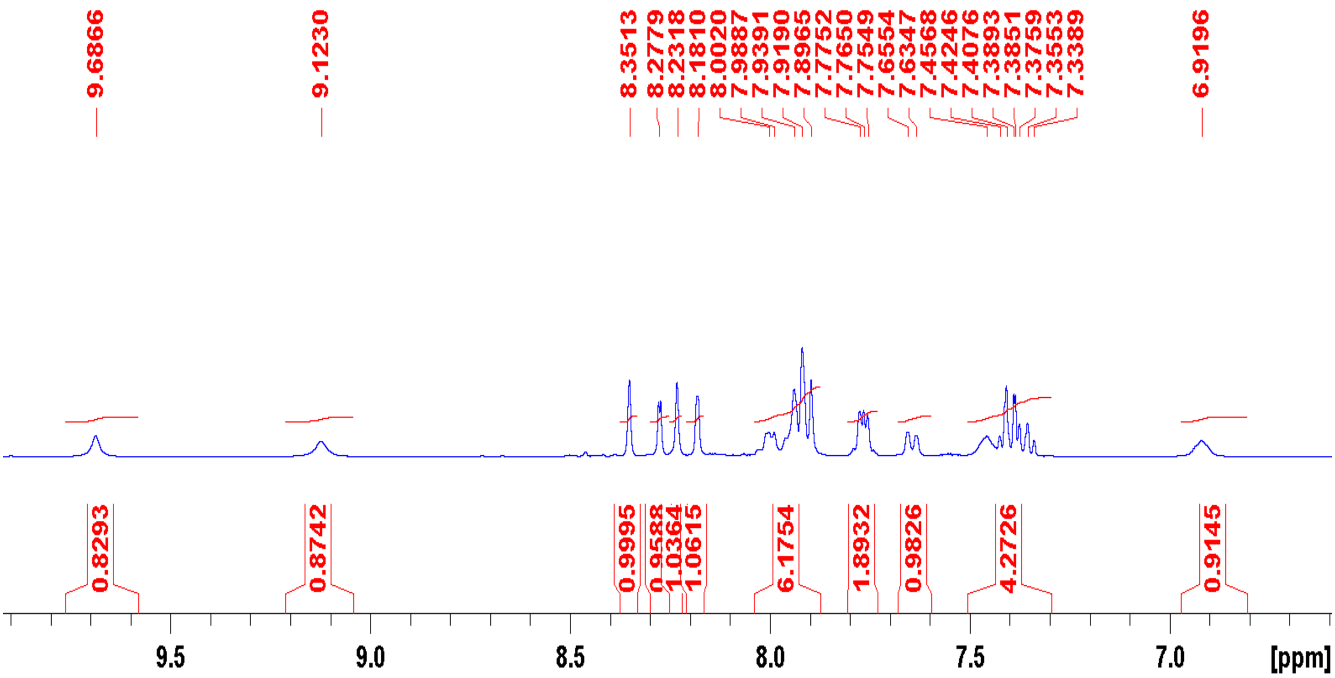

Figure S17. A zoomed in ^{1}H NMR spectrum of compounds **1** and **2** (1:1) in DMSO-d_6 at 298 K.

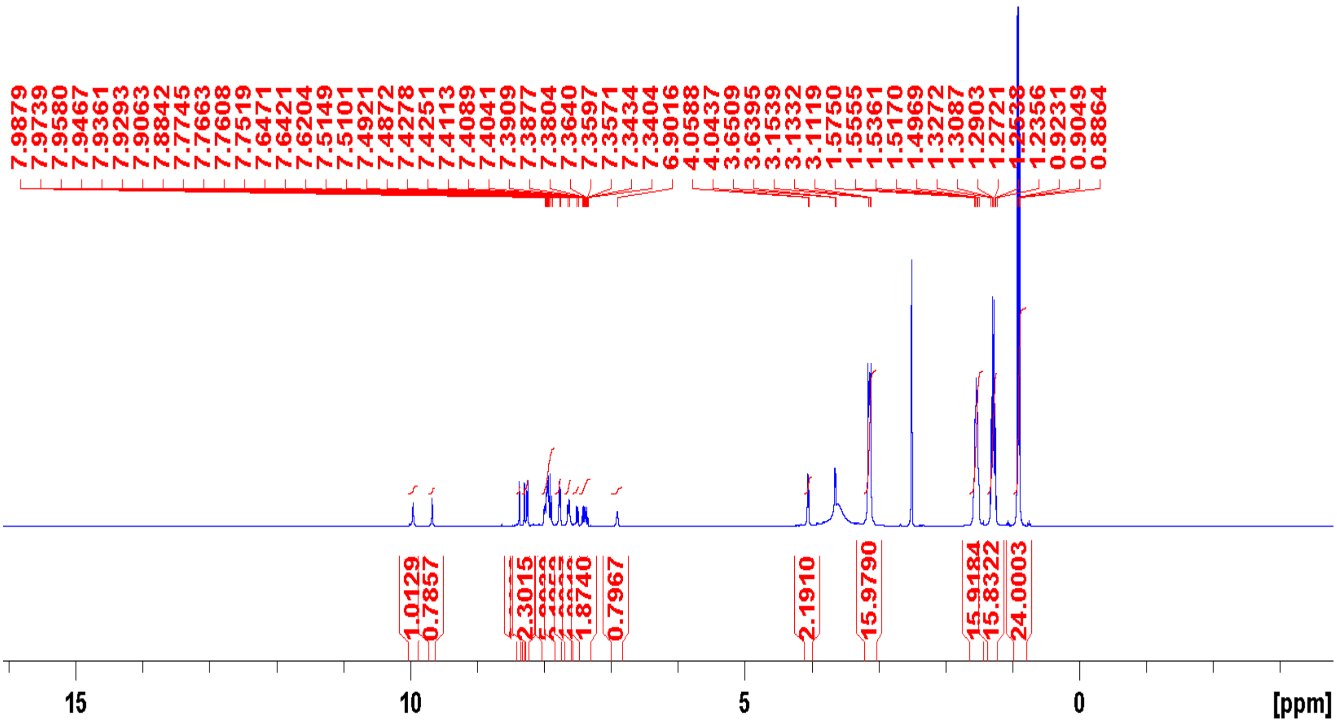

Figure S18. A ^{1}H NMR spectrum of compounds **1** and **4** (1:1) in DMSO-d_6 at 298 K.

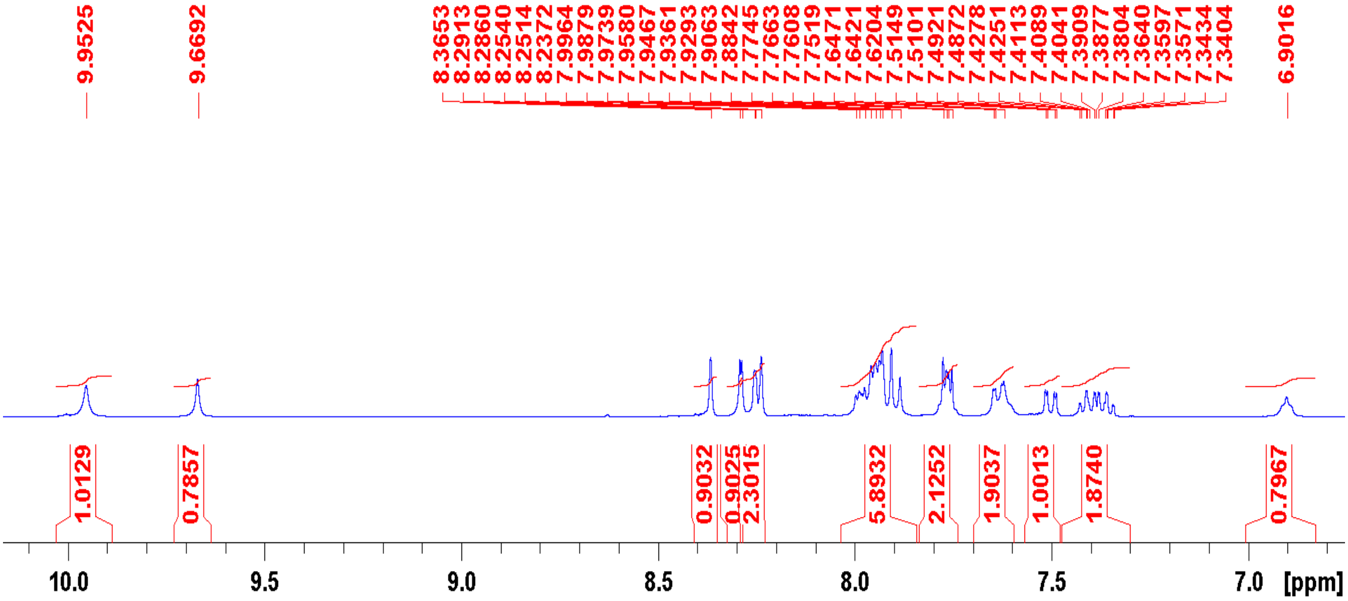

Figure S19. A zoomed in ^{1}H NMR spectrum of compounds **1** and **4** (1:1) in DMSO-d_6 at 298 K.

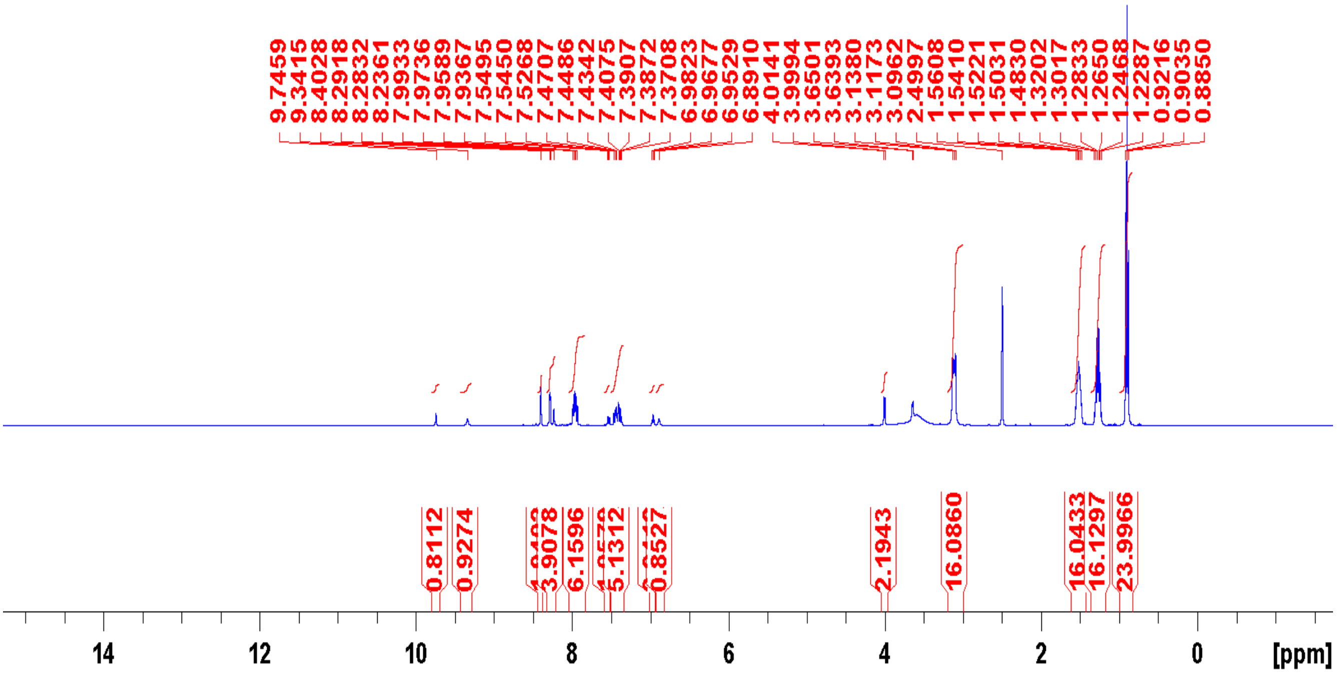

Figure S20. A ^{1}H NMR spectrum of compounds **2** and **4** in DMSO-d_6 at 298 K.

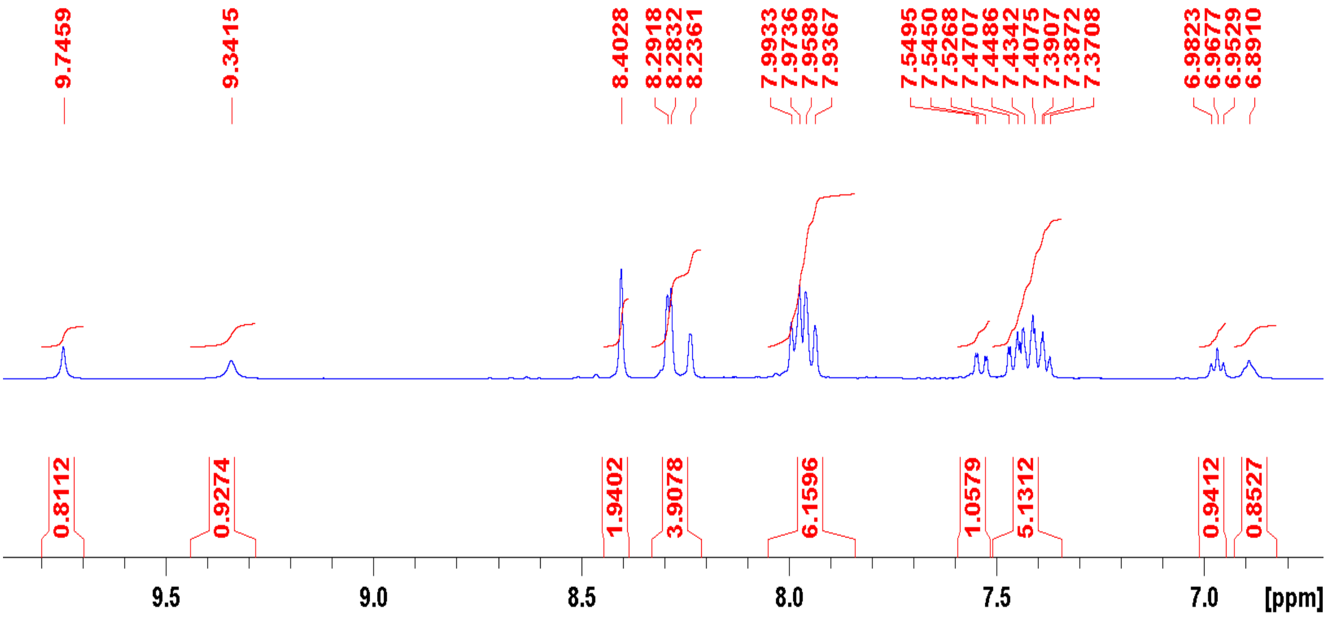

Figure S21. A zoomed in ^{1}H NMR spectrum of compounds **2** and **4** (1:1) in DMSO-d_6 at 298 K.

Mass Spectrometry Results

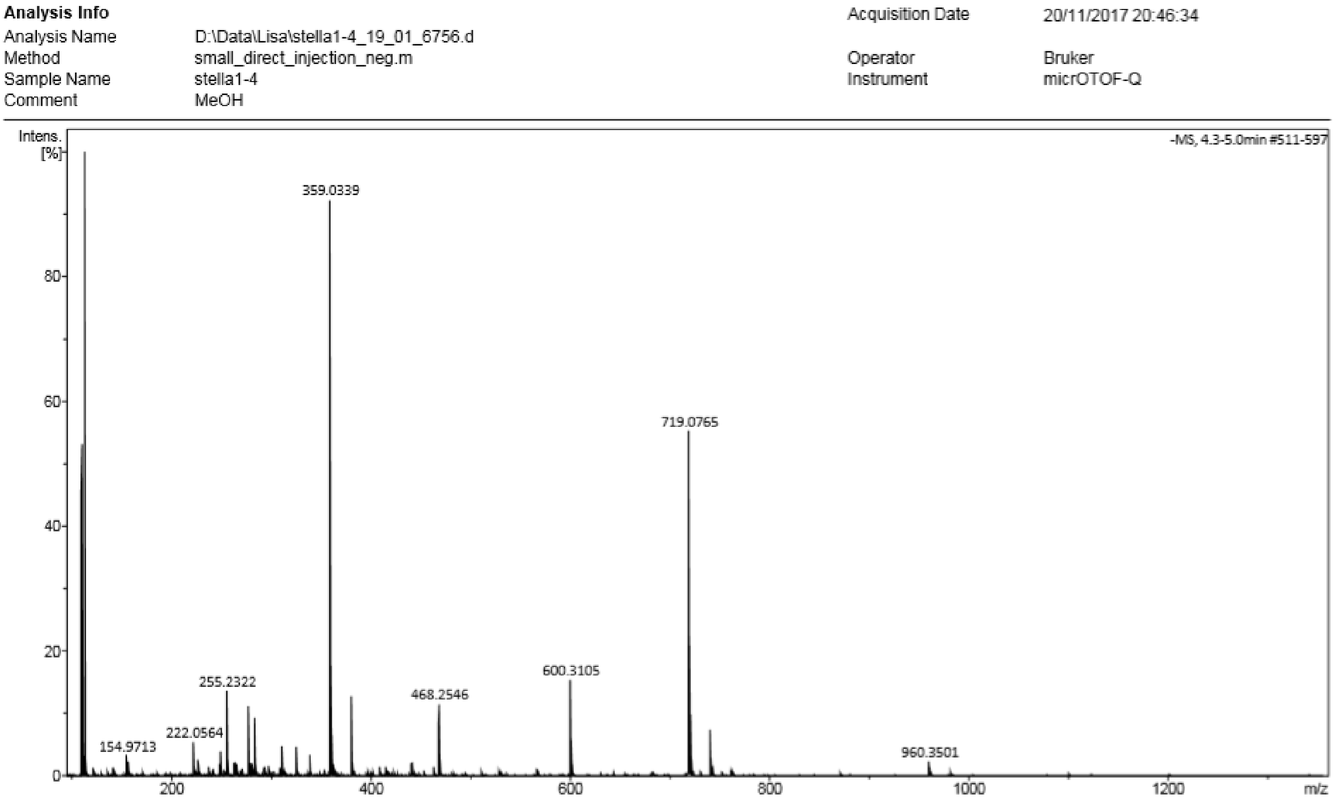

Figure S22. A high-resolution mass spectrum (ESI –ve) obtained for compound **1** in methanol.

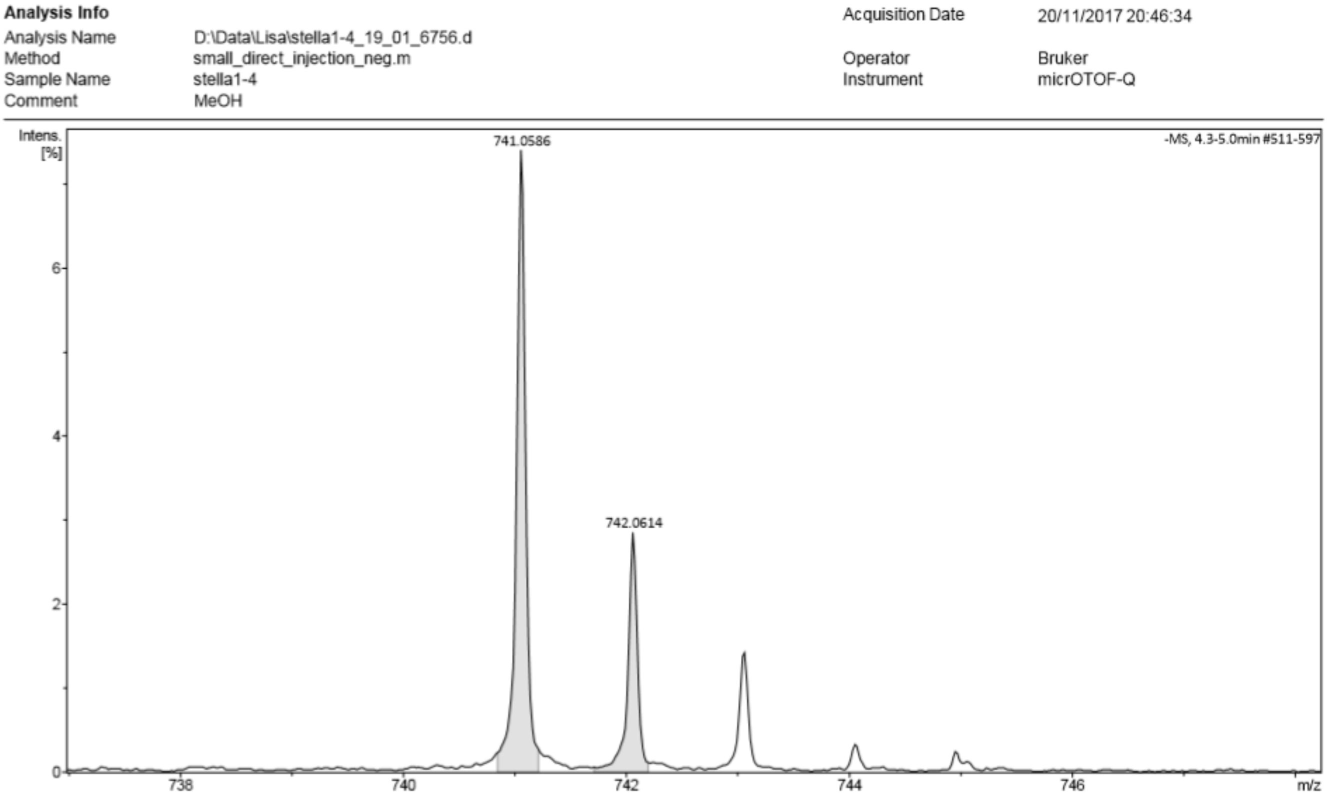

Figure S23. A high-resolution mass spectrum (ESI –ve) obtained for compound **1** in methanol.

Acquisition Parameter

Source Type	ESI	Ion Polarity	Negative	Set Nebulizer	0.4 Bar
Focus	Not active	Set Capillary	3500 V	Set Dry Heater	180 °C
Scan Begin	100 m/z	Set End Plate Offset	-100 V	Set Dry Gas	4.0 l/min
Scan End	1350 m/z	Set Collision Cell RF	150.0 Vpp	Set Divert Valve	Source

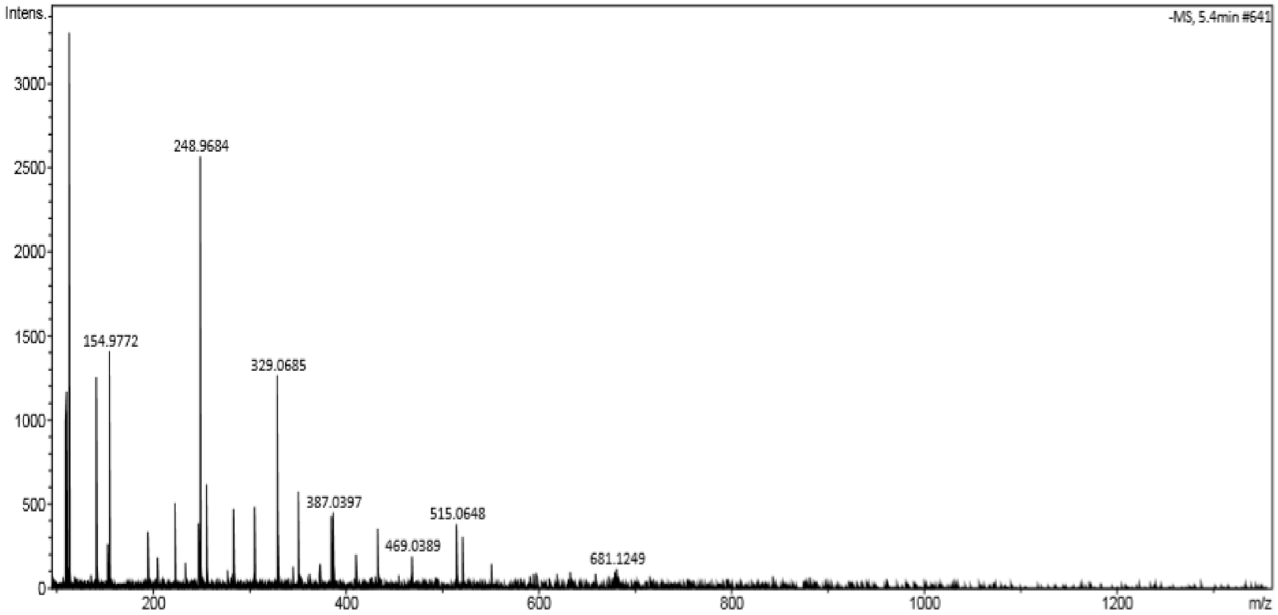

Figure S24. A high-resolution mass spectrum (ESI –ve) obtained for compound **2** in methanol.

Analysis Info

Analysis Name	D:\Data\Lisa\stella 3-1_19_01_7345.d	Acquisition Date	07/06/2018 15:00:24
Method	small_direct_injection_neg.m	Operator	Bruker
Sample Name	stella 3-1	Instrument	micrOTOF-Q
Comment	MeOH		

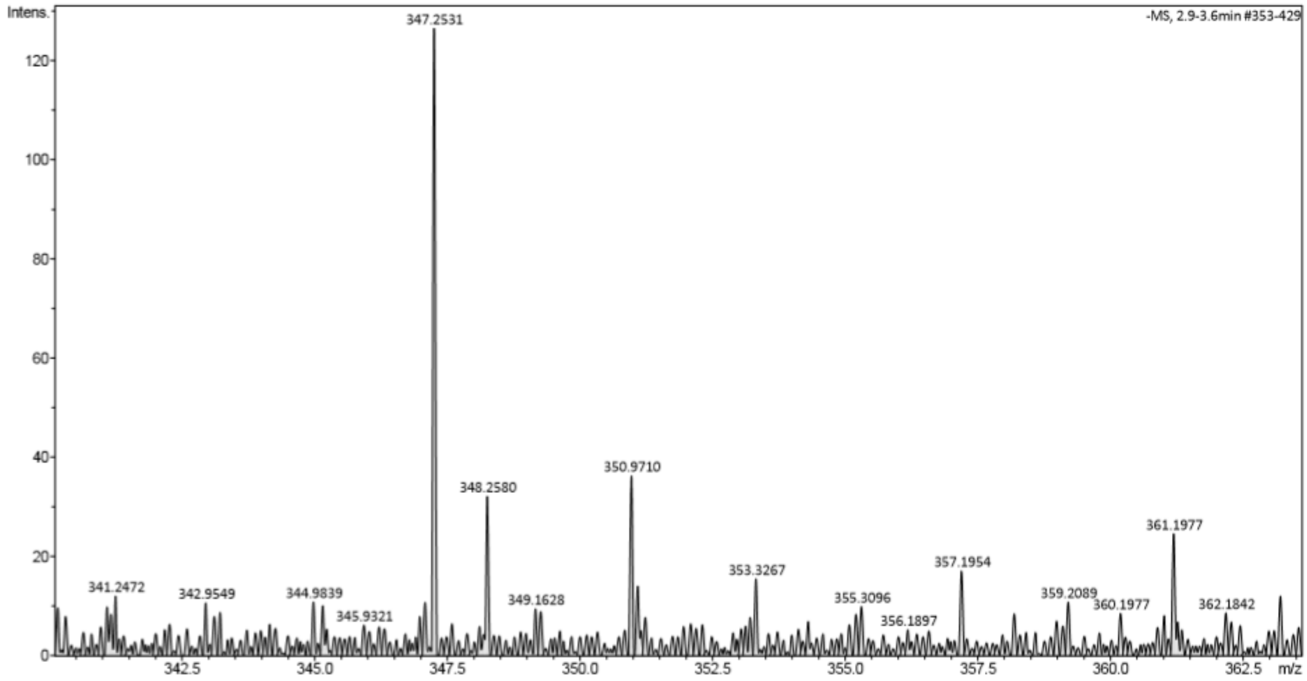

Figure S25. A high-resolution mass spectrum (ESI –ve) obtained for compound **3** in methanol.

Analysis Info

Analysis Name	D:\Data\Lisa\stella 3-3_16_01_7342.d	Acquisition Date	07/06/2018 14:26:49
Method	small_direct_injection_neg.m	Operator	Bruker
Sample Name	stella 3-3	Instrument	micrOTOF-Q
Comment	MeOH		

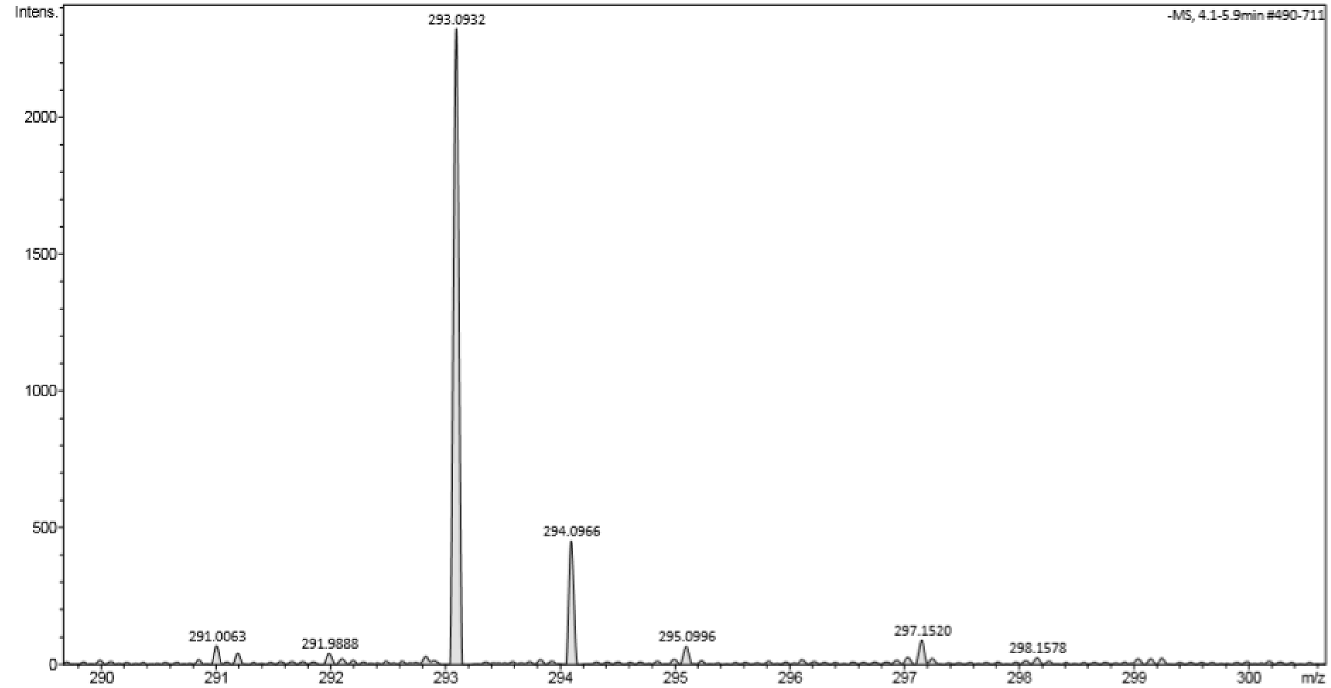

Figure S26. A high-resolution mass spectrum (ESI –ve) obtained for compound **4** in methanol.

Analysis Info

Analysis Name	D:\Data\Lisa\stella 3-3_16_01_7342.d	Acquisition Date	07/06/2018 14:26:49
Method	small_direct_injection_neg.m	Operator	Bruker
Sample Name	stella 3-3	Instrument	micrOTOF-Q
Comment	MeOH		

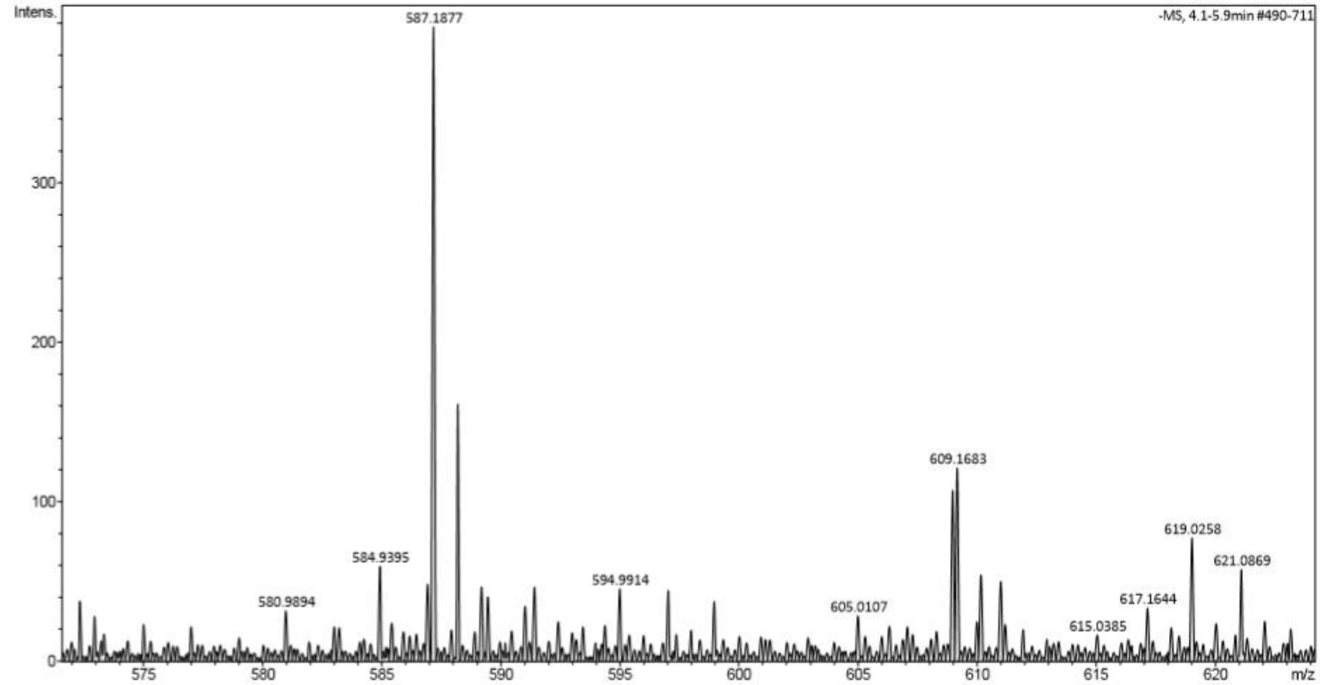

Figure S27. A high-resolution mass spectrum (ESI –ve) obtained for compound **4** in methanol.

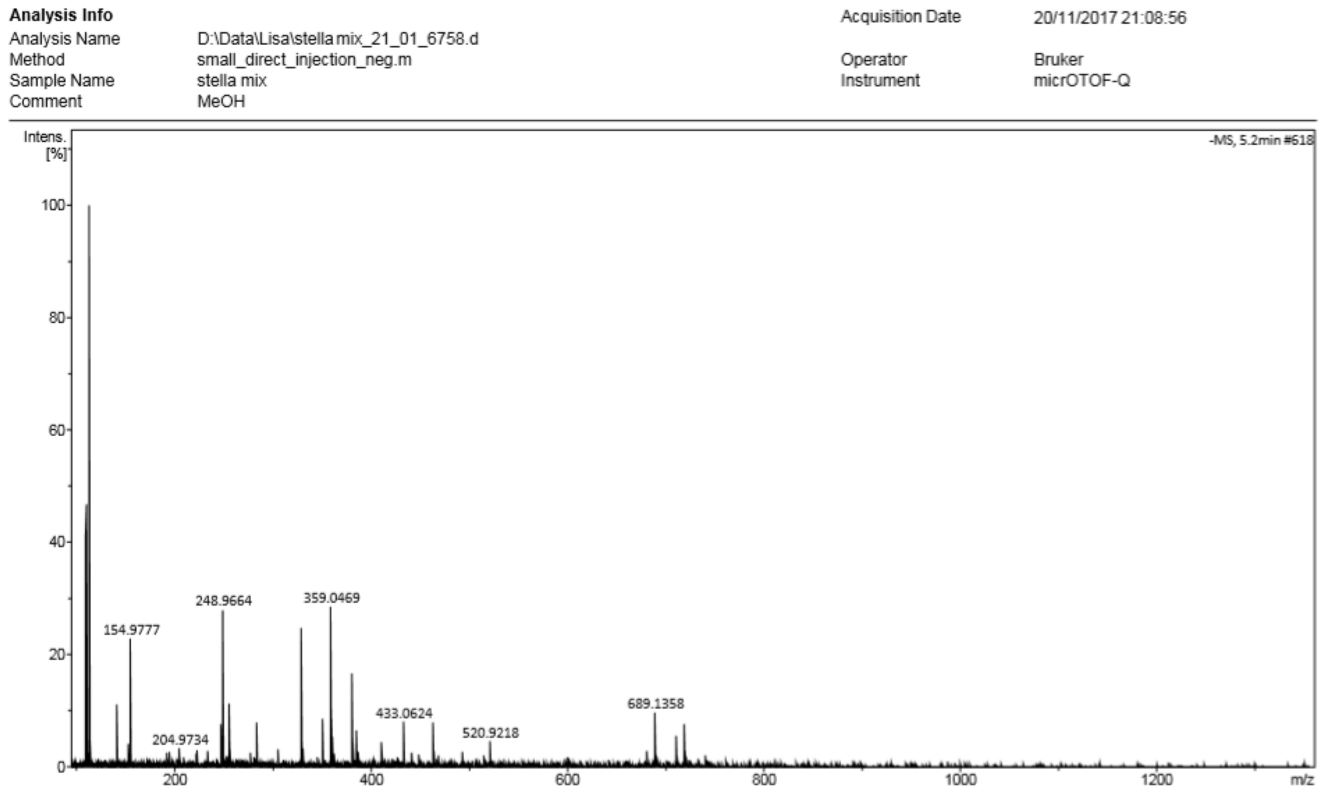

Figure S28. A high-resolution mass spectrum (ESI –ve) obtained for compounds **1** and **2** in methanol.

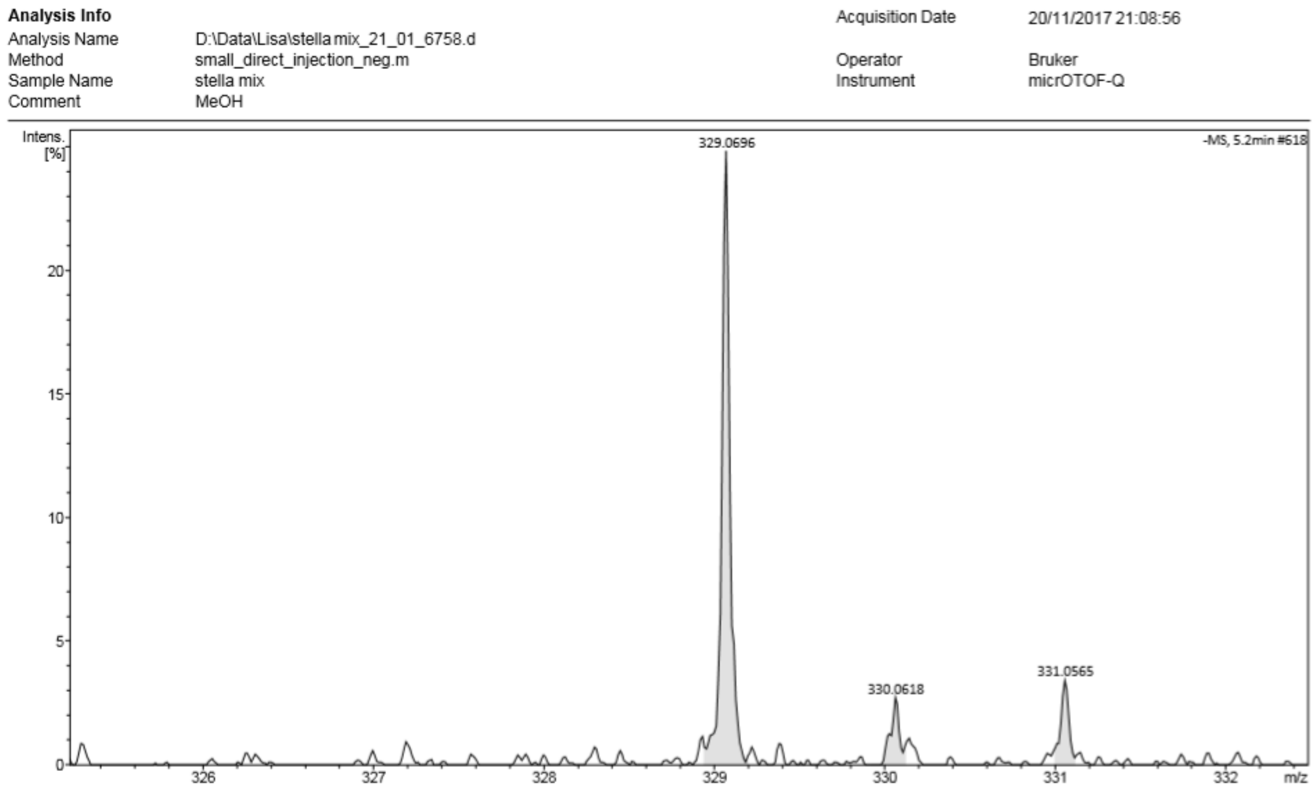

Figure S29. A high-resolution mass spectrum (ESI –ve) obtained for compounds **1** and **2** in methanol.

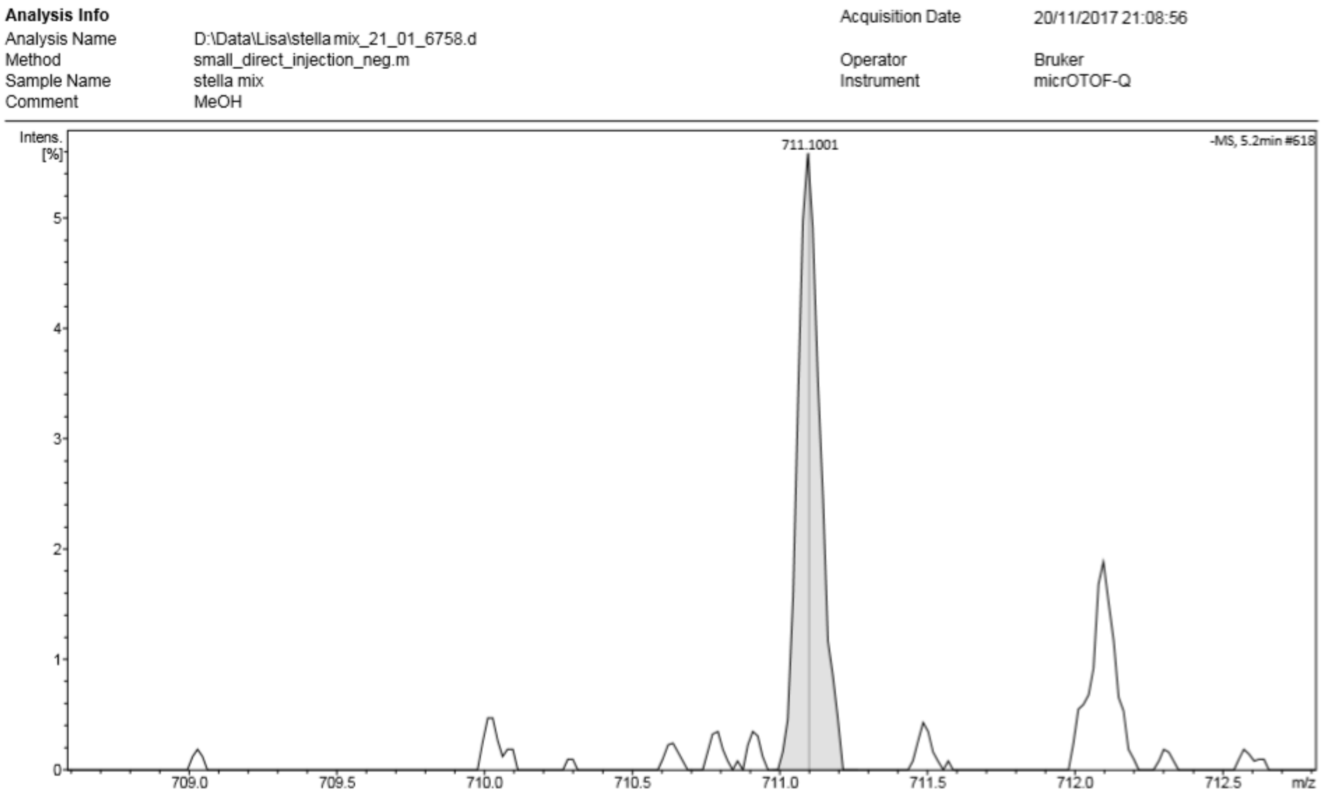

Figure S30. A high-resolution mass spectrum (ESI –ve) obtained for compounds **1** and **2** in methanol.

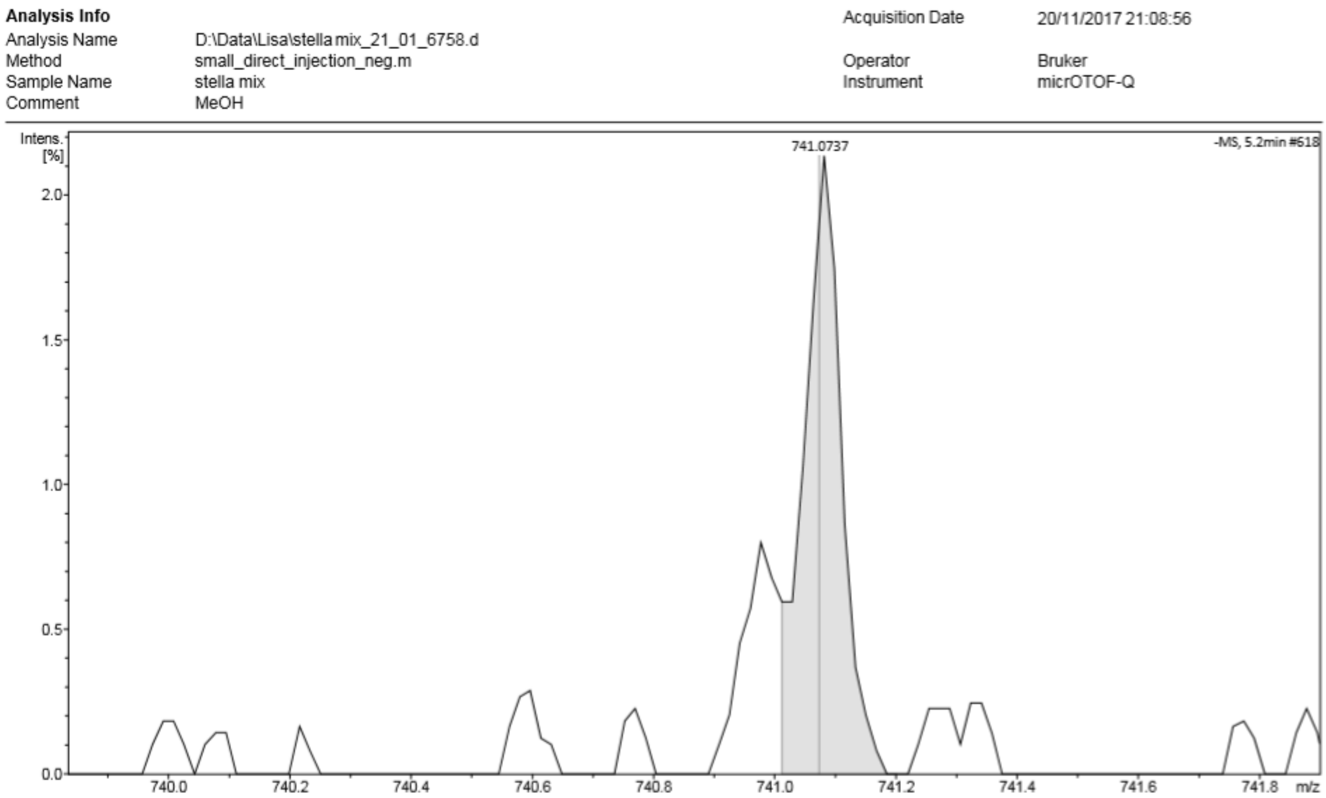

Figure S31. A high-resolution mass spectrum (ESI –ve) obtained for compounds **1** and **2** in methanol.

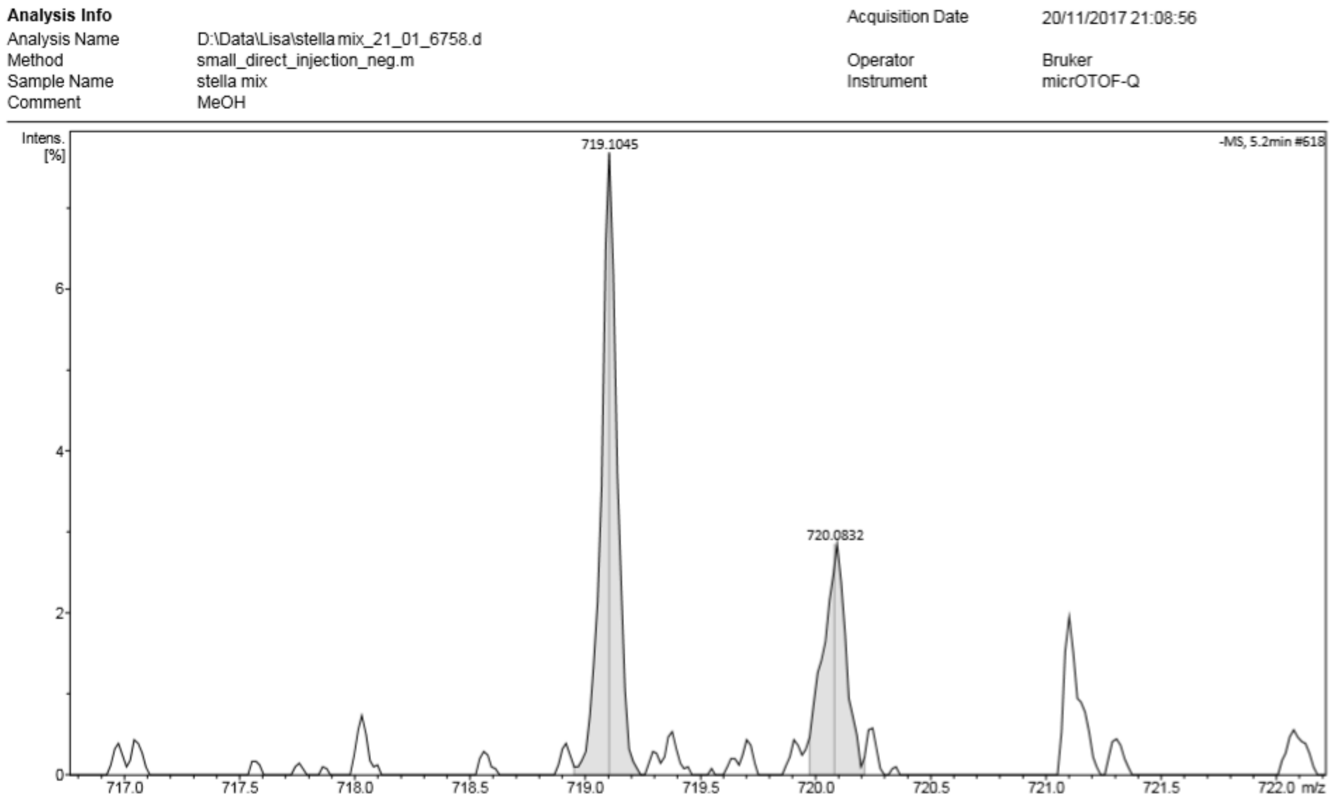

Figure S32. A high-resolution mass spectrum (ESI –ve) obtained for compounds **1** and **2** in methanol.

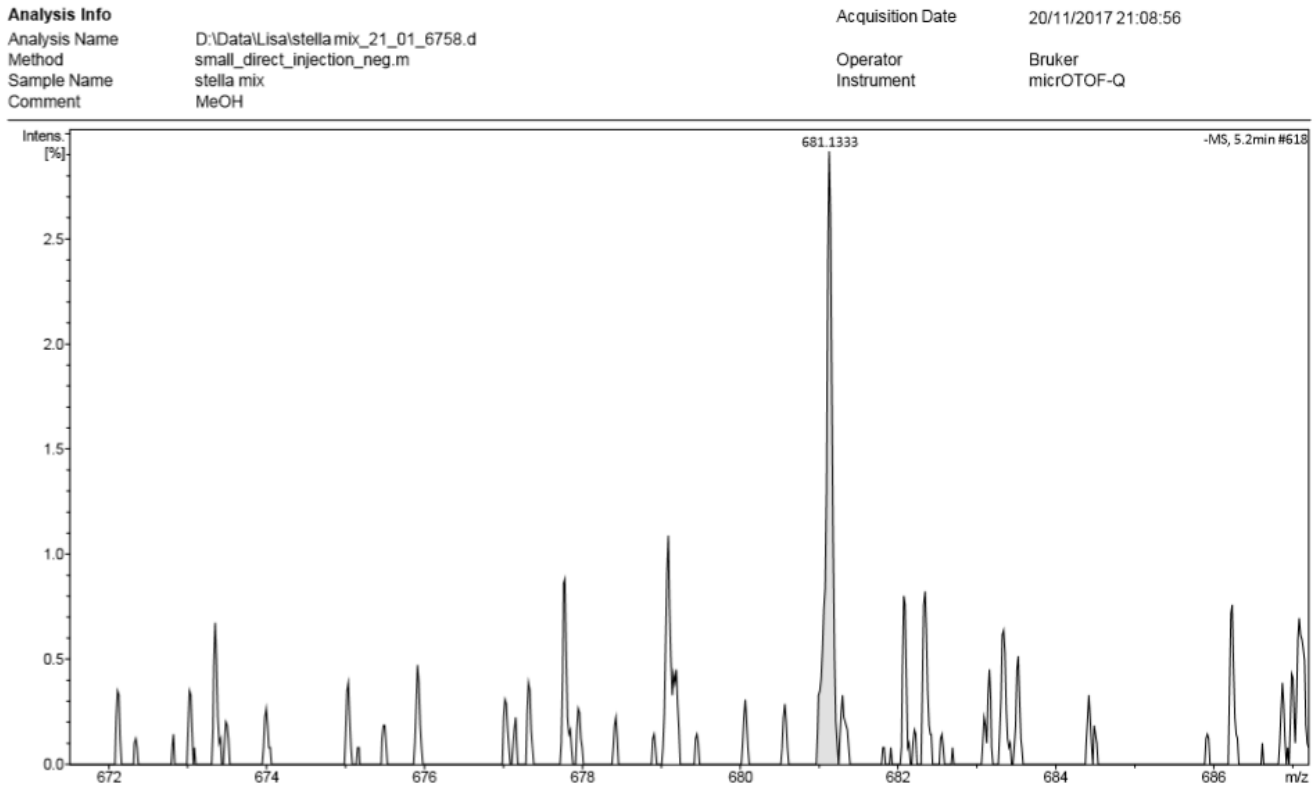

Figure S33. A high-resolution mass spectrum (ESI –ve) obtained for compounds **1** and **2** in methanol.

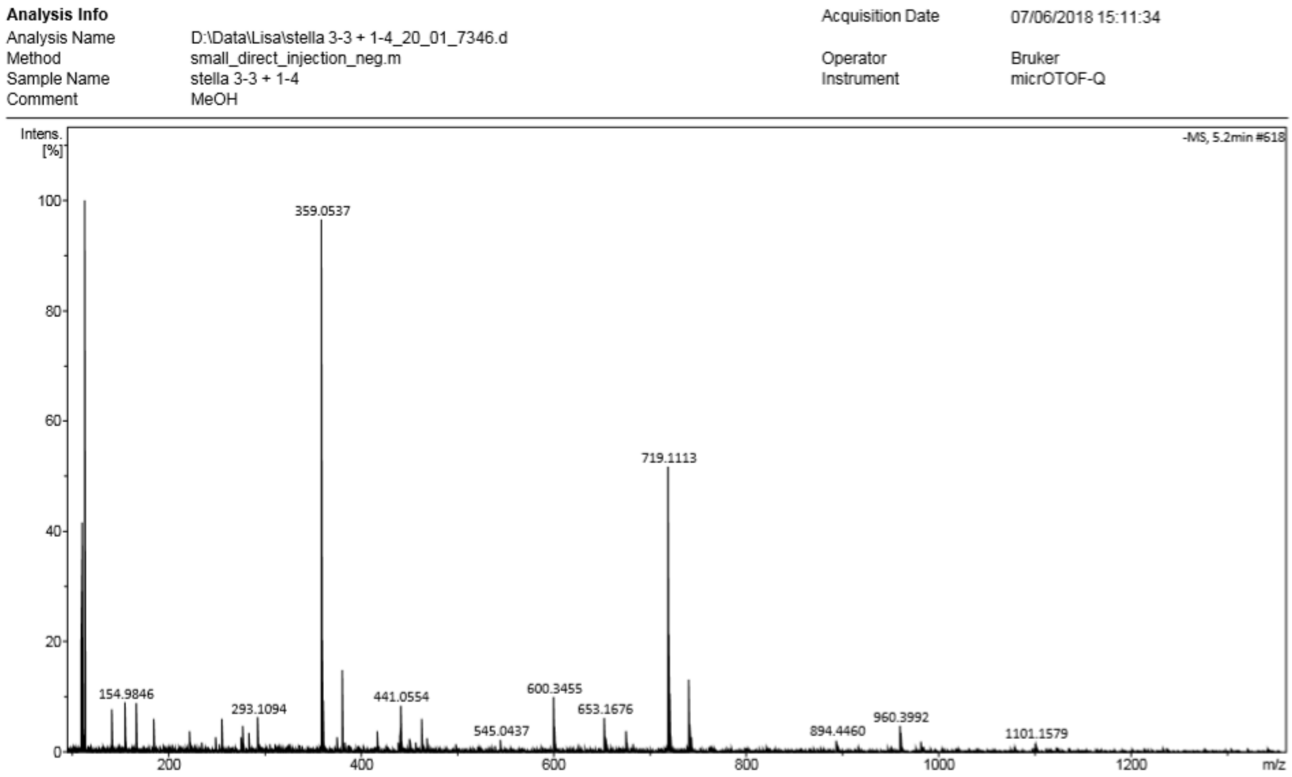

Figure S34. A high-resolution mass spectrum (ESI –ve) obtained from a mixture for compounds **1** and **4** in methanol.

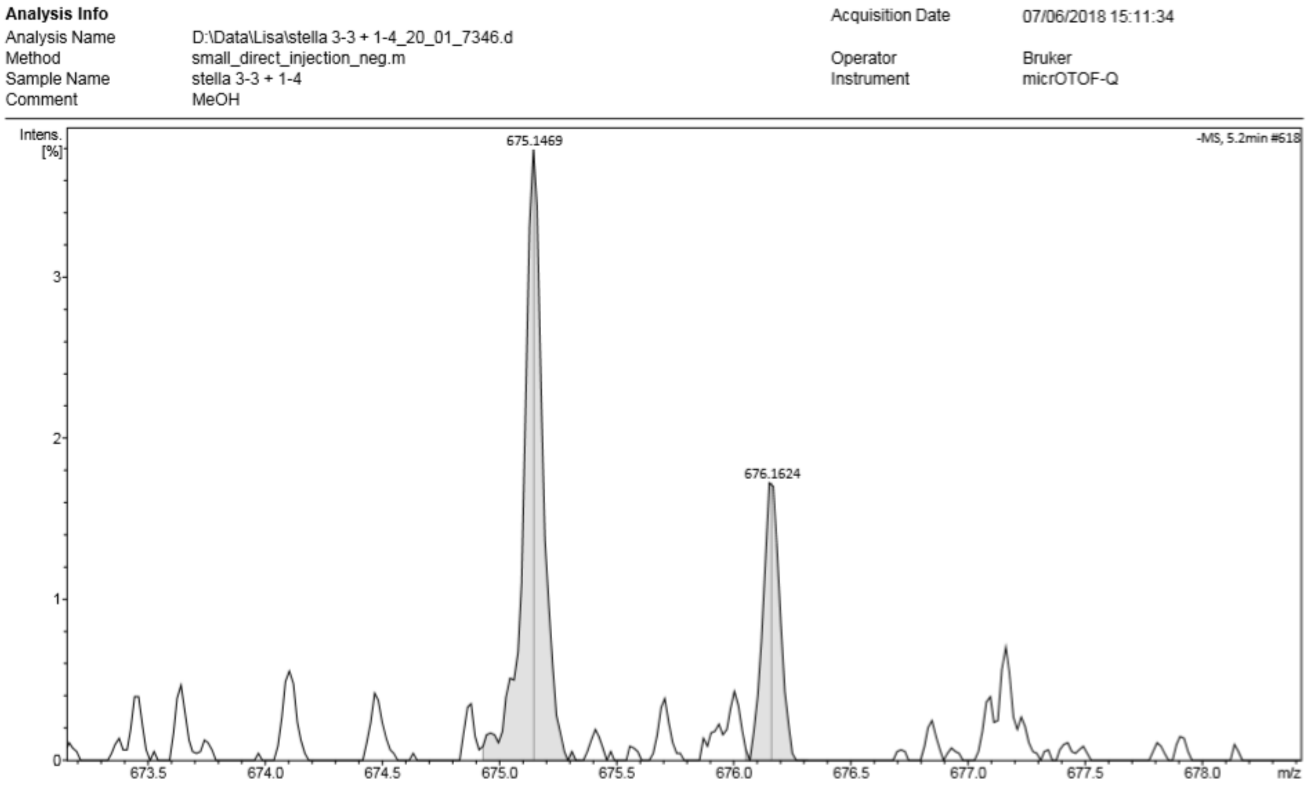

Figure S35. A high-resolution mass spectrum (ESI –ve) obtained from a mixture for compounds **1** and **4** in methanol.

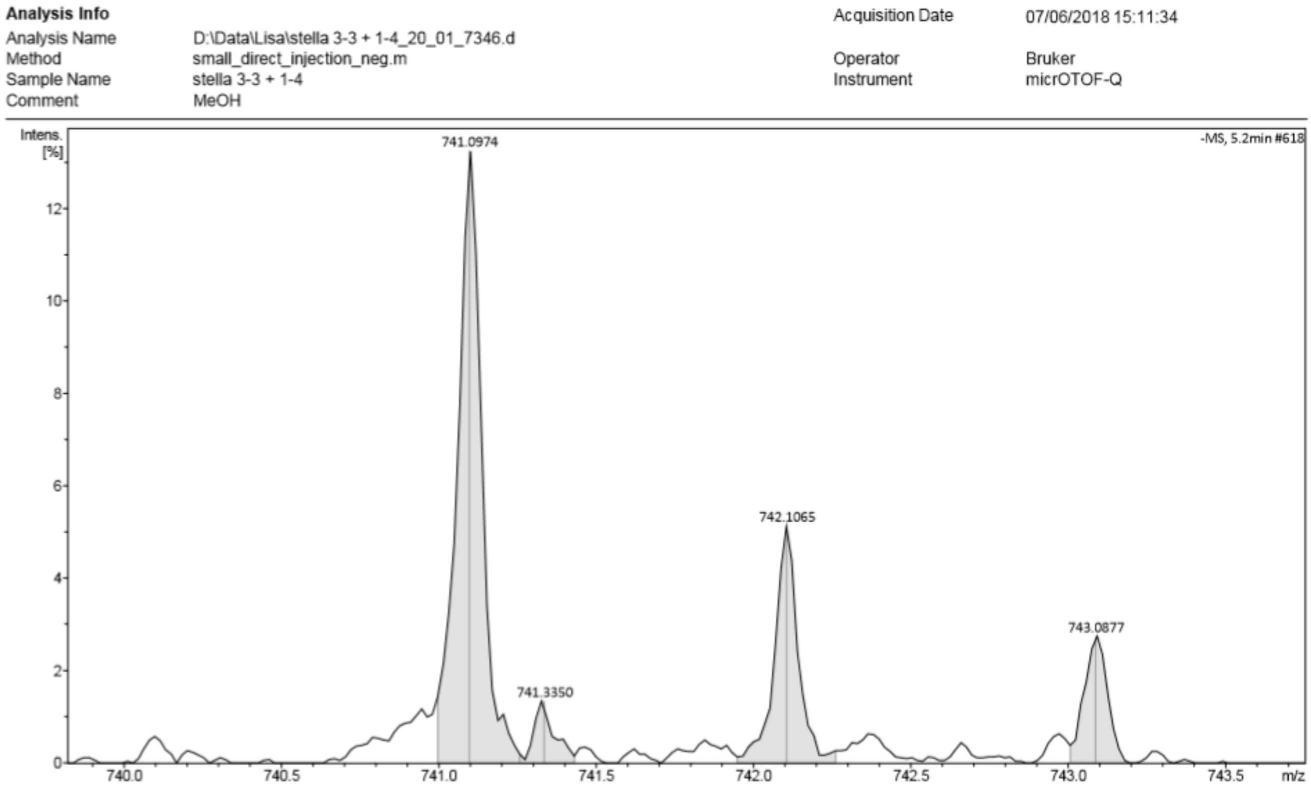

Figure S36. A high-resolution mass spectrum (ESI –ve) obtained from a mixture for compounds **1** and **4** in methanol.

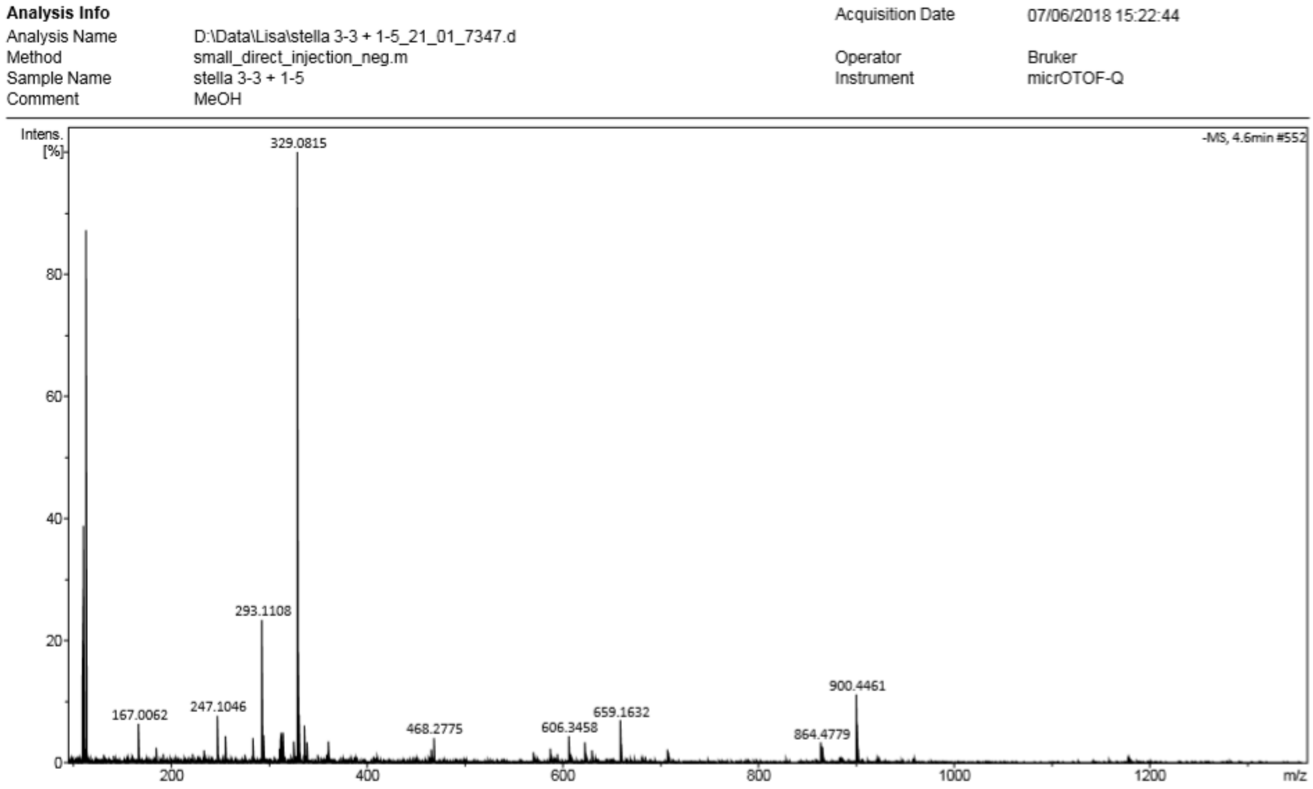

Figure S37. A high-resolution mass spectrum (ESI –ve) obtained from a mixture for compounds **2** and **4** in methanol.

Analysis Info

Analysis Name	D:\Data\Lisa\stella 3-3 + 1-5_21_01_7347.d	Acquisition Date	07/06/2018 15:22:44
Method	small_direct_injection_neg.m	Operator	Bruker
Sample Name	stella 3-3 + 1-5	Instrument	micrOTOF-Q
Comment	MeOH		

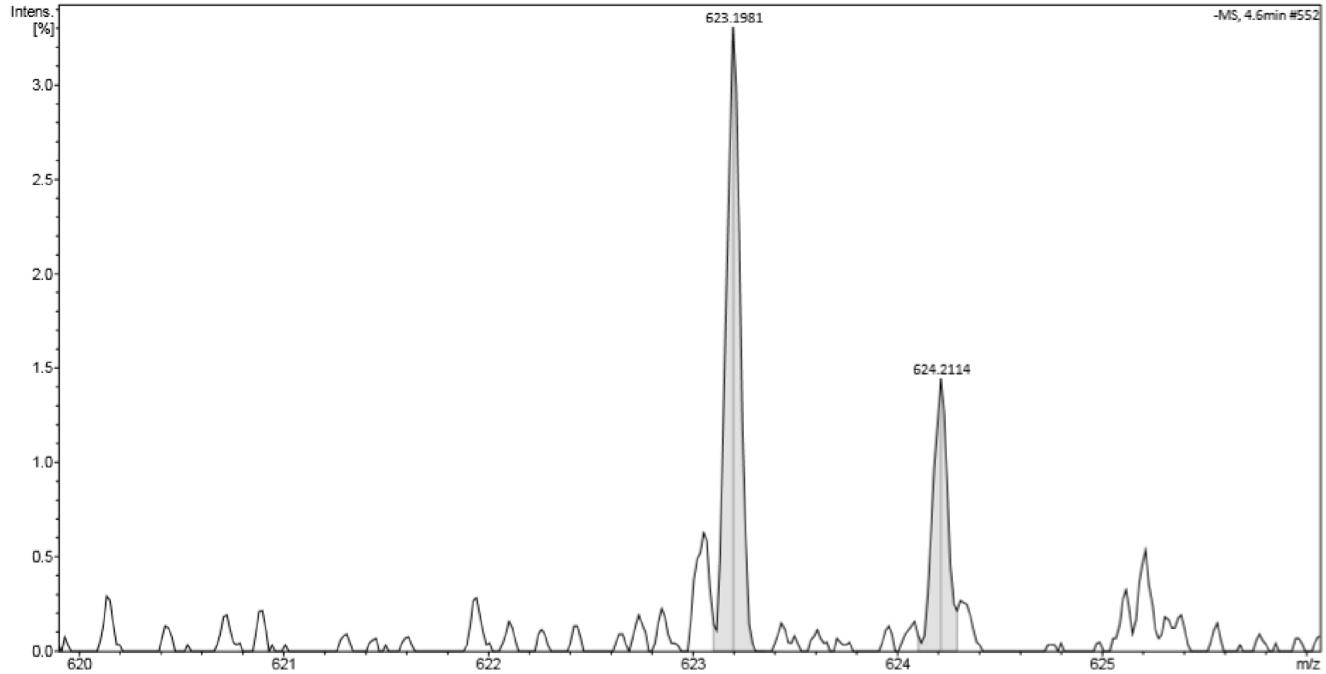

Figure S38. A high-resolution mass spectrum (ESI –ve) obtained from a mixture for compounds **2** and **4** in methanol.

Analysis Info

Analysis Name	D:\Data\Lisa\stella 3-3 + 1-5_21_01_7347.d	Acquisition Date	07/06/2018 15:22:44
Method	small_direct_injection_neg.m	Operator	Bruker
Sample Name	stella 3-3 + 1-5	Instrument	micrOTOF-Q
Comment	MeOH		

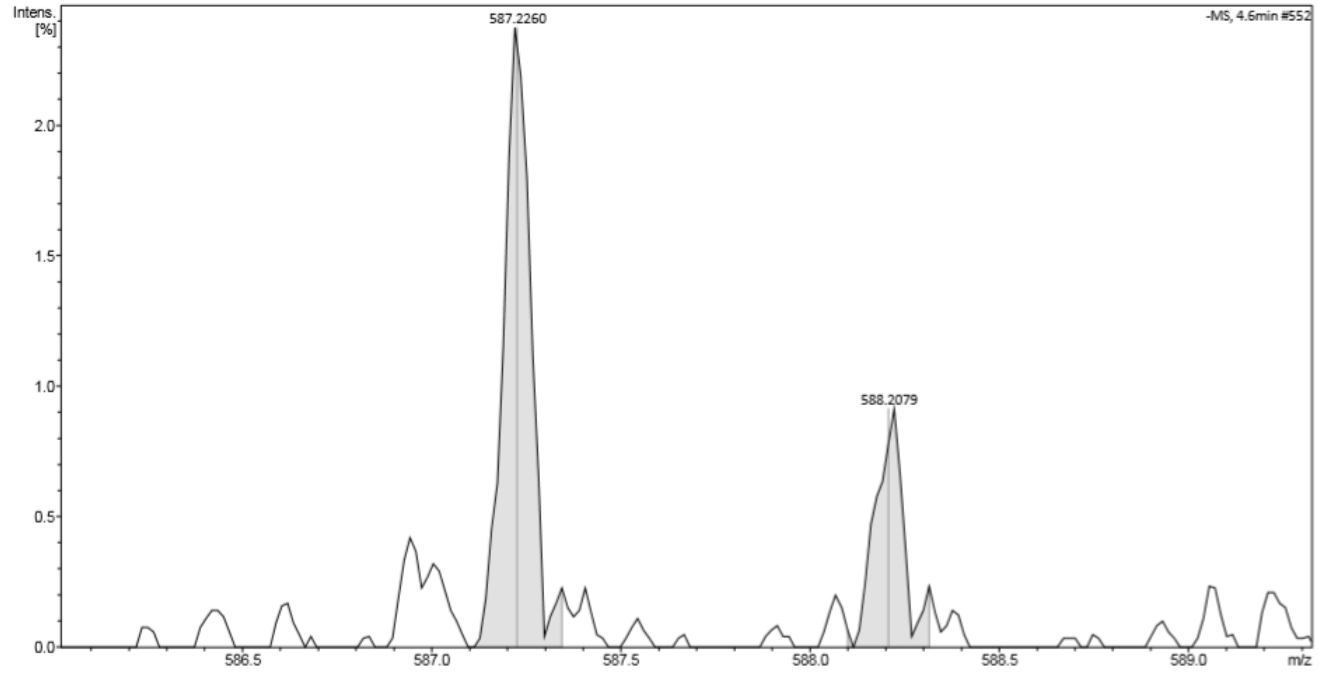

Figure S39. A high-resolution mass spectrum (ESI –ve) obtained from a mixture for compounds **2** and **4** in methanol.

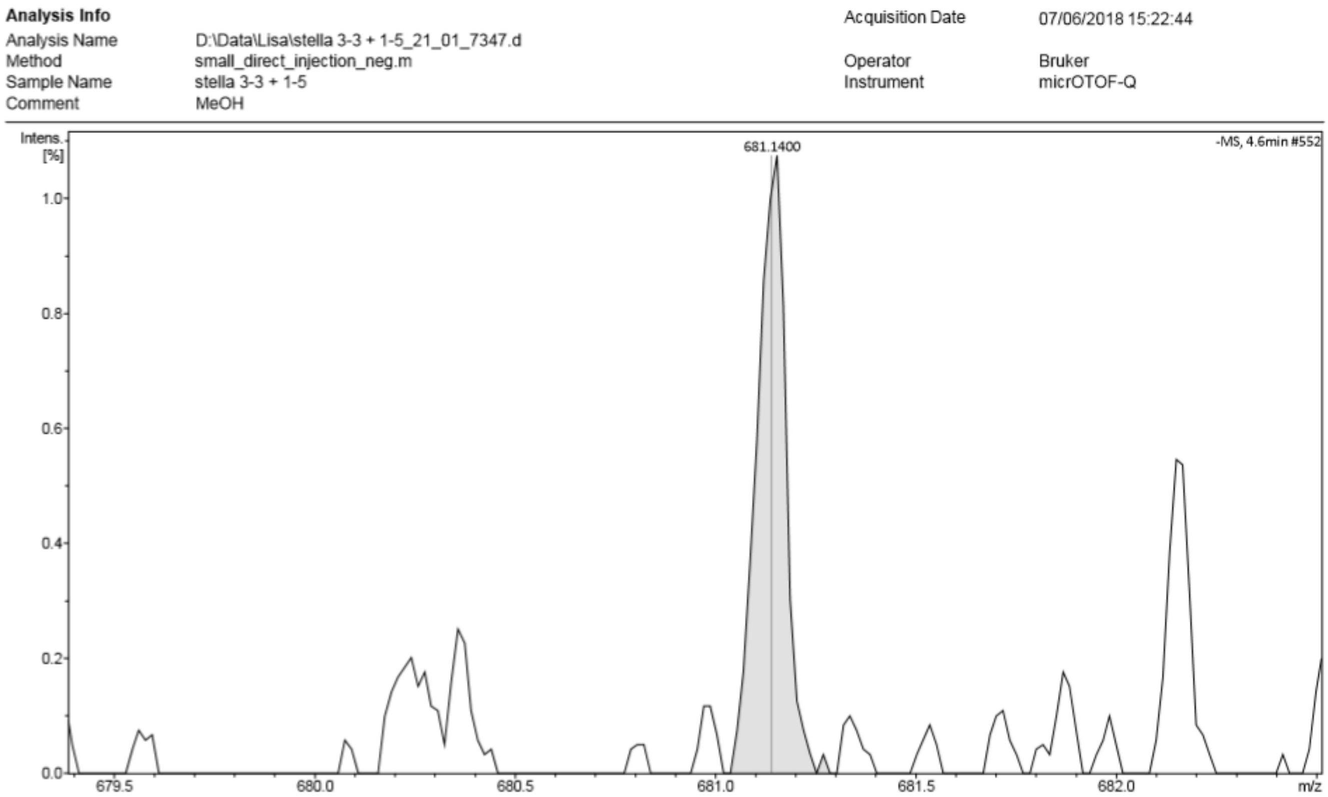

Figure S40. A high-resolution mass spectrum (ESI –ve) obtained from a mixture for compounds **2** and **4** in methanol.

Table S1. An overview of the single molecular species observed by high resolution ESI –ve mass spectrometry for **1**-**4**.

Compound	Theoretical *(m/z)*		Actual *(m/z)*	
	$[M]^-$	$[M-H]^-$	$[M]^-$	$[M-H]^-$
**1**	359.0343	N/A	359.0339	N/A
**2**	329.0602	N/A	329.0685	N/A
**3**	N/A	349.1557	N/A	349.1628
**4**	293.0932	N/A	293.0932	N/A

Table S2. An overview of self-associated species observed by high resolution ESI –ve mass spectrometry for **1-4**.

Compound	Theoretical *(m/z)*				Actual *(m/z)*			
	$[2M+H]^-$	$[2M-H]^-$	$[2M+Na]^-$	$[2M+K]^-$	$[2M+H]^-$	$[2M-H]^-$	$[2M+Na]^-$	$[2M+K]^-$
**1**	719.0759	N/A	741.0578	[a]	719.0765	N/A	741.0586	[a]
**2**	[a]	N/A	681.1096	[a]	[a]	N/A	681.1249	[a]
**3**	N/A	[a]	N/A	N/A	N/A	[a]	N/A	N/A
**4**	587.1937	N/A	609.1756	[a]	587.1877	N/A	609.1683	[a]

[a] Ion not observed.

Table S3. An overview of species observed by high resolution ESI -ve mass spectrometry for mixtures containing **1**, **2** and **4** in a 1:1 ratio. Ma and Mb represent the anionic component of that amphiphilic salts contained within the mixtures analysed.

Molecular complex	**1** (Ma) + **2** (Mb)		**1** (Ma) + **4** (Mb)		**2** (Ma) + **4** (Mb)	
	Theoretical *(m/z)*	Actual *(m/z)*	Theoretical *(m/z)*	Actual *(m/z)*	Theoretical *(m/z)*	Actual *(m/z)*
$[Ma]^-$	359.0343	359.0469	359.0343	359.0537	329.0602	329.0815
$[Mb]^-$	329.0602	329.0696	293.0932	293.1094	293.0932	293.1108
$[Ma+Mb+H]^-$	689.1018	689.1358	653.1348	653.1676	623.1607	623.1981
$[Ma+Mb+Na]^-$	711.0837	711.1001	675.1167	675.1469	[a]	[a]
$[Ma+Mb+K]^-$	[a]	[a]	[a]	[a]	[a]	[a]
$[Ma+Ma+H]^-$	719.0759	719.1045	719.0759	719.1113	659.1277	659.1632
$[Ma+Ma+Na]^-$	741.0578	741.0737	741.0578	741.0974	681.1096	681.1400
$[Ma+Ma+K]^-$	[a]	[a]	[a]	[a]	[a]	[a]
$[Mb+Mb+H]^-$	[a]	[a]	[a]	[a]	587.1937	587.2260
$[Mb+Mb+Na]^-$	681.1096	681.1333	[a]	[a]	[a]	[a]
$[Mb+Mb+K]^-$	[a]	[a]	[a]	[a]	[a]	[a]

[a] Ion not observed

^{1}H NMR DOSY Data

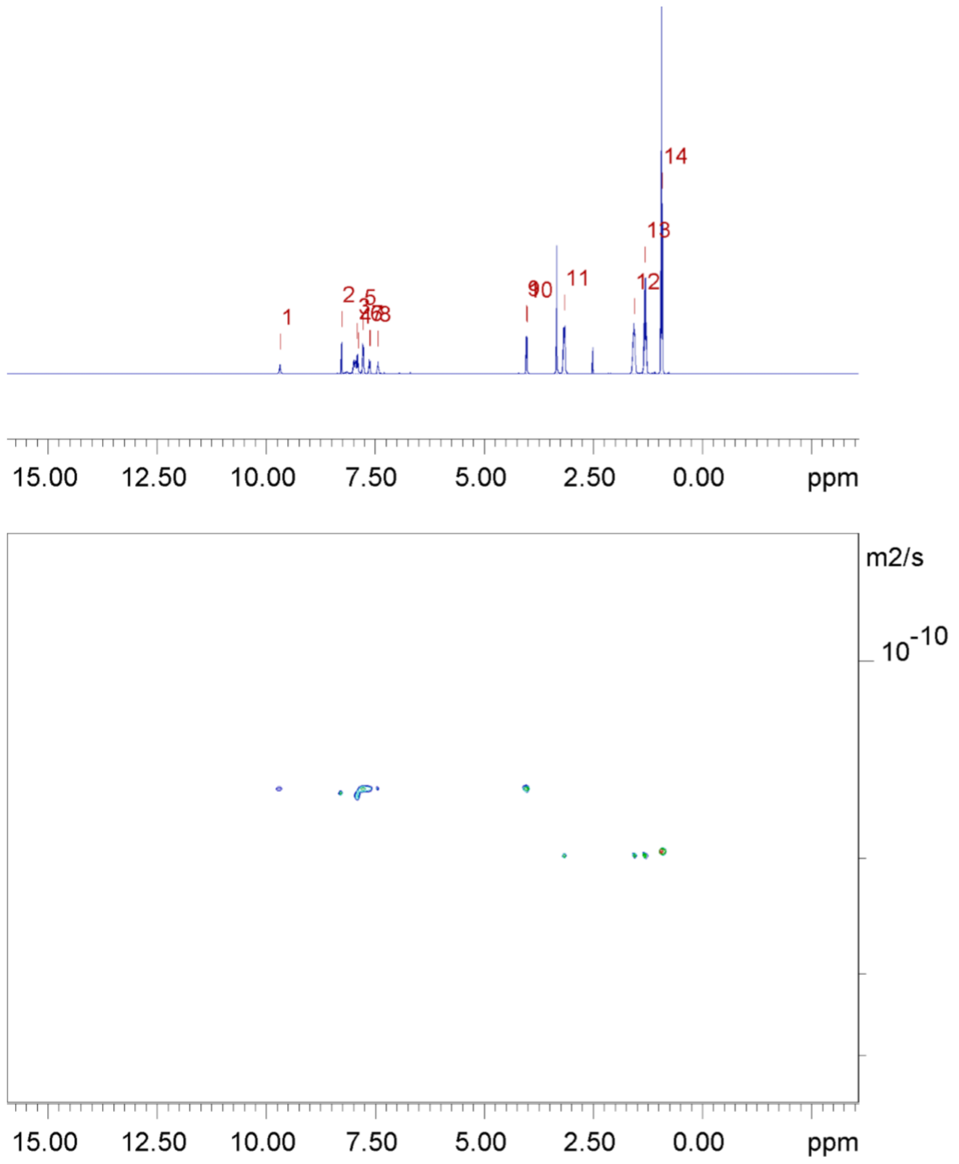

Peak name	F2 [ppm]	Io	error	D [m2/s]	error
1	9.677	6.03e+06	9183	1.58e-10	5.268e-13
2	8.262	2.12e+07	9186	1.58e-10	1.501e-13
3	7.913	1.25e+07	9241	1.61e-10	2.619e-13
4	7.891	1.32e+07	9185	1.58e-10	2.404e-13
5	7.774	1.90e+07	9175	1.57e-10	1.660e-13
6	7.633	9.51e+06	9172	1.57e-10	3.320e-13
7	7.611	8.33e+06	9174	1.57e-10	3.796e-13
8	7.432	7.86e+06	9172	1.57e-10	4.019e-13
9	4.032	2.47e+07	9149	1.56e-10	1.266e-13
10	4.017	2.49e+07	9153	1.56e-10	1.256e-13
11	3.160	3.11e+07	9829	1.99e-10	1.362e-13
12	1.563	3.37e+07	9822	1.99e-10	1.254e-13
13	1.313	6.38e+07	9793	1.97e-10	6.547e-14
14	0.927	2.74e+08	9775	1.96e-10	1.515e-14

Figure S41. ^{1}H DOSY NMR spectrum of compound **1** (55.56 mM) in DMSO-d_6 at 298 K and a Table reporting the diffusion constants calculated for each peak used to determine the hydrodynamic diameter of the anionic component of **1** (d_H = 1.39 nm). Peaks 1-10 correspond to the anionic component of **1** while peaks 11-14 correspond to the cationic component of **1**.

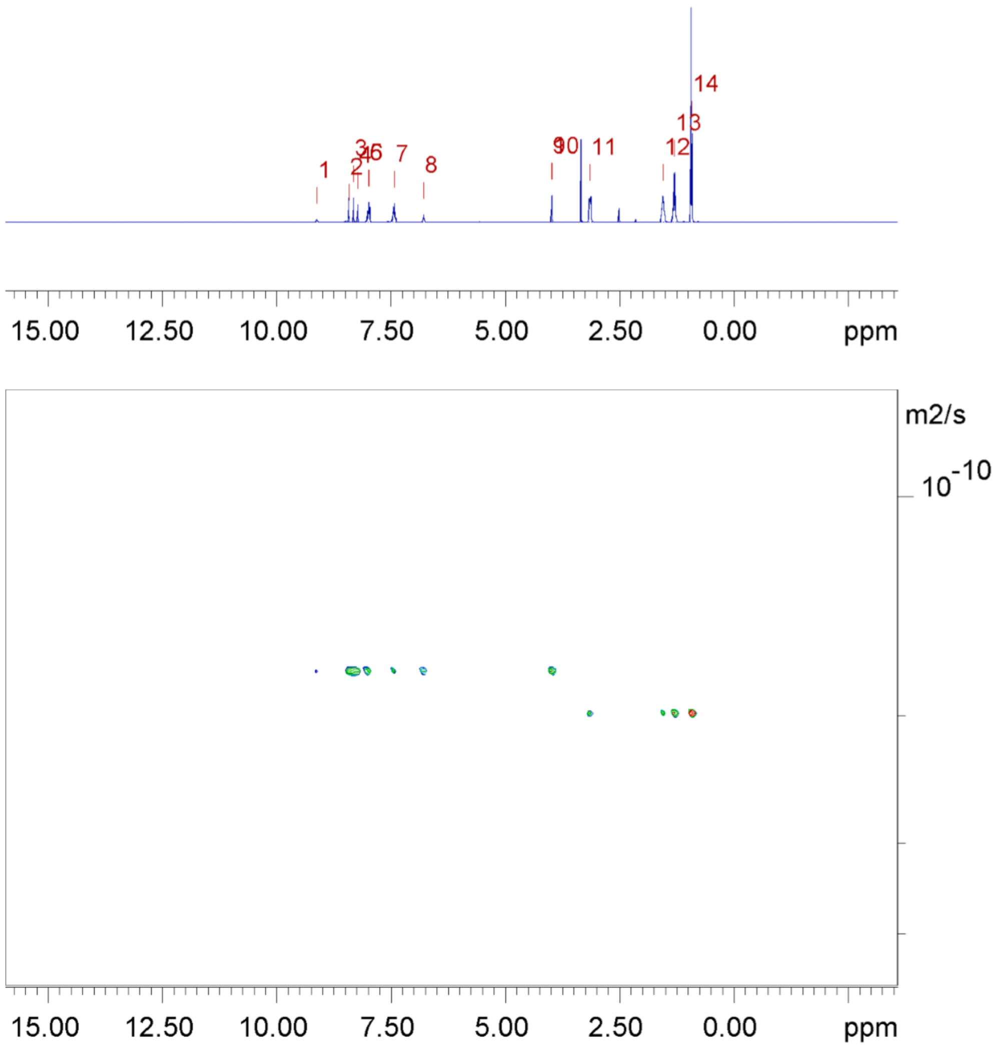

Peak name	F2 [ppm]	Io	error	D [m2/s]	error
1	9.121	3.43e+06	9331	1.73e-10	1.024e-12
2	8.422	3.23e+07	9343	1.74e-10	1.094e-13
3	8.316	3.20e+07	9341	1.74e-10	1.104e-13
4	8.221	2.33e+07	9348	1.74e-10	1.520e-13
5	7.990	2.66e+07	9332	1.73e-10	1.322e-13
6	7.976	2.63e+07	9334	1.73e-10	1.338e-13
7	7.421	2.43e+07	9335	1.74e-10	1.447e-13
8	6.781	1.03e+07	9339	1.74e-10	3.420e-13
9	3.987	3.48e+07	9334	1.73e-10	1.012e-13
10	3.972	3.50e+07	9349	1.74e-10	1.014e-13
11	3.142	3.20e+07	9698	1.99e-10	1.295e-13
12	1.547	3.43e+07	9699	1.99e-10	1.208e-13
13	1.305	6.44e+07	9700	1.99e-10	6.442e-14
14	0.925	2.80e+08	9703	1.99e-10	1.485e-14

Figure S42. ^{1}H DOSY NMR spectrum of compound **2** (55.56 mM) in DMSO-d_6 conducted at 298 K and a Table reporting the calculated diffusion constant for each peak used to determine the hydrodynamic diameter for the anionic component of **2** (d_H = 1.26 nm). Peaks 1-10 correspond to the anionic component of **2** while peaks 11-14 correspond to the cationic component of **2**.

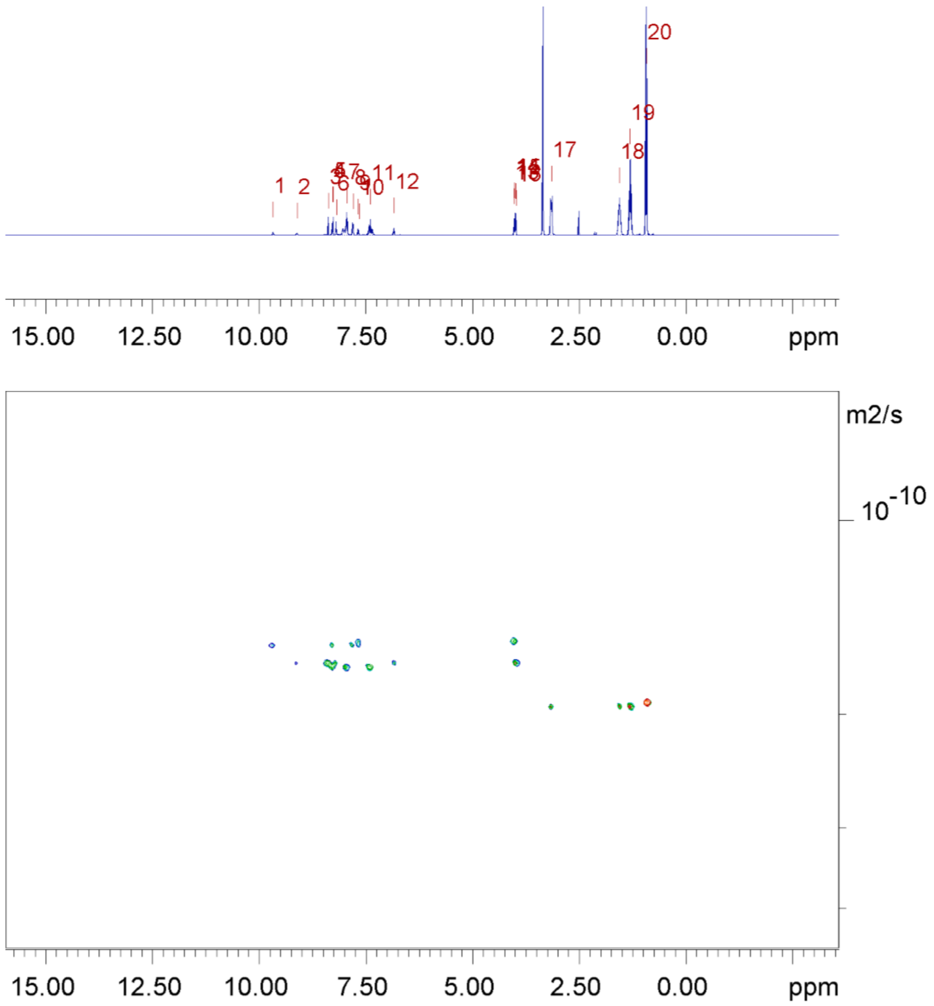

Peak name	F2 [ppm]	Io	error	D [m2/s]	error
1	9.672	3.27e+06	9041	1.56e-10	9.666e-13
2	9.111	2.28e+06	9210	1.67e-10	1.499e-12
3	8.378	2.04e+07	9210	1.67e-10	1.677e-13
4	8.280	1.41e+07	9045	1.56e-10	2.245e-13
5	8.264	2.02e+07	9222	1.68e-10	1.703e-13
6	8.189	1.51e+07	9214	1.68e-10	2.276e-13
7	7.945	2.55e+07	9233	1.69e-10	1.355e-13
8	7.794	1.17e+07	9040	1.56e-10	2.703e-13
9	7.683	6.39e+06	9024	1.55e-10	4.898e-13
10	7.661	5.55e+06	9042	1.56e-10	5.691e-13
11	7.394	1.76e+07	9229	1.69e-10	1.958e-13
12	6.840	7.83e+06	9211	1.67e-10	4.369e-13
13	4.025	1.84e+07	9004	1.54e-10	1.687e-13
14	4.010	1.89e+07	9024	1.55e-10	1.653e-13
15	3.992	2.53e+07	9185	1.66e-10	1.335e-13
16	3.978	2.53e+07	9201	1.67e-10	1.348e-13
17	3.150	3.88e+07	9609	1.95e-10	1.058e-13
18	1.555	4.13e+07	9605	1.95e-10	9.903e-14
19	1.309	8.35e+07	9590	1.94e-10	4.868e-14
20	0.925	4.51e+08	9583	1.93e-10	8.979e-15

Figure S43. ^{1}H DOSY NMR spectrum of compounds **1** (27.78 mM) and **2** (27.78 mM) in DMSO-d_6 at 298 K and a Table reporting the diffusion constants calculated for each peak used to determine the hydrodynamic diameter of the anionic components of **1** (d_H = 1.41 nm) and **1** (d_H = 1.31 nm). Peaks 1-16 correspond to the anionic component of **1** and **2** while peaks 17-20 correspond to the cationic component of **1** and **2**.

^{1}H NMR Titration Study Data

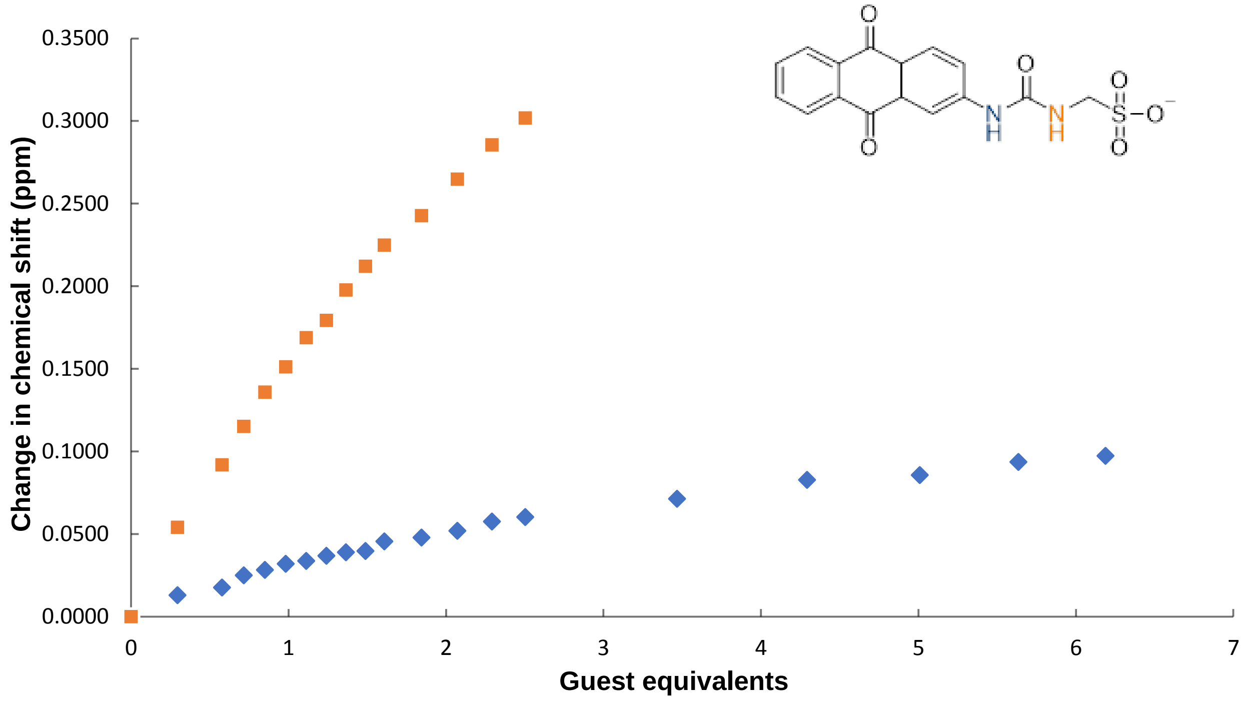

Figure S44. A graph showing the downfield ^{1}H NMR change in chemical shift for the NHs of compound **1** (host) with increasing the concentration of compound **2** (guest) in a DMSO-d_6 /0.5 % H_2O solution (298 K).

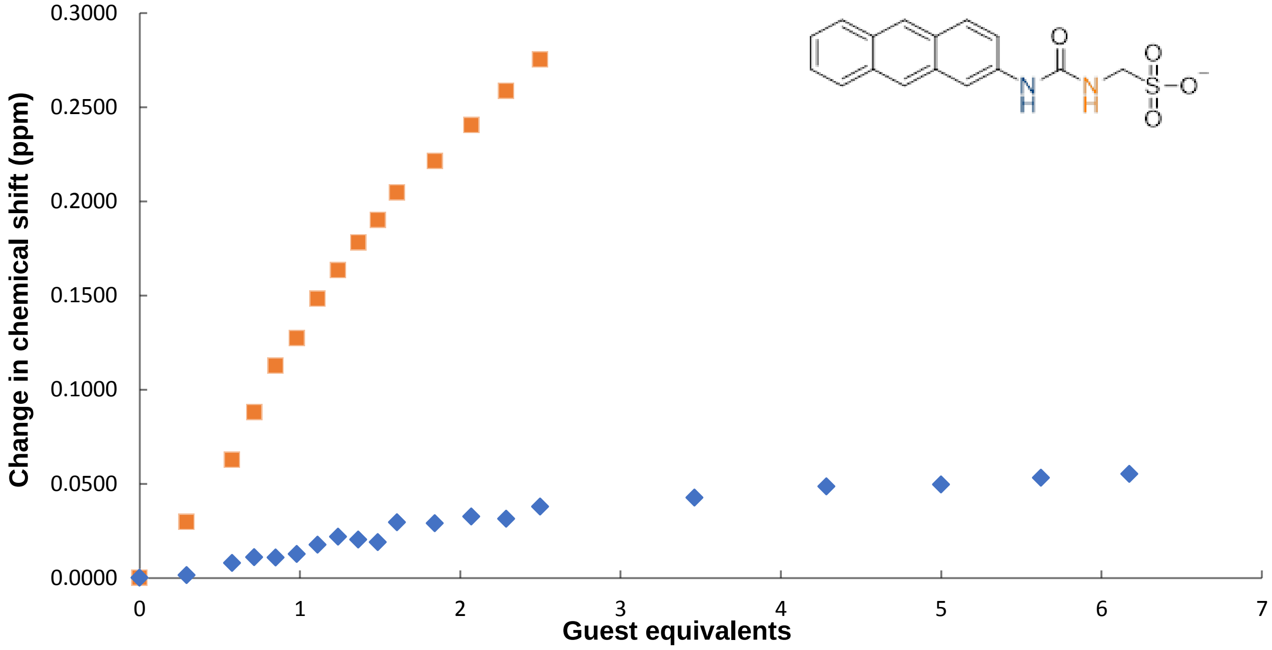

Figure S45. A graph showing the downfield ^{1}H NMR change in chemical shift for the NHs of compound **2** (host) with increasing the concentration of compound **1** (guest) in a DMSO-d_6 /0.5 % H_2O solution (298 K).

^{1}H NMR Dilution Study Data

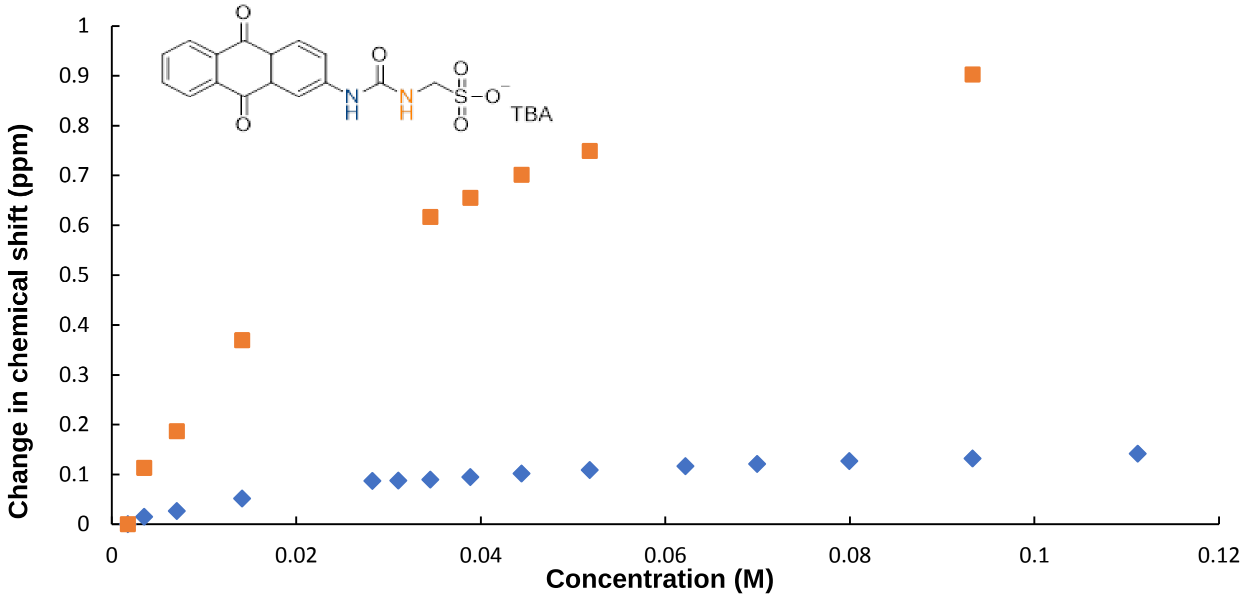

Figure S46. Graph showing the ^{1}H NMR downfield change in chemical shift of the urea NH resonances for compound **1** with increasing concentration in a DMSO- d_6/H_2O solution (298 K). Peak overlapping during the experiment prevented the fitting of the data, shown in orange, to a binding isotherm.

Self-association constant calculation using the change in chemical shift of a single urea NH

Equal K/Dimerization model

K_e = 33.68 M^{-1} ± 3.2234 % K_{dim} = 16.84 M^{-1} 1.6117 %

http://app.supramolecular.org/bindfit/view/744e6c77-6407-49f5-9bc7-b4046934f1b6

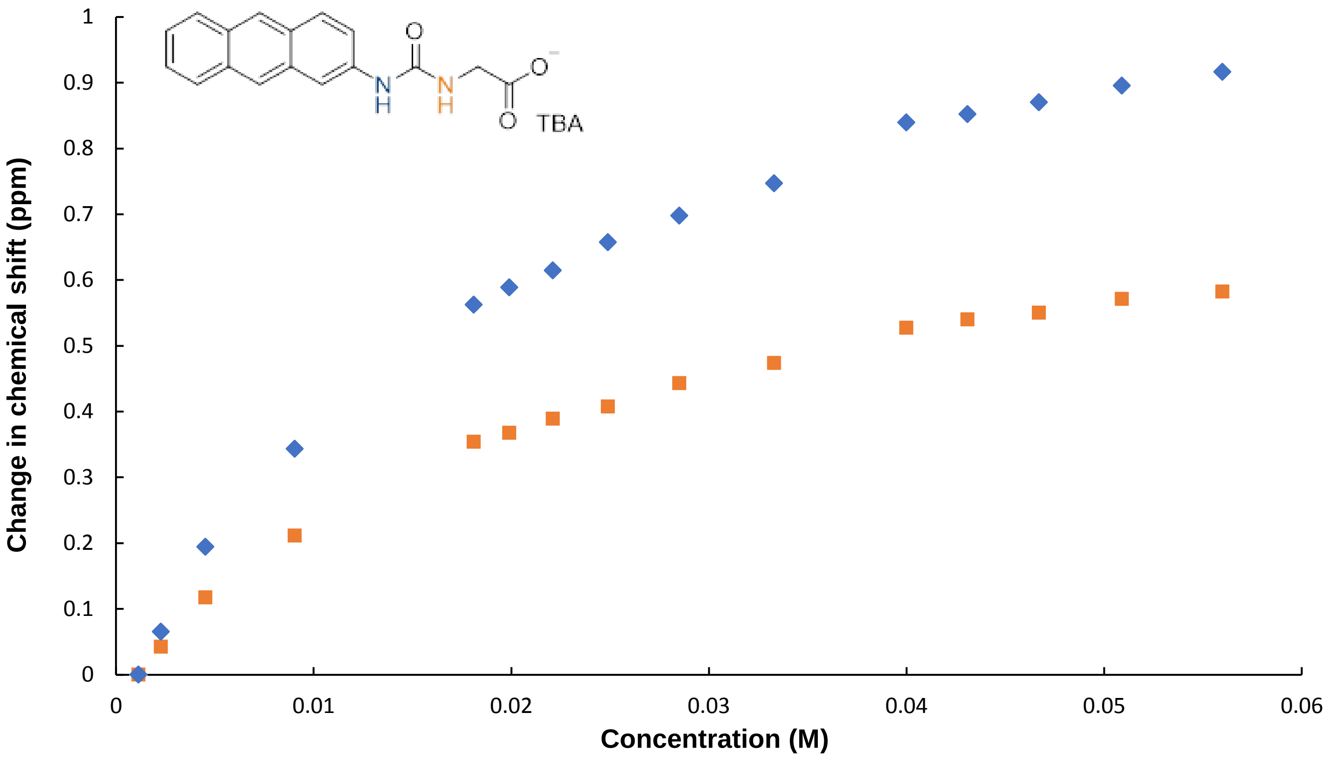

Figure S47. Graph showing the ^{1}H NMR down-field change in chemical shift of the urea NH resonances for compound **4** with increasing concentration in a DMSO- d_6/ 0.5% H_2O solution (298 K). As it is probable that larger aggregate structures of **4** exist within the DMSO solution that may not be visible using NMR, self-association constants were not determined using these data.

Quantitative ^{1}H NMR Data

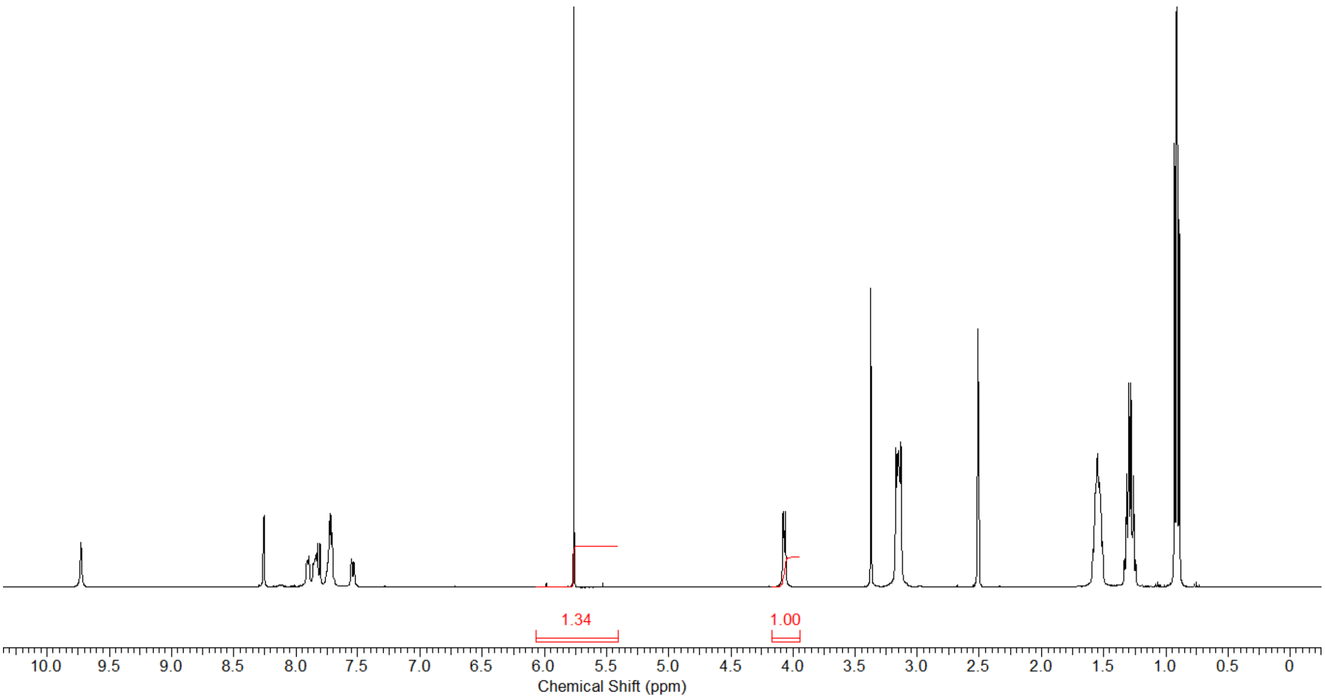

Figure S48. A ^{1}H NMR spectrum of compound **1** (33.50 mg, 0.055 mM) in a solution of DMSO-d_6 (0.495 mL) and dichloromethane (5 µL, 0.078 mM) with an extended delay (d_1 = 60 s). No apparent loss of compound observed.

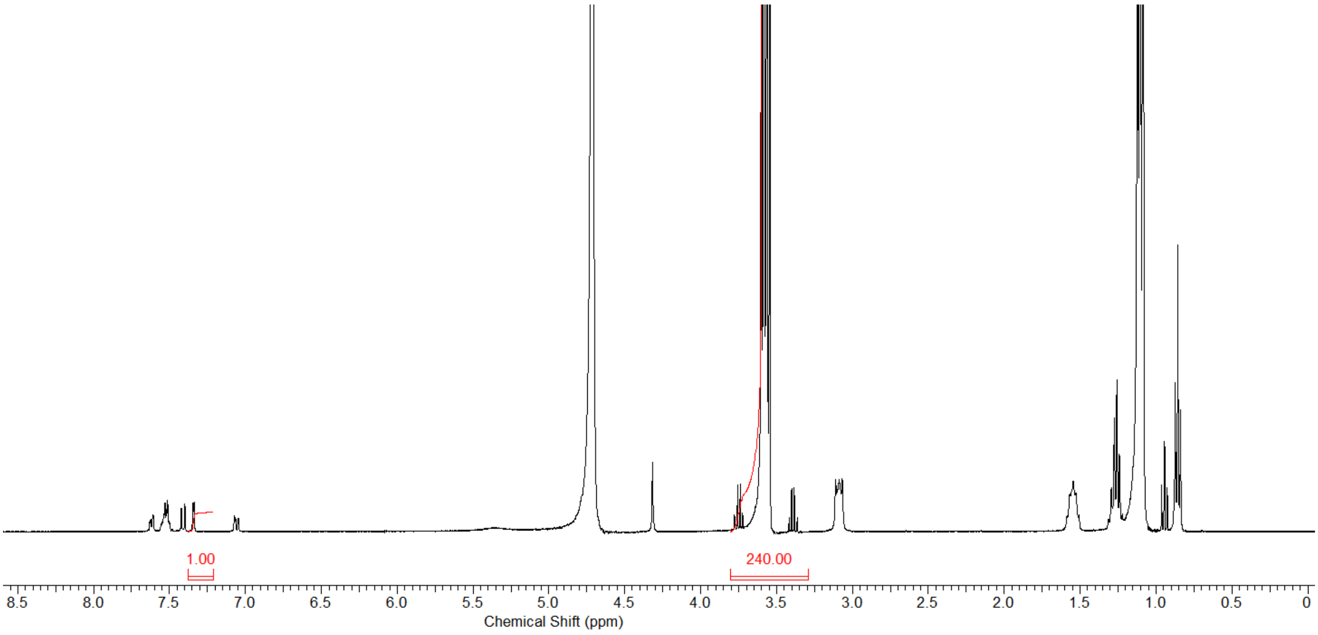

Figure S49. A ^{1}H NMR spectrum of compound **1** (1.65mg, 0.0027 mM) in a solution of ethanol (25 µL, 0.43 mM) and D_2O (0.475 mL) with an extended delay (d_1 = 60 s). A 34 % reduction of compound observed upon comparative integration.

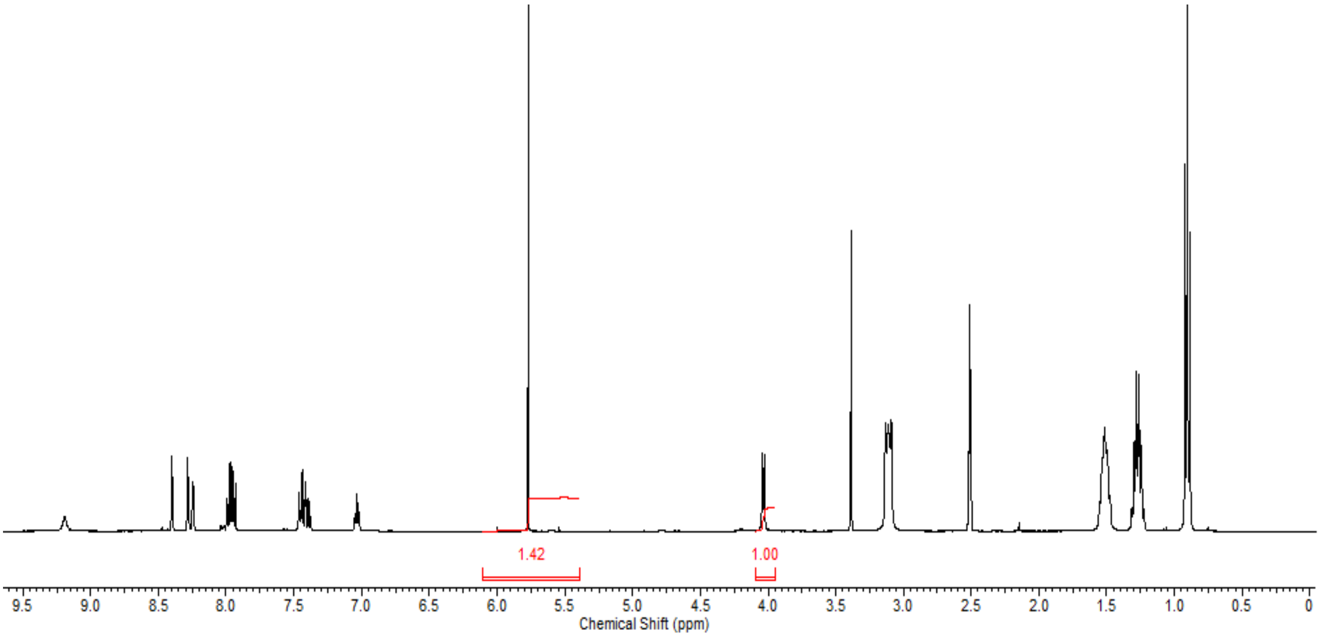

Figure S50. A ^{1}H NMR spectrum of compound **2** (31.73 mg, 0.055 mM) in a solution of DMSO-d_6 (0.495 mL) and dichloromethane (5 µL, 0.078 mM) with an extended delay (d_1 = 60 s). No apparent loss of compound observed.

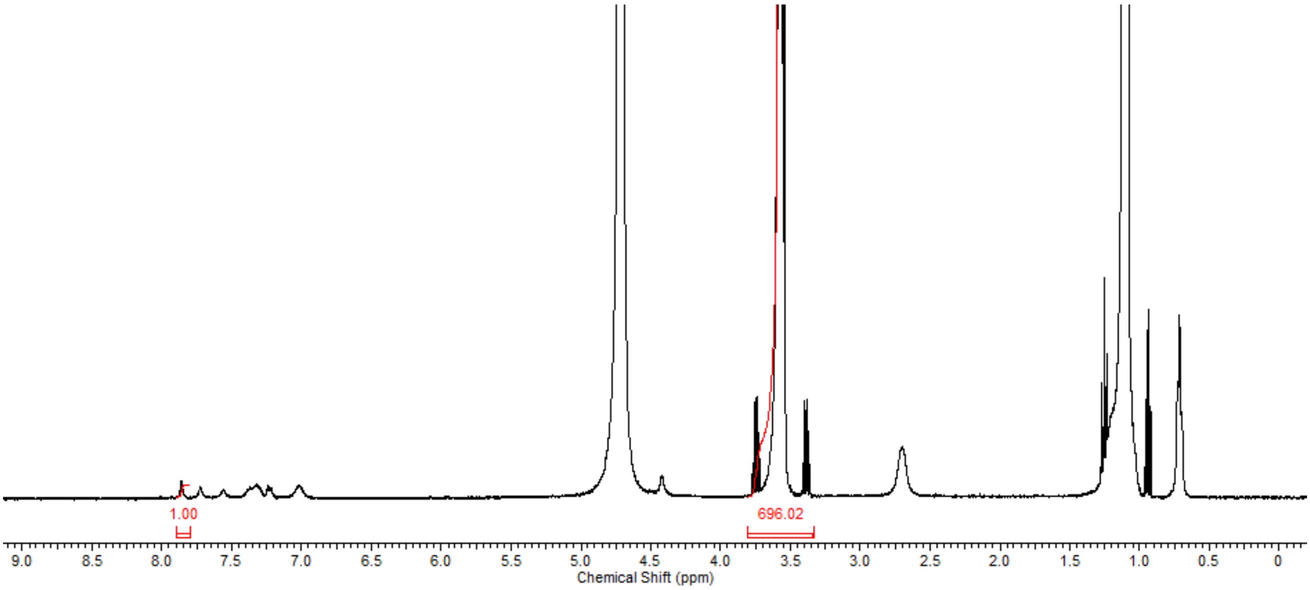

Figure S51. A ^{1}H NMR spectrum of compound **2** (1.57 mg, 0.0027 mM) in a solution of ethanol (25 µL, 0.43 mM) and D_2O (0.475 mL) with an extended delay (d_1 = 60 s). A 77 % reduction of compound observed upon comparative integration.

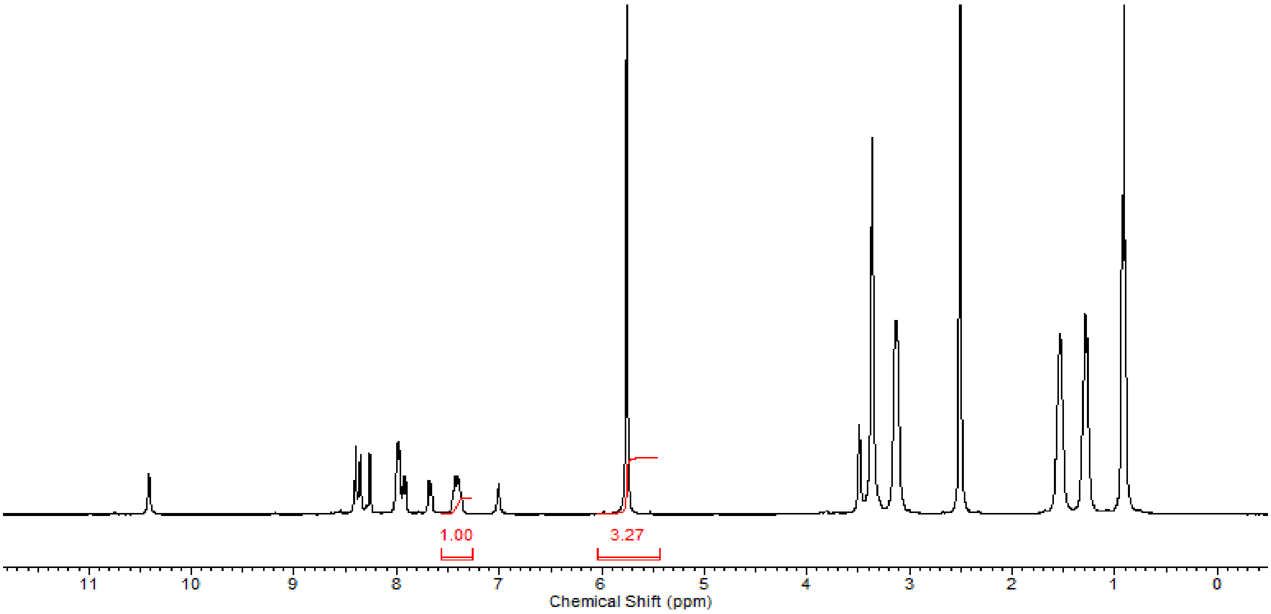

Figure S52. A ^{1}H NMR spectrum of compound **4** (14.98 mg, 0.028 mM) in a solution of DMSO-d_6 (0.495 mL) and dichloromethane (5 µL, 0.078 mM) with an extended delay (d_1 = 60 s). The experiment was carried out at a lower concentration due to solubility. A 15% reduction of compound observed upon comparative integration.

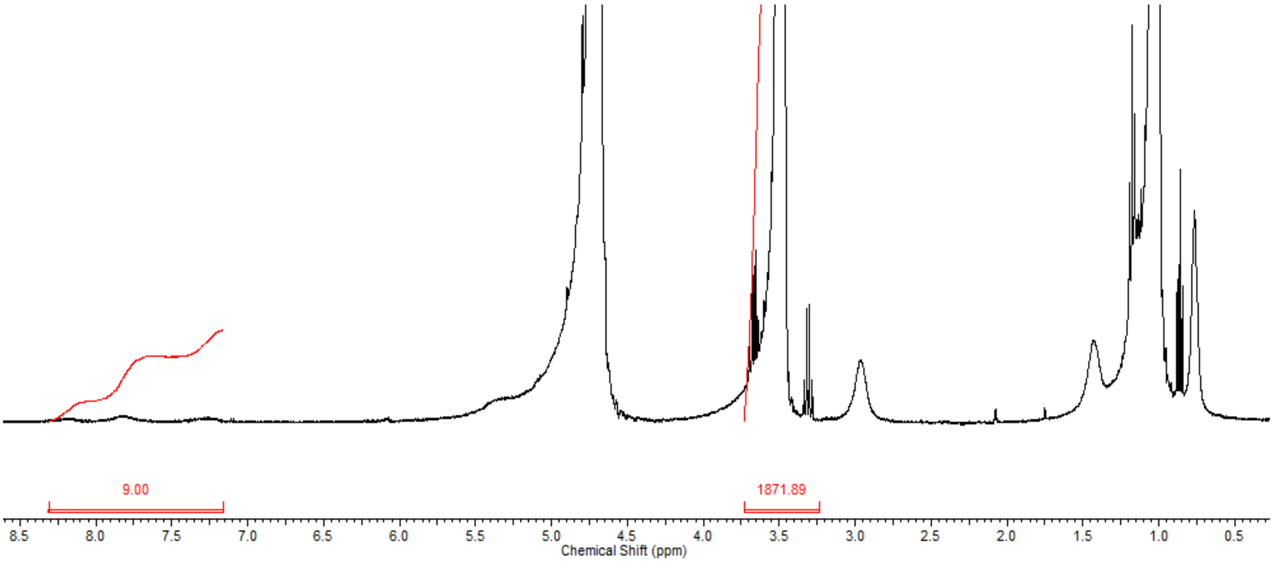

Figure S53. A ^{1}H NMR spectrum of compound **4** (1.51 mg, 0.0028 mM) in a solution of ethanol (25 µL, 0.43 mM) and D_2O (0.475 mL) with an extended delay (d_1 = 60 s). A 92 % reduction of compound observed upon comparative integration.

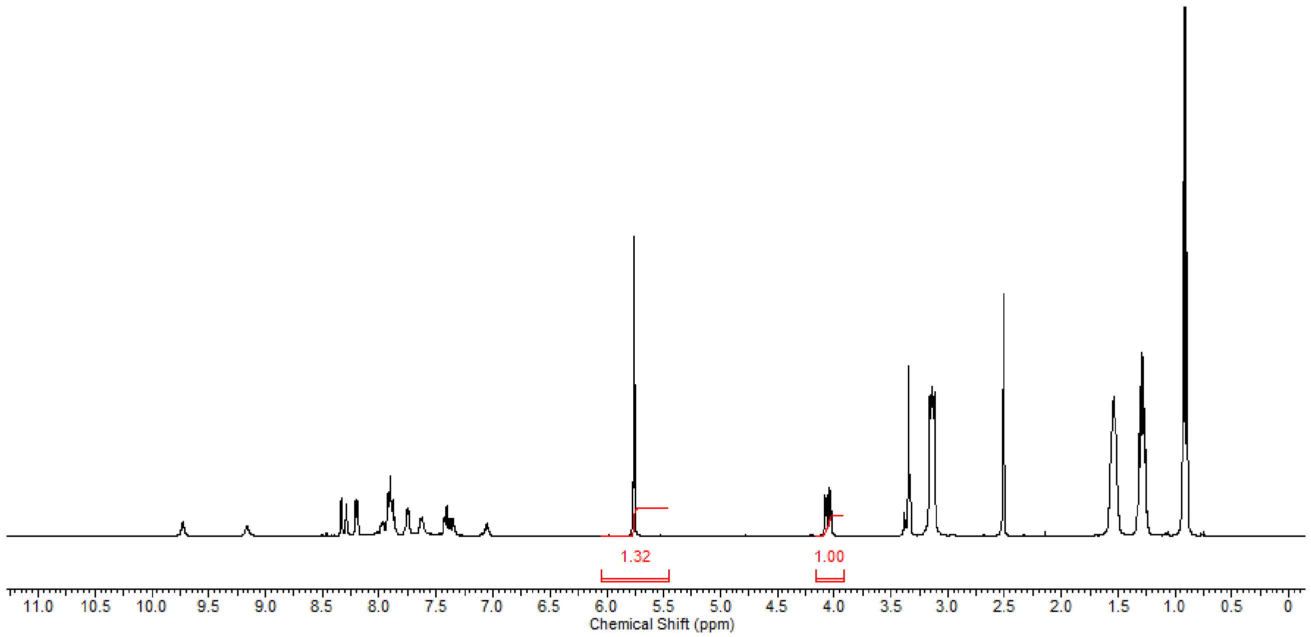

Figure S54. ^{1}H NMR of compound **1** (16.73 mg, 0.028 mM) and **2** (15.86 mg, 0.028 mM) in a solution of DMSO-d_6 (0.495 mL) and dichloromethane (5 µL, 0.078 mM) solution with an extended delay (d_1 = 60 s). No apparent loss of compound observed.

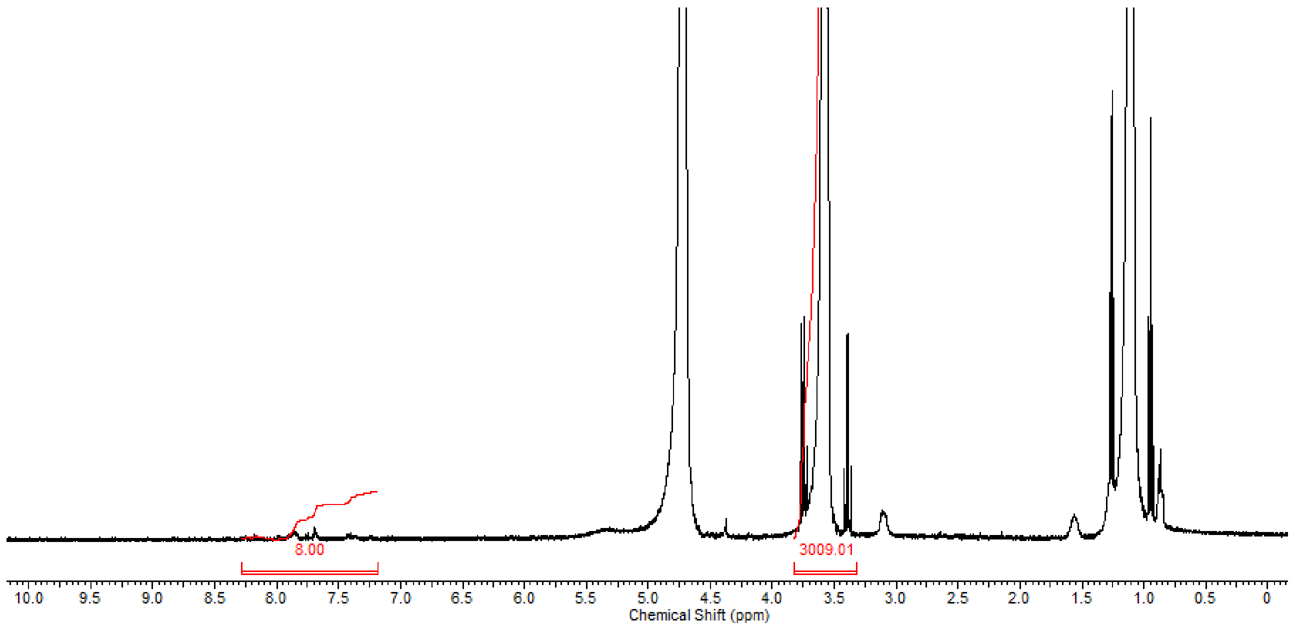

Figure S55. A ^{1}H NMR spectrum of compound **1** (0.89 mg, 0.0015 mM) and **2** (0.78 mg, 0.0014 mM) (both together 1.68 mg, 0.0029 mM) in solution of ethanol (25 µL, 0.43 mM) and D_2O (0.475 mL) with an extended delay (d_1 = 60 s). A 95 % reduction of compound observed upon comparative integration.

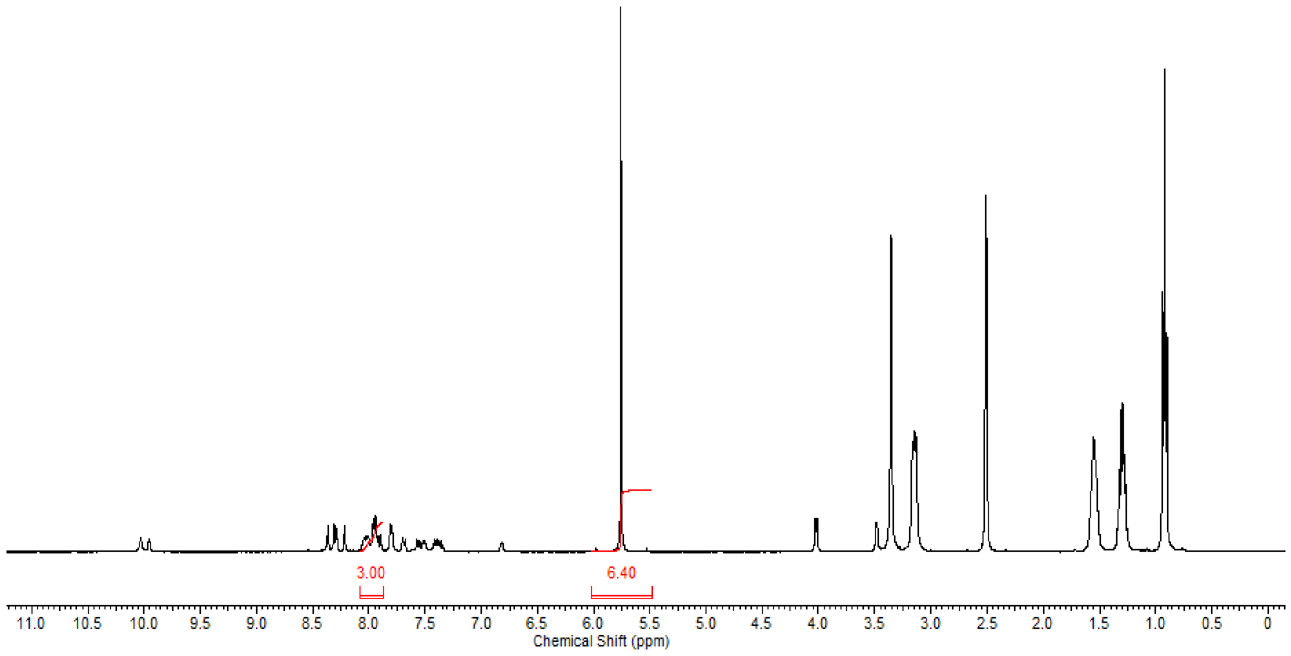

Figure S56. A ^{1}H NMR of compound **1** (8.40 mg, 0.014 mM) and **4** (7.54 mg, 0.014 mM) (both together 15.94 mg, 0.028 mM) in a solution of DMSO-d_6 (0.495 mL) and dichloromethane (5 µL, 0.078 mM) solution with an extended delay (d_1 = 60 s). The experiment was carried out at a lower concentration due to solubility. A 56% reduction of compound was observed upon comparative integration.

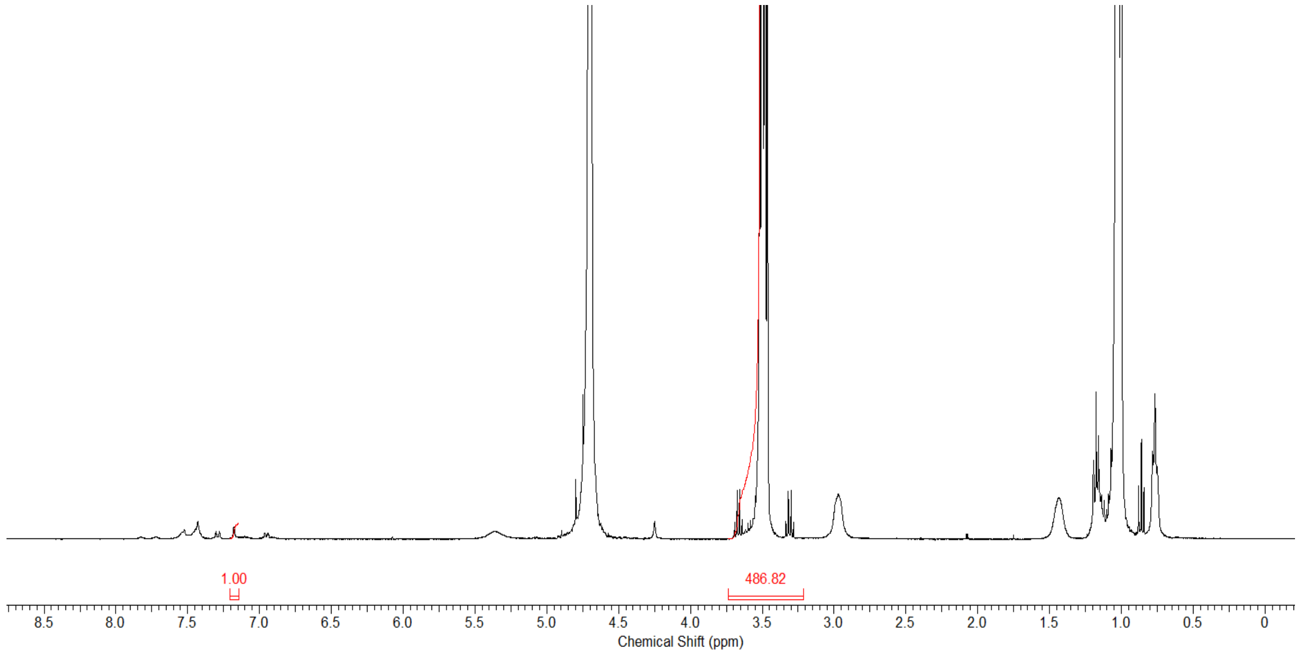

Figure S57. A ^{1}H NMR spectrum of compound **1** (0.838 mg, 0.0014 mM) and **4** (0.744 mg, 0.0014mM) (both together 1.58 mg, 0.0028 mM) in a solution of ethanol (25 µL, 0.43 mM) and D_2O (0.475 mL) with an extended delay (d_1 = 60 s). A 68% reduction of compound observed upon comparative integration.

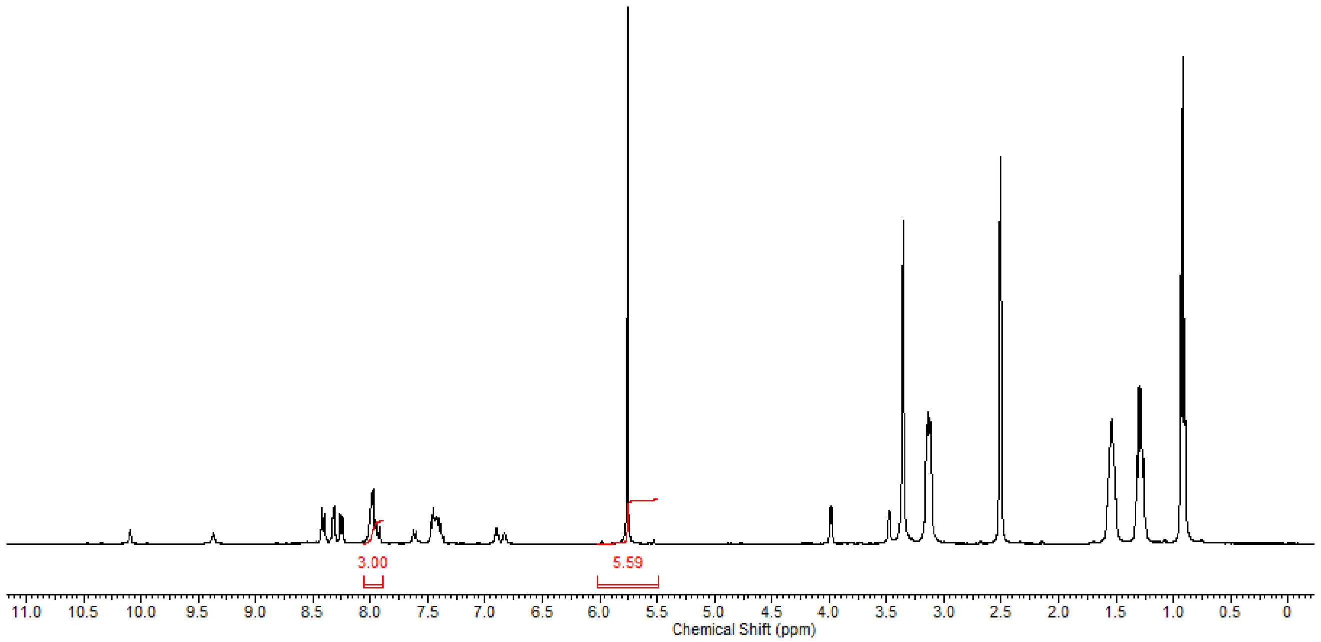

Figure S58. A ^{1}H NMR of compound **2** (7.95 mg, 0.014 mM) and **4** (7.40 mg, 0.014 mM) (both together 15.35 mg, 0.028 mM) in a solution of DMSO-d_6 (0.495 mL) and dichloromethane (5 µL, 0.078 mM) solution with an extended delay (d_1 = 60 s). The experiment was carried out at a lower concentration due to solubility. A 50% reduction of compound observed upon comparative integration.

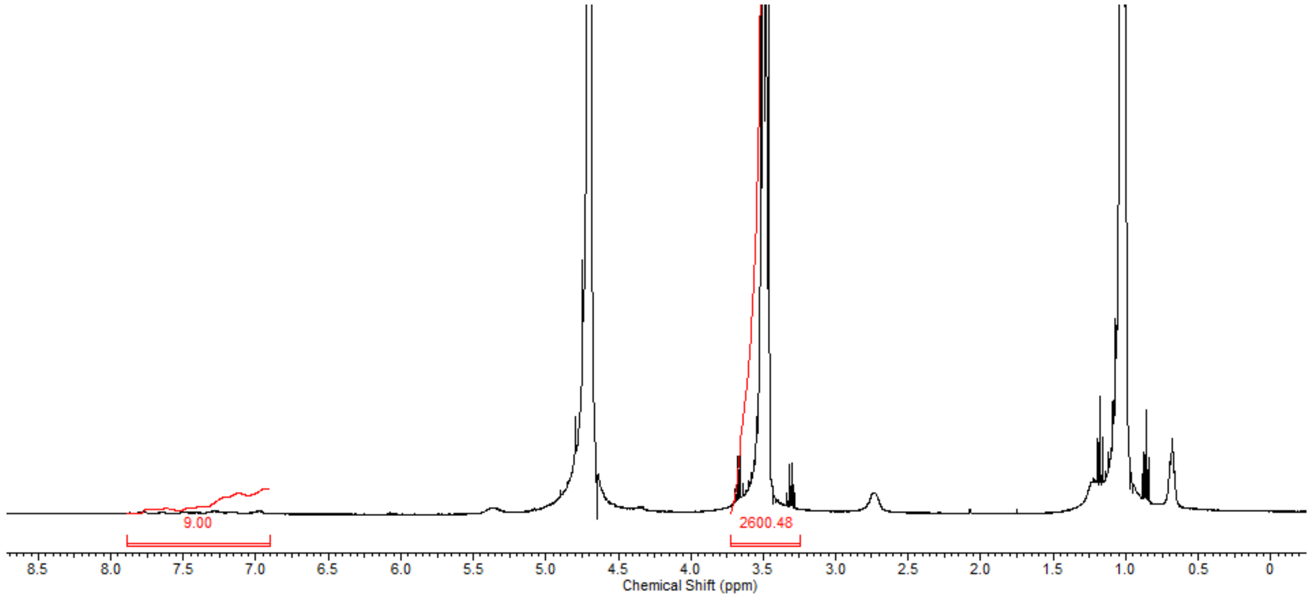

Figure S59. A ^{1}H NMR spectrum of compound **2** (0.75 mg, 0.0013 mM) and **4** (0.72 mg, 0.0013mM) (both together 1.47 mg, 0.0026 mM) in a solution of ethanol (25 µL, 0.43 mM) and D_2O (0.475 mL) with an extended delay (d_1 = 60 s). A 94 % reduction of compound observed upon comparative integration.

Fluorescence Data

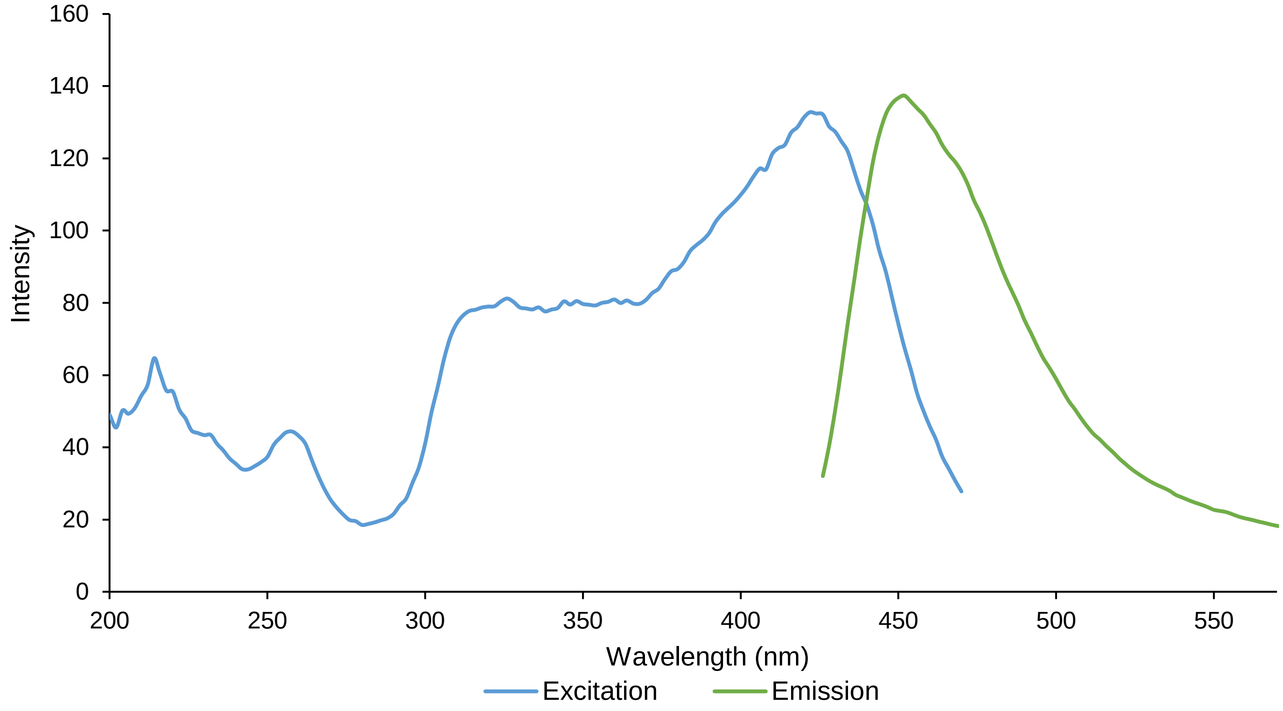

Figure S60. Fluorescence excitation and emission spectra of compound **1** (1 mM) in EtOH: H_2O (1: 19).

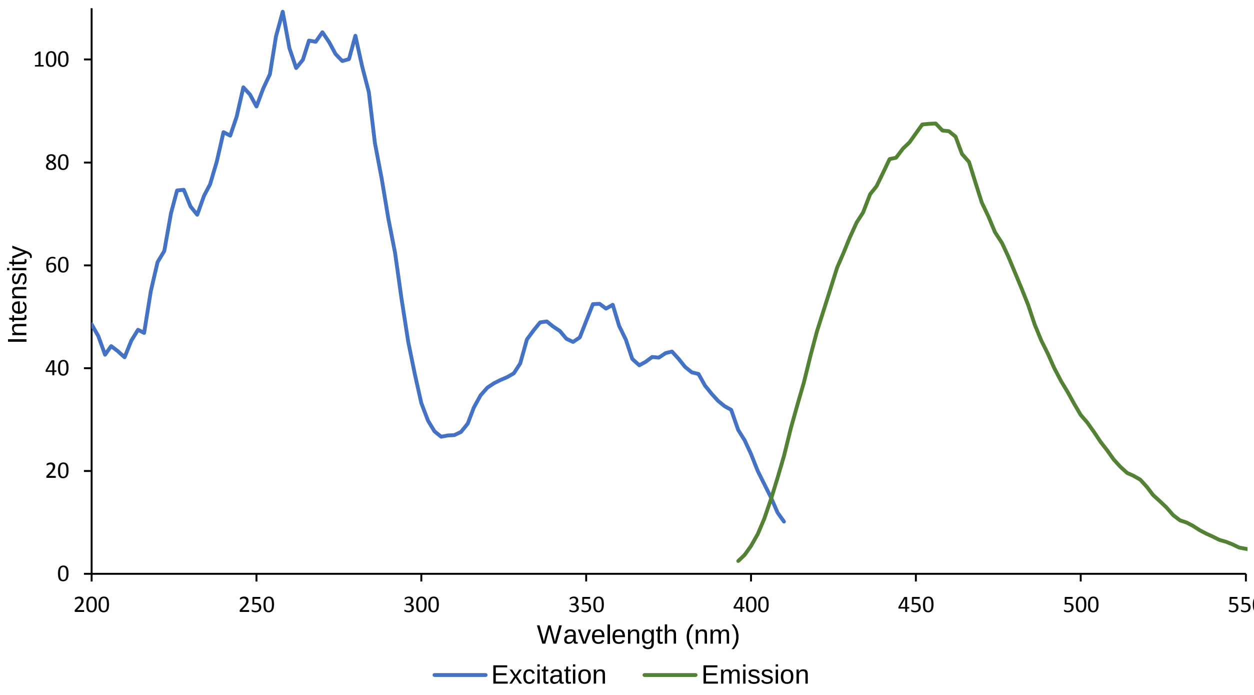

Figure S61. Fluorescence excitation and emission spectra of compound **4** (0.1 mM) in EtOH: H_2O (1: 19).

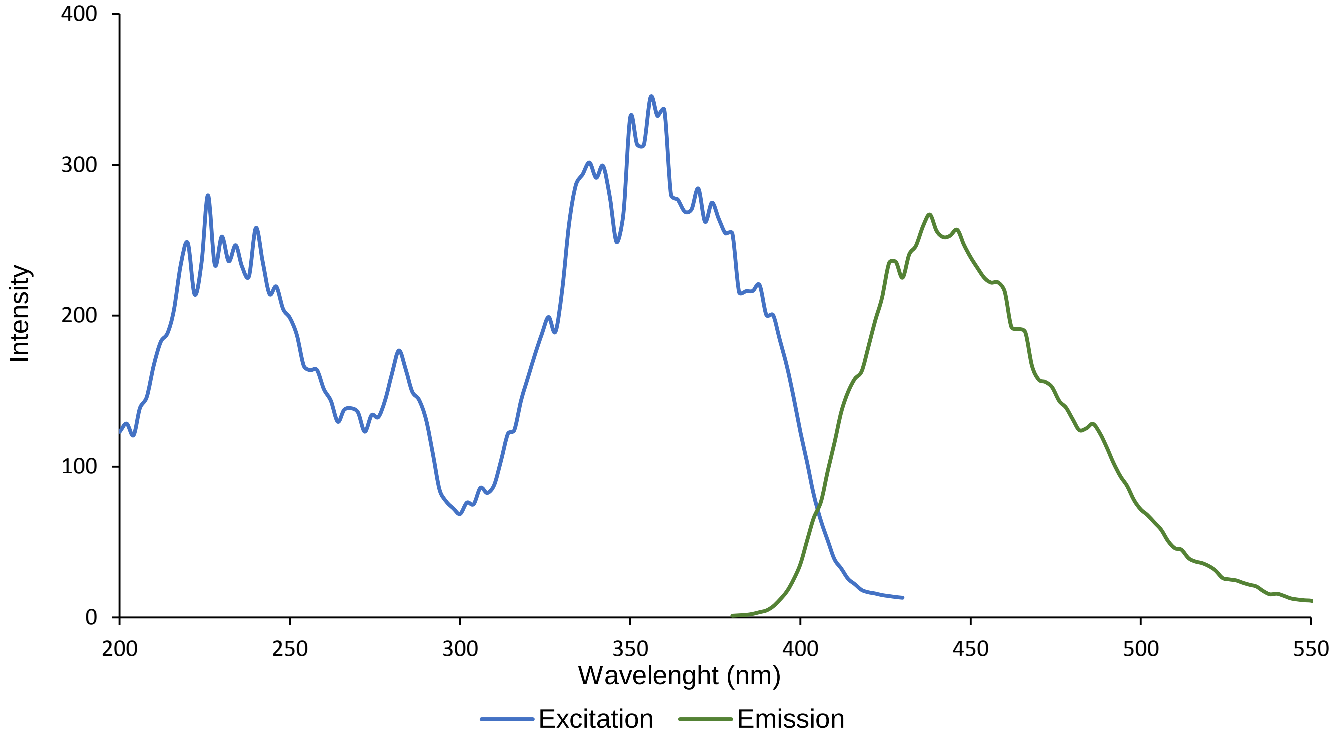

Figure S62. Fluorescence emission and excitation spectra of compound **1** (0.5 mM) and **2** (0.5 mM) in EtOH: H_2O (1: 19).

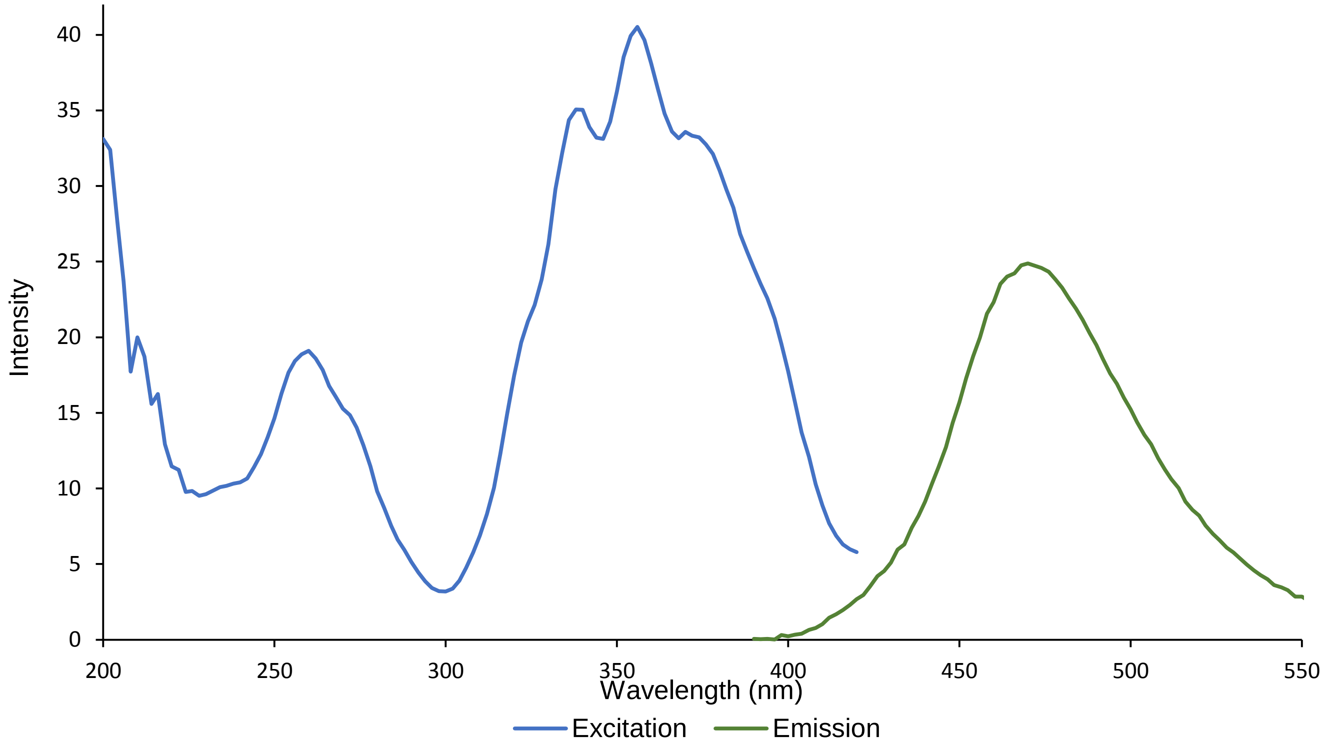

Figure S63. Fluorescence emission and excitation spectra of compound **1** (0.5 mM) and **4** (0.5 mM) in EtOH: H_2O (1: 19).

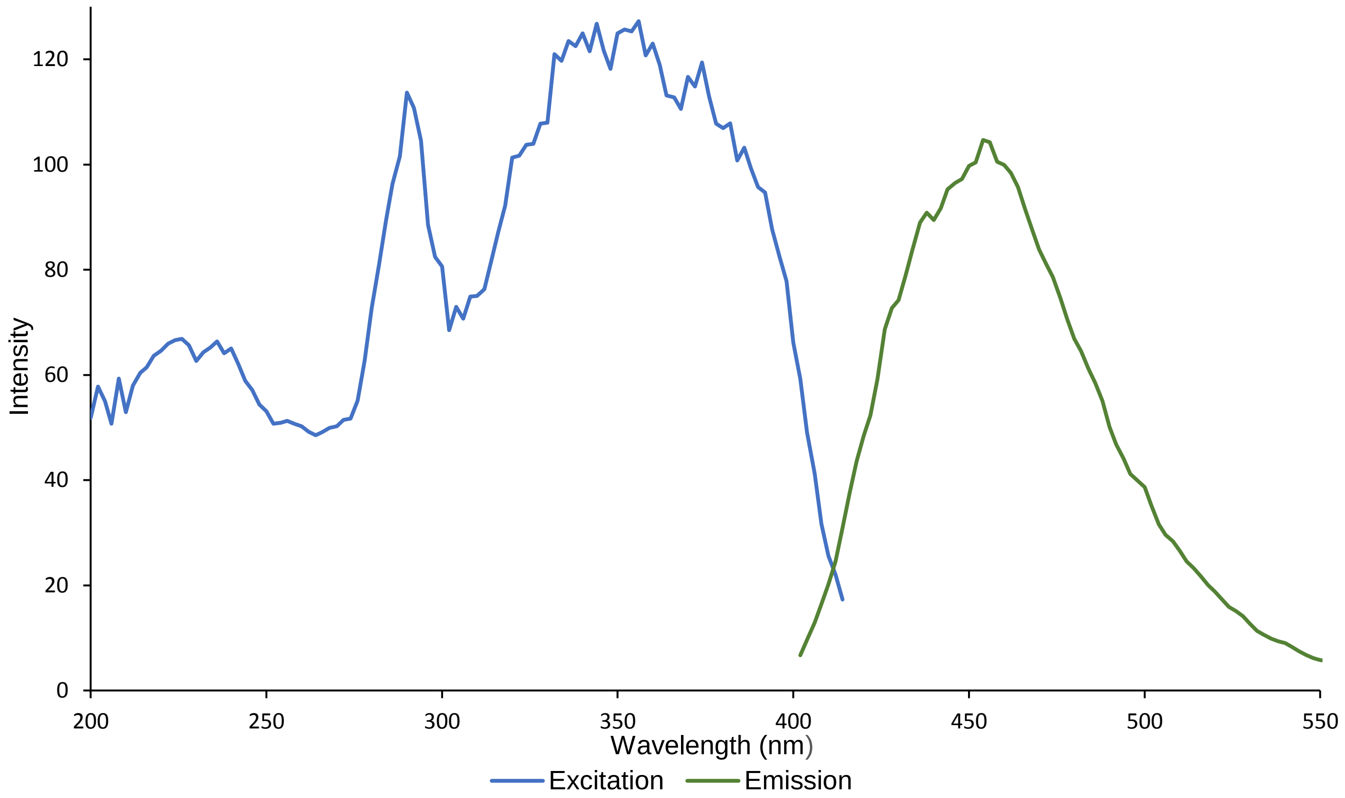

Figure S64. Fluorescence spectra of compound **2** (0.5 mM) and **4** (0.5 mM) in EtOH: H_2O (1: 19).

Table S4. The fluorescence excitation and emission values for compounds **1**, **2**, **4** and mixtures of **1** and **2**, **1** and **4**, **2** and **4** in EtOH: H_2O (1: 19).

compound	$\lambda_{Ex\ (nm)}$	$\lambda_{Em\ (nm)}$	$\Delta\lambda_{ST}$
**1**	422	476	54
**2**[6]	260	437	177
**4**	270	456	186
**1** + **2**	356	438	82
**1** + **4**	356	470	114
**2** + **4**	356	454	98

Surface Tension Measurements and Critical Micelle Concentration (CMC) Determination

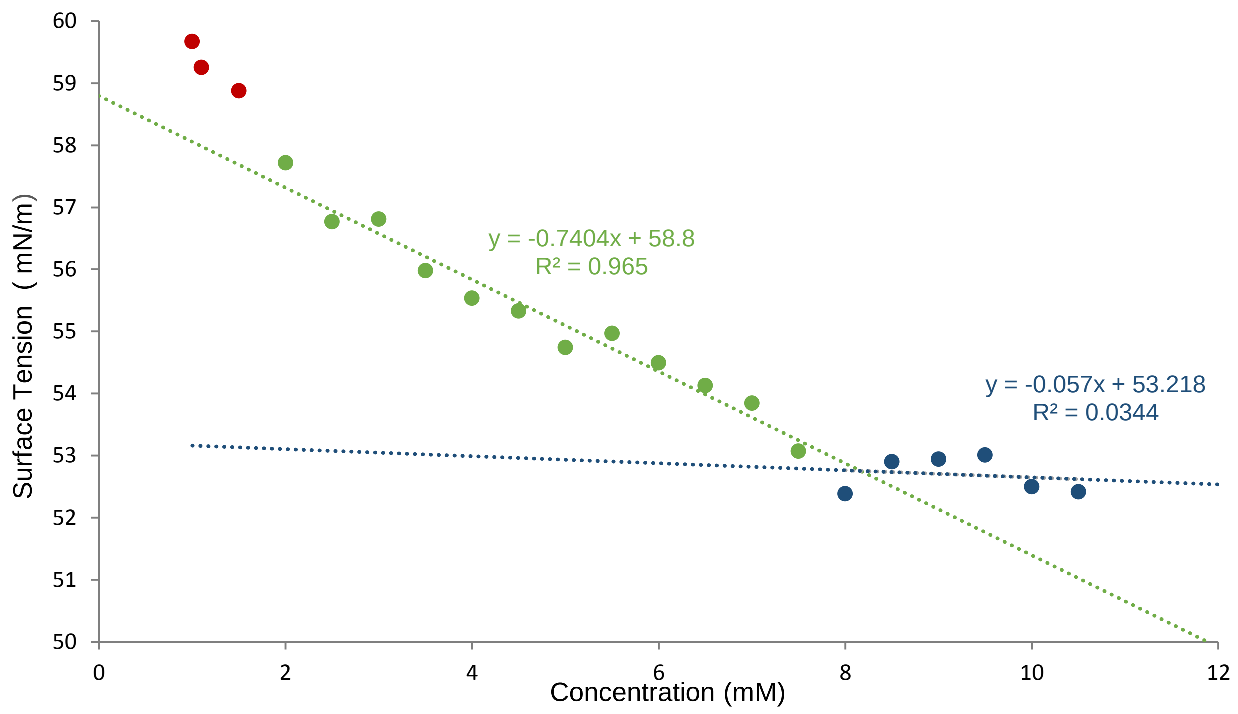

Figure S65. Calculation of CMC (**8.17** mM) for compound **1** in an EtOH:H_2O (1:19) mixture using surface tension measurements.

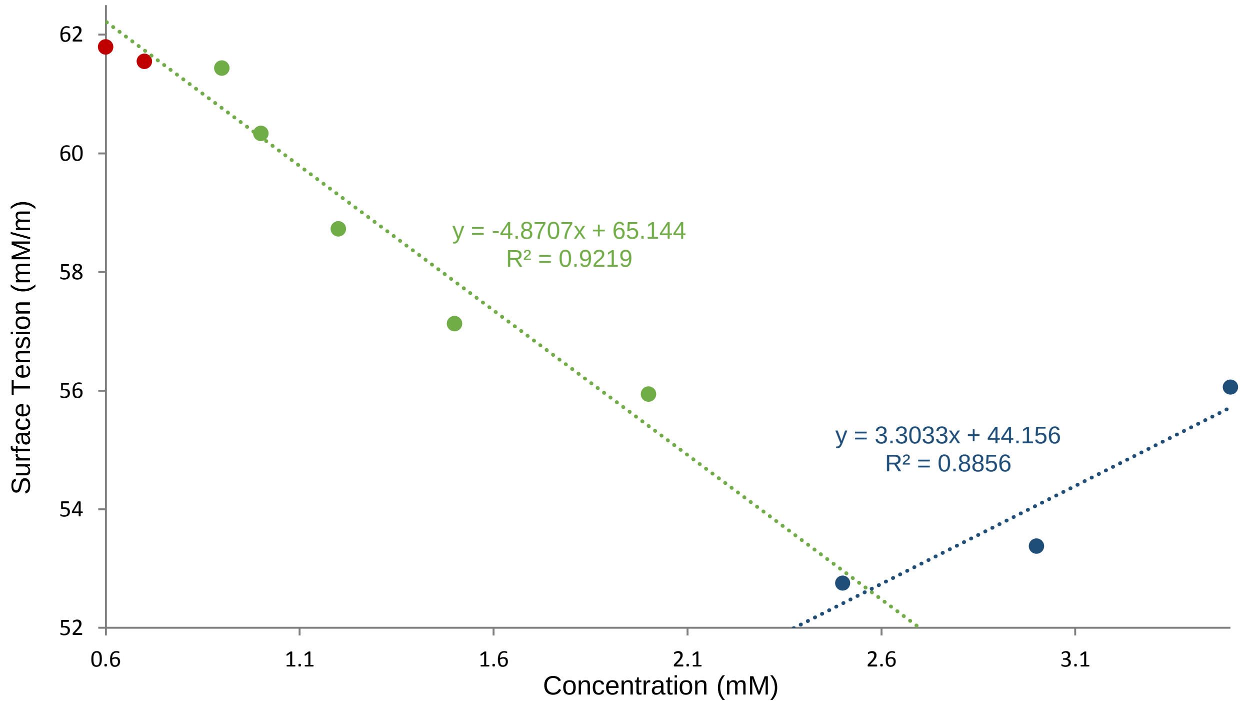

Figure S66. Calculation of CMC (**2.57** mM) for compound **4** in an EtOH:H_2O (1:19) mixture using surface tension measurements.

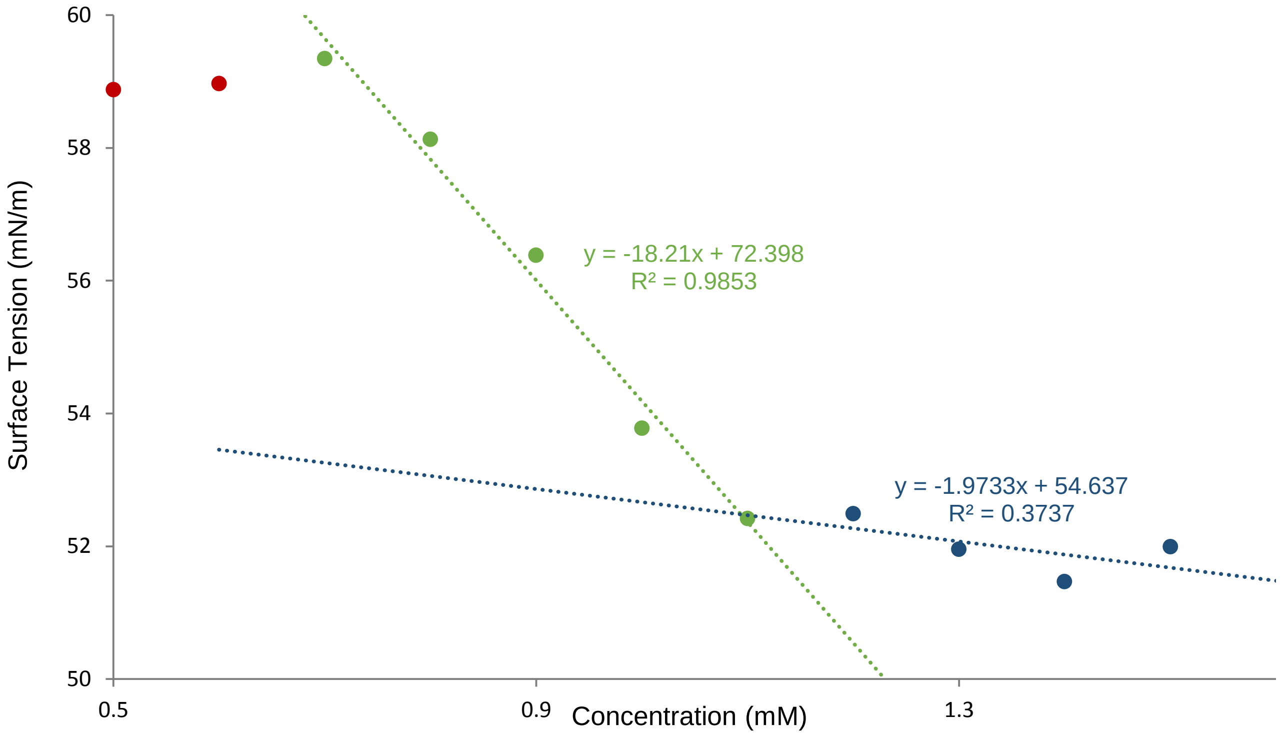

Figure S67. Calculation of CMC (**1.09** mM) for compounds **1** and **2** (1:1 mix) in an EtOH:H_2O (1:19) mixture using surface tension measurements.

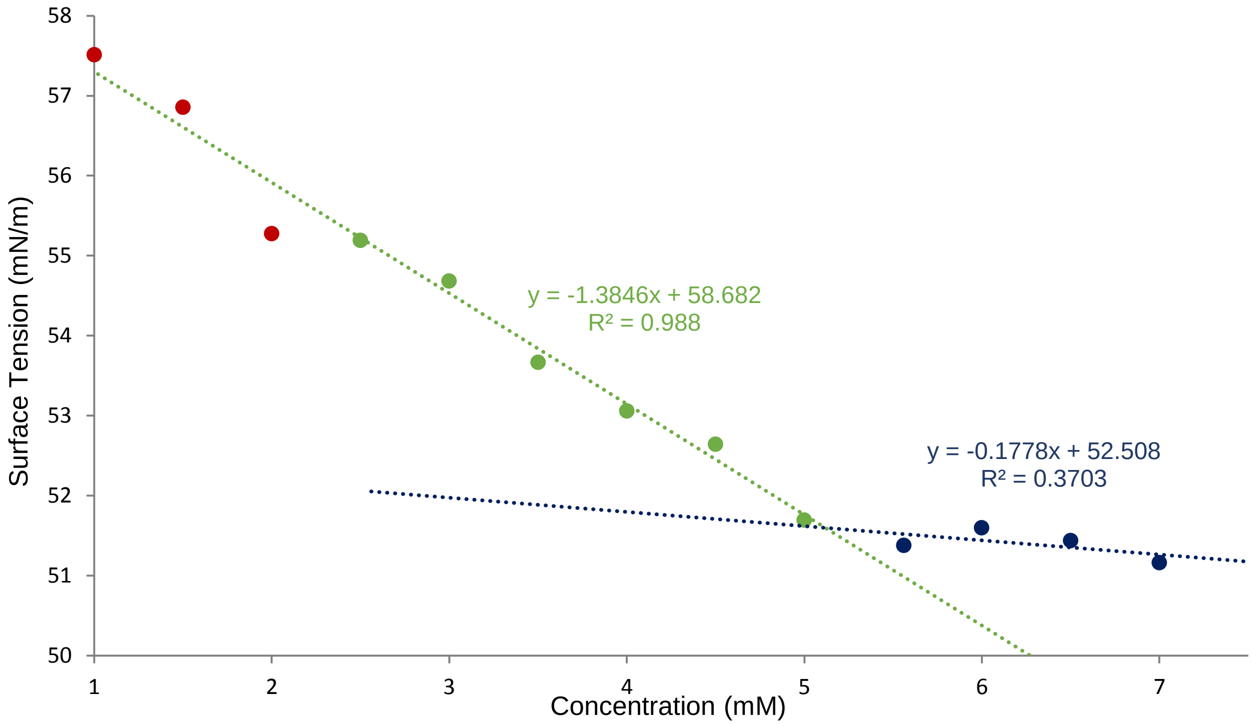

Figure S68. Calculation of CMC (**5.12** mM) for compound **1** and **4** (1:1 mix) in an EtOH:H_2O (1:19) mixture using surface tension measurements.

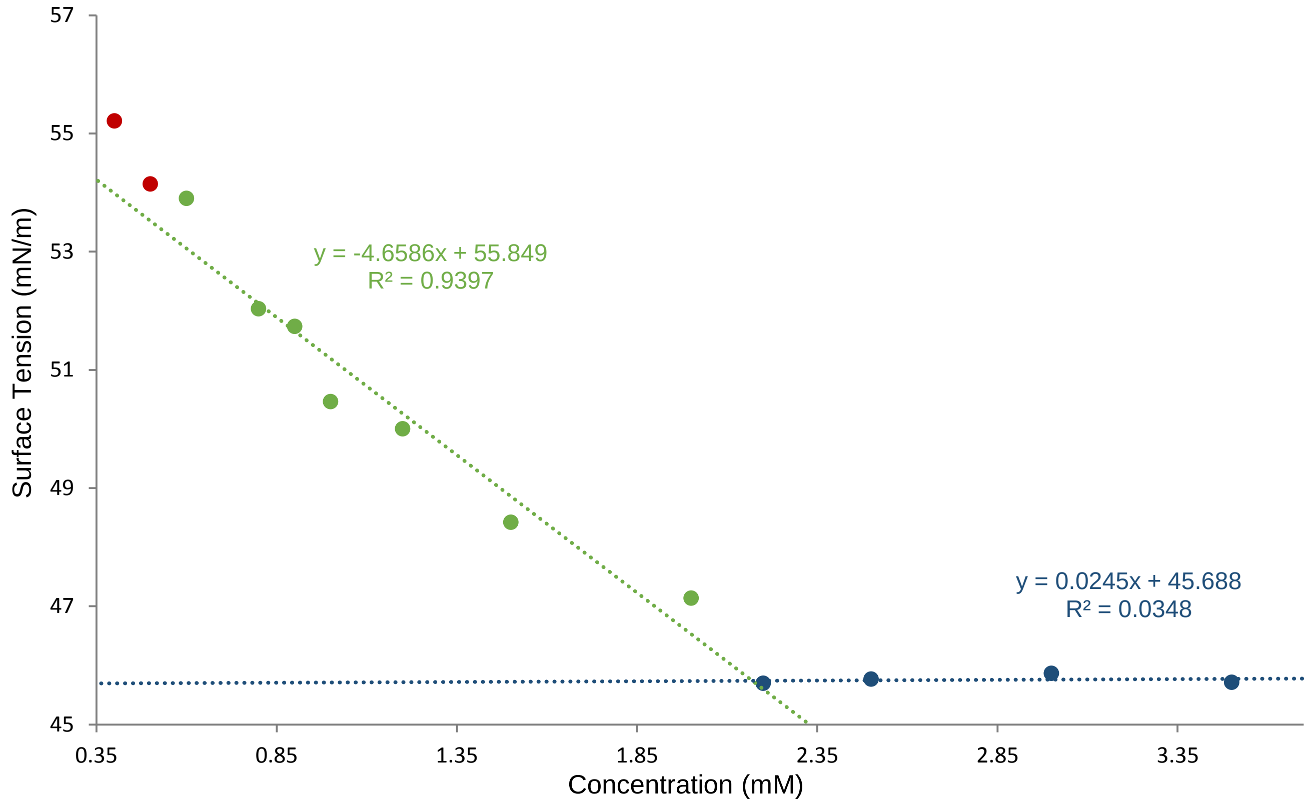

Figure S69. Calculation of CMC (**2.17** mM) for compound **2** and **4** (1:1 mix) in an EtOH:H_2O (1:19) mixture using surface tension measurements.

Dynamic Light Scattering (H_2O:EtOH 19:1)

Particle Size Distribution

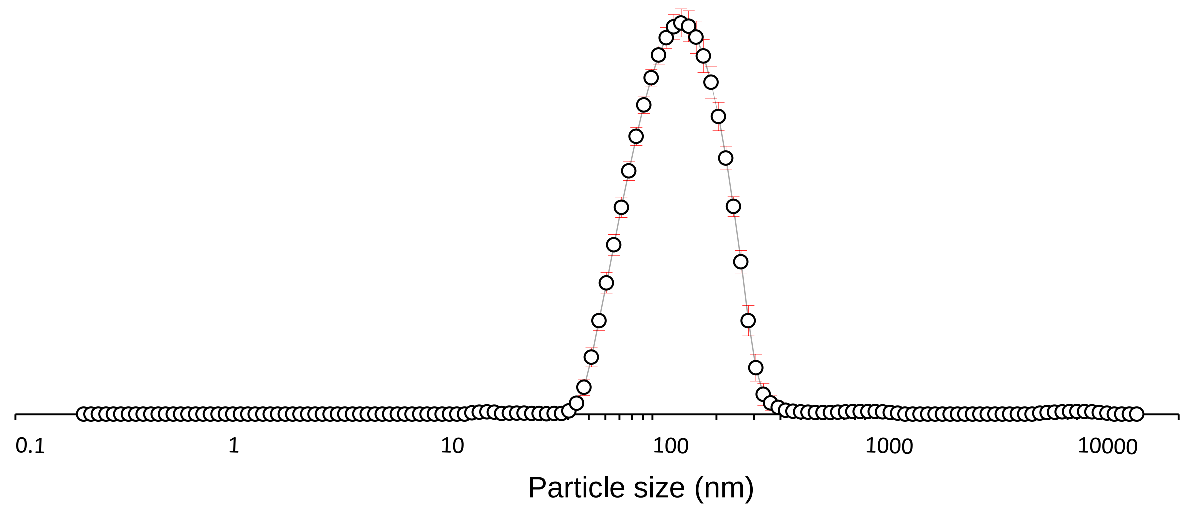

Figure S70. The average intensity particle size distribution calculated using 10 DLS runs for compound **1** (5.56 mM) in an EtOH:H_2O (1:19) solution at 298 K.

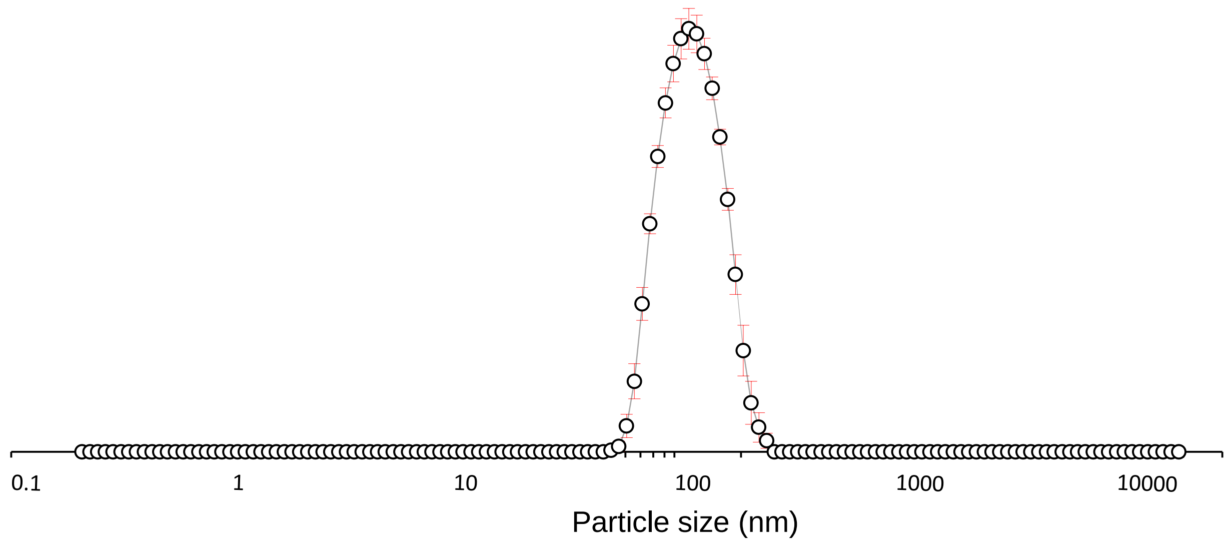

Figure S71. The average intensity particle size distribution calculated using 10 DLS runs for compound **1** (0.56 mM) in an EtOH:H_2O (1:19) solution at 298 K.

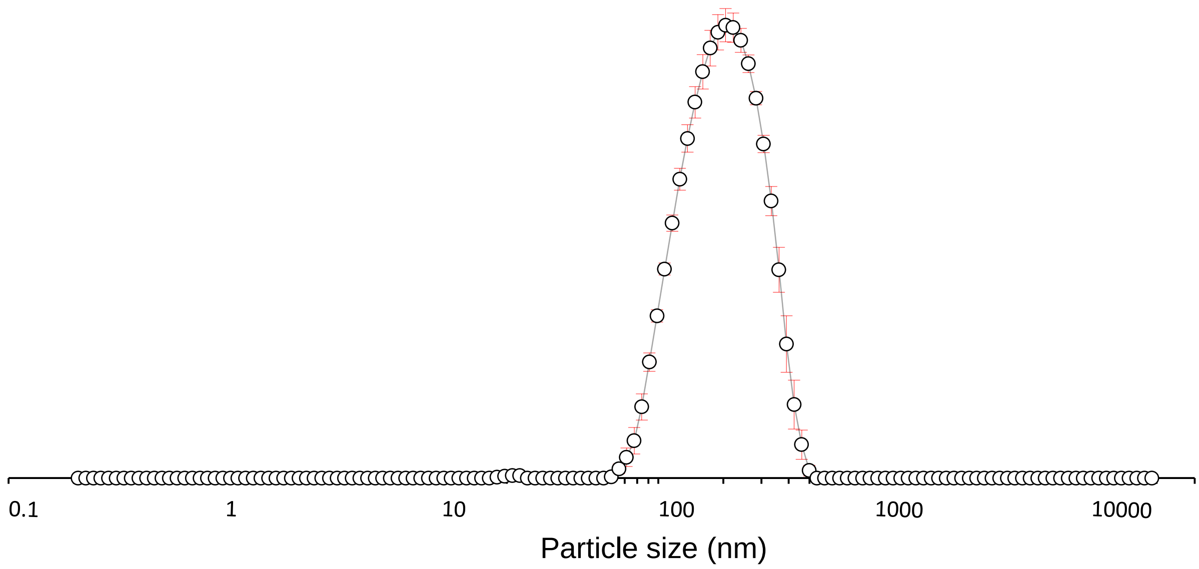

Figure S72. The average intensity particle size distribution calculated using 10 DLS runs for compound **2** (5.56 mM) in an EtOH:H_2O (1:19) solution at 298 K.

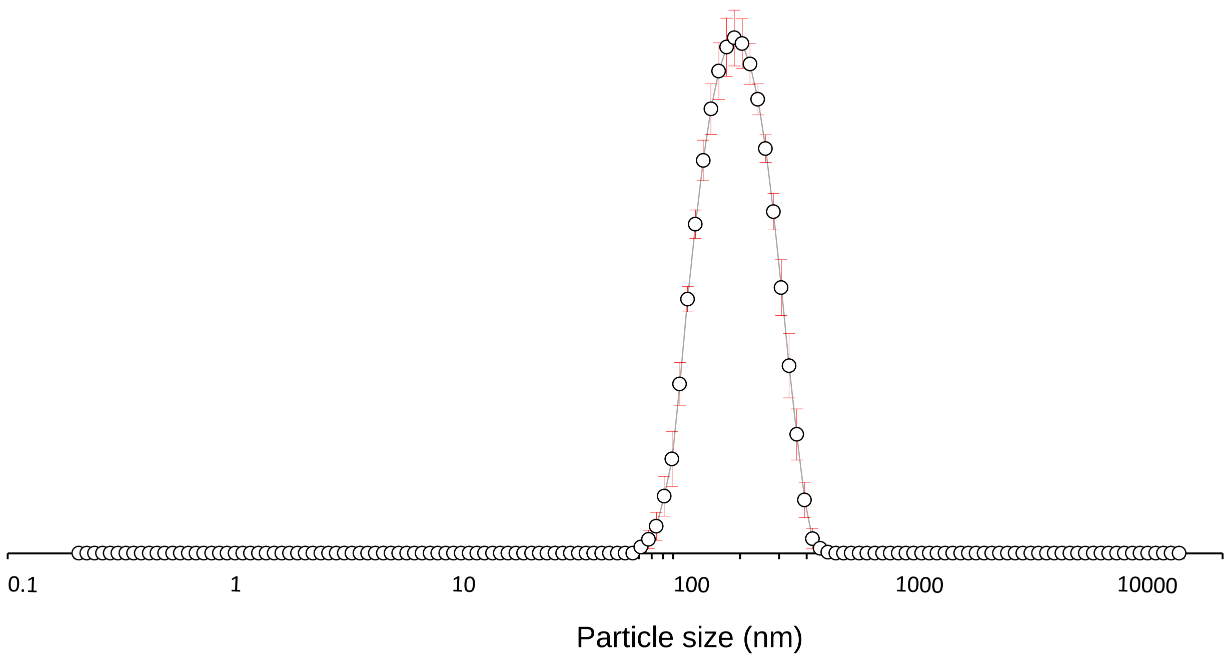

Figure S73. The average intensity particle size distribution calculated using 10 DLS runs for compound **2** (0.56 mM) in an EtOH:H_2O (1:19) solution at 298 K.

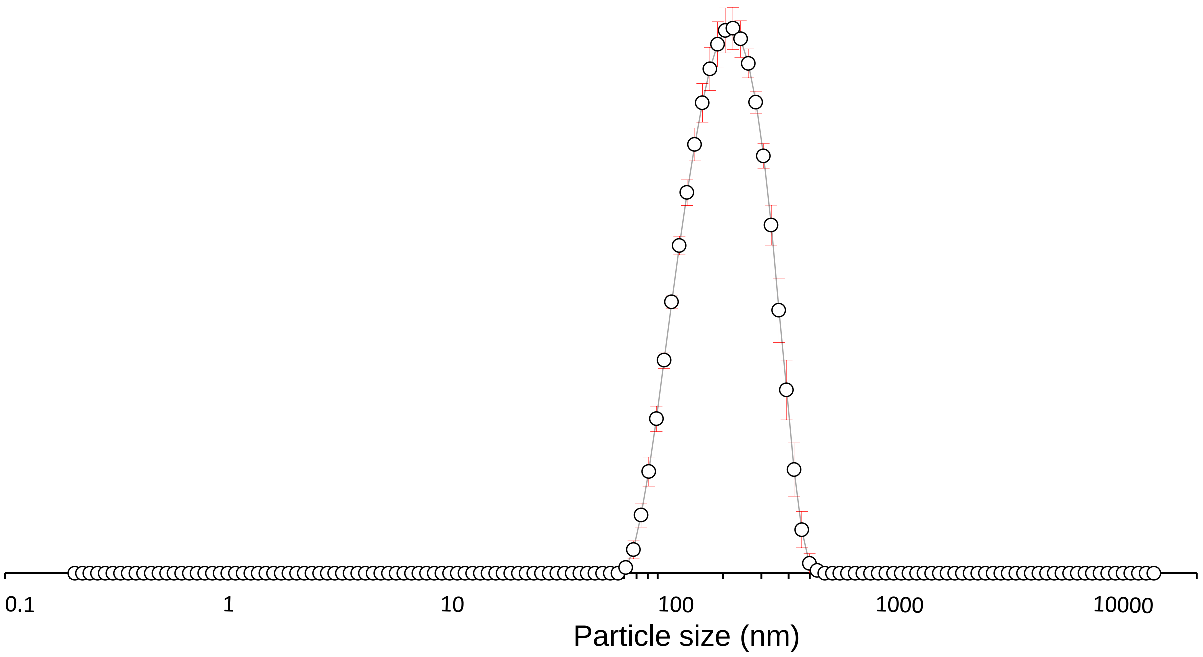

Figure S74. The average intensity particle size distribution calculated using 10 DLS runs for compound **4** (5.56 mM) in an EtOH:H_2O (1:19) solution at 298 K.

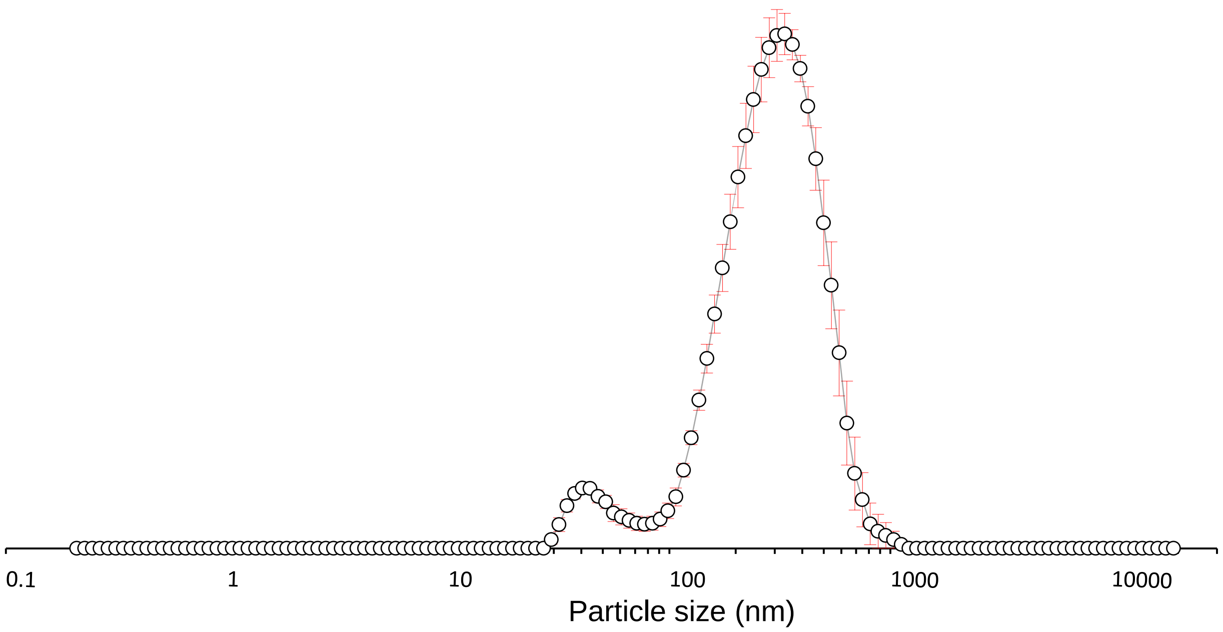

Figure S75. The average intensity particle size distribution calculated using 10 DLS runs for compound **4** (0.56 mM) in an EtOH:H_2O (1:19) solution at 298 K.

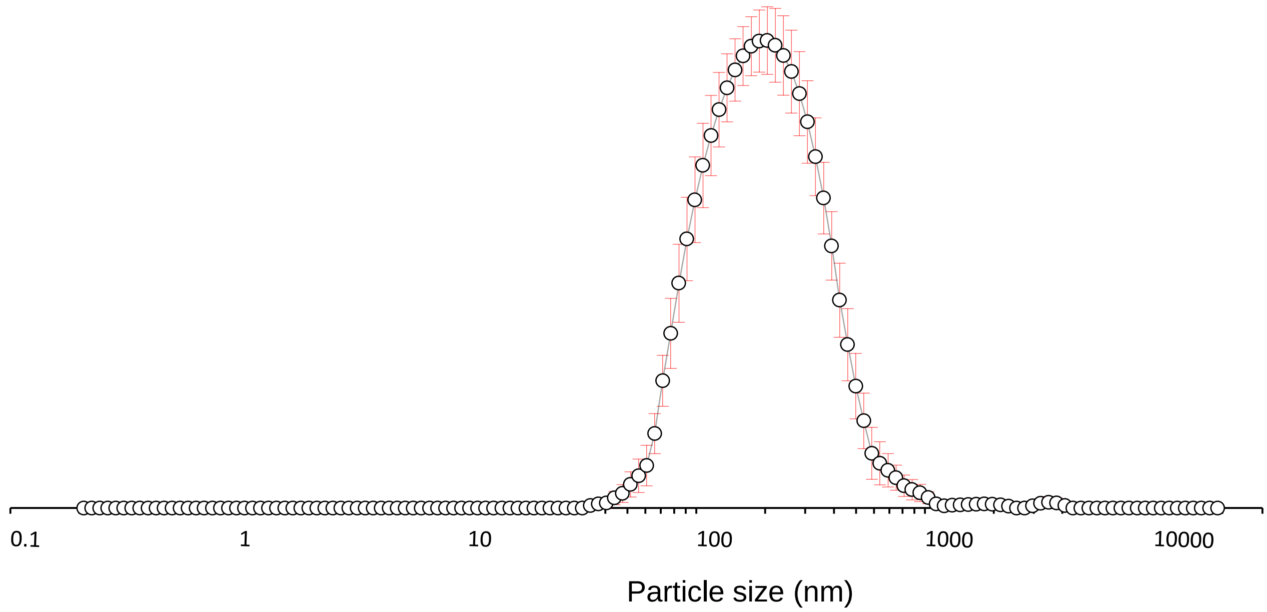

Figure S76. The average intensity particle size distribution calculated using 10 DLS runs for compounds **1** and **2** in a 1:1 mixture (total molar concentration 0.56 mM) and a EtOH:H_2O (1:19) solution at 298 K. The average intensity particle size distribution for compounds **1** and **2** (1:1 mix) at 5.56mM could not be obtained due to solubility.

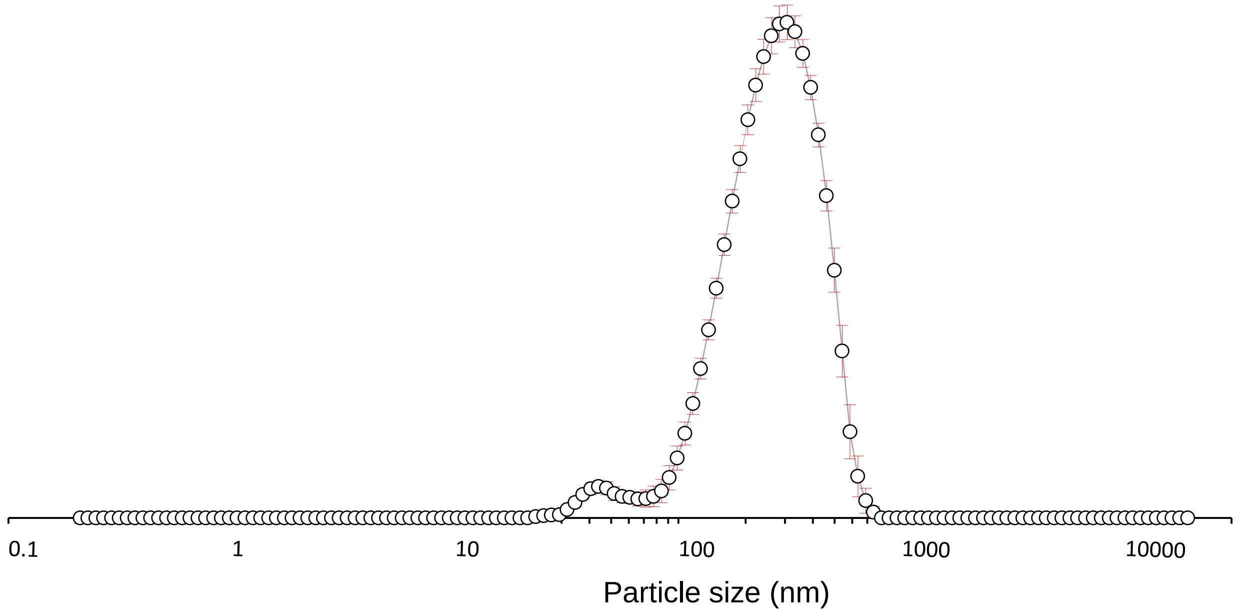

Figure S77. The average intensity particle size distribution calculated using 10 DLS runs for compounds **1** and **4** in a 1:1 mixture (total molar concentration 5.56 mM) and a EtOH:H_2O (1:19) solution at 298 K.

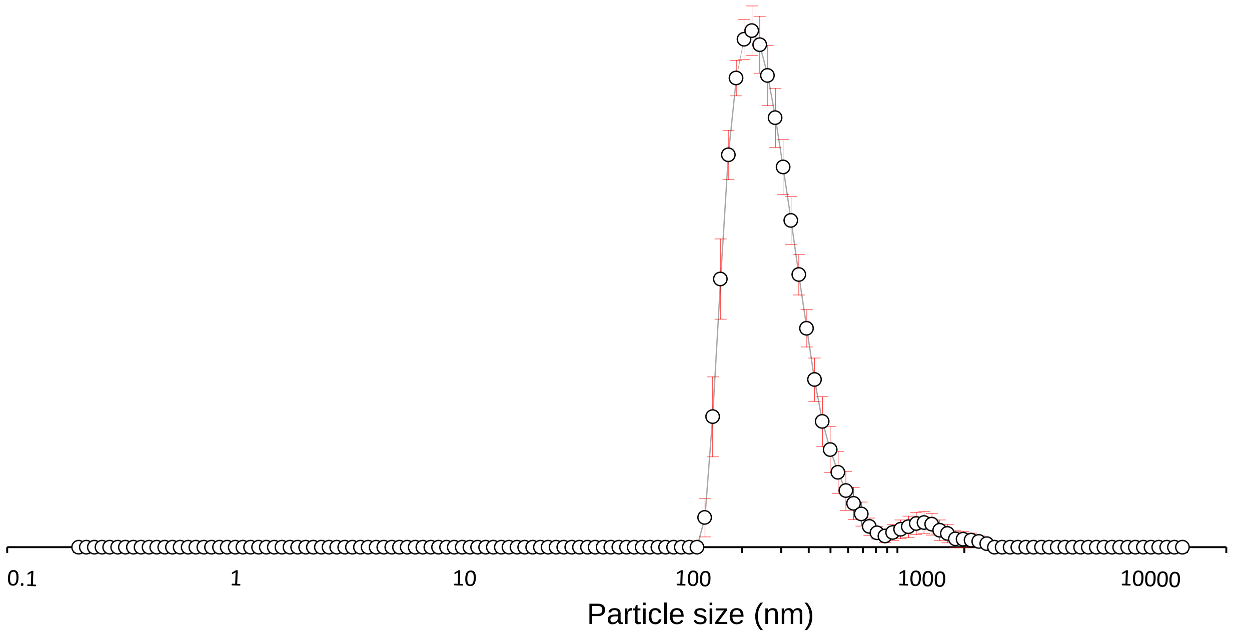

Figure S78. The average intensity particle size distribution calculated using 10 DLS runs for compounds **1** and **4** in a 1:1 mixture (total molar concentration 0.56 mM) and a EtOH:H_2O (1:19) solution at 298 K.

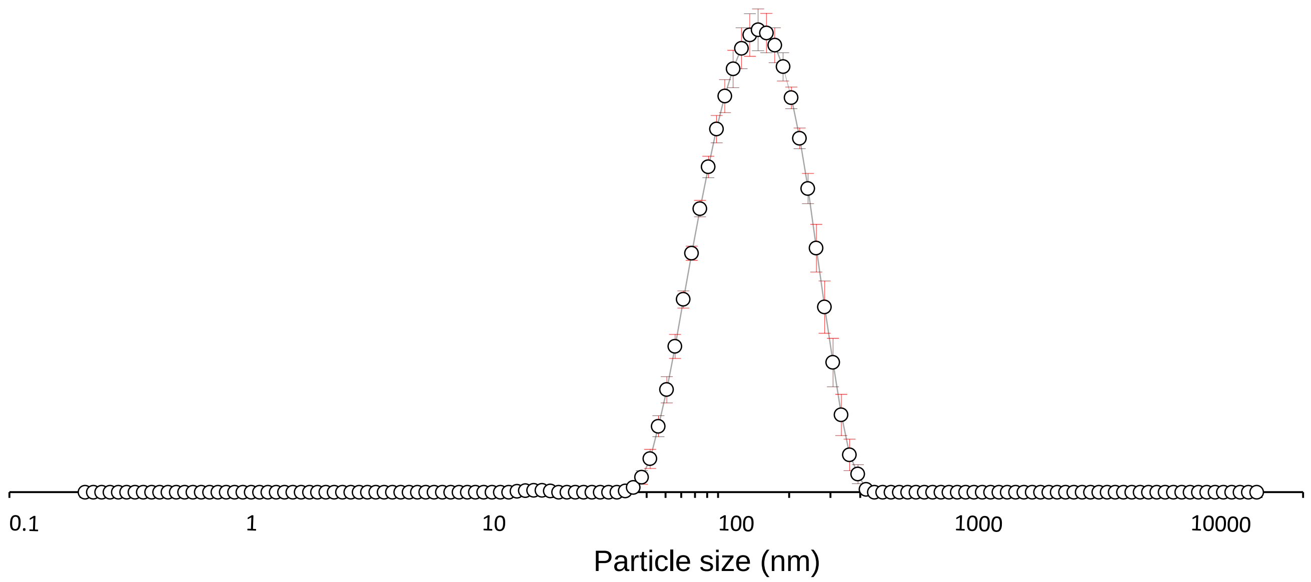

Figure S79. The average intensity particle size distribution calculated using 10 DLS runs for compounds **2** and **4** in a 1:1 mixture (total molar concentration 5.56 mM) and a EtOH:H_2O (1:19) solution at 298 K.

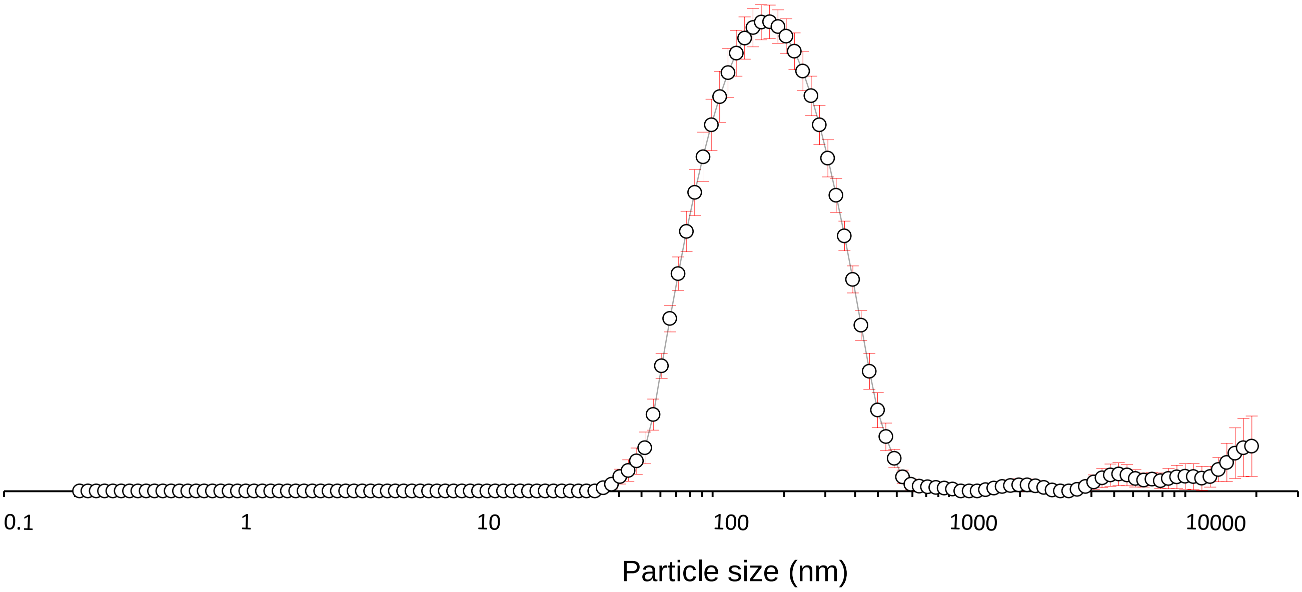

Figure S80. The average intensity particle size distribution calculated using 10 DLS runs for compounds **2** and **4** in a 1:1 mixture (total molar concentration 0.56 mM) and a EtOH:H_2O (1:19) solution at 298 K.

Correlation Function Data

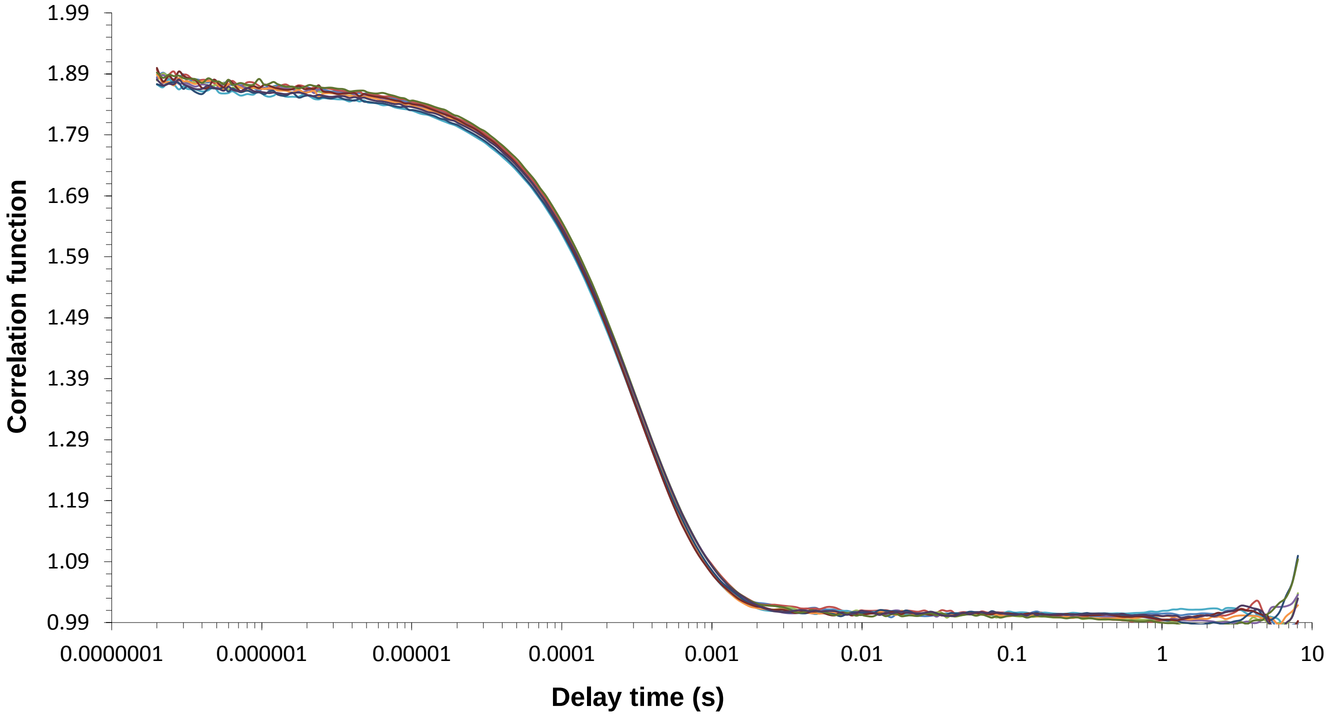

Figure S81. Correlation function data for 10 DLS runs of compound **1** (5.56 mM) in an EtOH:H_2O (1:19) solution at 298 K.

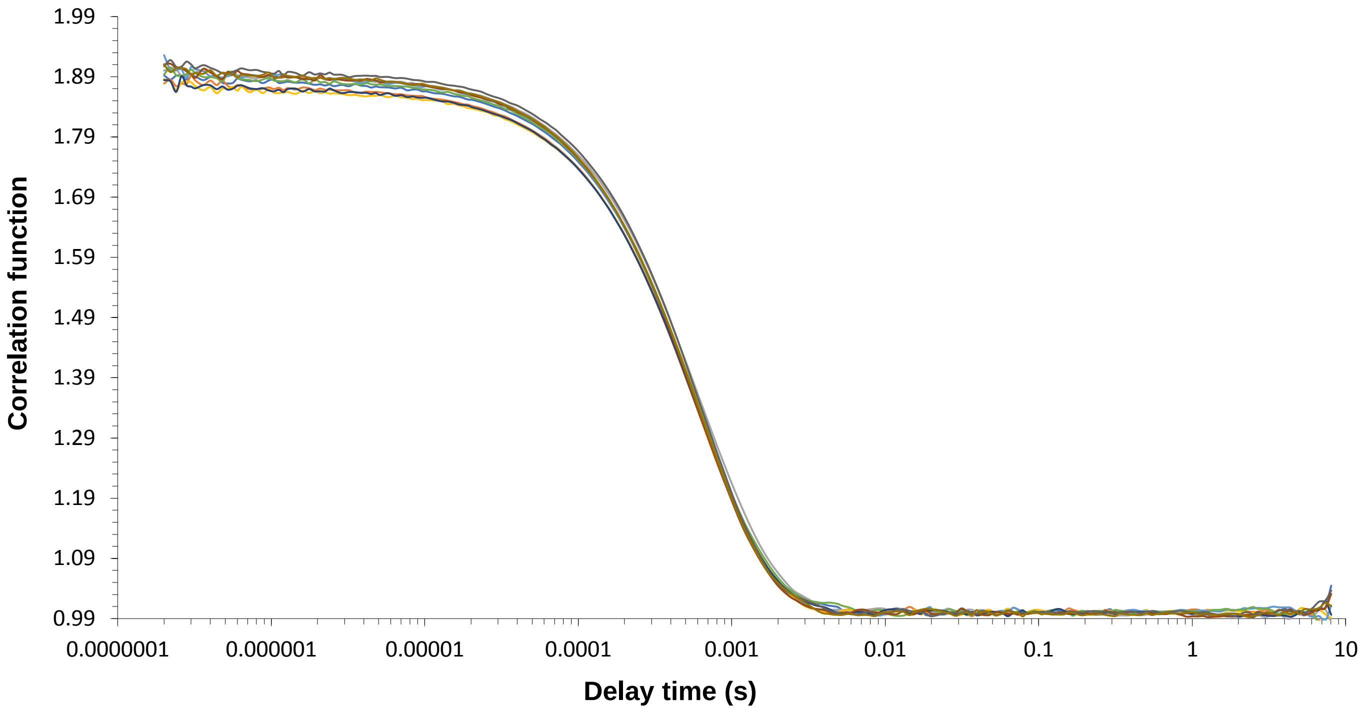

Figure S82. Correlation function data for 10 DLS runs of compound **1** (0.56 mM) in an EtOH:H_2O (1:19) solution at 298 K.

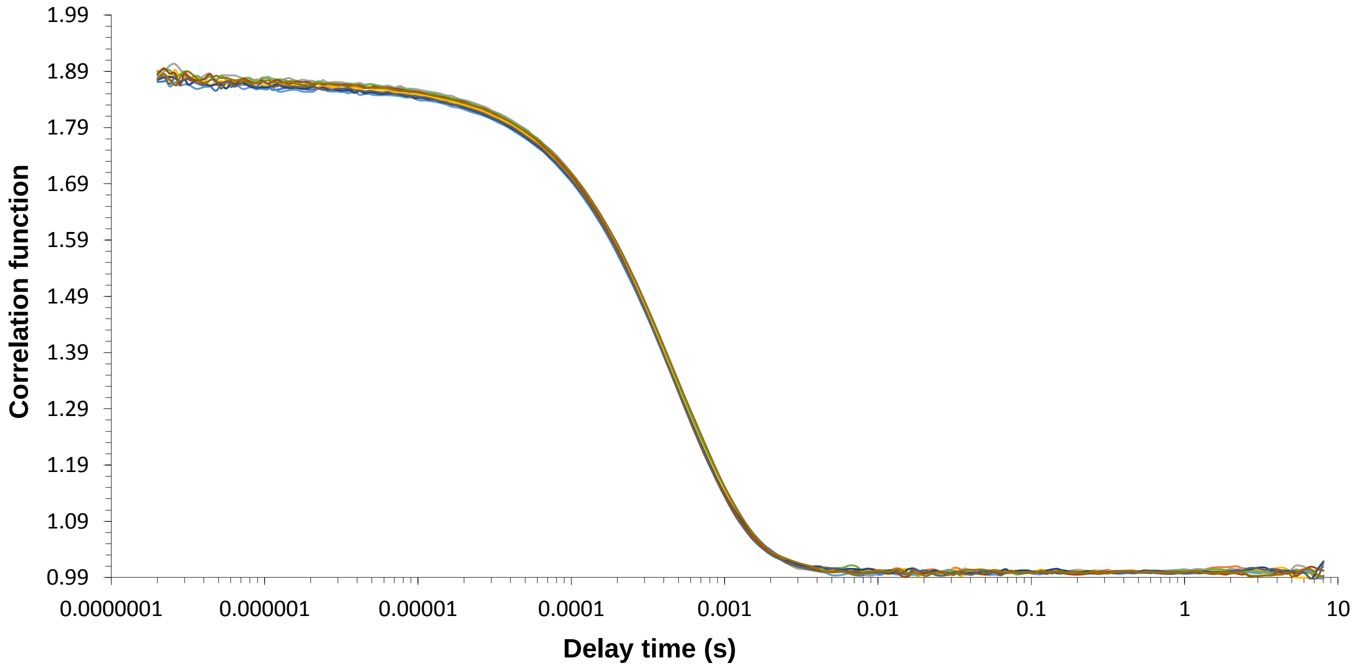

Figure S83. Correlation function data for 10 DLS runs of compound **2** (5.56 mM) in an EtOH:H_2O (1:19) solution at 298 K.

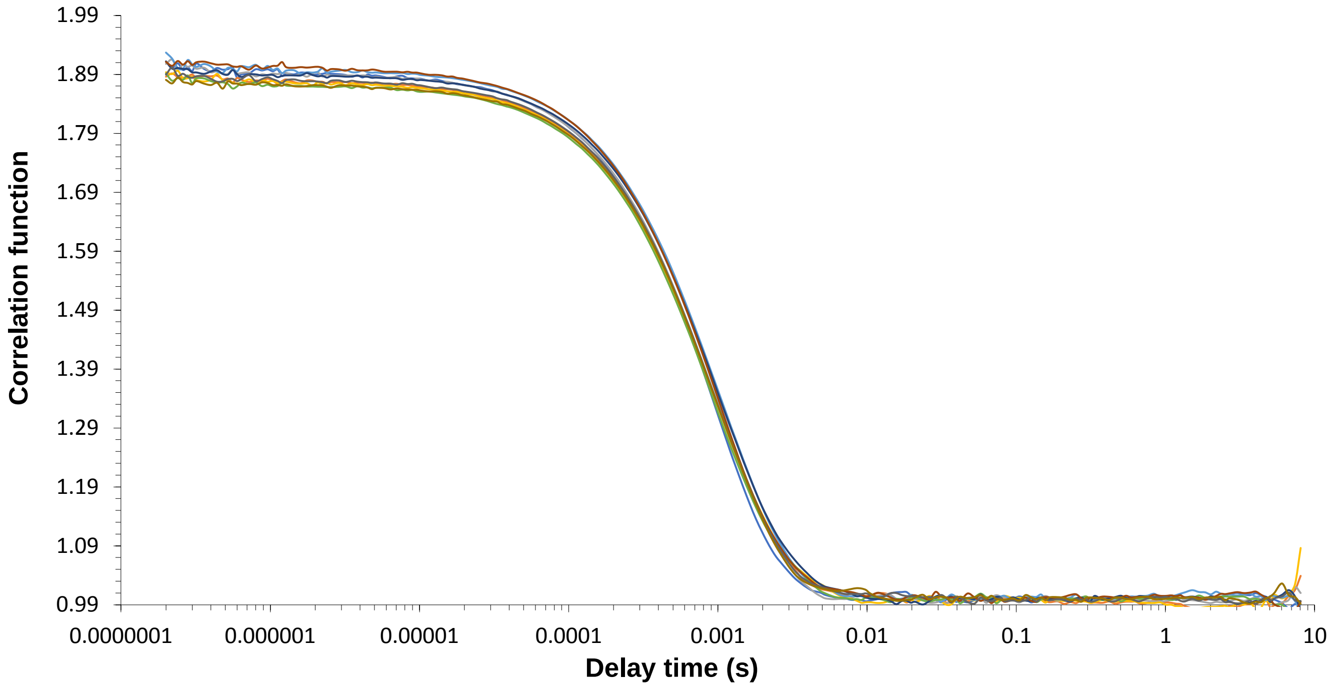

Figure S84. Correlation function data for 10 DLS runs of compound **2** (0.56 mM) in an $EtOH:H_2O$ (1:19) solution at 298 K.

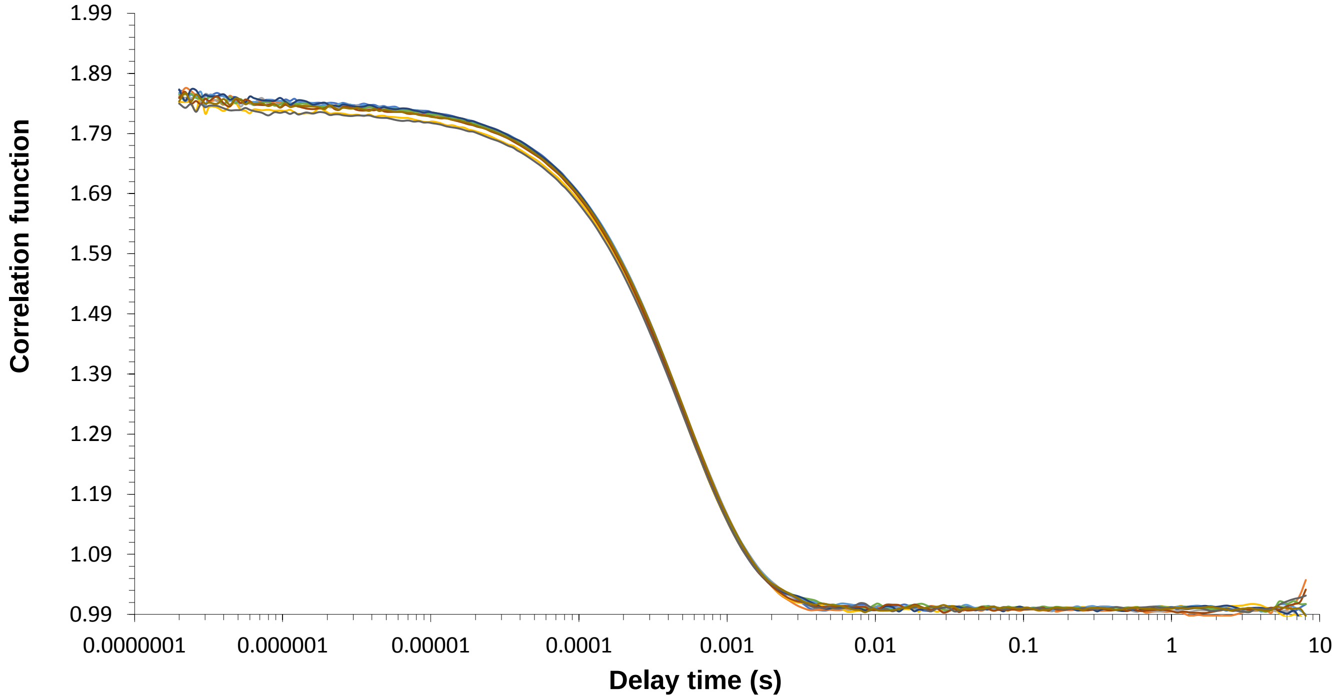

Figure S85. Correlation function data for 10 DLS runs of compound **4** (5.56 mM) in an $EtOH:H_2O$ (1:19) solution at 298 K.

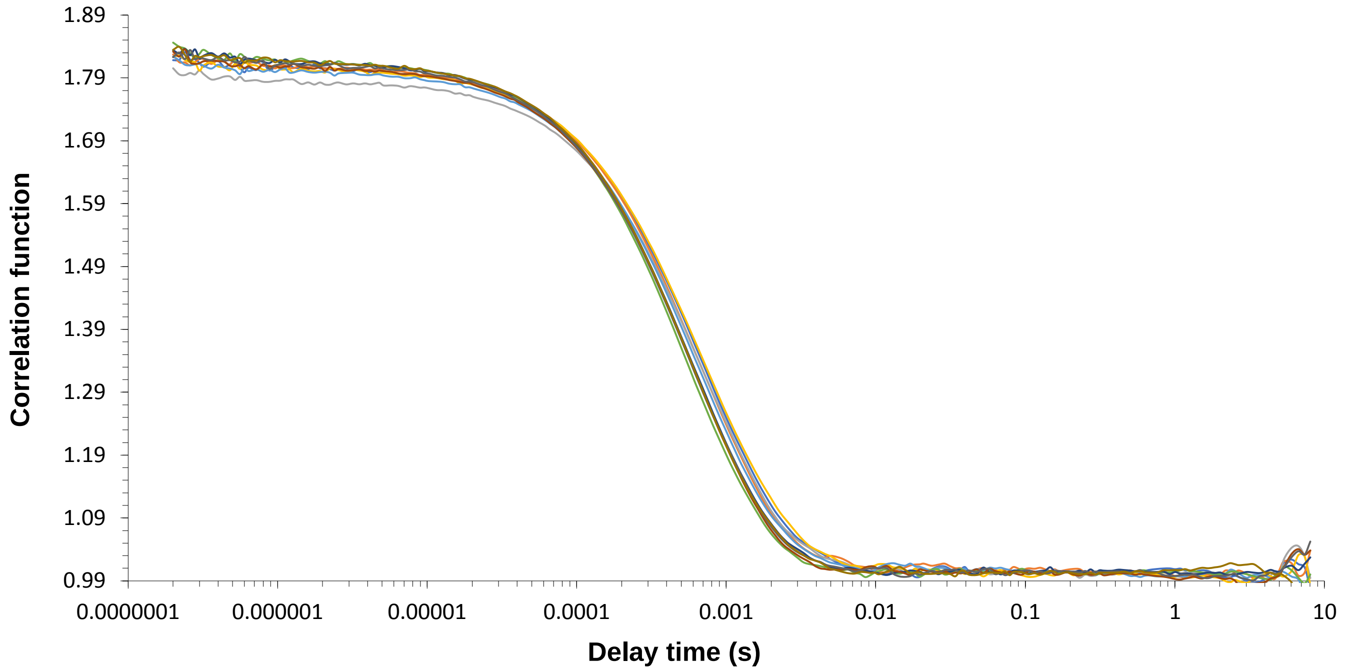

Figure S86. Correlation function data for 10 DLS runs of compound **4** (0.56 mM) in an EtOH:H_2O (1:19) solution at 298 K.

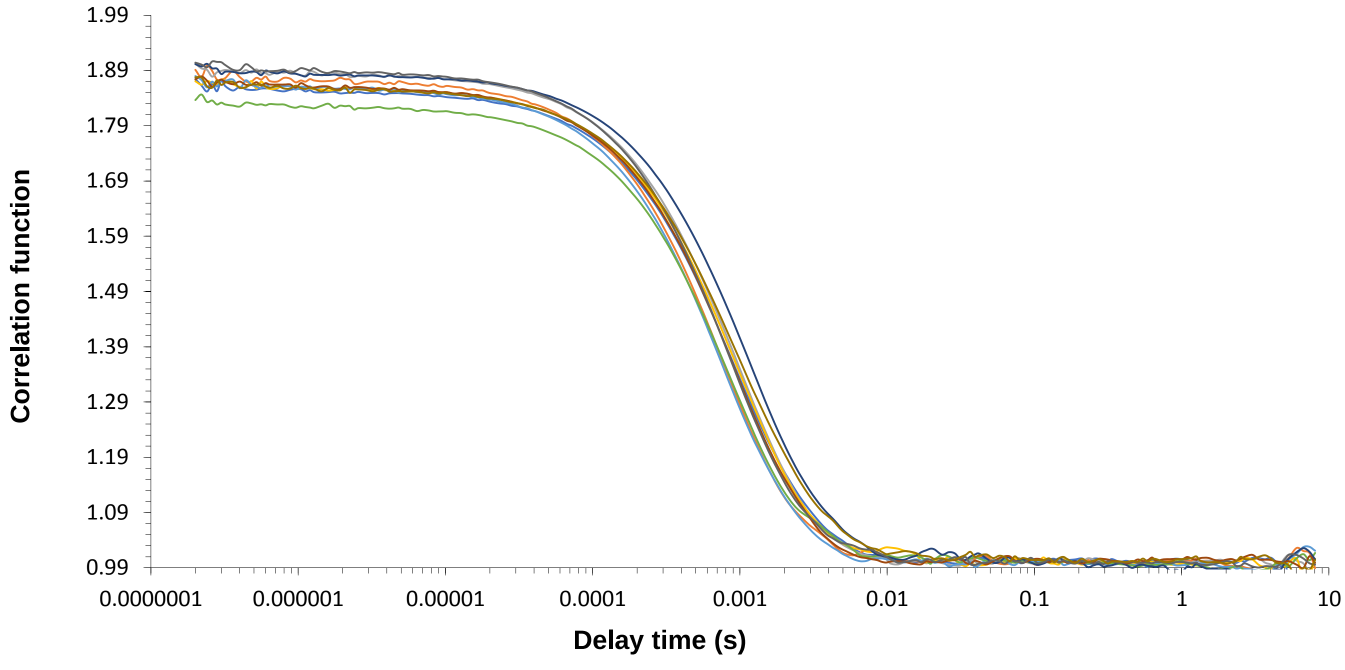

Figure S87. Correlation function data for 10 DLS runs of compounds **1** and **2** in a 1:1 mix (total concentration 0.56 mM) and a EtOH:H_2O (1:19) solution at 298 K. The correlation function data for the compound **1** and **2** 1:1 mix at 5.56mM could not be obtained due to solubility.

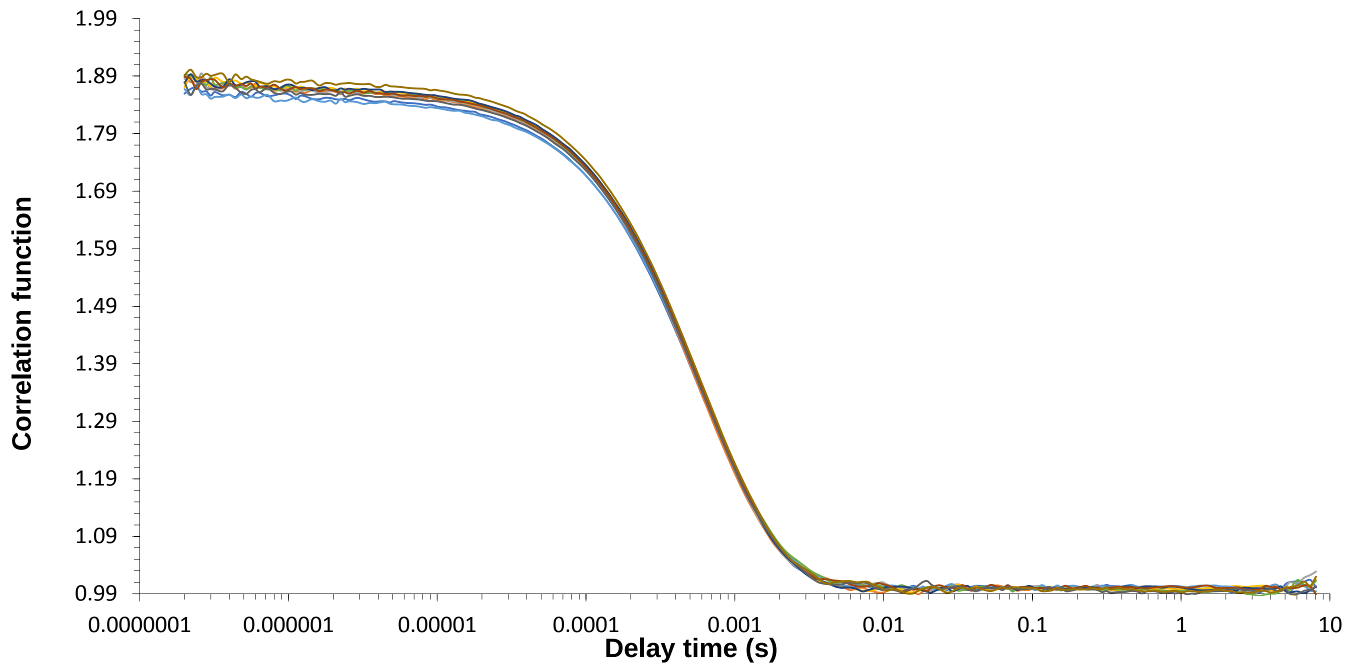

Figure S88. Correlation function data for 10 DLS runs of compounds **1** and **4** in a 1:1 mix (total concentration 5.56 mM) and a EtOH:H_2O (1:19) solution at 298 K.

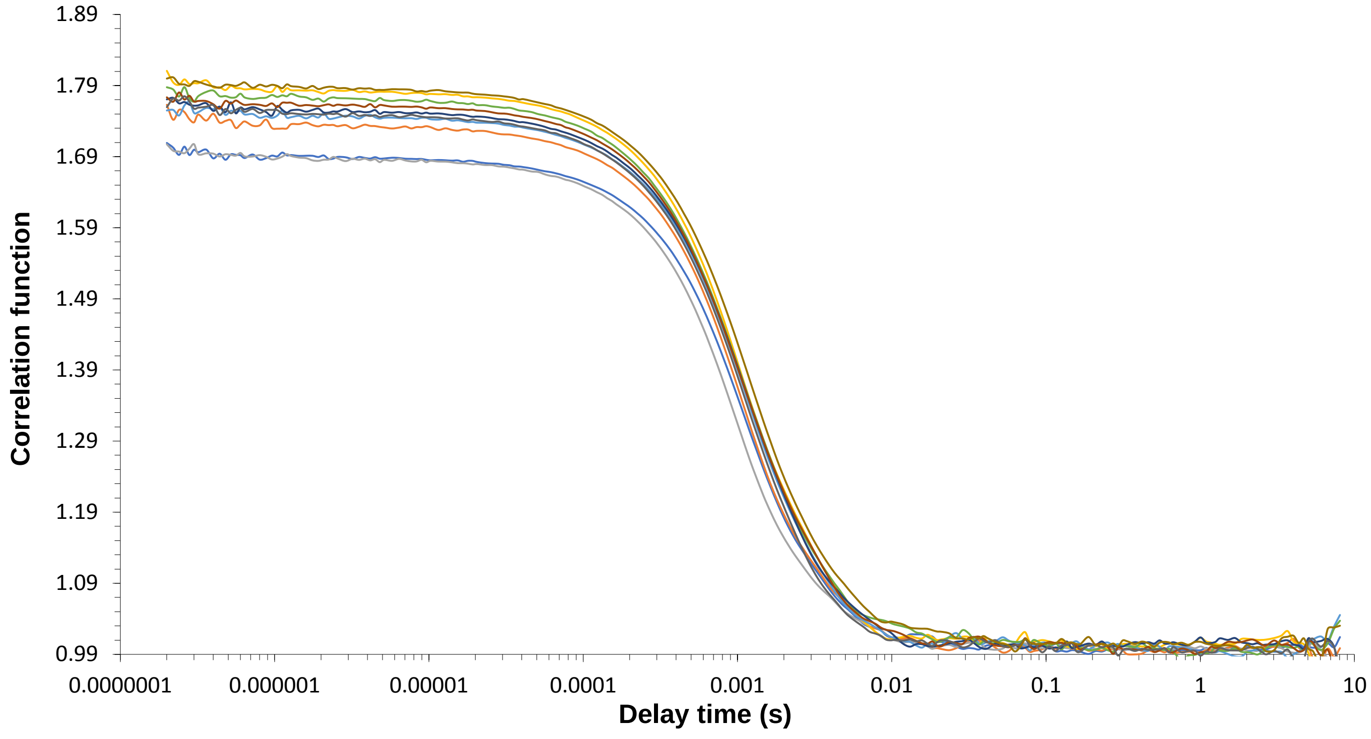

Figure S89. Correlation function data for 10 DLS runs of compounds **1** and **4** in a 1:1 mix (total concentration 0.56 mM) and a EtOH:H_2O (1:19) solution at 298 K.

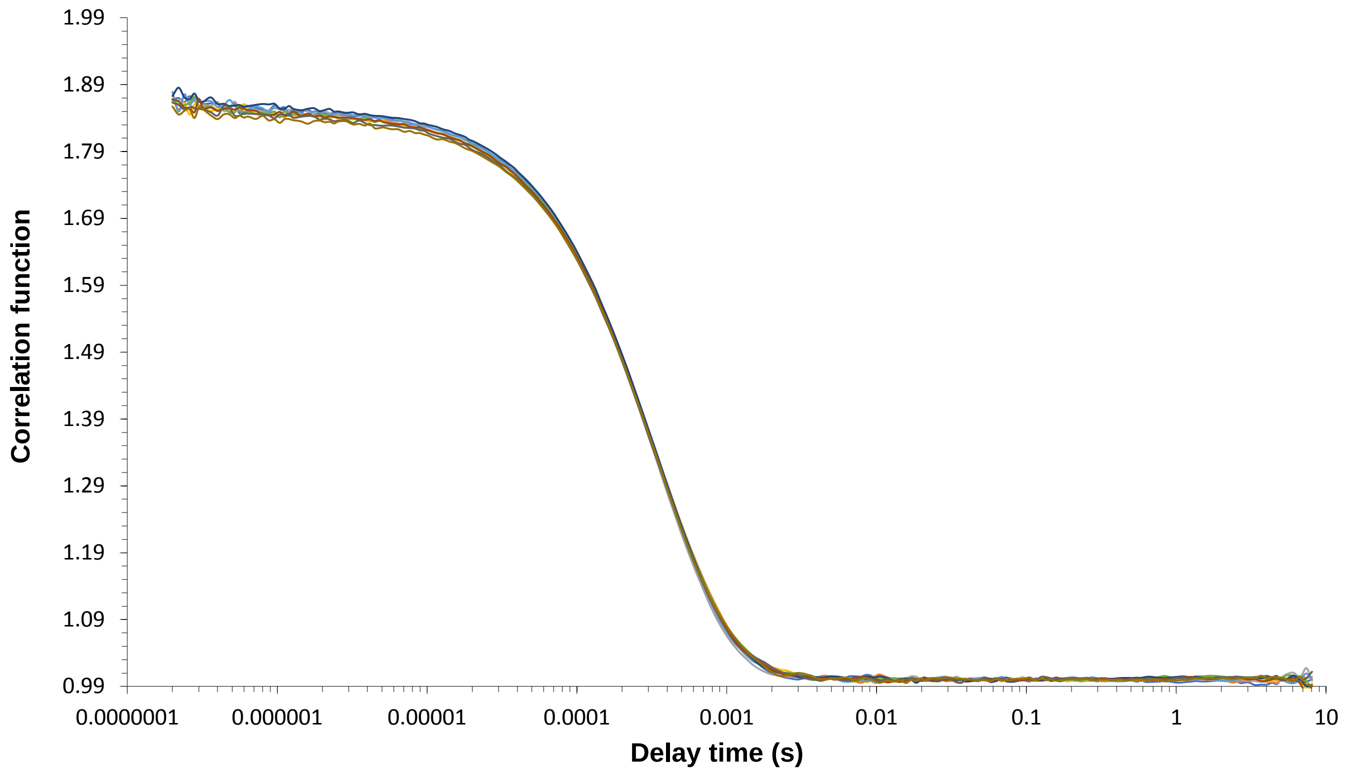

Figure S90. Correlation function data for 10 DLS runs of compounds **2** and **4** in a 1:1 mix (total concentration 5.56 mM) and a EtOH:H_2O (1:19) solution at 298 K.

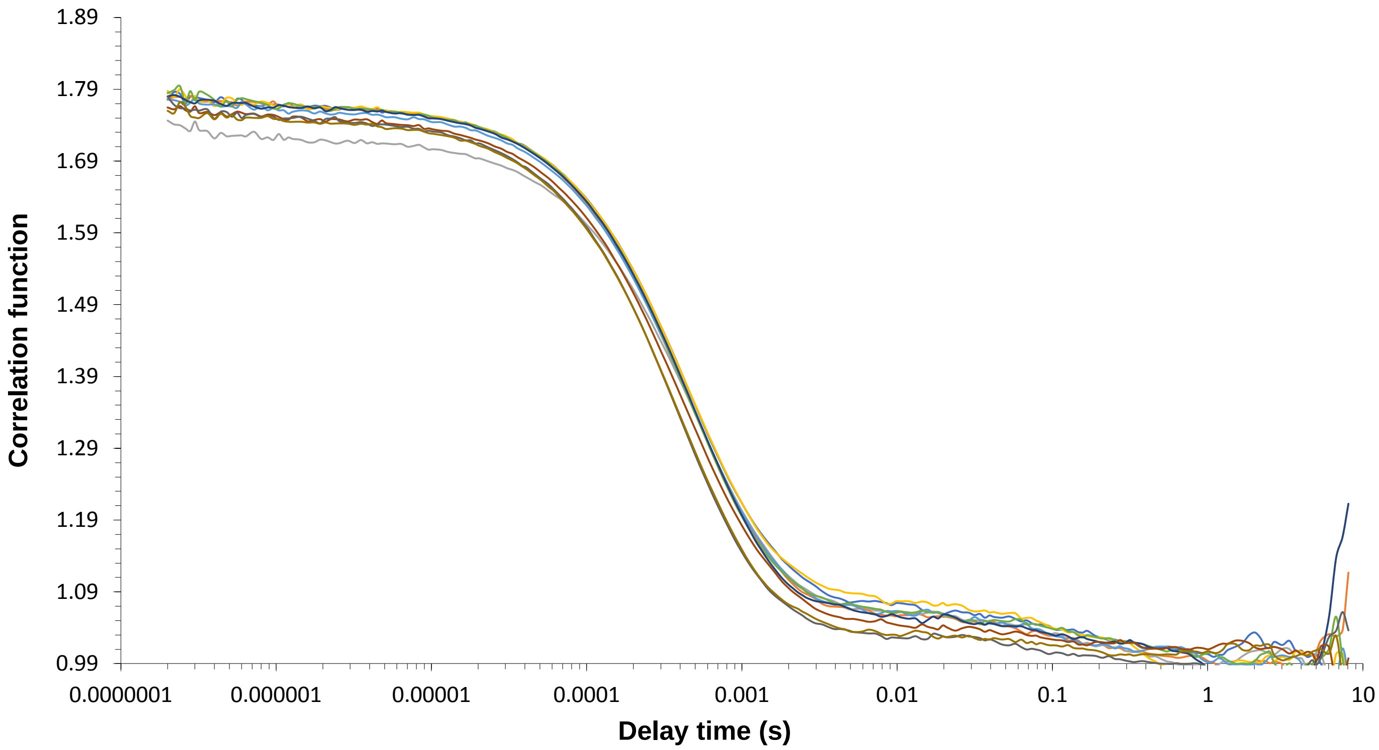

Figure S91. Correlation function data for 10 DLS runs of compounds **2** and **4** in a 1:1 mix (total concentration 0.56 mM) and a EtOH:H_2O (1:19) solution at 298 K.

Dynamic Light Scattering (DMSO)

Particle Size Distribution

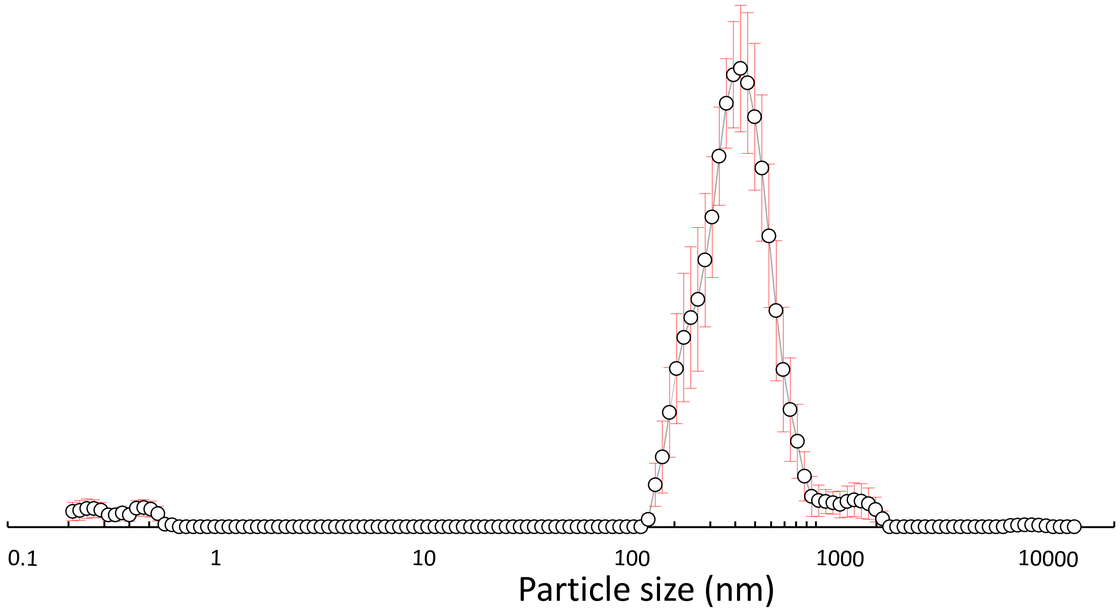

Figure S92. The average intensity particle size distribution calculated using 10 DLS runs for compound **1** (55.56 mM) in a DMSO solution at 298 K.

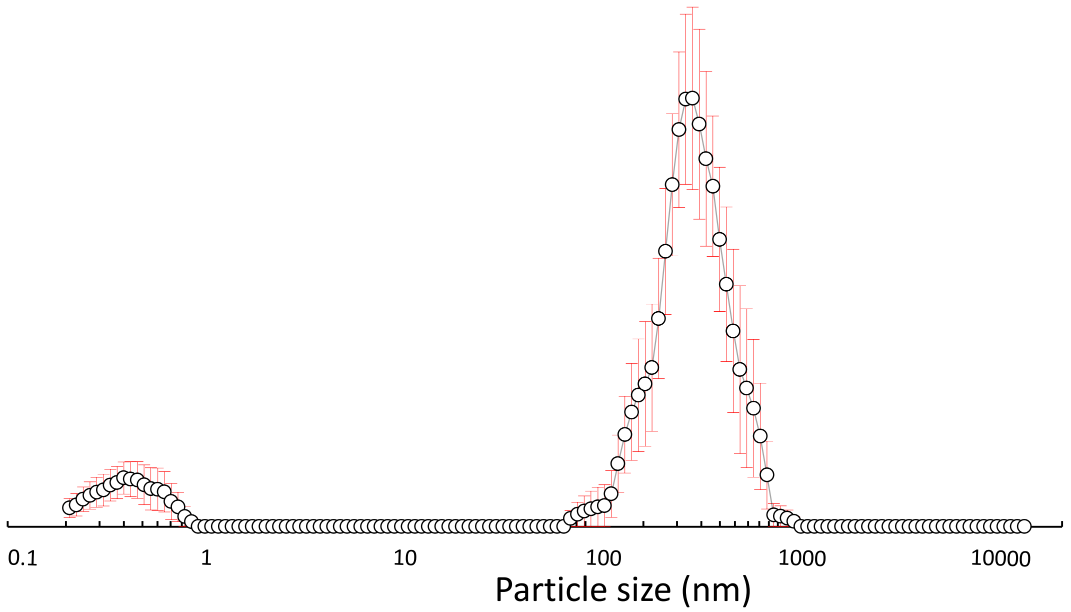

Figure S93. The average intensity particle size distribution calculated using 10 DLS runs for compound **2** (55.56 mM) in a DMSO solution at 298 K.

.

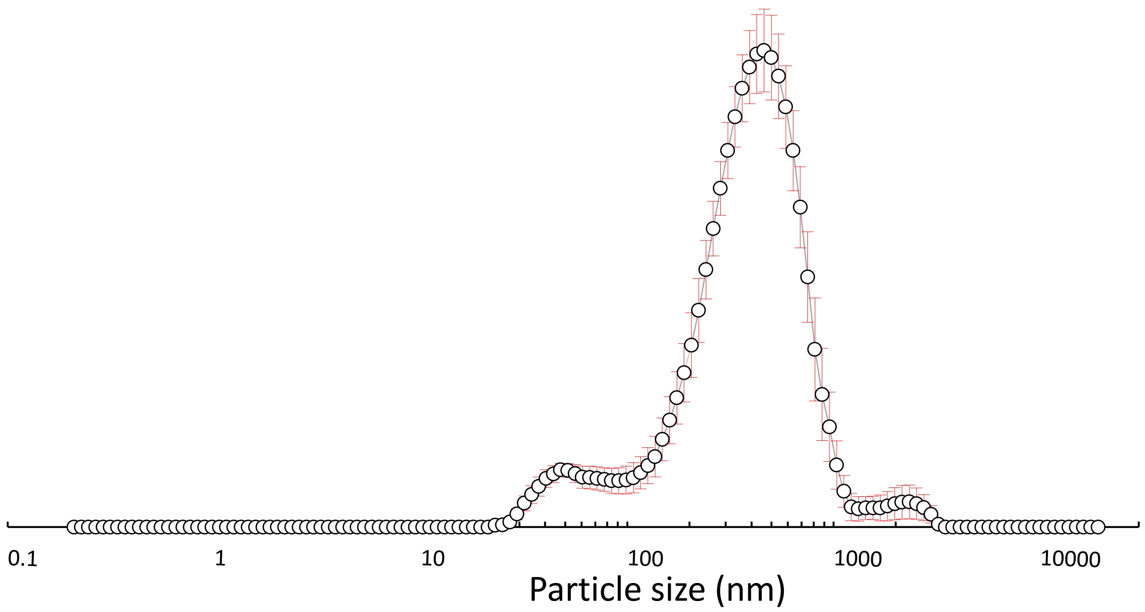

Figure S94. The average intensity particle size distribution calculated using 10 DLS runs for compound **4** (55.56 mM) in a DMSO solution at 298 K.

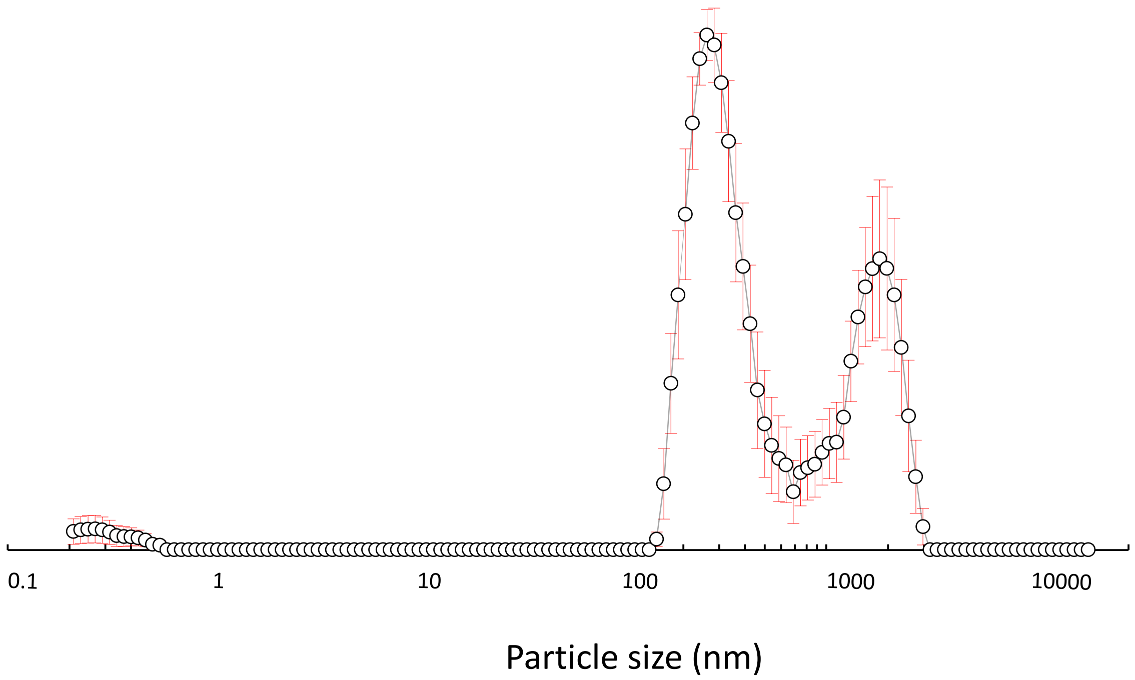

Figure S95. The average intensity particle size distribution calculated using 10 DLS runs for compounds **1** and **2** in a 1:1 mix (total concentration 55.56 mM) and a DMSO solution at 298 K.

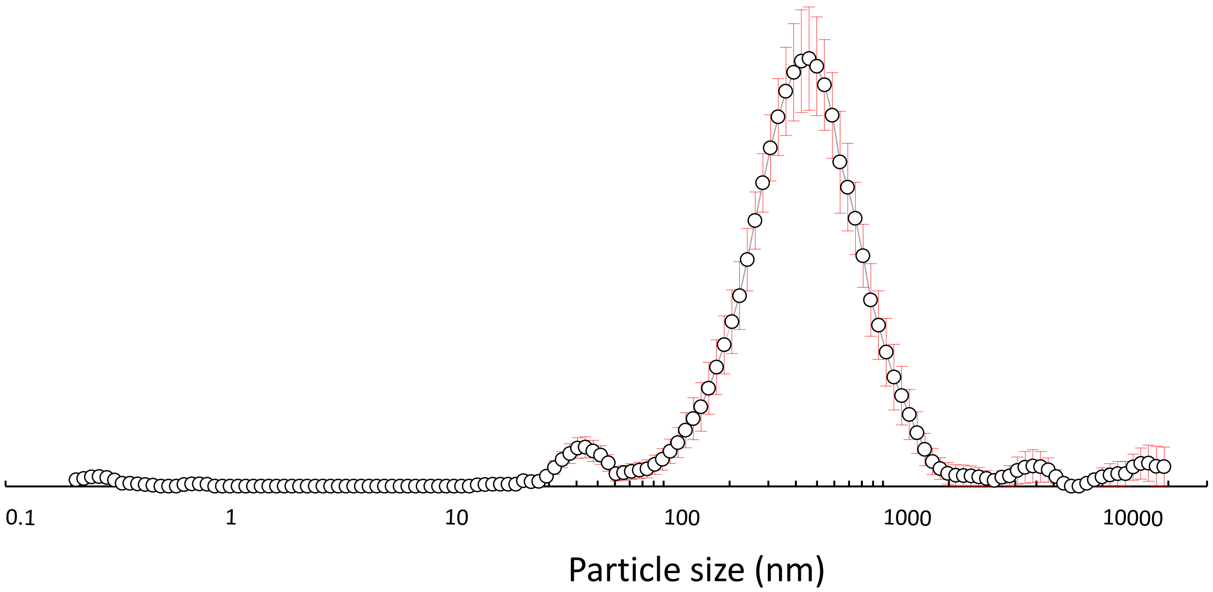

Figure S96. The average intensity particle size distribution calculated using 10 DLS runs for compounds **1** and **4** in a 1:1 mix (total concentration 55.56 mM) and a DMSO solution at 298 K.

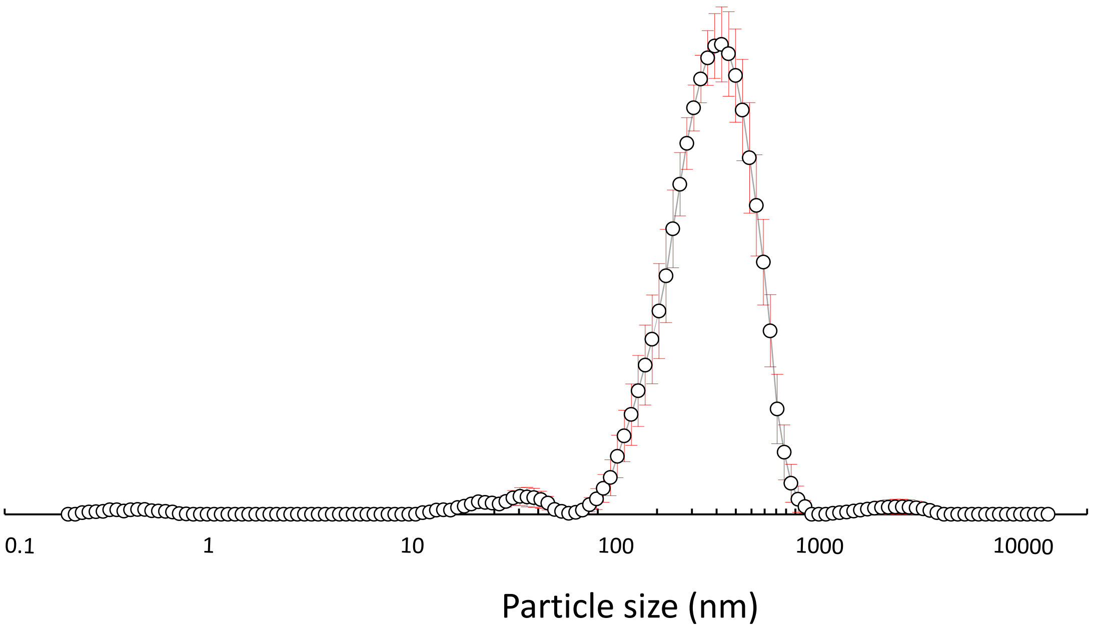

Figure S97. The average intensity particle size distribution calculated using 10 DLS runs for compounds **2** and **4** in a 1:1 mix (total concentration 55.56 mM) and a DMSO solution at 298 K.

Correlation Function Data

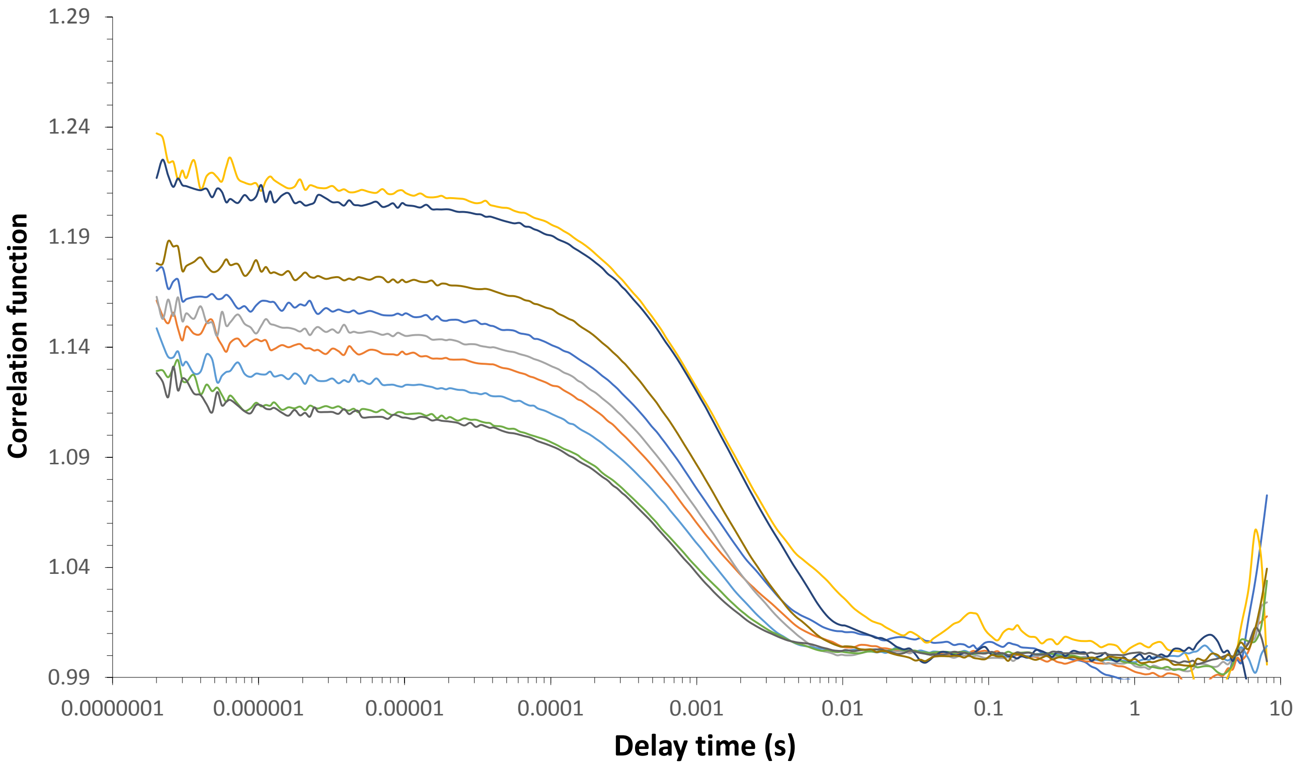

Figure S98. Correlation function data for 10 DLS runs of compound **1** (55.56 mM) in a DMSO solution at 298 K.

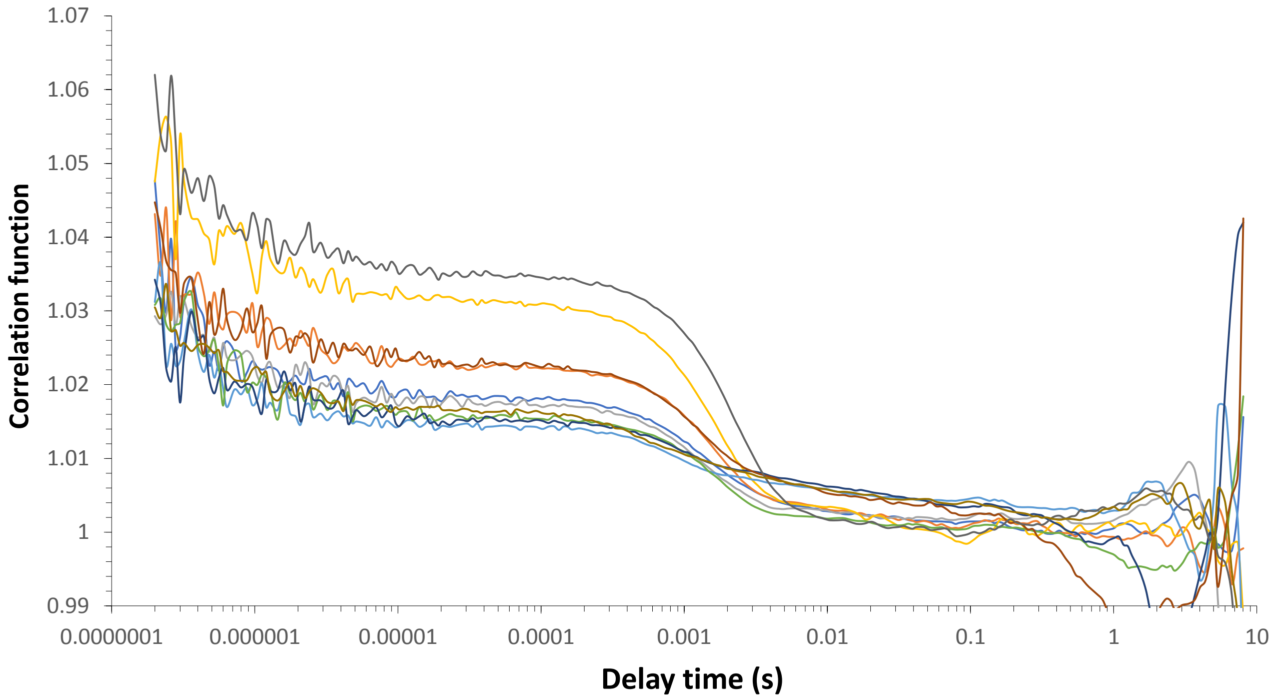

Figure S99. Correlation function data for 10 DLS runs of compound **2** (55.56 mM) in a DMSO solution at 298 K.

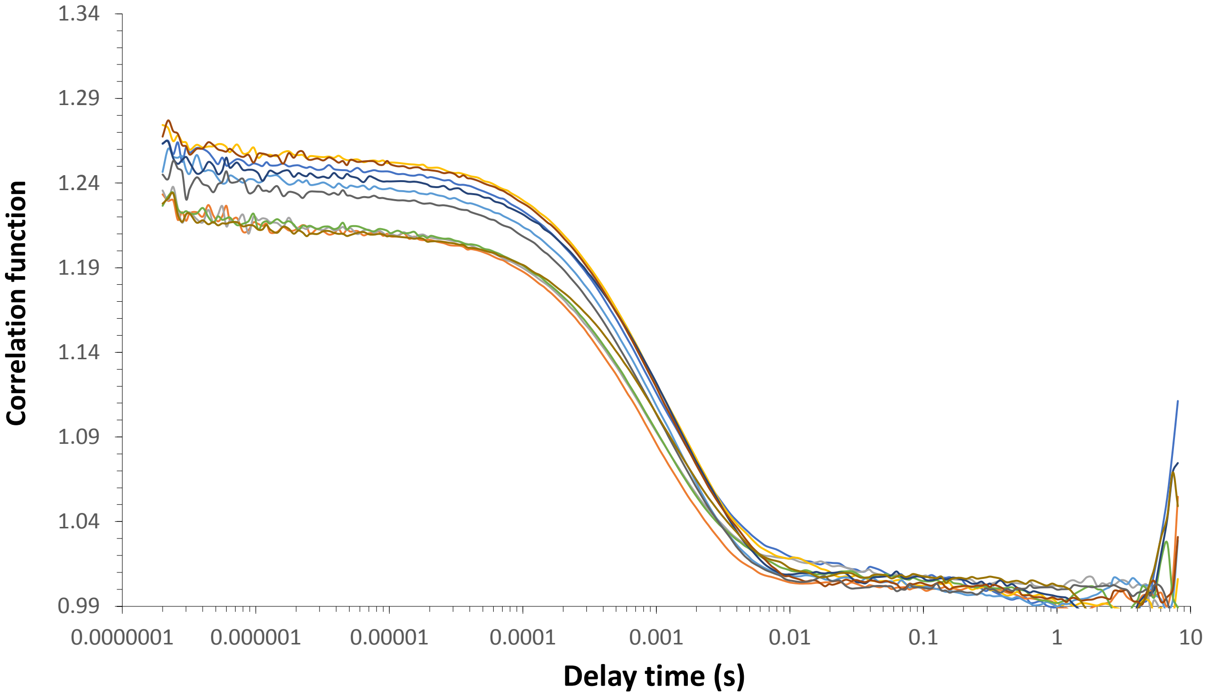

Figure S100. Correlation function data for 10 DLS runs of compound **4** (55.56 mM) in a DMSO solution at 298 K.

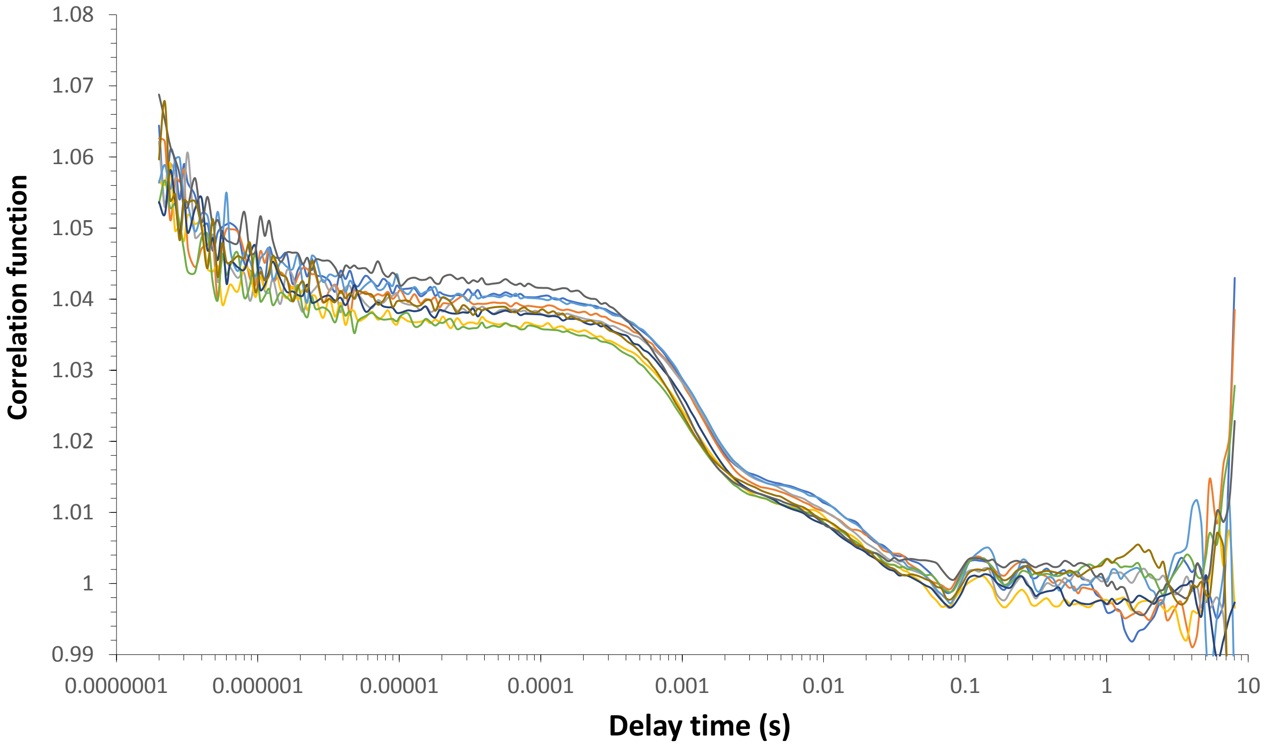

Figure S101. Correlation function data for 10 DLS runs of compounds **1** and **2** in a 1:1 mix (total concentration 55.56 mM) and a DMSO solution at 298 K.

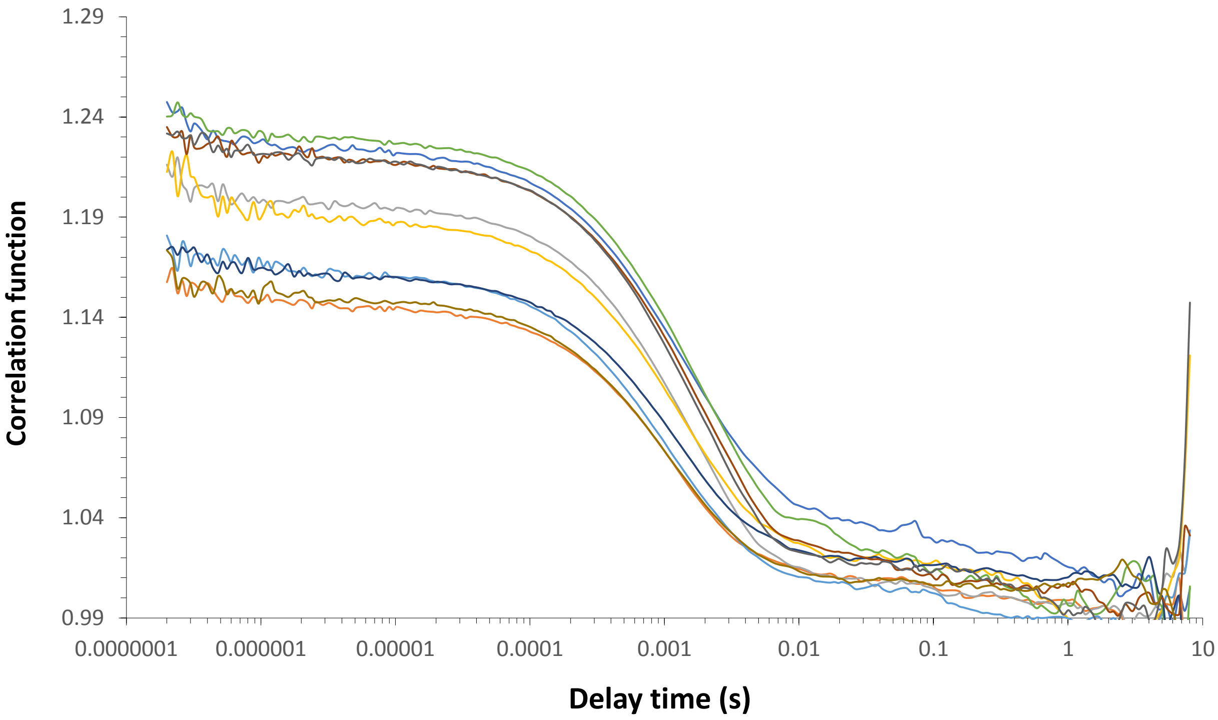

Figure S102. Correlation function data for 10 DLS runs of compounds **1** and **4** in a 1:1 mix (total concentration 55.56 mM) and a DMSO solution at 298 K.

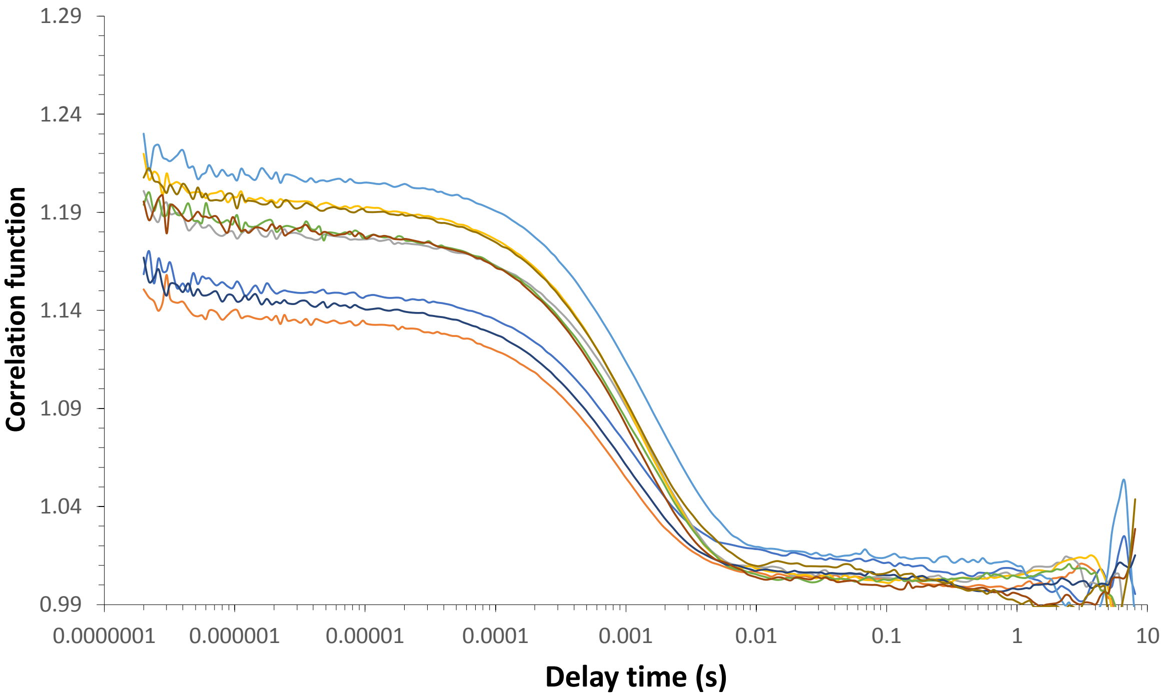

Figure S103. Correlation function data for 10 DLS runs of compounds **2** and **4** in a 1:1 mix (total concentration 55.56 mM) and a DMSO solution at 298 K.

Dynamic Light Scattering Data Overview

Table S5. Summary of average intensity particle size distribution data from Figures S70-S80, S92-S97 for solutions of **1**, **2**, **4** and associated 1:1 mixtures. Error = standard error of the mean.

Compound	DMSO			H_2O:EtOH 19:1		
	Concentration (mM)	Peak maxima (nm)	Polydispersity (%)	Concentration (mM)	Peak maxima (nm)	Polydispersity (%)
**1**	55.56[a]	421 (± 32.3467)	29 (± 3.2331)	5.56	142 (± 1.4874)	27 (± 0.6656)
				0.56	124 (± 1.5359)	18 (± 1.2114)
**2**	55.56[a]	378 (± 38.7352)	45 (± 4.5526)	5.56	209 (± 2.8953)	22 (± 0.4392)
				0.56	198 (± 3.8297)	16 (± 2.5222)
**4**	55.56	436 (± 23.1479)	27 (± 0.9065)	5.56	217 (± 2.7448)	23 (± 0.3937)
				0.56	324 (± 14.2343)	27 (± 0.5010)
**1** + **2**	55.56[b, d]	651 (± 223.0165)	199 (± 31.2061)	5.56	[c]	[c]
				0.56	224 (± 7.8719)	26 (± 1.1607)
**1** + **4**	55.56[d]	496 (± 21.3874)	26 (± 1.0594)	5.56	288 (± 4.6891)	24 (± 0.5152)
				0.56	275 (± 7.4803)	25 (± 0.9882)
**2** + **4**	55.56[d]	404 (± 23.0437)	27 (± 0.7172)	5.56	155 (± 2.1293)	22 (± 0.7278)
				0.56	193 (± 2.7340)	27 (± 0.6518)

[a] Reproducible correlation functions could not be obtained. [b] Data quality was not sufficient to enable accurate data analysis, which led to high errors and untrustworthy calculated values. [c] Compound solubility prevented accurate data acquisition during the experiment. [d] Total molar concentration.

Zeta Potential Data

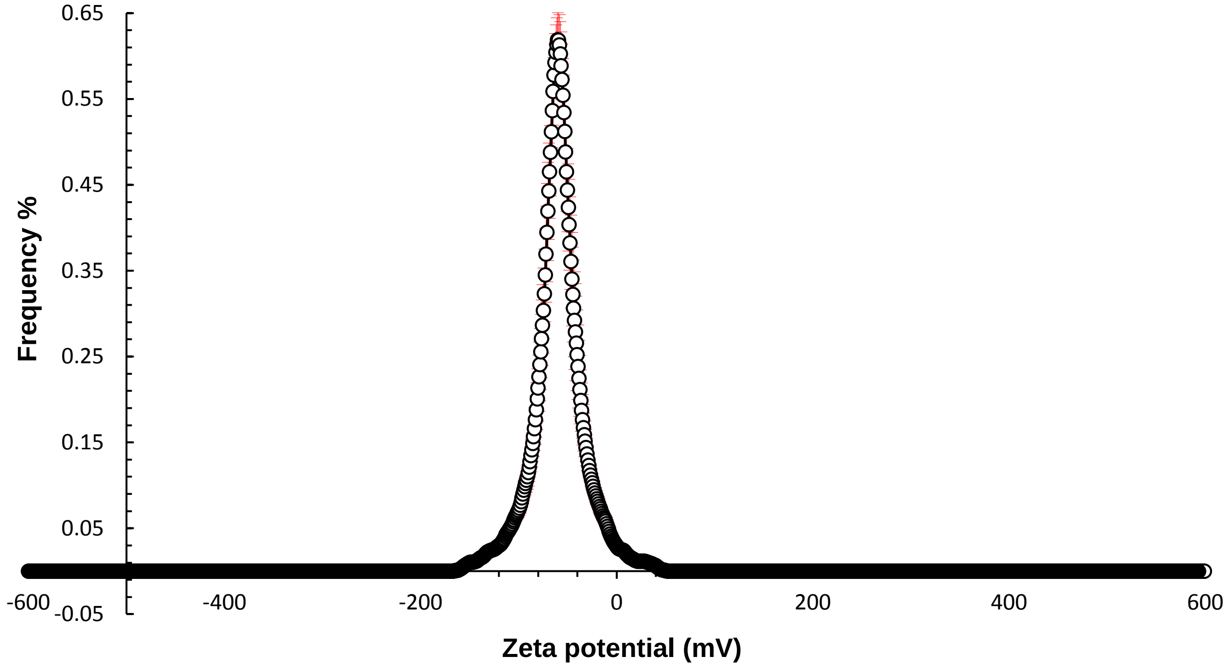

Figure S104. The average zeta potential distribution calculated using 10 runs for compound **1** (5.56 mM) in an EtOH:H_2O (1:19) solution at 298 K.

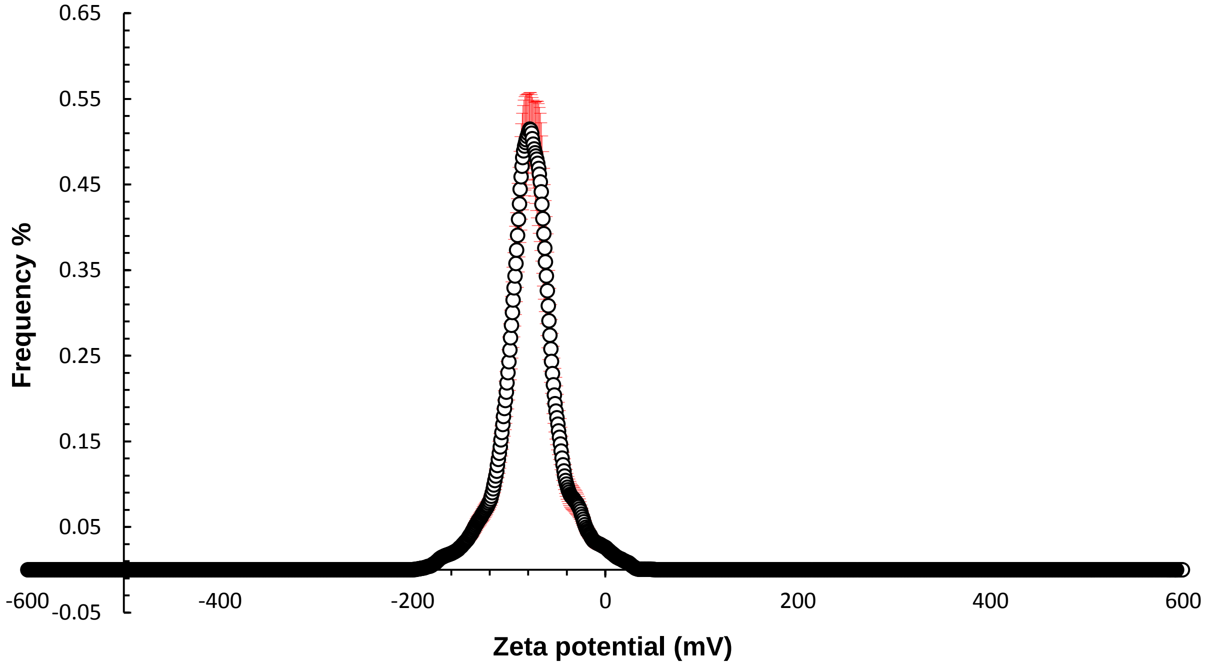

Figure S105. The average zeta potential distribution calculated using 10 runs for compound **1** (0.56 mM) in an EtOH:H_2O (1:19) solution at 298 K.

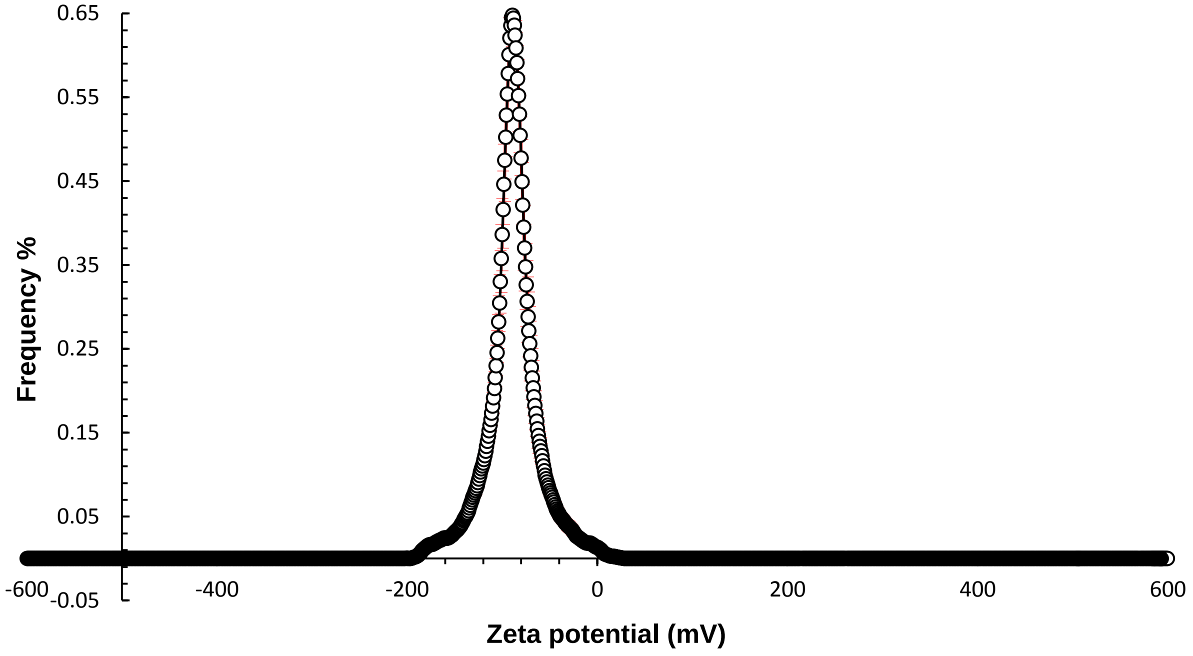

Figure S106. The average zeta potential distribution calculated using 10 runs for compound **2** (5.56 mM) in an EtOH:H_2O (1:19) solution at 298 K.

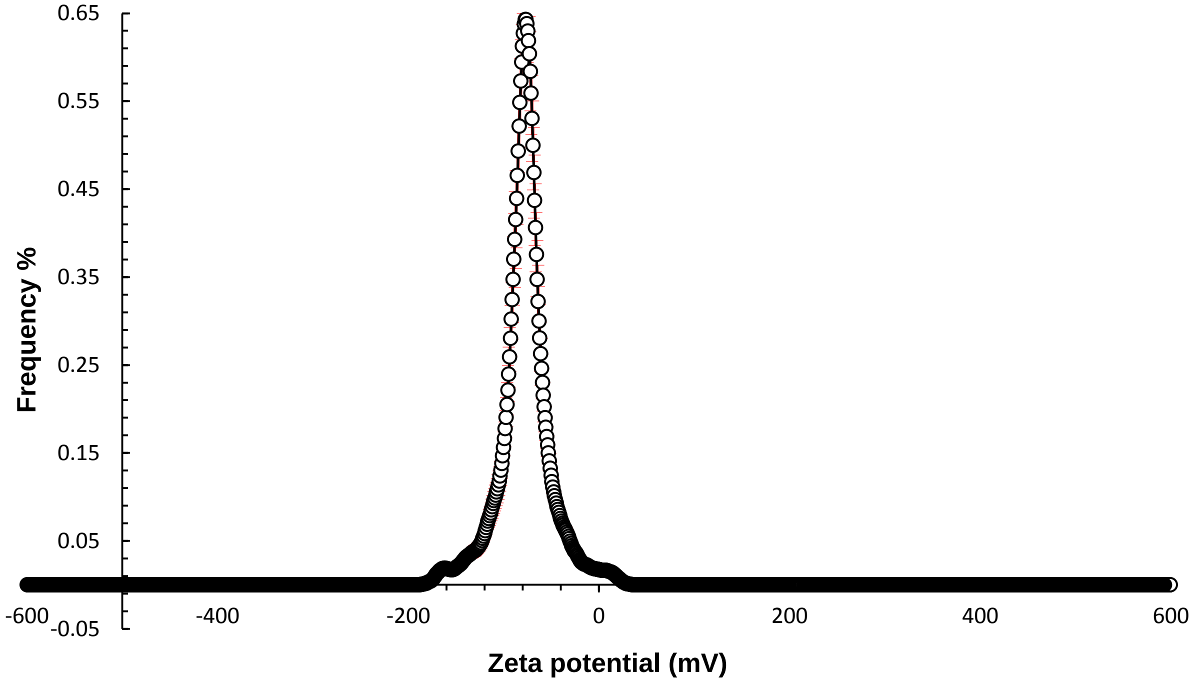

Figure S107. The average zeta potential distribution calculated using 10 runs for compound **2** (0.56 mM) in an EtOH:H_2O (1:19) solution at 298 K.

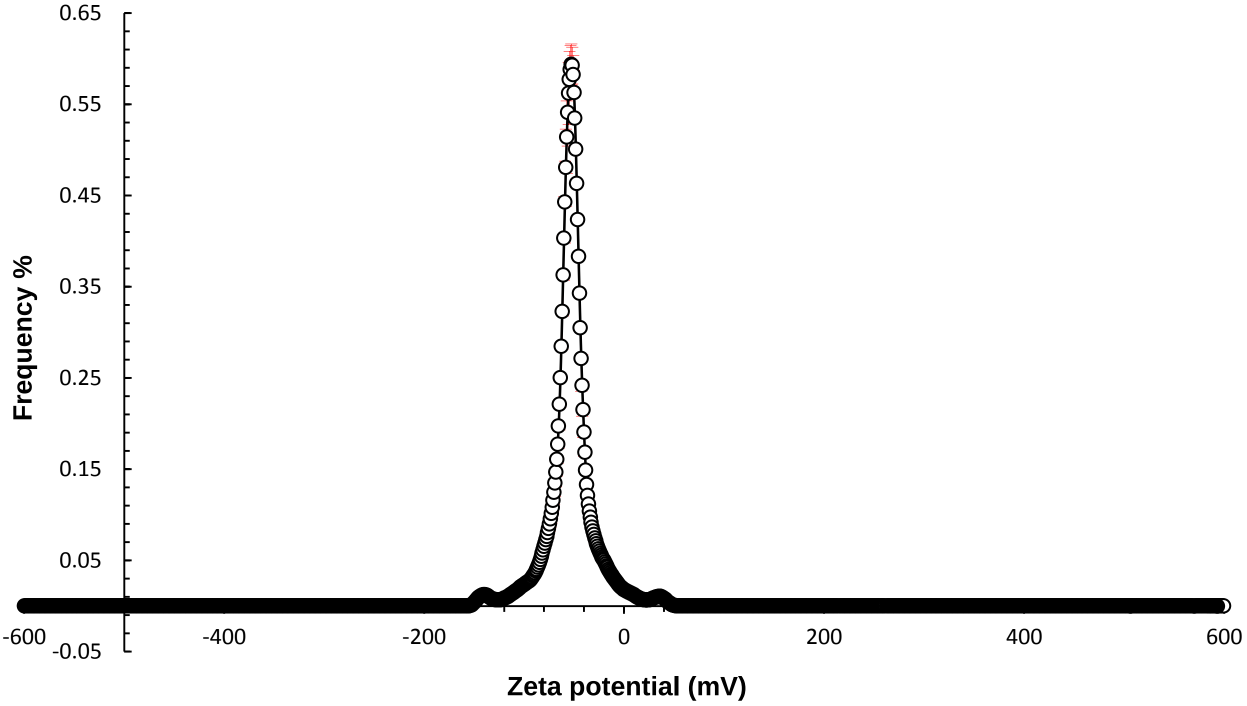

Figure S108. The average zeta potential distribution calculated using DLS runs for compound **4** (5.56 mM) in an EtOH:H_2O (1:19) solution at 298 K.

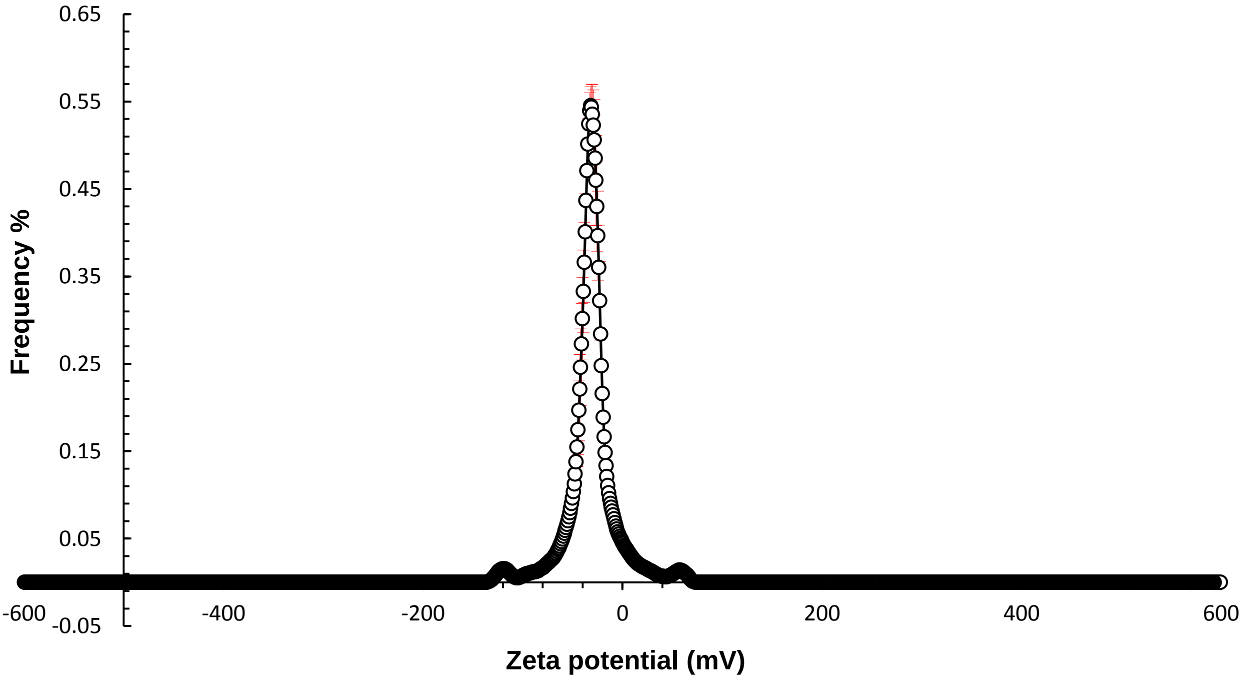

Figure S109. The average zeta potential distribution calculated using DLS runs for compound **4** (0.56 mM) in an EtOH:H_2O (1:19) solution at 298 K.

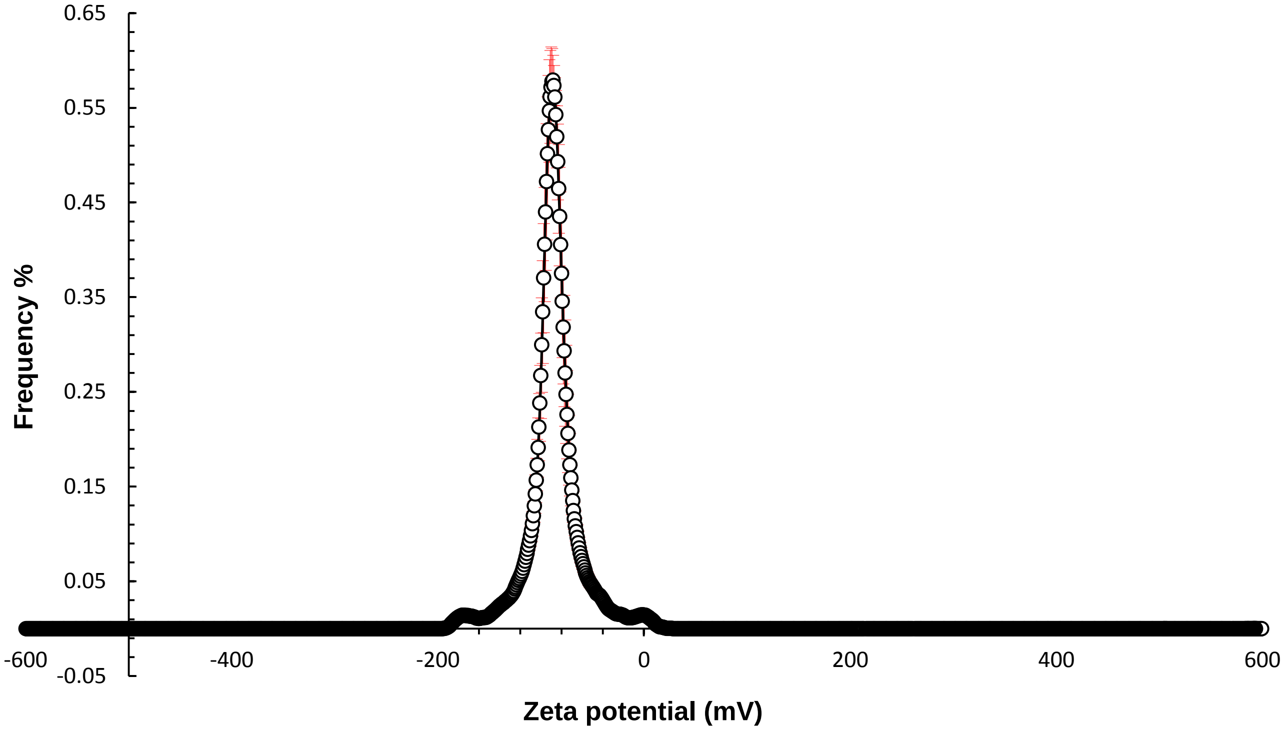

Figure S110. The average zeta potential distribution calculated using DLS runs for compounds **1** and **2** in a 1:1 mixture (total concentration 0.56 mM) and a EtOH:H_2O (1:19) solution at 298 K. An analogous experiment could not be run at the comparative 5.56 mM concentration due to compound solubility.

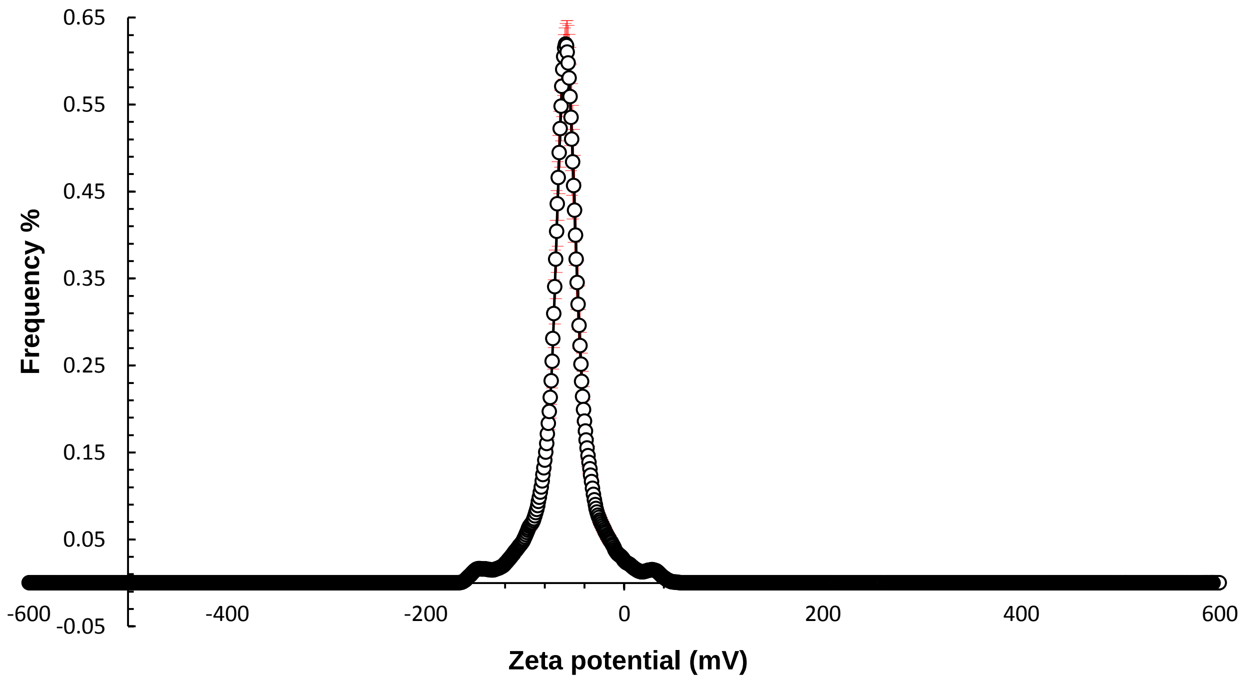

Figure S111. The average zeta potential distribution calculated using 10 runs for compounds **1** and **4** in a 1:1 mixture (total concentration 5.56 mM) and a EtOH:H_2O (1:19) solution at 298 K.

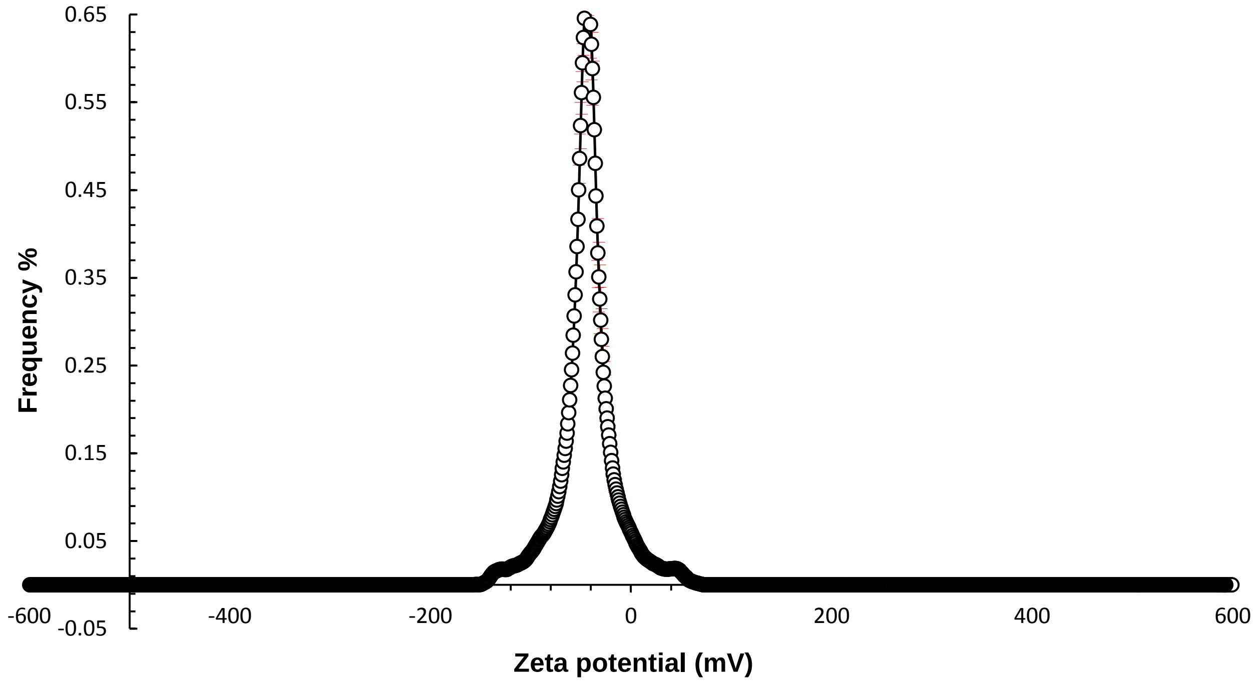

Figure S112. The average zeta potential distribution calculated using 10 runs for compounds **1** and **4** in a 1:1 mixture (total concentration 0.56 mM) and a EtOH:H_2O (1:19) solution at 298 K.

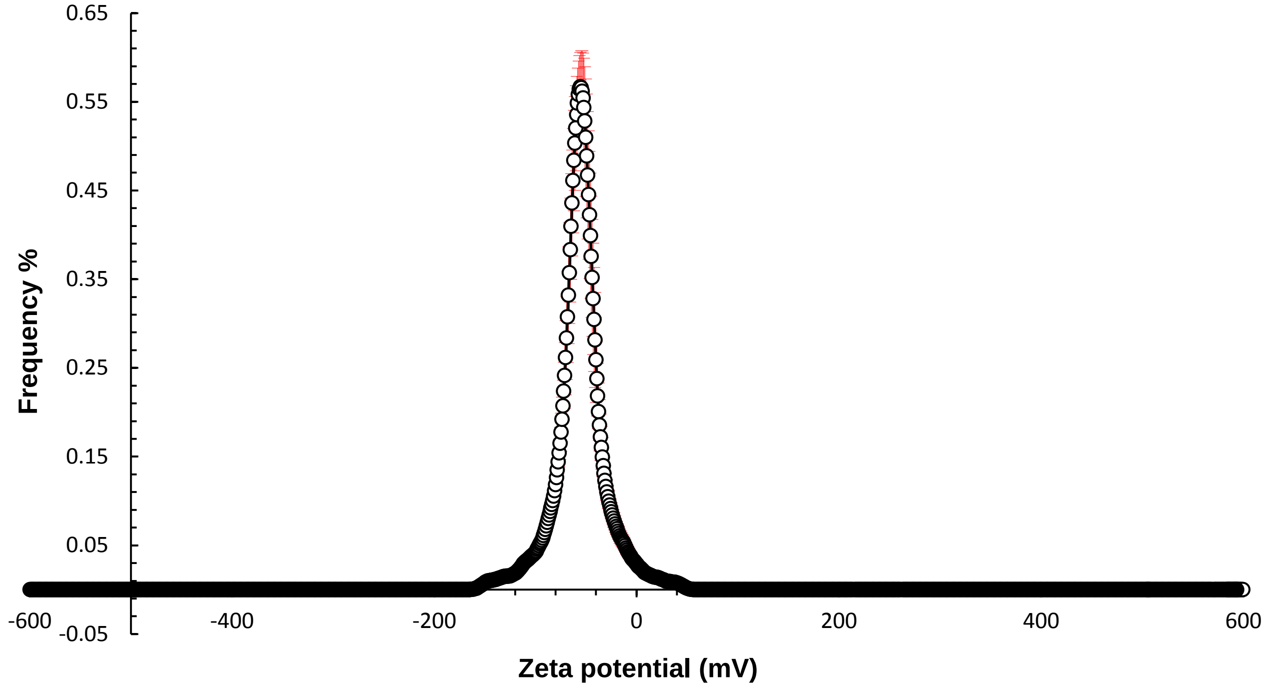

Figure S113. The average zeta potential distribution calculated using 10 runs for compounds **2** and **4** in a 1:1 mixture (total concentration 5.56 mM) and a EtOH:H_2O (1:19) solution at 298 K.

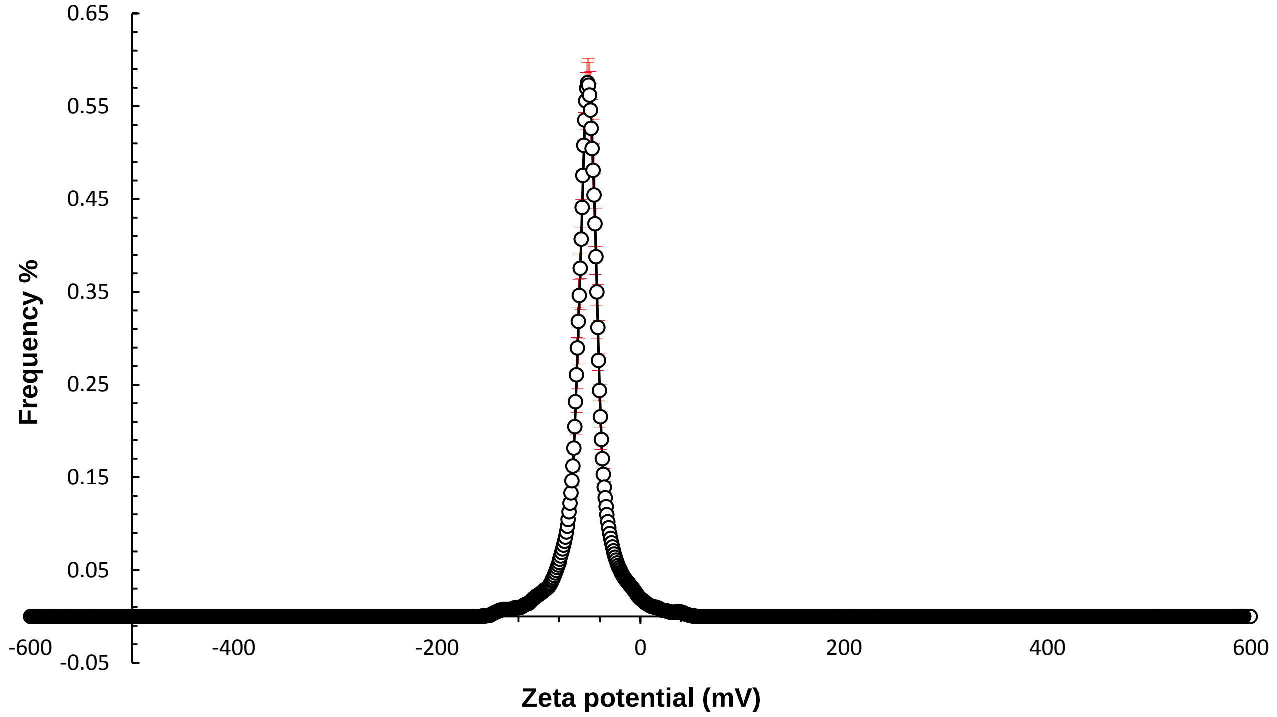

Figure S114. The average zeta potential distribution calculated using 10 runs for compounds **2** and **4** in a 1:1 mixture (total concentration 0.56 mM) and a EtOH:H_2O (1:19) solution at 298 K.

Table S6. Summary of zeta potential, CMC and surface tension at CMC data obtained for compounds **1**, **2**, **4** and mixtures of compound **1** and **2**, **1** and **4**, **2** and **4** in an EtOH:H_2O (1:19) solution.

Compound	Zeta potential (mV)		CMC (mM)	Surface tension at CMC (mN/m)
	5.56 mM	0.56 mM		
**1**	- 64	-78	8.17	52.75
**2**	- 88	-78	2.52[6]	43.15[6]
**4**	- 57	-38	2.75	52.65
**1** + **2**	[a]	-83	1.09	52.49
**1** + **4**	- 65	-48	5.12	51.60
**2** + **4**	- 60	-41	2.48	45.74

[a] Compound solubility prevented experiment.

Single Crystal X-Ray Diffraction Structures

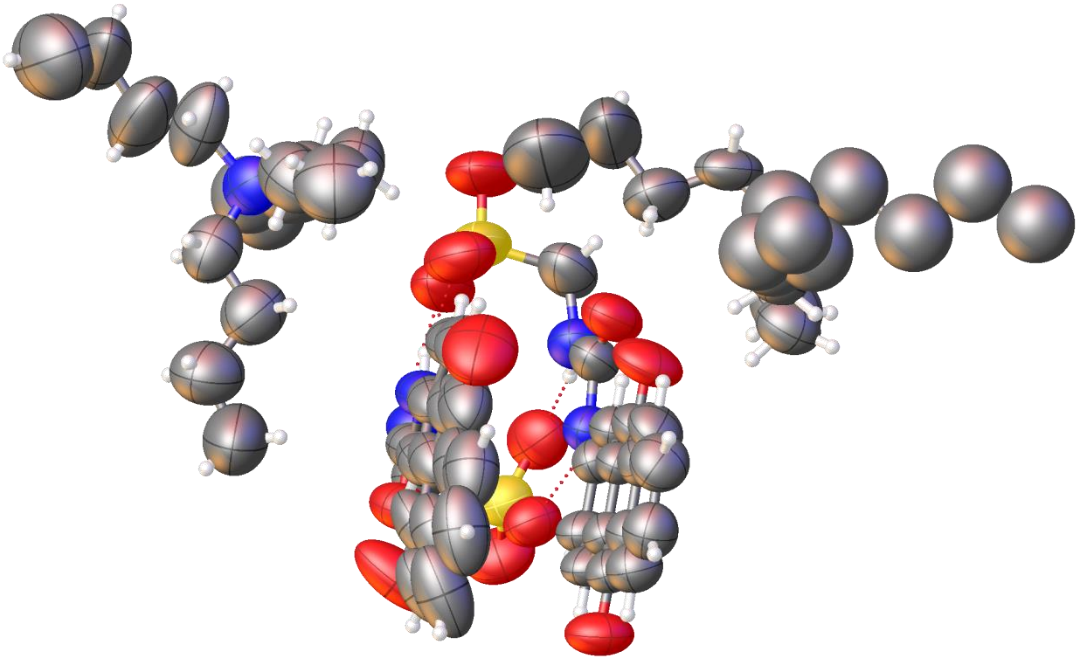

Figure S115. Crystal data for compound **1**: red = oxygen; yellow = sulfur; blue = nitrogen; white = hydrogen; grey = carbon. CCDC 1866274, $C_{64}H_{64}$ $N_6O_{12}S_2$ (M =1173.33): triclinic, space group P -1, a = 11.9182(9) Å, b = 12.8979(8) Å, c = 23.0180(13) Å, α = 84.931(5)°, β = 84.247(6)°, γ = 75.288(6)°, V = 3397.8(4) $Å^3$, Z = 2, T = 293(1) K, CuK\α = 1.5418 Å, Dcalc = 1.147 g/cm^3, 23306 reflections measured (7.102 ≤ 2Θ ≤ 133.200), 12023 unique (R_{int} = 0.0256, R_{sigma} = 0.0346) which were used in all calculations. The final R_1 was 0.1495 (I > 2σ(I)) and wR_2 was 0.4690 (all data). Disorder within the tetrabutylammonium counter cations has meant that the carbons atom from three of the eight butyl arms have been modelled isotopically, without the presence of the associated hydrogen atoms. This provides the most accurate model based on the limitations placed on these data by those single crystal samples obtained.

Table S7. Hydrogen bond distances and angles observed for hydrogen bonded complex formation, calculated from single crystal X-ray structure, Figure S115.

Compound	Hydrogen bond donor	Hydrogen atom	Hydrogen bond length (D•••A) (Å)	Hydrogen bond angle (D-H•••A) (°)
**2**	N1	H1	O7	2.957(10)
**2**	N2	H2	O9	2.862(7)
**2**	N3	H3	O2	2.893(8)
**2**	N4	H4	O3	2.868(9)

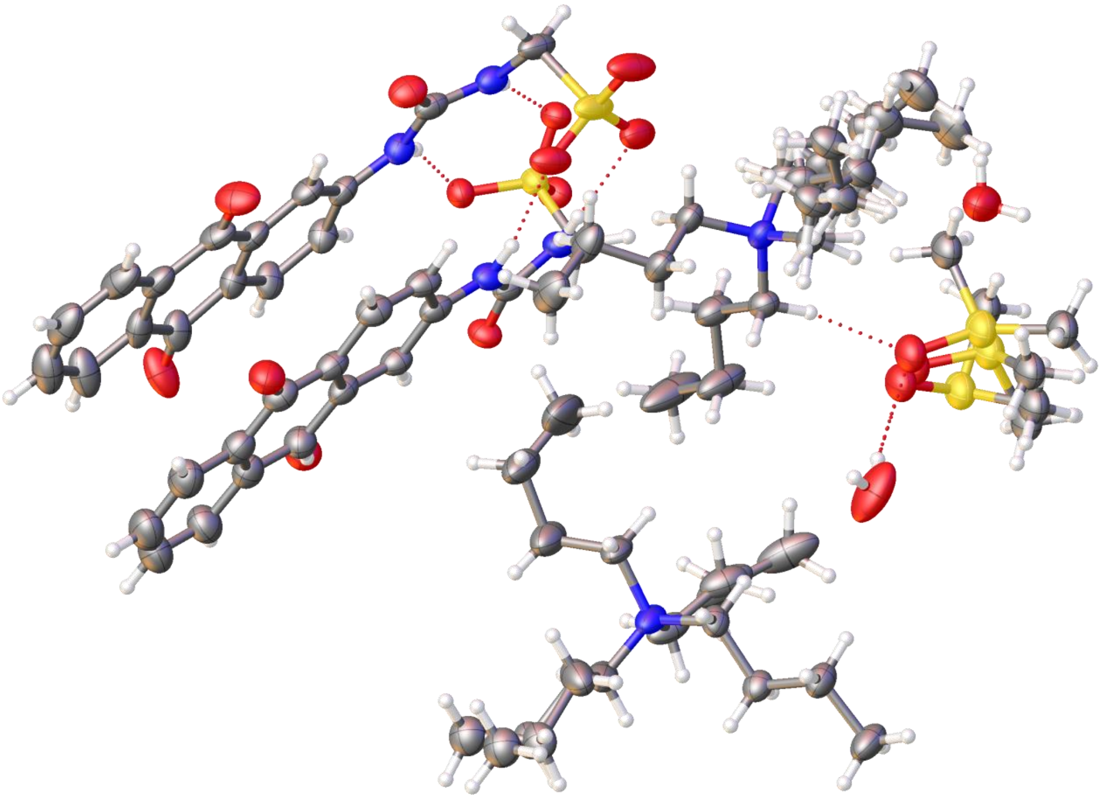

Figure S116. Crystal data for compounds **1** and **2**: red = oxygen; yellow = sulfur; blue = nitrogen; white = hydrogen; grey = carbon. CCDC 1866275, $C_{66}H_{105.09}N_2O_{13.31}S_3$ (M =1395.66): triclinic, space group P -1, a = 11.4872(10) Å, b = 12.4679(2) Å, c = 25.0105(13) Å, α = 95.236(5)°, β = 90.694(6)°, γ = 105.370(7)°, V = 3437.1(4) $Å^3$, Z = 2, T = 100(1) K, CuK\α = 1.5418 Å, Dcalc = 1.248 g/cm^3, 22986 reflections measured (7.388 ≤ 2Θ ≤ 133.192), 12134 unique (R_{int} = 0.0784, R_{sigma} = 0.0808) which were used in all calculations. The final R_1 was 0.0981 (I > 2σ(I)) and wR_2 was 0.2846 (all data). This hydrated dimer includes both the 1:1 dimer containing compounds **1** and **2** (85 % occupancy) and a dimer containing compound **2** only (15 % occupancy). This crystal was obtained from a 1:1 solution of compounds **1** and **2** in DMSO-d_6. The remaining bulk of this sample was analysed though powder diffraction techniques (Figure S117).

Table S8. Hydrogen bond distances and angles observed for hydrogen bonded complex formation, calculated from single crystal X-ray structure, Figure S116.

Compound	Hydrogen bond donor	Hydrogen atom	Hydrogen bond length (D•••A) (Å)	Hydrogen bond angle (D-H•••A) (°)
**1** and **2**	N00G	H00G	O004	2.863(4)
**1** and **2**	N9	H9	O003	2.873(4)
**1** and **2**	N10	H10	O00D	3.003(4)
**1** and **2**	N11	H11	O1AA	2.868(9)

Powder X-Ray Diffraction Study Data

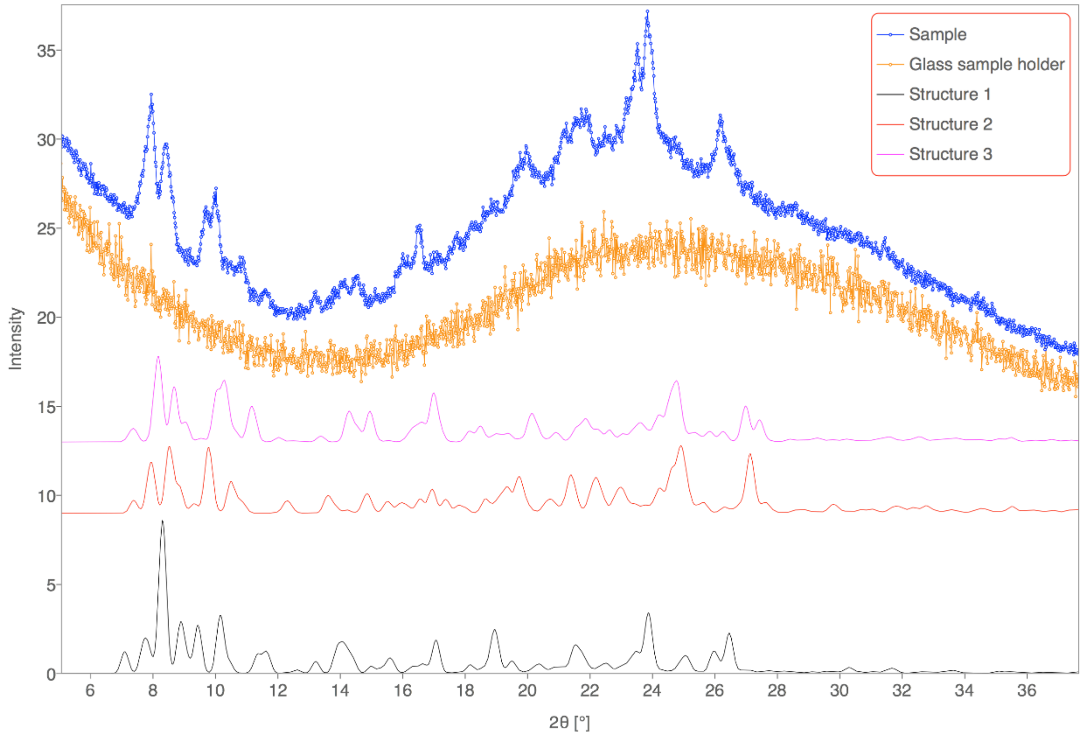

Figure S117. Comparison of the observed powder X-ray diffraction pattern (in blue) compared to that of the glass plate used as a sample holder (orange), and those calculated for Structures **1** (CCDC 1866274, Figure S115), **2** (CCDC 1866275, Figure S116) and **3** (CCDC 1562758[6]) in black, red and magenta, respectively.

Microscopy

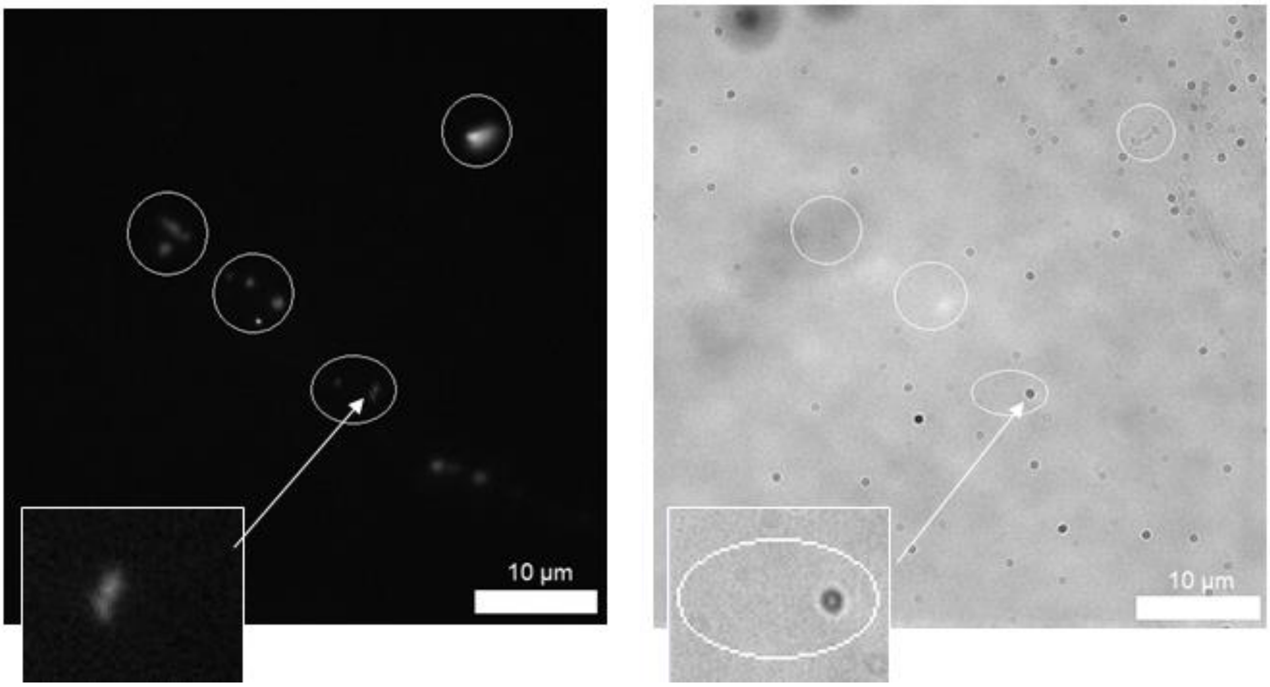

Figure S118. Left: a DAPI filtered fluorescence microscopy image of compound **1** (1mM) in an EtOH: H_2O (1: 19) solution. Right: an analogous transmitted light microscopy image. Evidence of aggregated spherical structures are circled for clarity. Photo bleaching during the imaging process resulted in loss of fluorescence emission intensity, as a result some aggregates could not be captured in the fluorescence microscopy image. Shape variation can be due to individual aggregation and or moving structures.

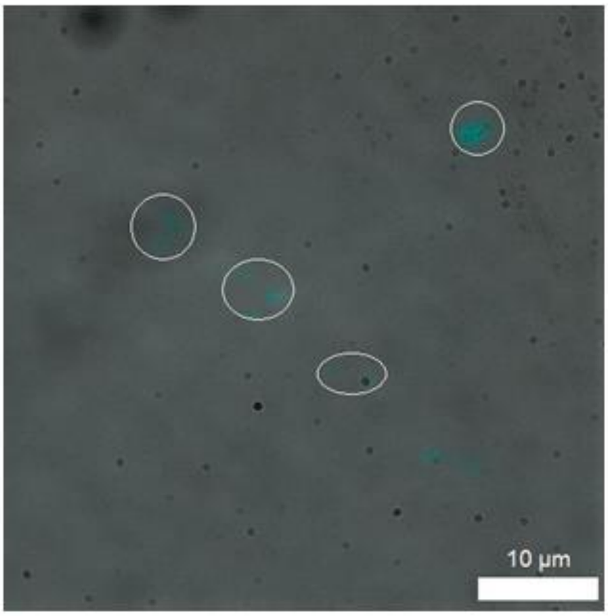

Figure S119. An overlaid image of those shown in Figure S118 (left and right) image. Clear evidence of aggregated spherical structures of compound **1** are circled for clarity.

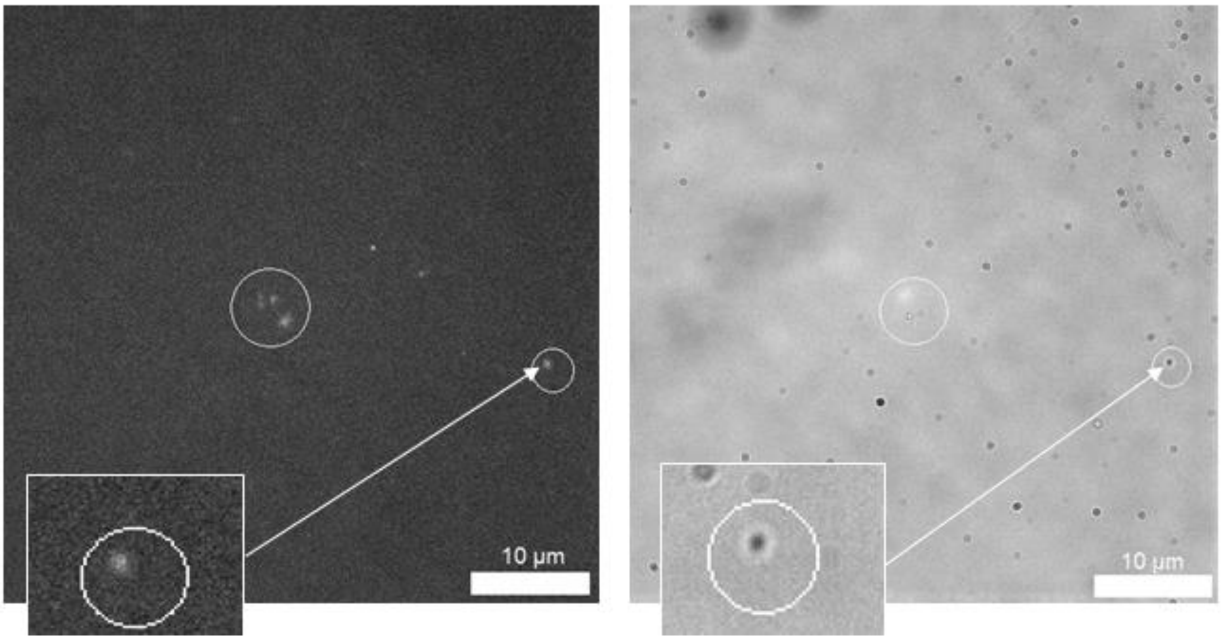

Figure S120. Left: a DAPI filtered fluorescence microscopy image of compound **1** (1mM) in an EtOH: H_2O (1: 19) solution. Right: an analogous transmitted light microscopy image. Evidence of aggregated spherical structures are circled for clarity. Photo bleaching during the imaging process resulted in loss of fluorescence emission intensity, as a result some aggregates could not be captured in the fluorescence microscopy image. Shape variation can be due to individual aggregation and or moving structures.

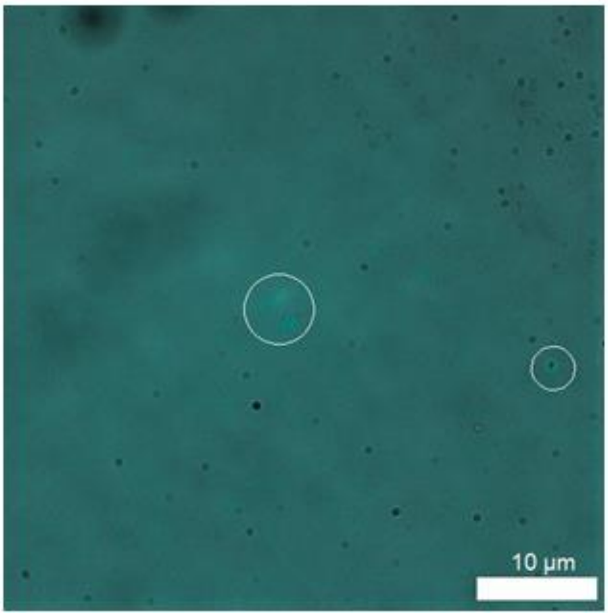

Figure S121. An overlaid image of those shown in Figure S120 (left and right) image. Clear evidence of aggregated spherical structures of compound **1** are circled for clarity.

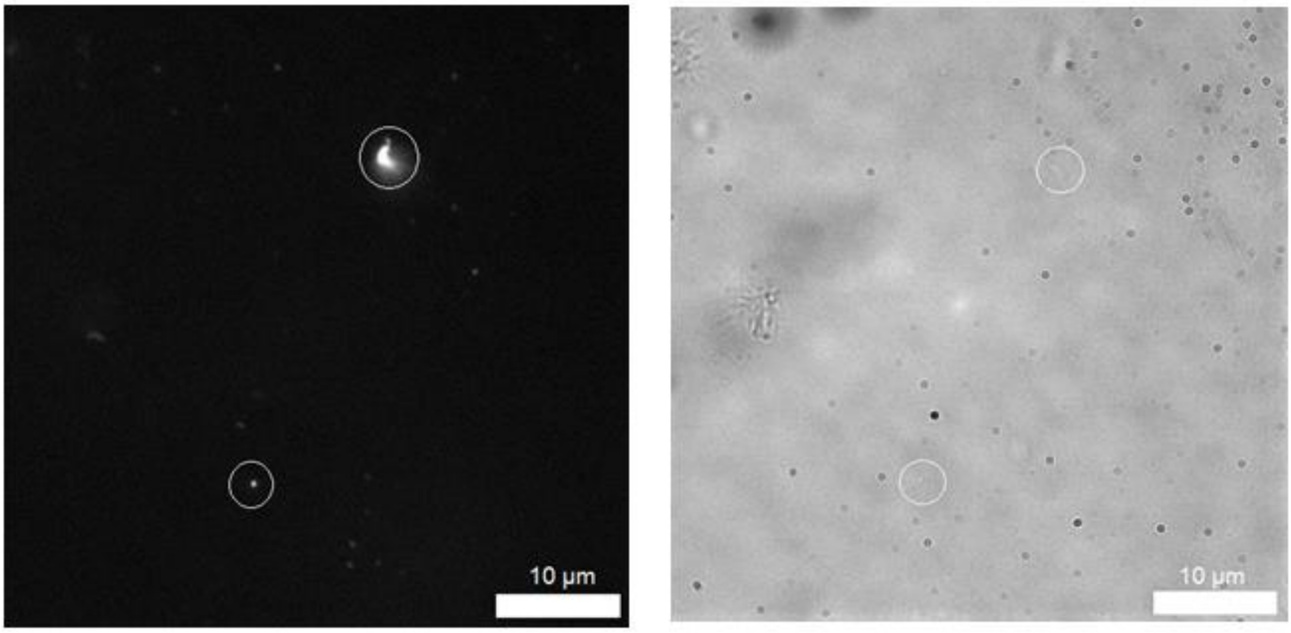

Figure S122. Left: a DAPI filtered fluorescence microscopy image of compound **1** (0.56mM) in an EtOH: H_2O (1: 19) solution. Right: an analogous transmitted light microscopy image. Evidence of aggregated spherical structures are circled for clarity. Photo bleaching during the imaging process resulted in loss of fluorescence emission intensity, as a result some aggregates could not be captured in the fluorescence microscopy image. Shape variation can be due to individual aggregation and or moving structures.

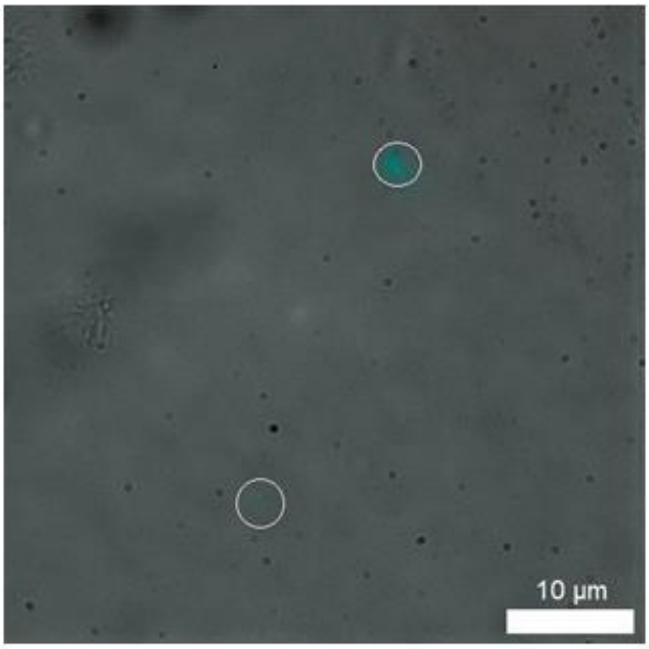

Figure S123. An overlaid image of those shown in Figure S122 (left and right) image. Clear evidence of aggregated spherical structures of compound **1** are circled for clarity.

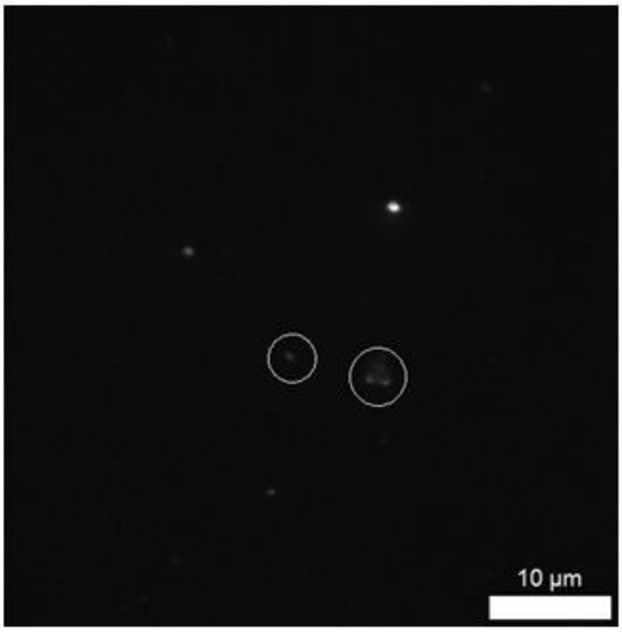

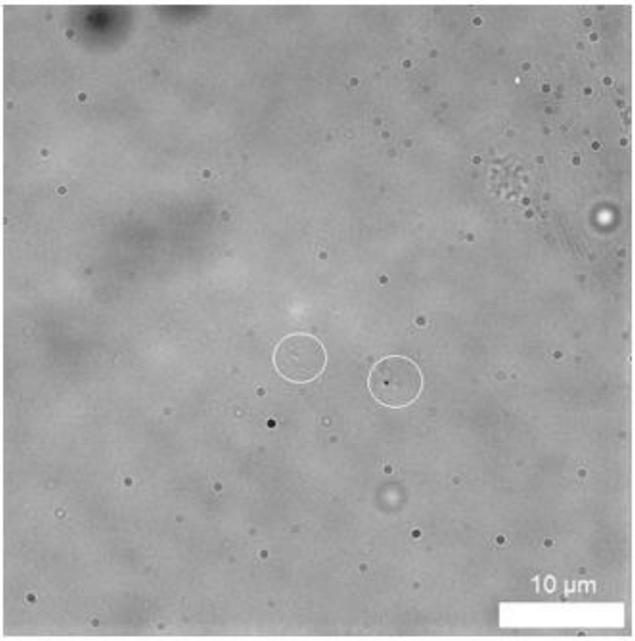

Figure S124. Left: a DAPI filtered fluorescence microscopy image of compound **1** (0.56mM) in an EtOH: H_2O (1: 19) solution. Right: an analogous transmitted light microscopy image. Evidence of aggregated spherical structures are circled for clarity. Photo bleaching during the imaging process resulted in loss of fluorescence emission intensity, as a result some aggregates could not be captured in the fluorescence microscopy image. Shape variation can be due to individual aggregation and or moving structures.

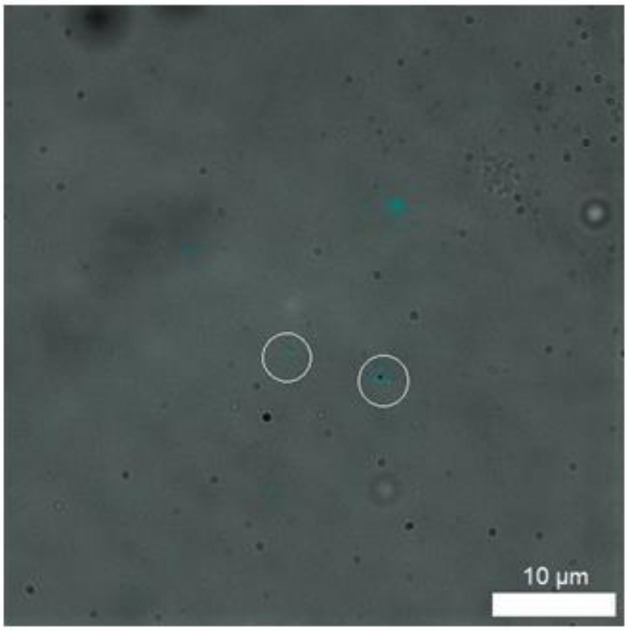

Figure S125. An overlaid image of those shown in Figure S124 (left and right) image. Clear evidence of aggregated spherical structures of compound **1** are circled for clarity.

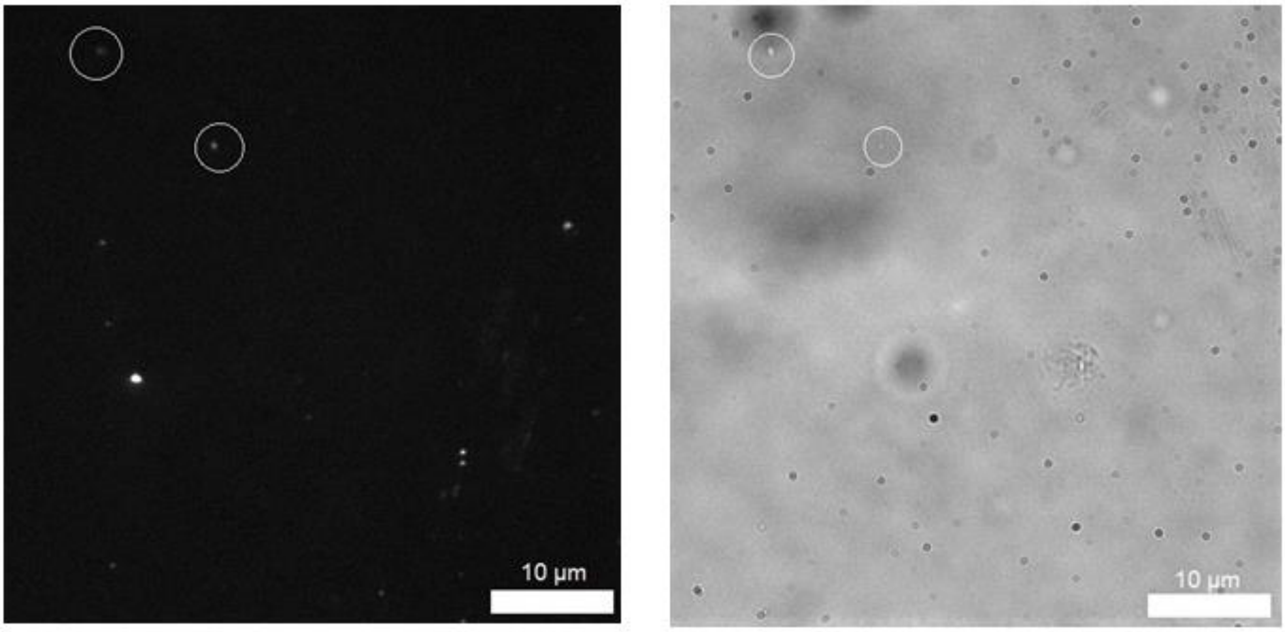

Figure S126. Left: a DAPI filtered fluorescence microscopy image of compound **1** (0.56mM) in an EtOH: H_2O (1: 19) solution. Right: an analogous transmitted light microscopy image. Evidence of aggregated spherical structures are circled for clarity. Photo bleaching during the imaging process resulted in loss of fluorescence emission intensity, as a result some aggregates could not be captured in the fluorescence microscopy image. Shape variation can be due to individual aggregation and or moving structures.

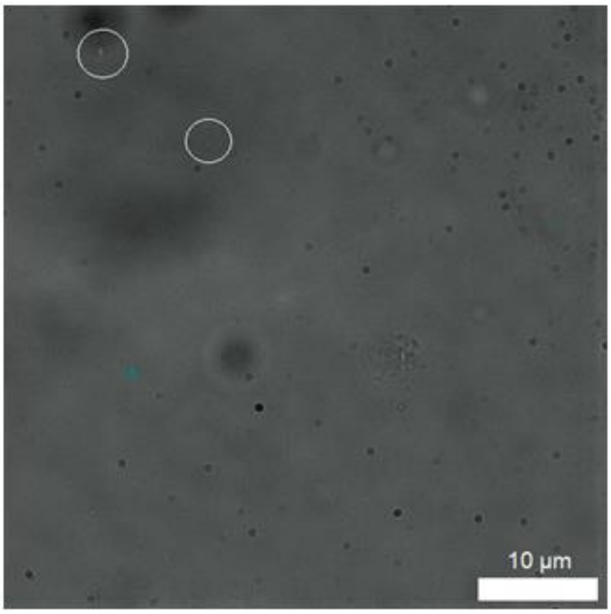

Figure S127. An overlaid image of those shown in Figure S126 (left and right) image. Clear evidence of aggregated spherical structures of compound **1** are circled for clarity.

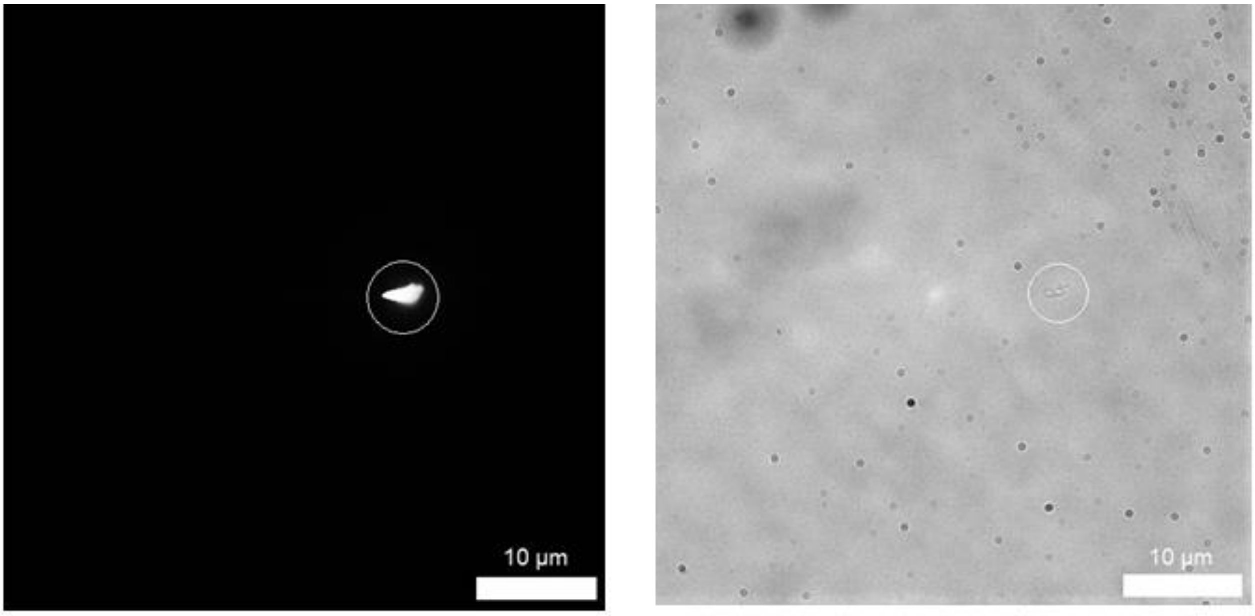

Figure S128. Left: a DAPI filtered fluorescence microscopy image of compound **2** (1mM) in an EtOH: H_2O (1: 19) solution. Right: an analogous transmitted light microscopy image. Evidence of aggregated spherical structures are circled for clarity. Photo bleaching during the imaging process resulted in loss of fluorescence emission intensity, as a result some aggregates could not be captured in the fluorescence microscopy image. Shape variation can be due to individual aggregation and or moving structures.

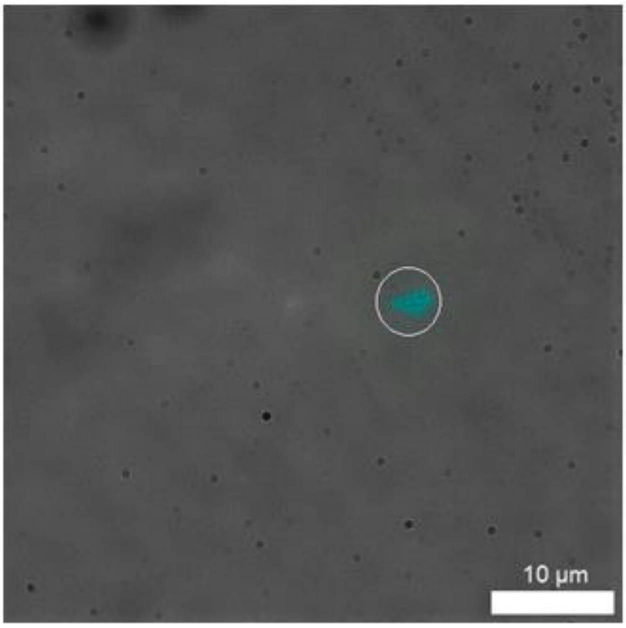

Figure S129. An overlaid image of those shown in Figure S128 (left and right) image. Clear evidence of aggregated spherical structures of compound **2** are circled for clarity.

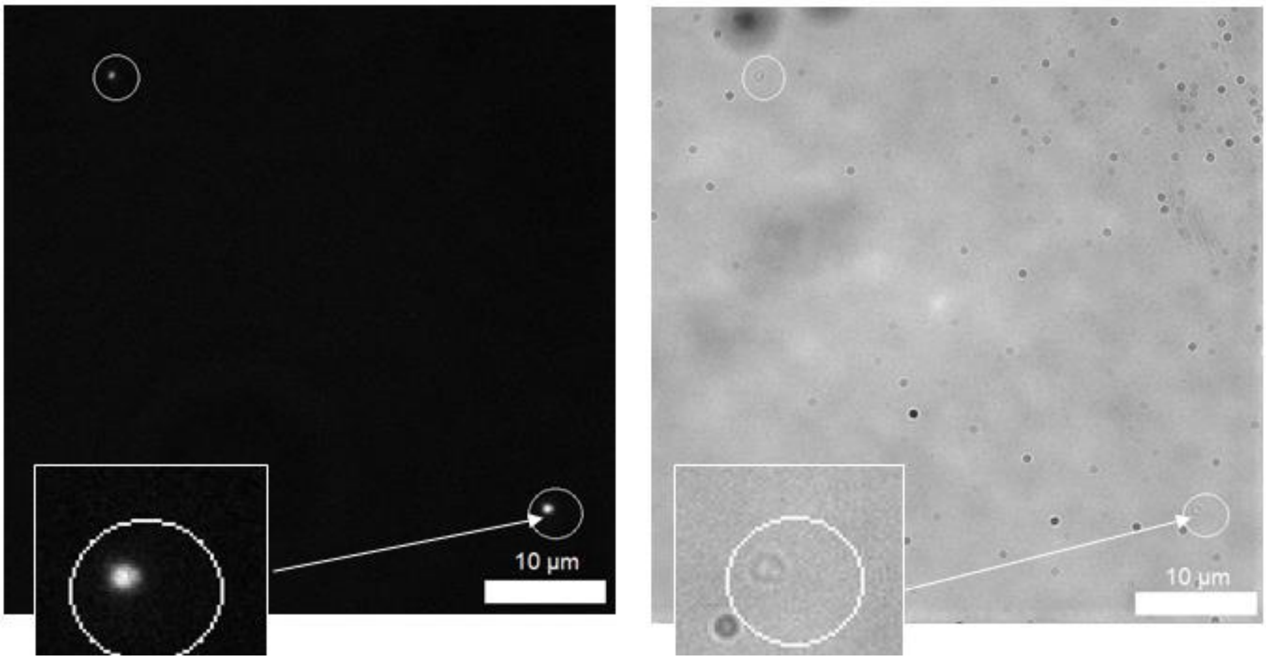

Figure S130. Left: a CFP filtered fluorescence microscopy image of compound **2** (1mM) in an EtOH: H_2O (1: 19) solution. Right: an analogous transmitted light microscopy image. Evidence of aggregated spherical structures are circled for clarity. Photo bleaching during the imaging process resulted in loss of fluorescence emission intensity, as a result some aggregates could not be captured in the fluorescence microscopy image. Shape variation can be due to individual aggregation and or moving structures.

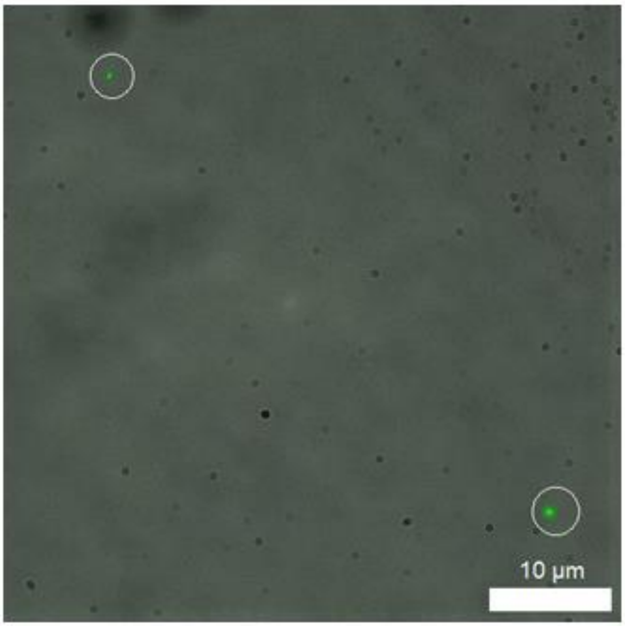

Figure S131. An overlaid image of those shown in Figure S130 (left and right) image. Clear evidence of aggregated spherical structures of compound **2** are circled for clarity.

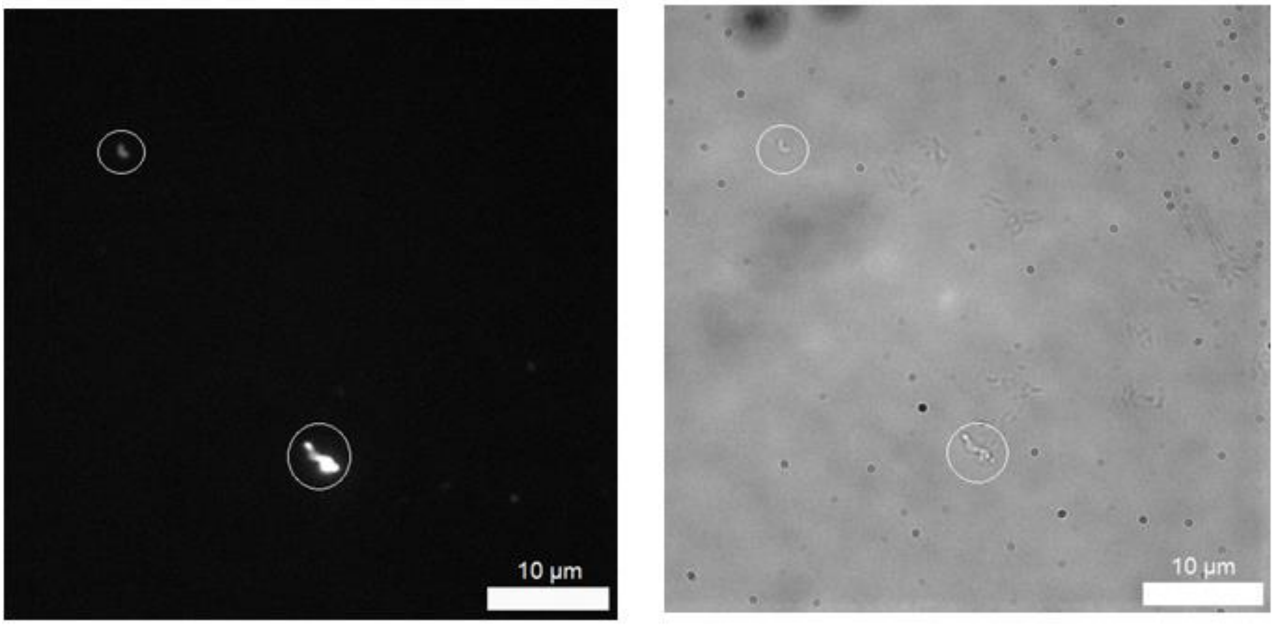

Figure S132. Left: a DAPI filtered fluorescence microscopy image of compound **2** (0.56mM) in an EtOH: H_2O (1: 19) solution. Right: an analogous transmitted light microscopy image. Evidence of aggregated spherical structures are circled for clarity. Photo bleaching during the imaging process resulted in loss of fluorescence emission intensity, as a result some aggregates could not be captured in the fluorescence microscopy image. Shape variation can be due to individual aggregation and or moving structures.

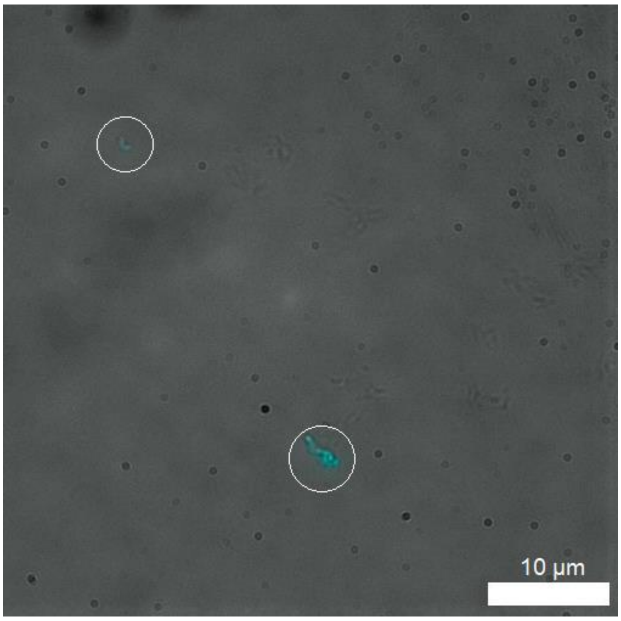

Figure S133. An overlaid image of those shown in Figure S132 (left and right) image. Clear evidence of aggregated spherical structures of compound **2** are circled for clarity.

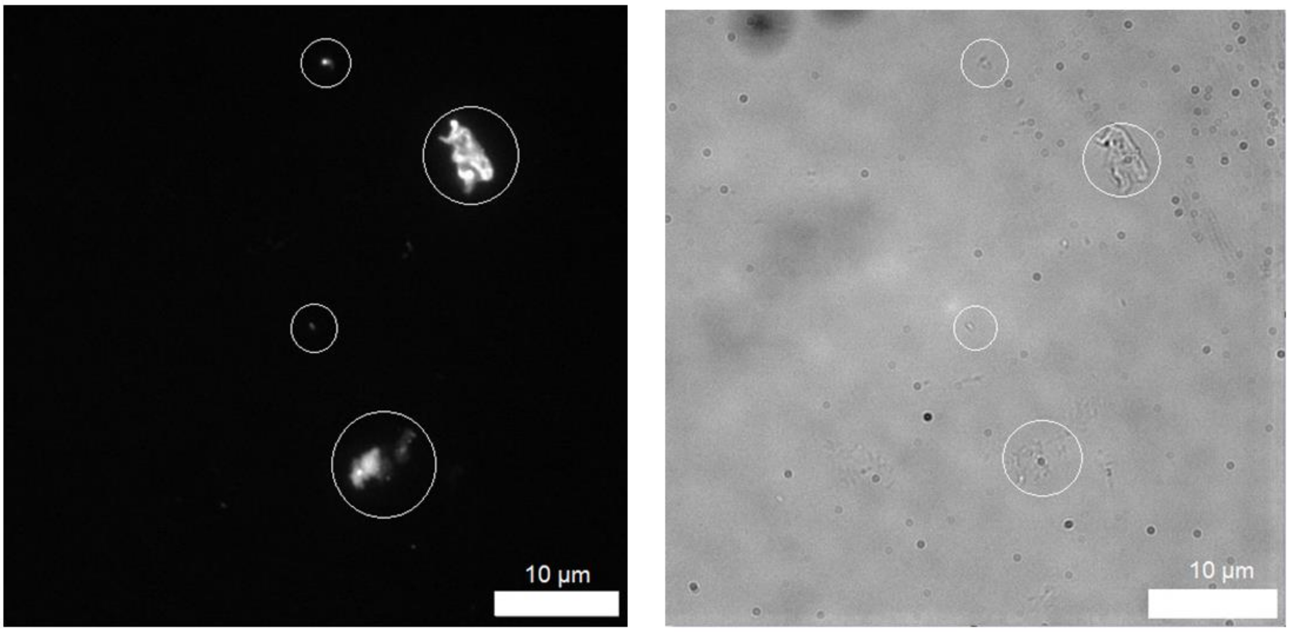

Figure S134. Left: a DAPI filtered fluorescence microscopy image of compound **2** (0.56mM) in an EtOH: H_2O (1: 19) solution. Right: an analogous transmitted light microscopy image. Evidence of aggregated spherical structures are circled for clarity. Photo bleaching during the imaging process resulted in loss of fluorescence emission intensity, as a result some aggregates could not be captured in the fluorescence microscopy image. Shape variation can be due to individual aggregation and or moving structures.

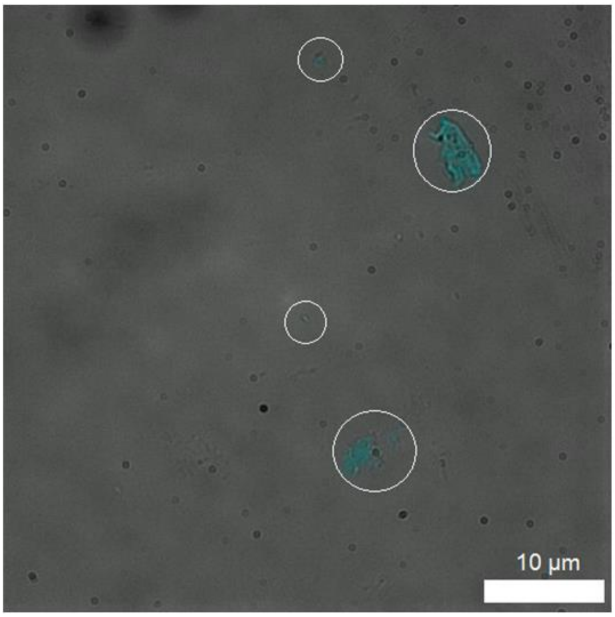

Figure S135. An overlaid image of those shown in Figure S134 (left and right) image. Clear evidence of aggregated spherical structures of compound **2** are circled for clarity.

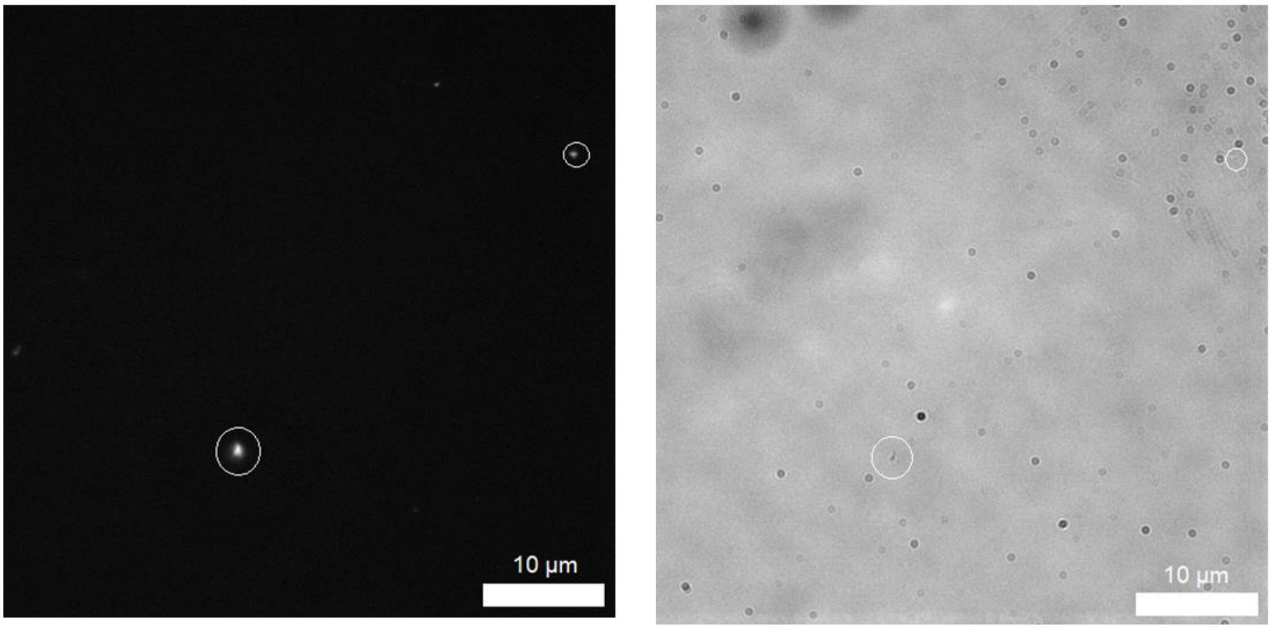

Figure S136. Left: a DAPI filtered fluorescence microscopy image of compound **2** (0.56mM) in an EtOH: H_2O (1: 19) solution. Right: an analogous transmitted light microscopy image. Evidence of aggregated spherical structures are circled for clarity. Photo bleaching during the imaging process resulted in loss of fluorescence emission intensity, as a result some aggregates could not be captured in the fluorescence microscopy image. Shape variation can be due to individual aggregation and or moving structures.

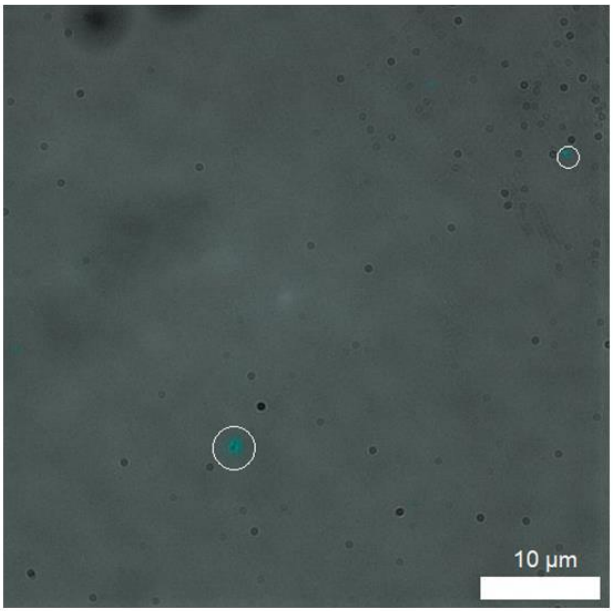

Figure S137. An overlaid image of those shown in Figure S136 (left and right) image. Clear evidence of aggregated spherical structures of compound **2** are circled for clarity.

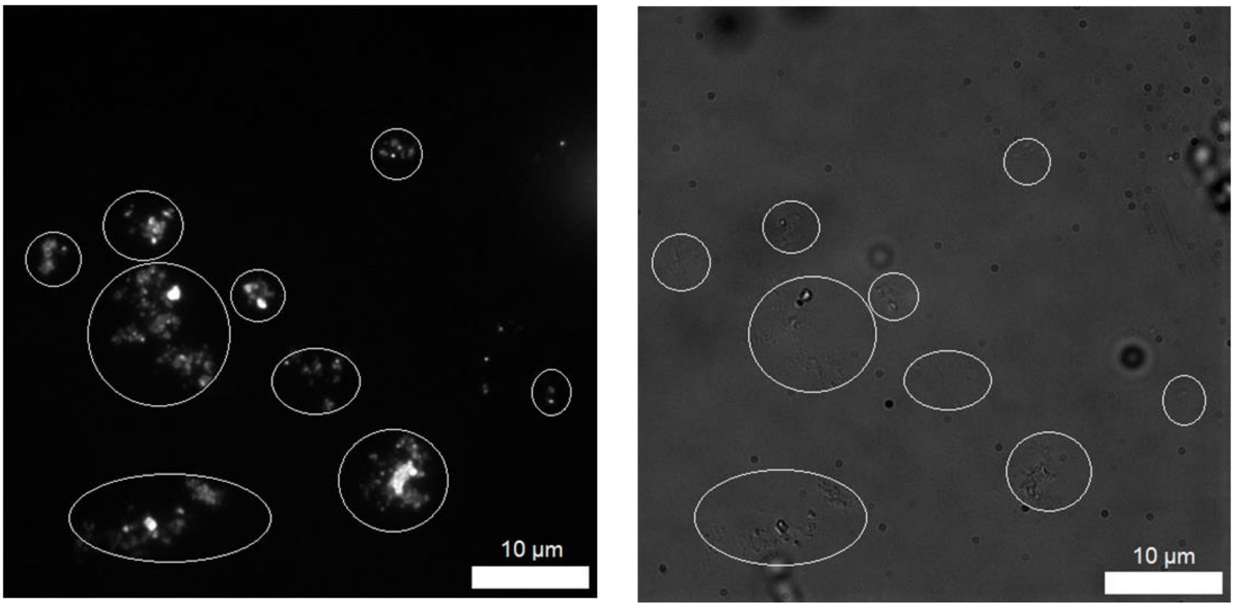

Figure S138. Left: a DAPI filtered fluorescence microscopy image of compound **4** (1mM) in an EtOH: H_2O (1: 19) solution. Right: an analogous transmitted light microscopy image. Evidence of aggregated spherical structures are circled for clarity. Photo bleaching during the imaging process resulted in loss of fluorescence emission intensity, as a result some aggregates could not be captured in the fluorescence microscopy image. Shape variation can be due to individual aggregation and or moving structures.

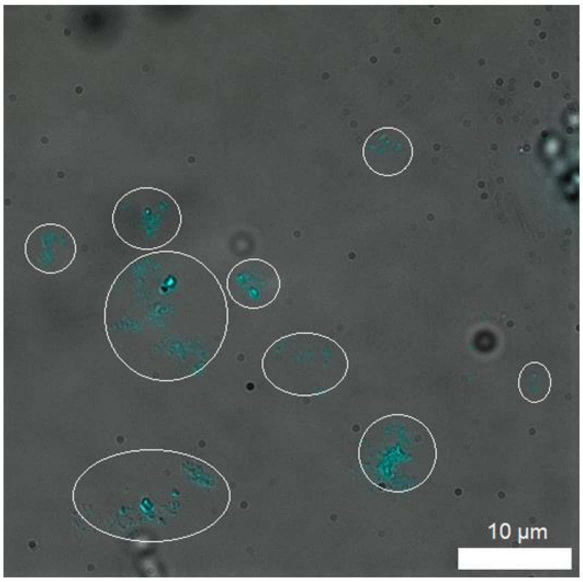

Figure S139. An overlaid image of those shown in Figure S138 (left and right) image. Clear evidence of aggregated spherical structures of compound **4** are circled for clarity.

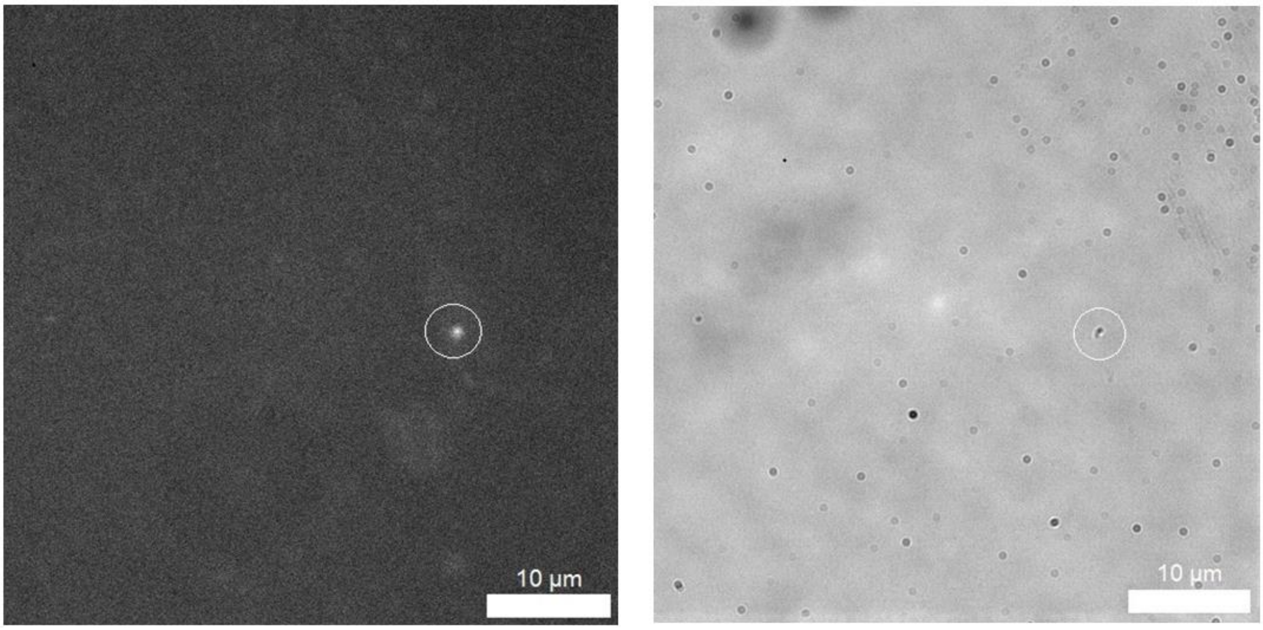

Figure S140. Left: a DAPI filtered fluorescence microscopy image of compound **4** (1mM) in an EtOH: H_2O (1: 19) solution. Right: an analogous transmitted light microscopy image. Evidence of aggregated spherical structures are circled for clarity. Photo bleaching during the imaging process resulted in loss of fluorescence emission intensity, as a result some aggregates could not be captured in the fluorescence microscopy image. Shape variation can be due to individual aggregation and or moving structures.

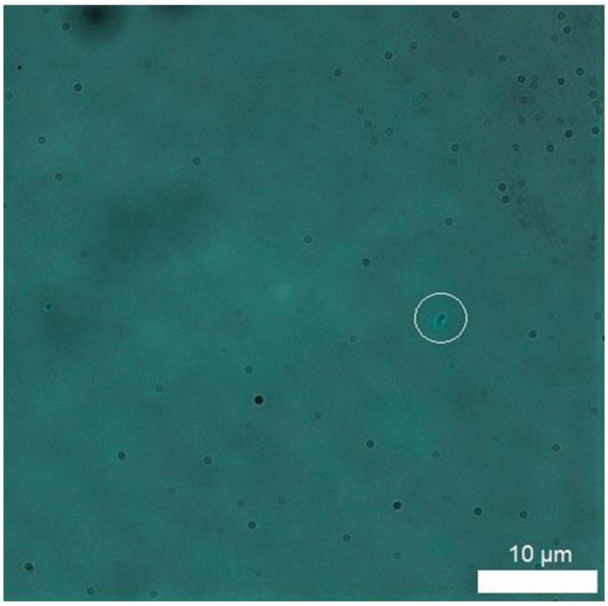

Figure S141. An overlaid image of those shown in Figure S140 (left and right) image. Clear evidence of aggregated spherical structures of compound **4** are circled for clarity.

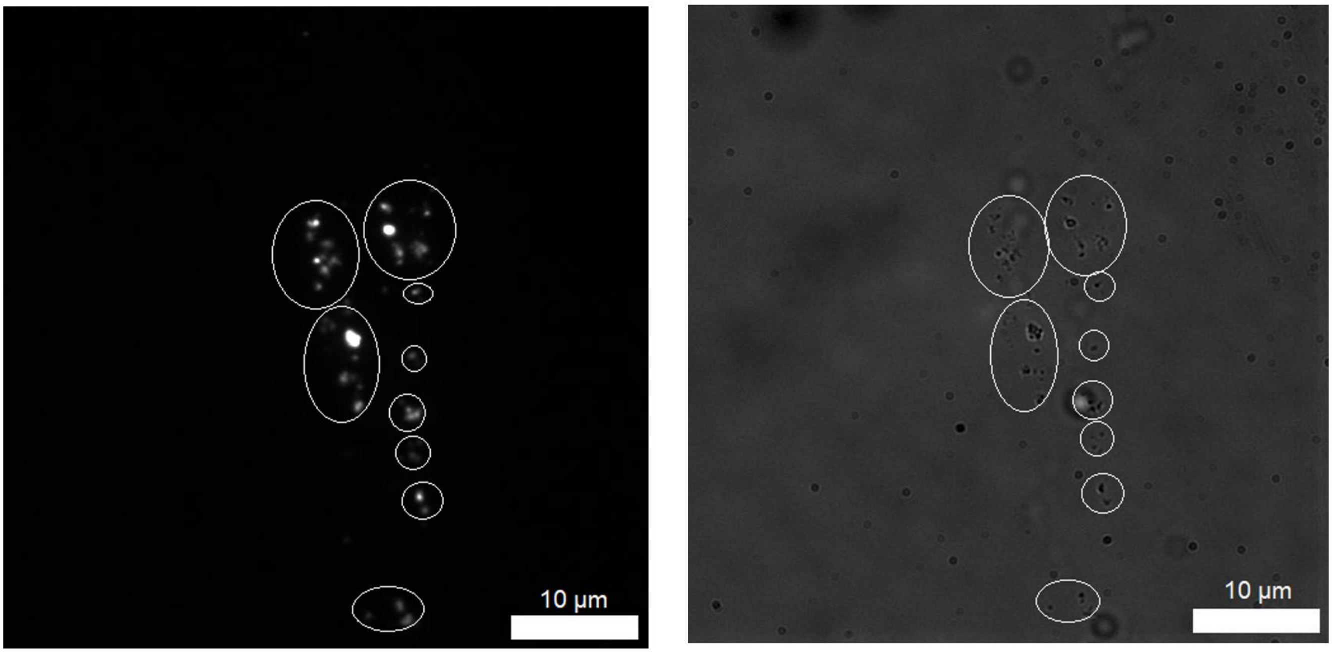

Figure S142. Left: a DAPI filtered fluorescence microscopy image of compound **4** (1mM) in an EtOH: H_2O (1: 19) solution. Right: an analogous transmitted light microscopy image. Evidence of aggregated spherical structures are circled for clarity. Photo bleaching during the imaging process resulted in loss of fluorescence emission intensity, as a result some aggregates could not be captured in the fluorescence microscopy image. Shape variation can be due to individual aggregation and or moving structures.

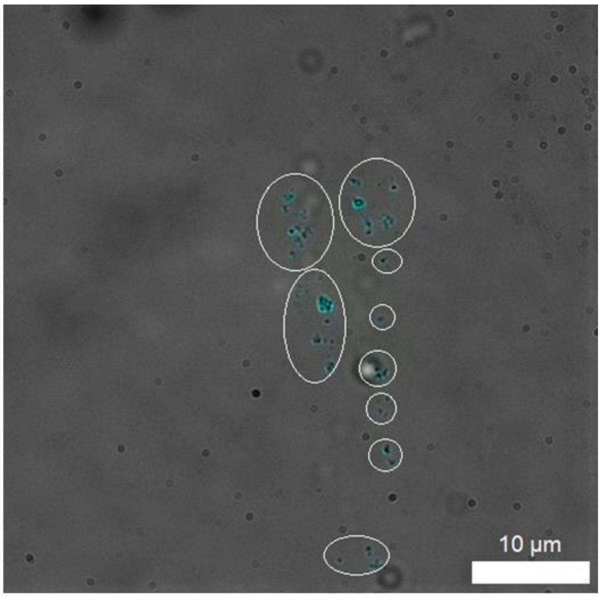

Figure S143. An overlaid image of those shown in Figure S142 (left and right) image. Clear evidence of aggregated spherical structures of compound **4** are circled for clarity.

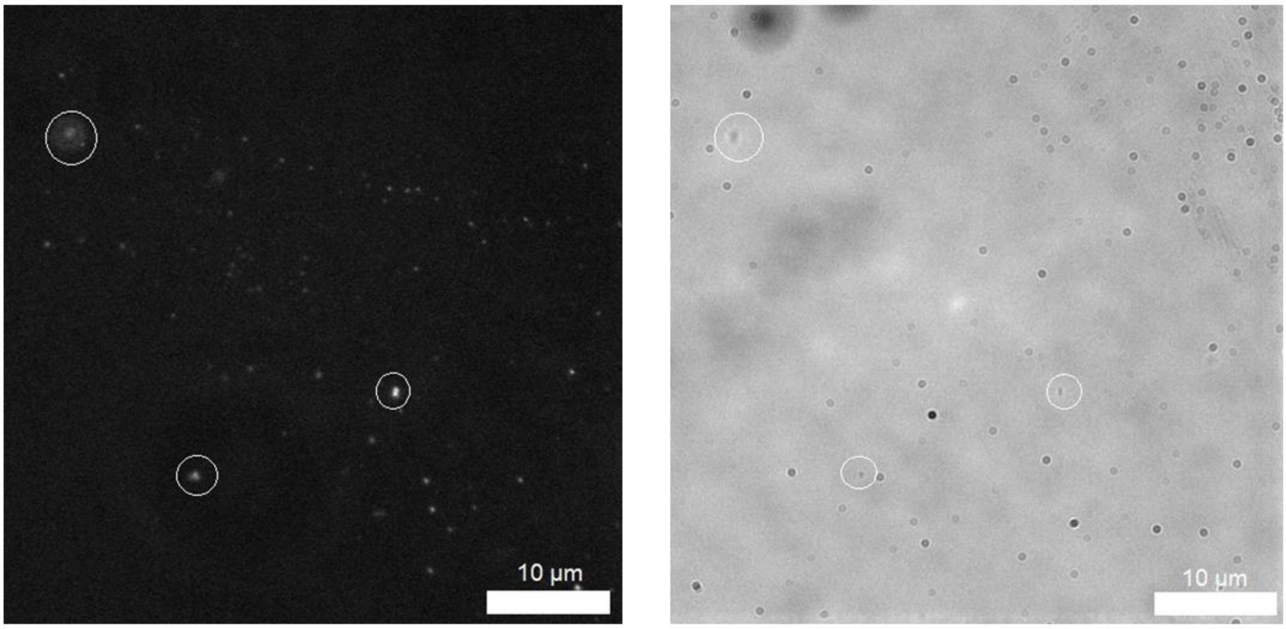

Figure S144. Left: a CFP filtered fluorescence microscopy image of compound **4** (0.56mM) in an EtOH: H_2O (1: 19) solution. Right: an analogous transmitted light microscopy image. Evidence of aggregated spherical structures are circled for clarity. Photo bleaching during the imaging process resulted in loss of fluorescence emission intensity, as a result some aggregates could not be captured in the fluorescence microscopy image. Shape variation can be due to individual aggregation and or moving structures.

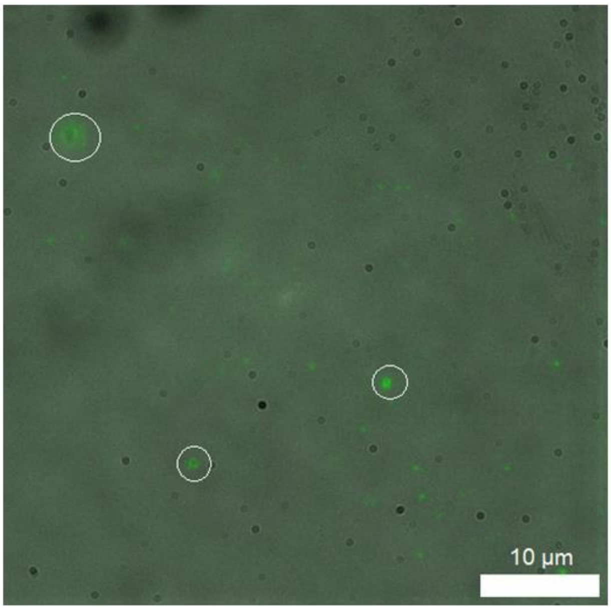

Figure S145. An overlaid image of those shown in Figure S144 (left and right) image. Clear evidence of aggregated spherical structures of compound **4** are circled for clarity.

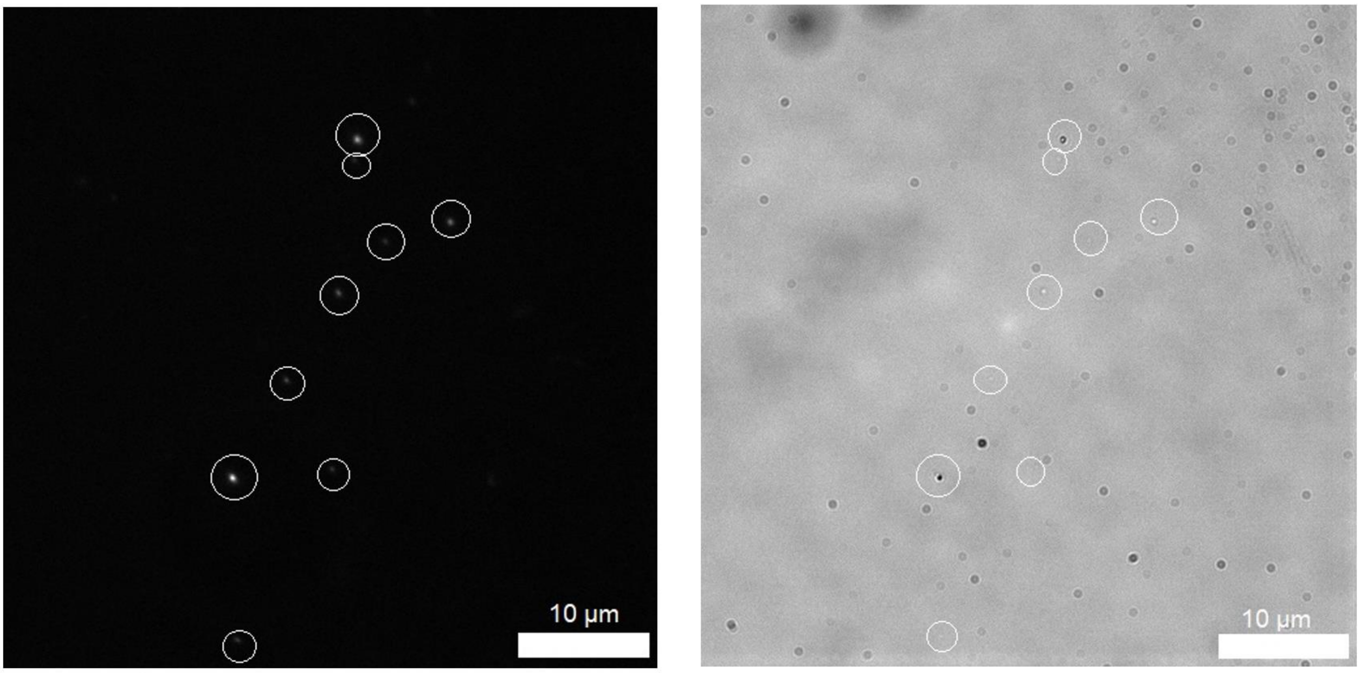

Figure S146. Left: a CFP filtered fluorescence microscopy image of compound **4** (0.56mM) in an EtOH: H_2O (1: 19) solution. Right: an analogous transmitted light microscopy image. Evidence of aggregated spherical structures are circled for clarity. Photo bleaching during the imaging process resulted in loss of fluorescence emission intensity, as a result some aggregates could not be captured in the fluorescence microscopy image. Shape variation can be due to individual aggregation and or moving structures.

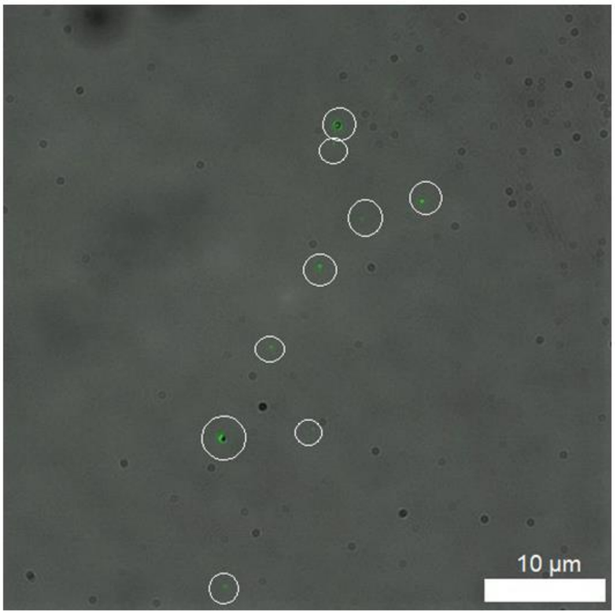

Figure S147. An overlaid image of those shown in Figure S146 (left and right) image. Clear evidence of aggregated spherical structures of compound **4** are circled for clarity.

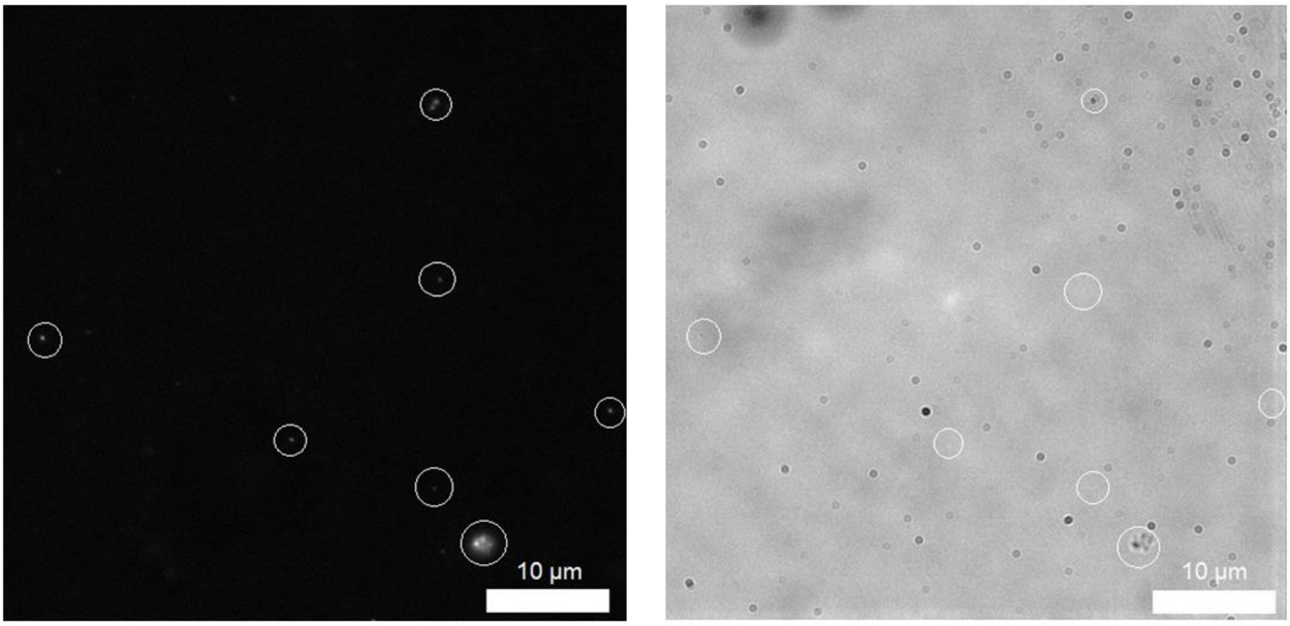

Figure S148. Left: a CFP filtered fluorescence microscopy image of compound **4** (0.56mM) in an EtOH: H_2O (1: 19) solution. Right: an analogous transmitted light microscopy image. Evidence of aggregated spherical structures are circled for clarity. Photo bleaching during the imaging process resulted in loss of fluorescence emission intensity, as a result some aggregates could not be captured in the fluorescence microscopy image. Shape variation can be due to individual aggregation and or moving structures.

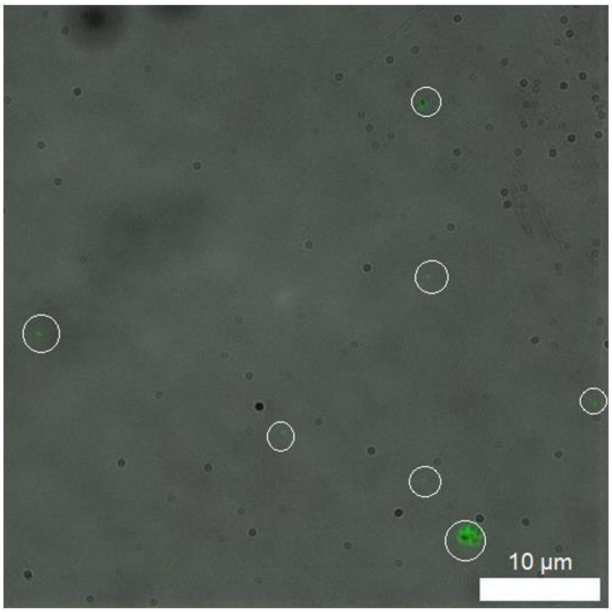

Figure S149. An overlaid image of those shown in Figure S148 (left and right) image. Clear evidence of aggregated spherical structures of compound **4** are circled for clarity.

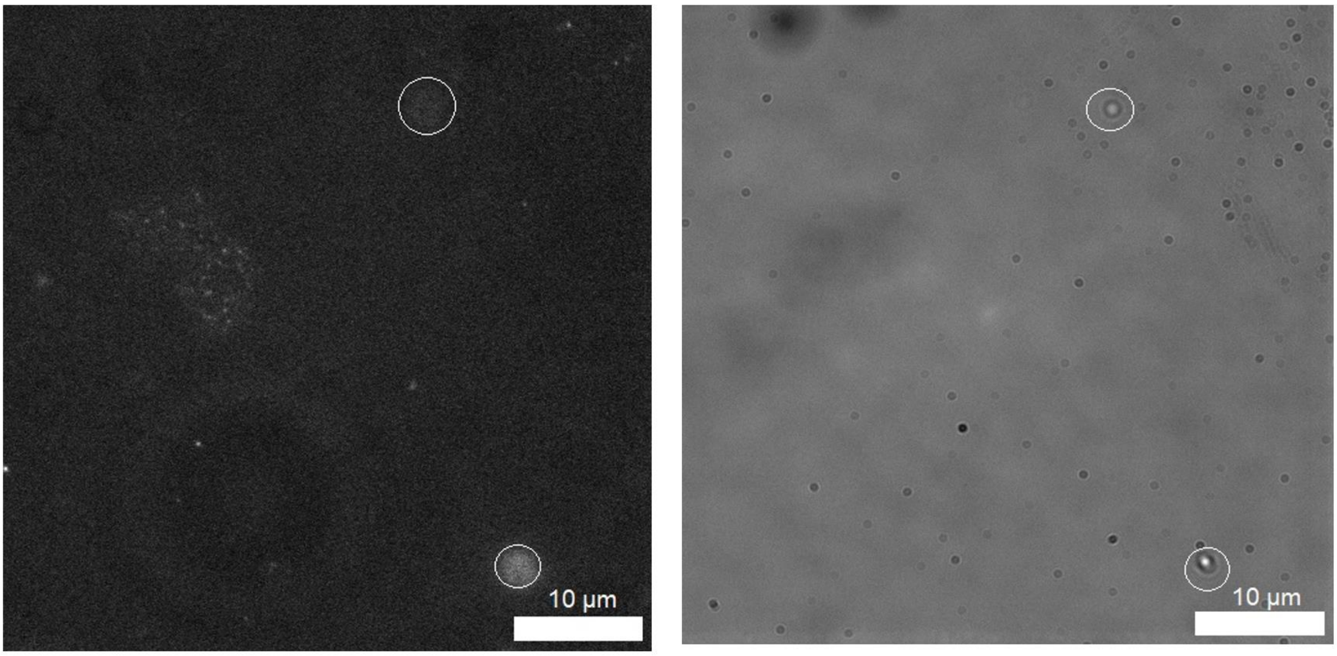

Figure S150. Left: a CFP filtered fluorescence microscopy image of compound **4** (0.56mM) in an EtOH: H_2O (1: 19) solution. Right: an analogous transmitted light microscopy image. Evidence of aggregated spherical structures are circled for clarity. Photo bleaching during the imaging process resulted in loss of fluorescence emission intensity, as a result some aggregates could not be captured in the fluorescence microscopy image. Shape variation can be due to individual aggregation and or moving structures.

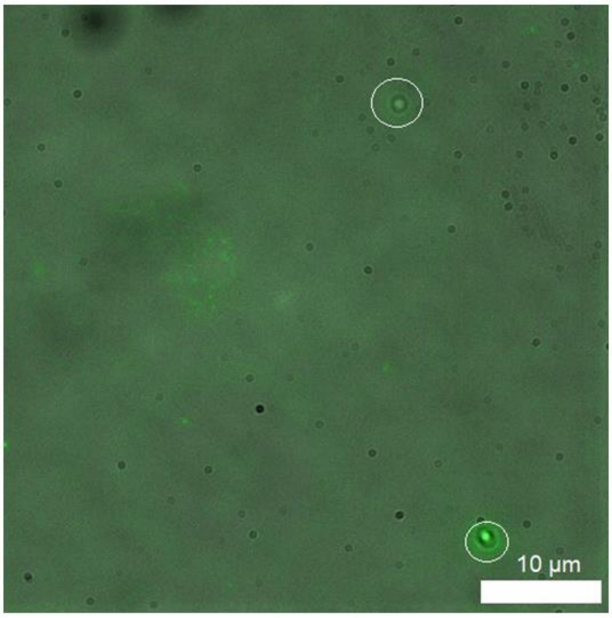

Figure S151. An overlaid image of those shown in Figure S150 (left and right) image. Clear evidence of aggregated spherical structures of compound **4** are circled for clarity.

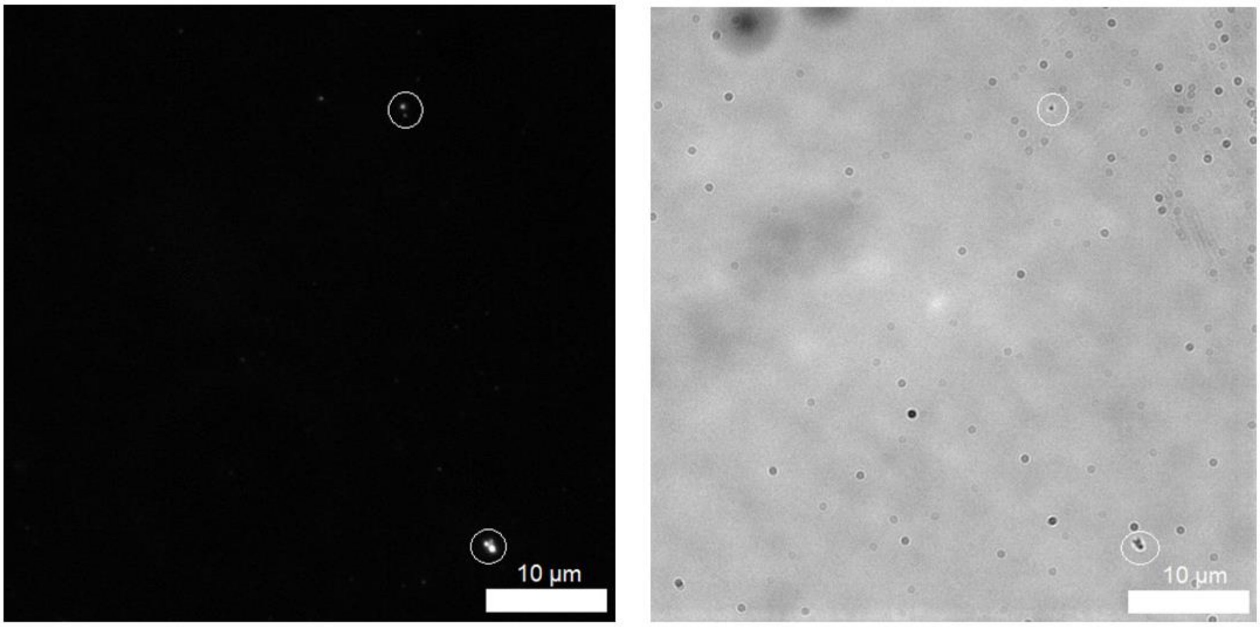

Figure S152. Left: a CFP filtered fluorescence microscopy image of compound **4** (0.56mM) in an EtOH: H_2O (1: 19) solution. Right: an analogous transmitted light microscopy image. Evidence of aggregated spherical structures are circled for clarity. Photo bleaching during the imaging process resulted in loss of fluorescence emission intensity, as a result some aggregates could not be captured in the fluorescence microscopy image. Shape variation can be due to individual aggregation and or moving structures.

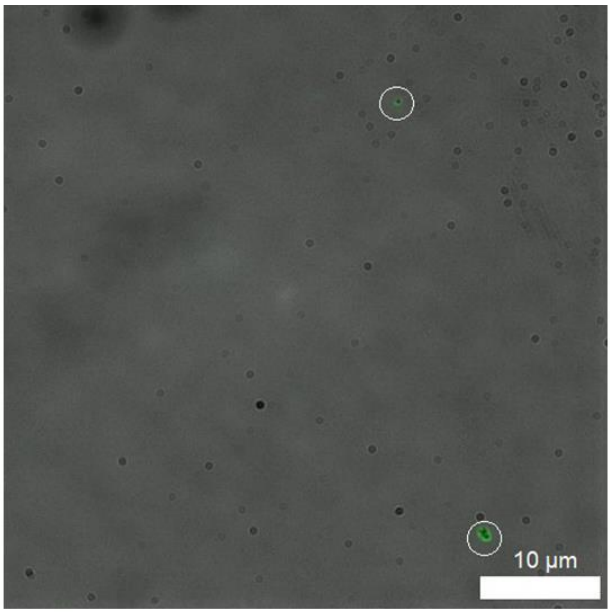

Figure S153. An overlaid image of those shown in Figure S152 (left and right) image. Clear evidence of aggregated spherical structures of compound **3** are circled for clarity.

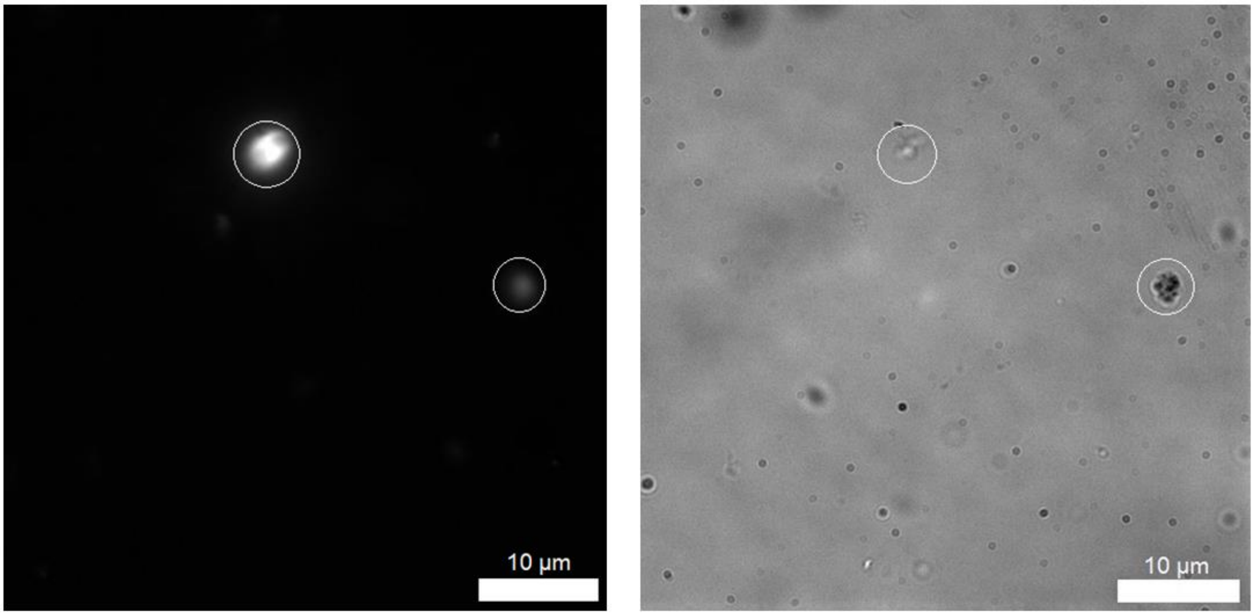

Figure S154. Left: a DAPI filtered fluorescence microscopy image of compound **1** and **2** (1:1 mix) (total concentration 1mM) in an EtOH: H_2O (1: 19) solution. Right: an analogous transmitted light microscopy image. Evidence of aggregated spherical structures are circled for clarity. Photo bleaching during the imaging process resulted in loss of fluorescence emission intensity, as a result some aggregates could not be captured in the fluorescence microscopy image. Shape variation can be due to individual aggregation and or moving structures.

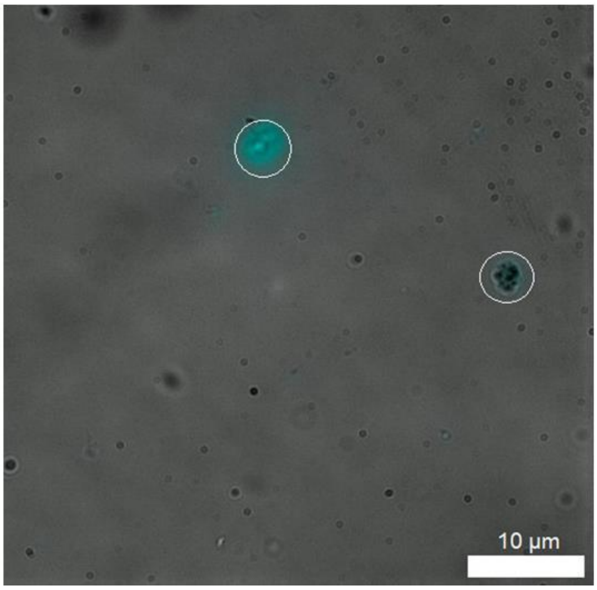

Figure S155. An overlaid image of those shown in Figure S154 (left and right) image. Clear evidence of aggregated spherical structures of compound **1** and **2** (1:1 mix) (total concentration 1mM) are circled for clarity.

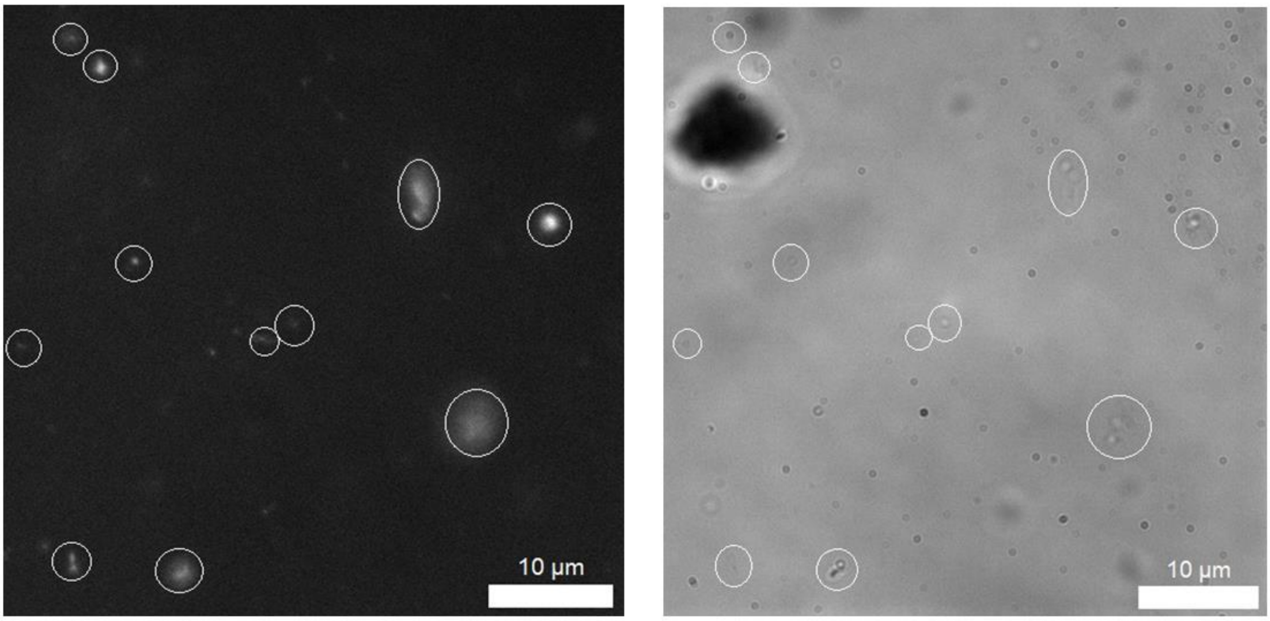

Figure S156. Left: a DAPI filtered fluorescence microscopy image of compound **1** and **2** (1:1 mix) (total concentration 1mM) in an EtOH: H_2O (1: 19) solution. Right: an analogous transmitted light microscopy image. Evidence of aggregated spherical structures are circled for clarity. Photo bleaching during the imaging process resulted in loss of fluorescence emission intensity, as a result some aggregates could not be captured in the fluorescence microscopy image. Shape variation can be due to individual aggregation and or moving structures.

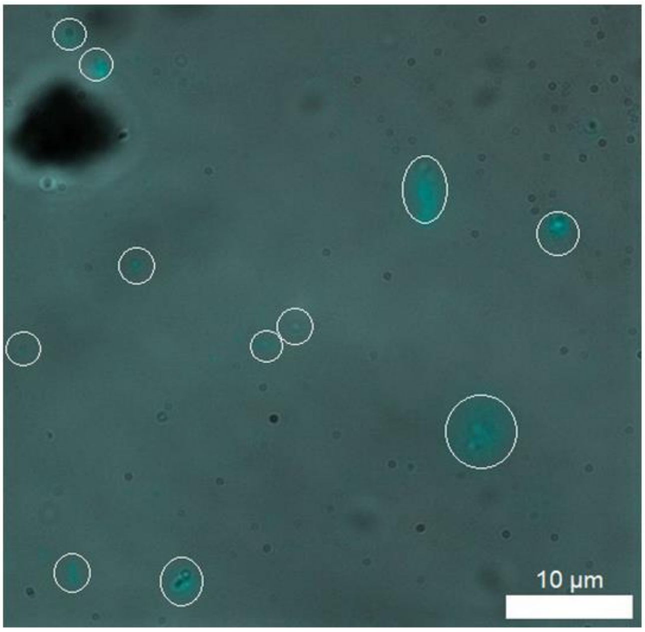

Figure S157. An overlaid image of those shown in Figure S156 (left and right) image. Clear evidence of aggregated spherical structures of compound **1** and **2** (1:1 mix) (total concentration 1mM) are circled for clarity.

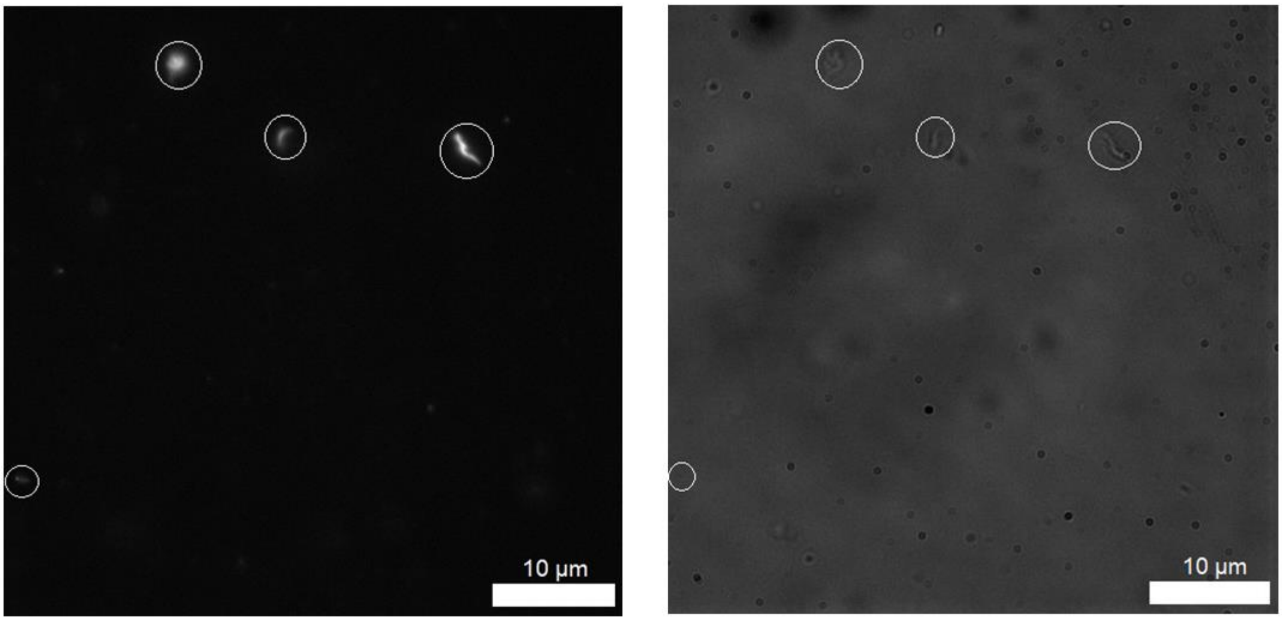

Figure S158. Left: a DAPI filtered fluorescence microscopy image of compound **1** and **2** (1:1 mix) (total concentration 1mM) in an EtOH: H_2O (1: 19) solution. Right: an analogous transmitted light microscopy image. Evidence of aggregated spherical structures are circled for clarity. Photo bleaching during the imaging process resulted in loss of fluorescence emission intensity, as a result some aggregates could not be captured in the fluorescence microscopy image. Shape variation can be due to individual aggregation and or moving structures.

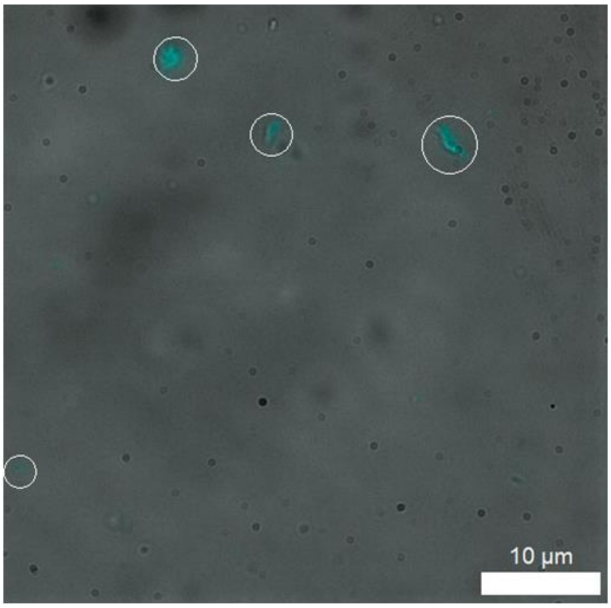

Figure S159. An overlaid image of those shown in Figure S158 (left and right) image. Clear evidence of aggregated spherical structures of compound **1** and **2** (1:1 mix) (total concentration 1mM) are circled for clarity.

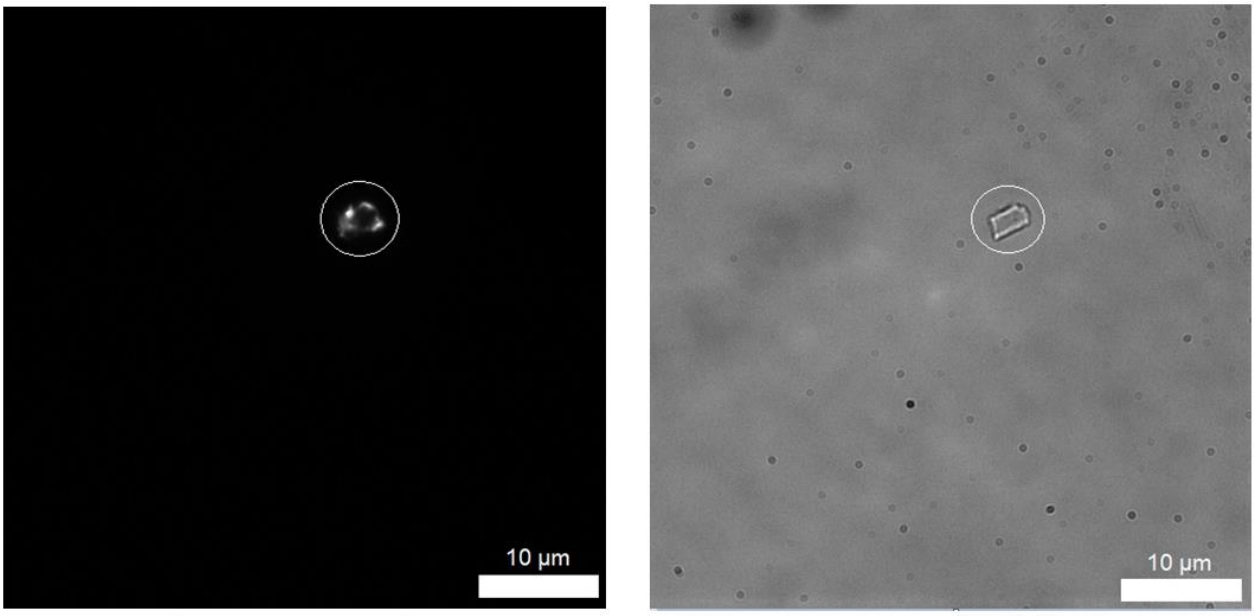

Figure S160. Left: a DAPI filtered fluorescence microscopy image of compound **1** and **2** (1:1 mix) (total concentration 0.56 mM) in an EtOH: H_2O (1: 19) solution. Right: an analogous transmitted light microscopy image. Evidence of aggregated spherical structures are circled for clarity. Photo bleaching during the imaging process resulted in loss of fluorescence emission intensity, as a result some aggregates could not be captured in the fluorescence microscopy image. Shape variation can be due to individual aggregation and or moving structures.

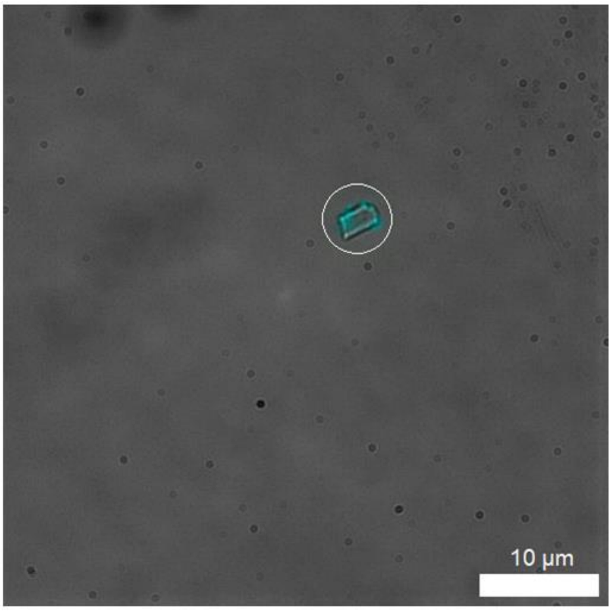

Figure S161. An overlaid image of those shown in Figure S160 (left and right) image. Clear evidence of aggregated spherical structures of compound **1** and **2** (1:1 mix) (total concentration 0.56 mM) are circled for clarity.

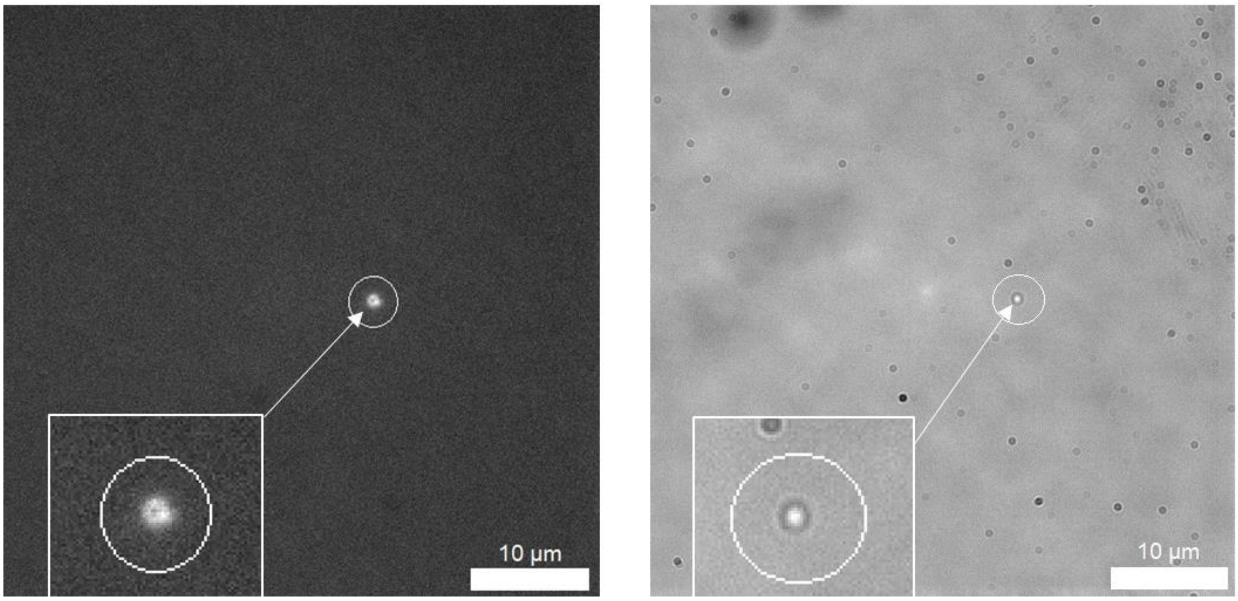

Figure S162. Left: a DAPI filtered fluorescence microscopy image of compound **1** and **2** (1:1 mix) (total concentration 0.56 mM) in an EtOH: H_2O (1: 19) solution. Right: an analogous transmitted light microscopy image. Evidence of aggregated spherical structures are circled for clarity. Photo bleaching during the imaging process resulted in loss of fluorescence emission intensity, as a result some aggregates could not be captured in the fluorescence microscopy image. Shape variation can be due to individual aggregation and or moving structures.

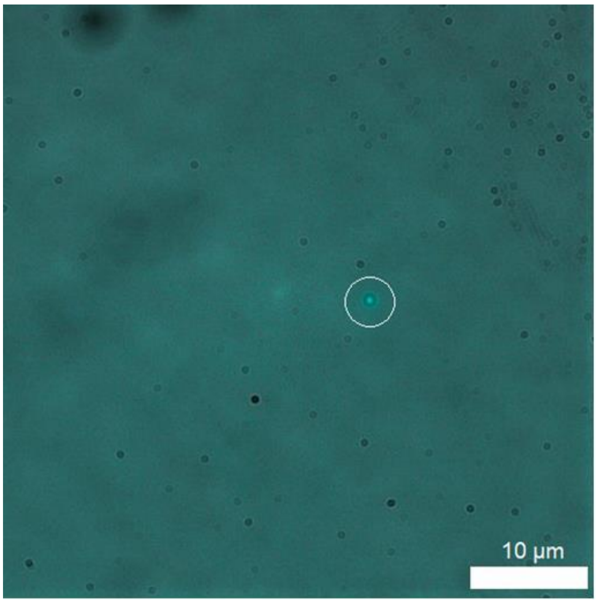

Figure S163. An overlaid image of those shown in Figure S162 (left and right) image. Clear evidence of aggregated spherical structures of compound **1** and **2** (1:1 mix) (total concentration 0.56 mM) are circled for clarity.

MIC$_{50}$ Determination

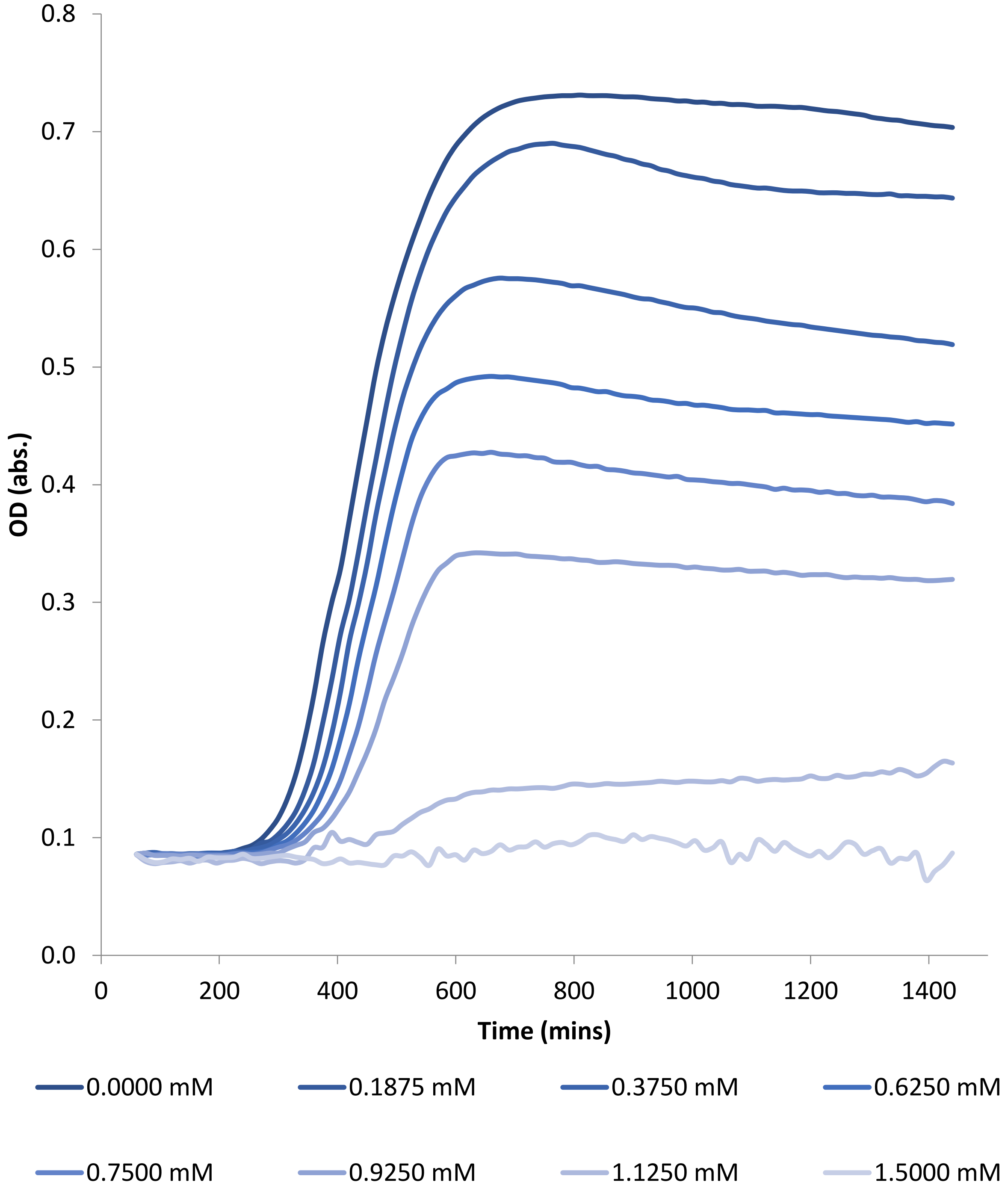

Figure S164. Average growth curves obtained for MRSA USA300 in the presence of compound **1** at eight different concentrations.

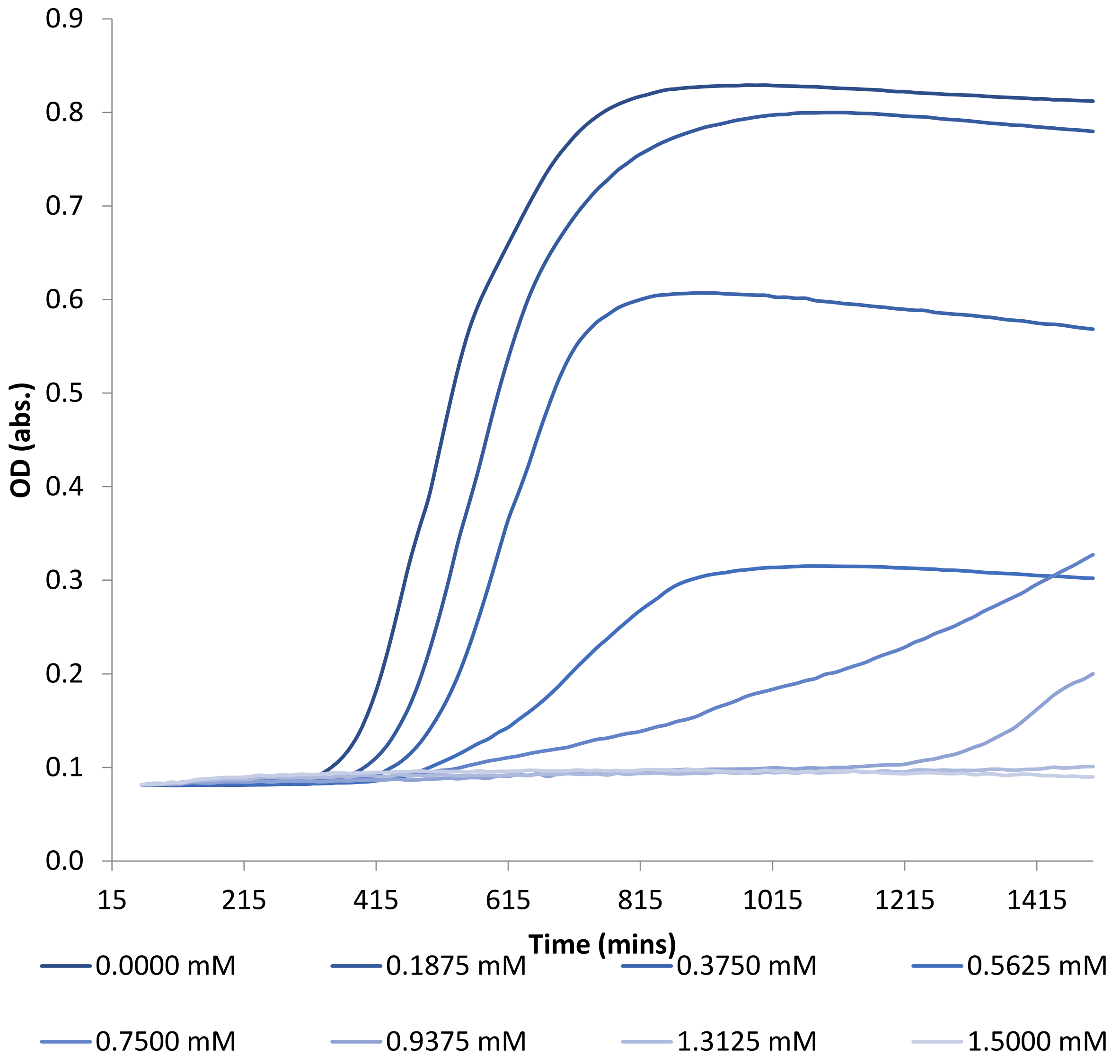

Figure S165. Average growth curves obtained for MRSA USA300 in the presence of compound **2** at eight different concentrations.

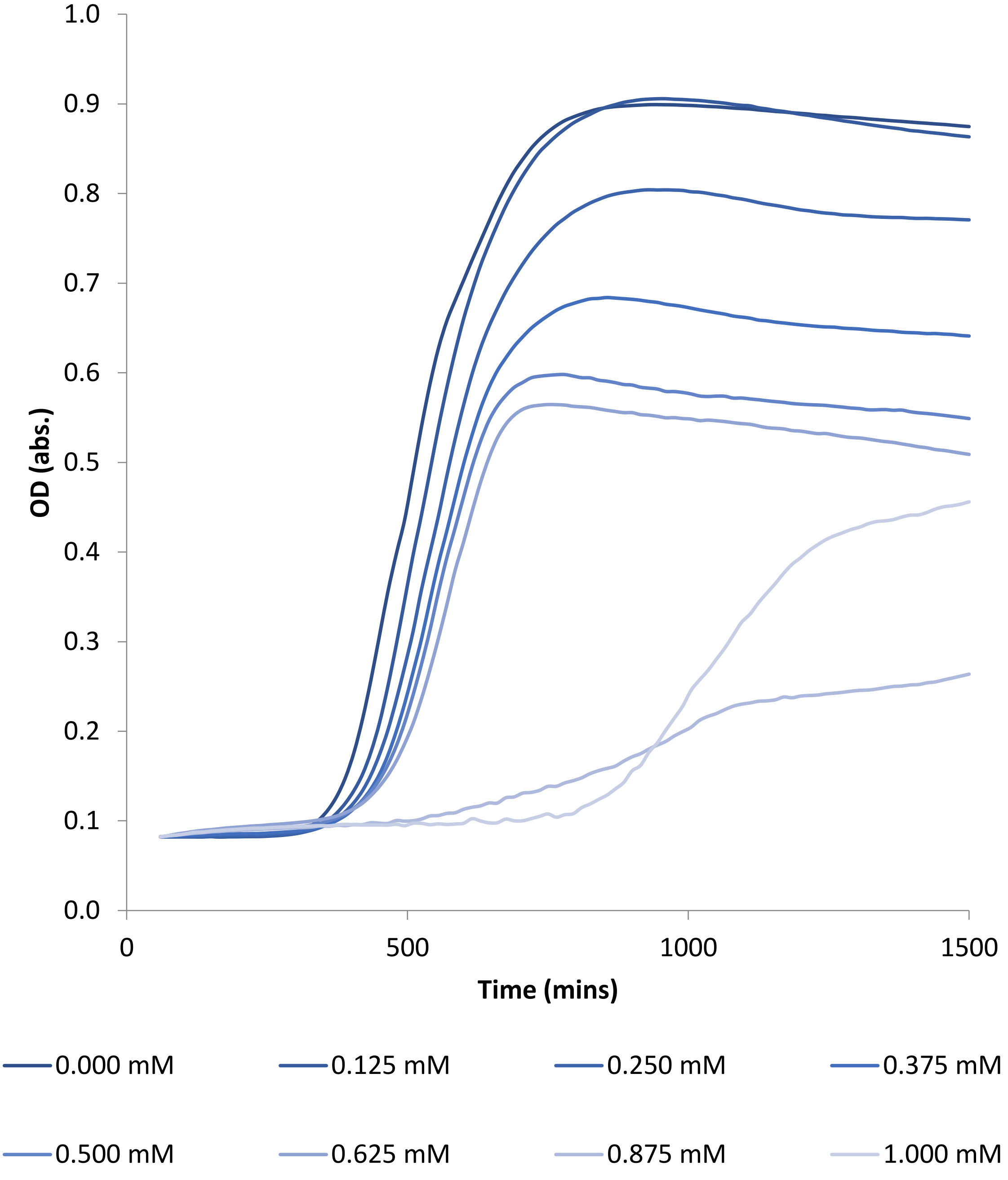

Figure S166. Average growth curves obtained for MRSA USA300 in the presence of compound **3** at eight different concentrations.

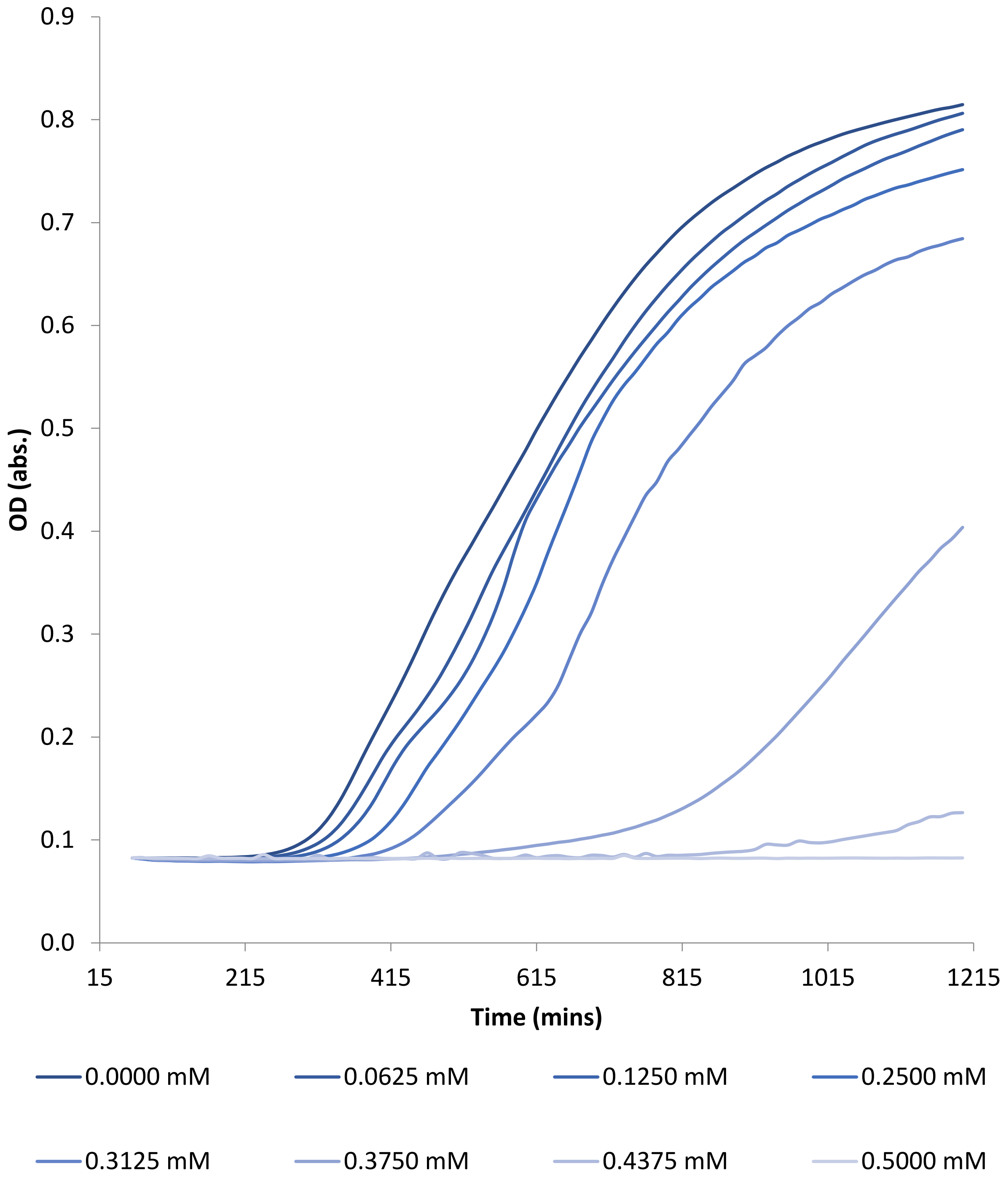

Figure S167. Average growth curves obtained for MRSA USA300 in the presence of compounds **1** and **2** (1:1 mixture) at eight different total molecular concentrations.

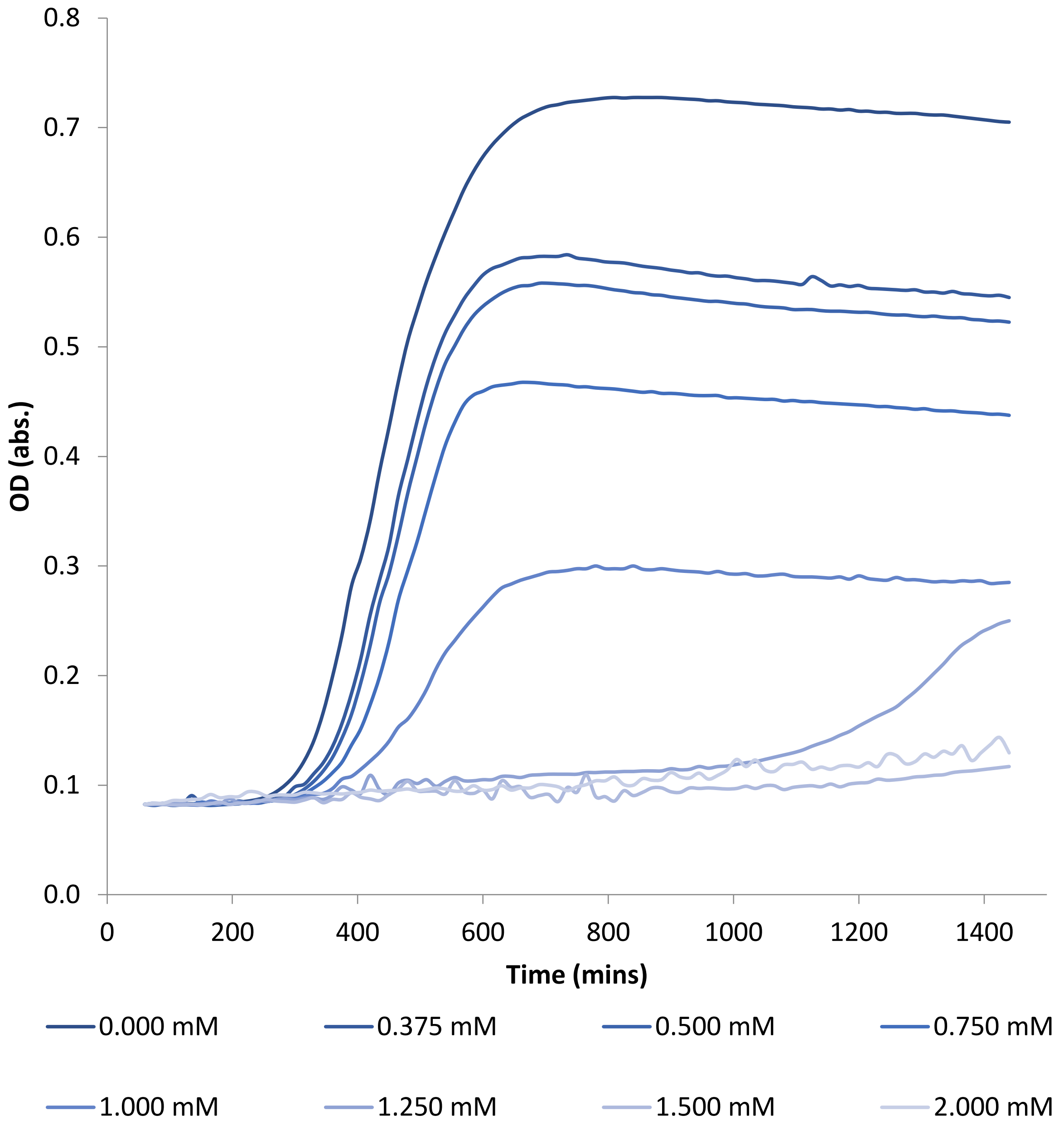

Figure S168. Average growth curves obtained for MRSA USA300 in the presence of compounds **1** and **4** (1:1 mixture) at eight different total molecular concentrations.

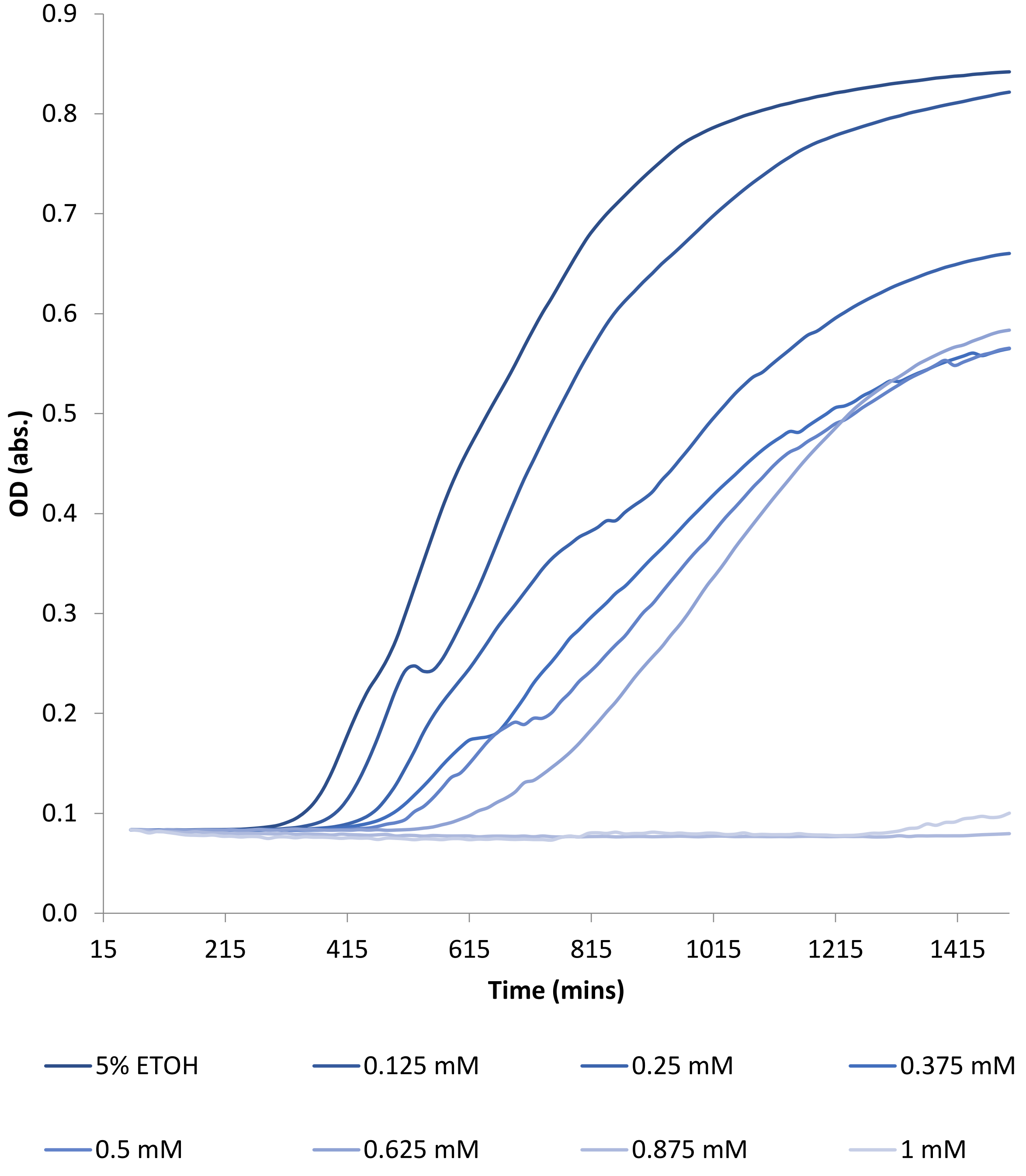

Figure S169. Average growth curves obtained for MRSA USA300 in the presence of compounds **2** and **4** (1:1 mixture) at eight different total molecular concentrations.

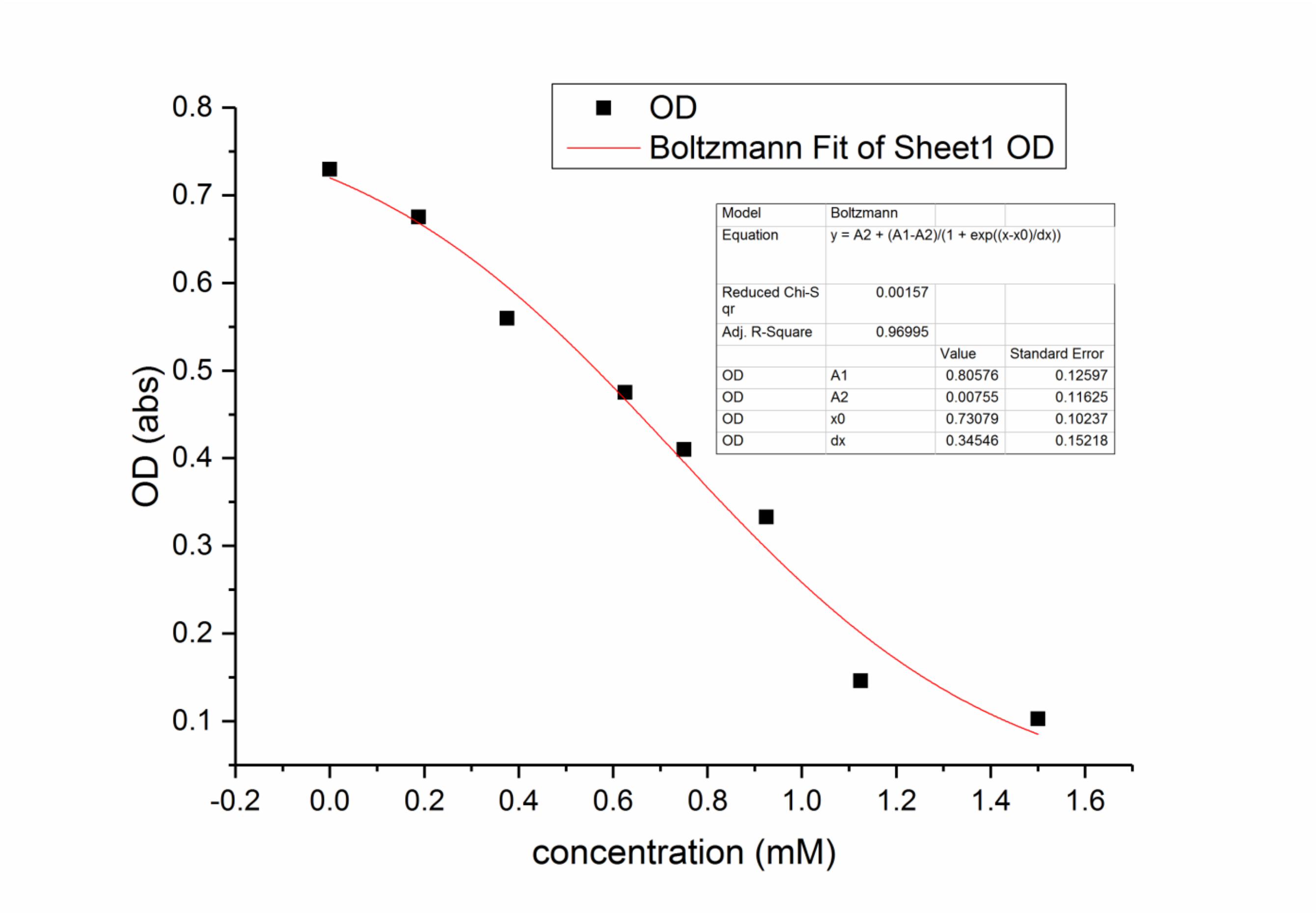

Figure S170. Data obtained from average growth curves with compound **1** at 900 mins and fitted using a Boltzmann fit. This fit was used to calculate the reported MIC_{50} value.

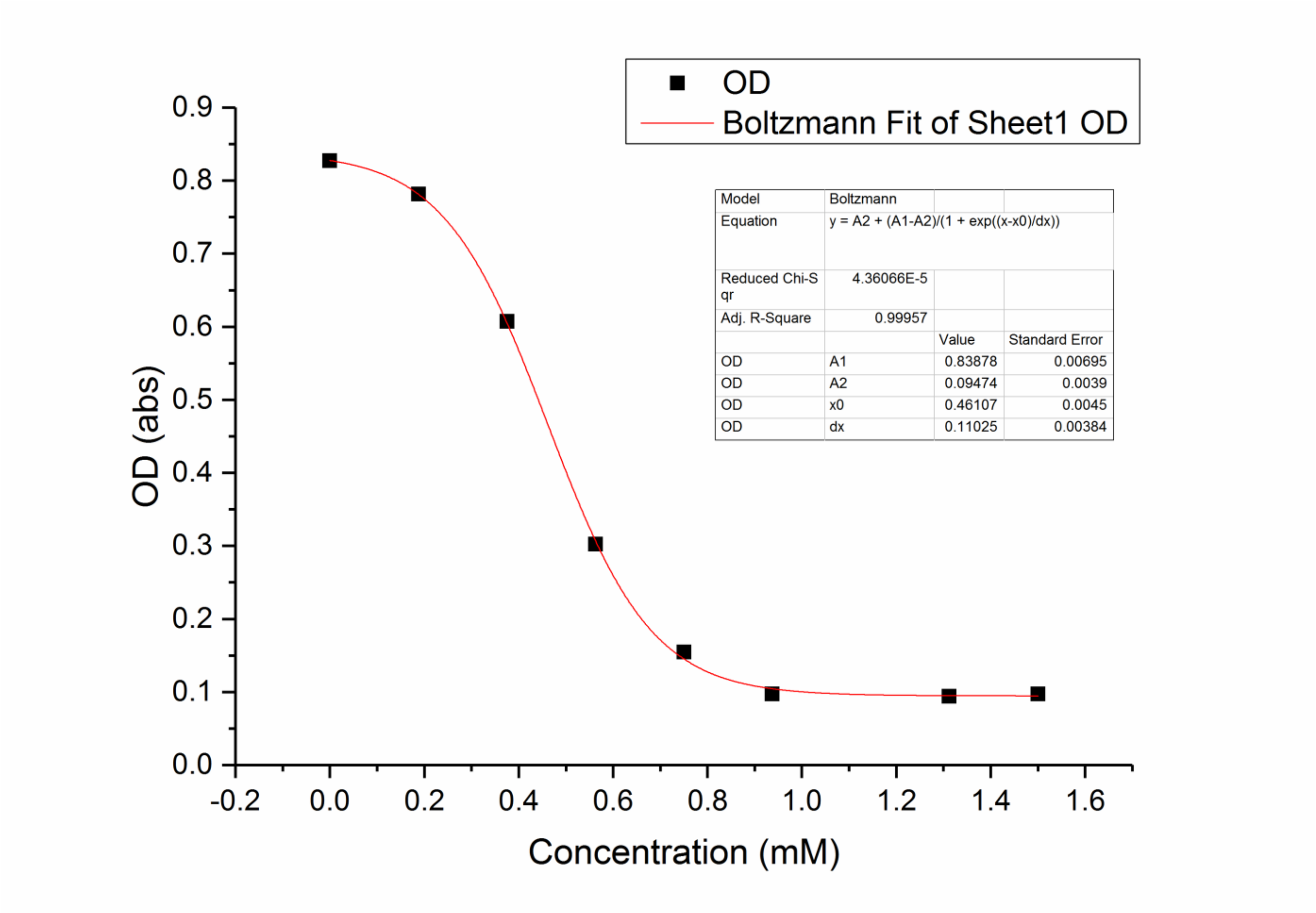

Figure S171. Data obtained from average growth curves with compound **2** at 900 mins and fitted using a Boltzmann fit. This fit was used to calculate the reported MIC_{50} value.

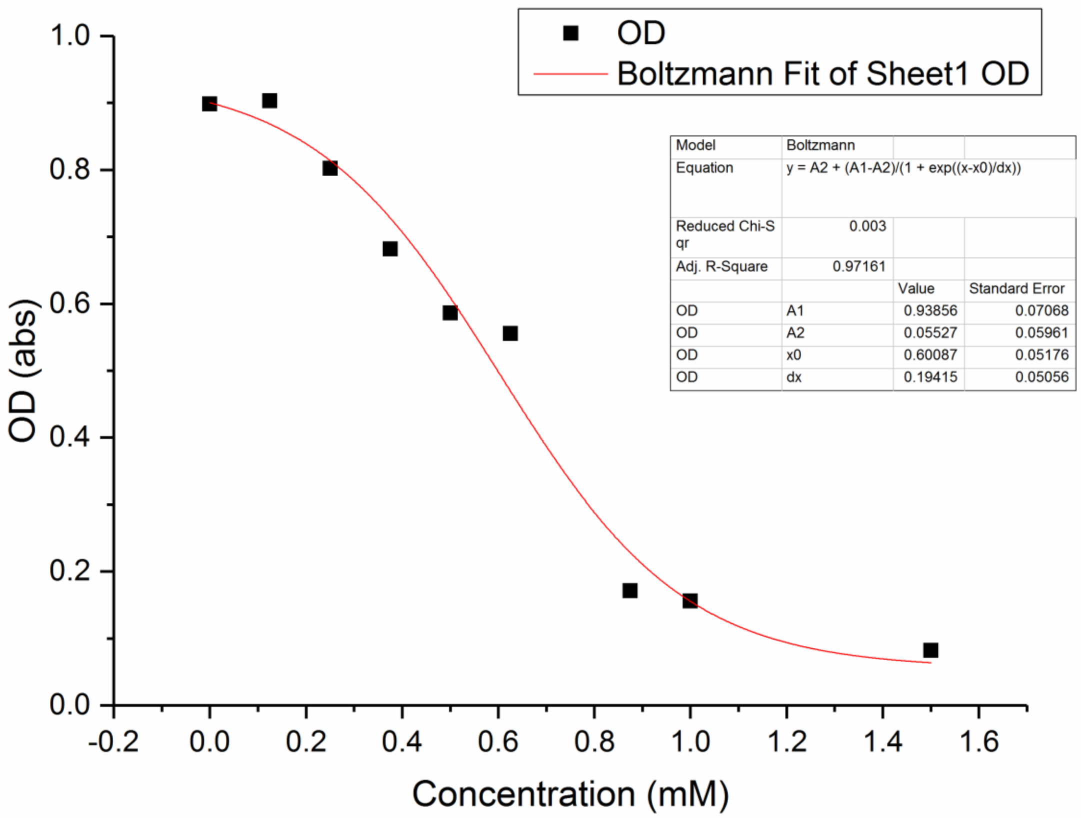

Figure S172. Data obtained from average growth curves with compound **4** at 900 mins and fitted using a Boltzmann fit. This fit was used to calculate the reported MIC_{50} value.

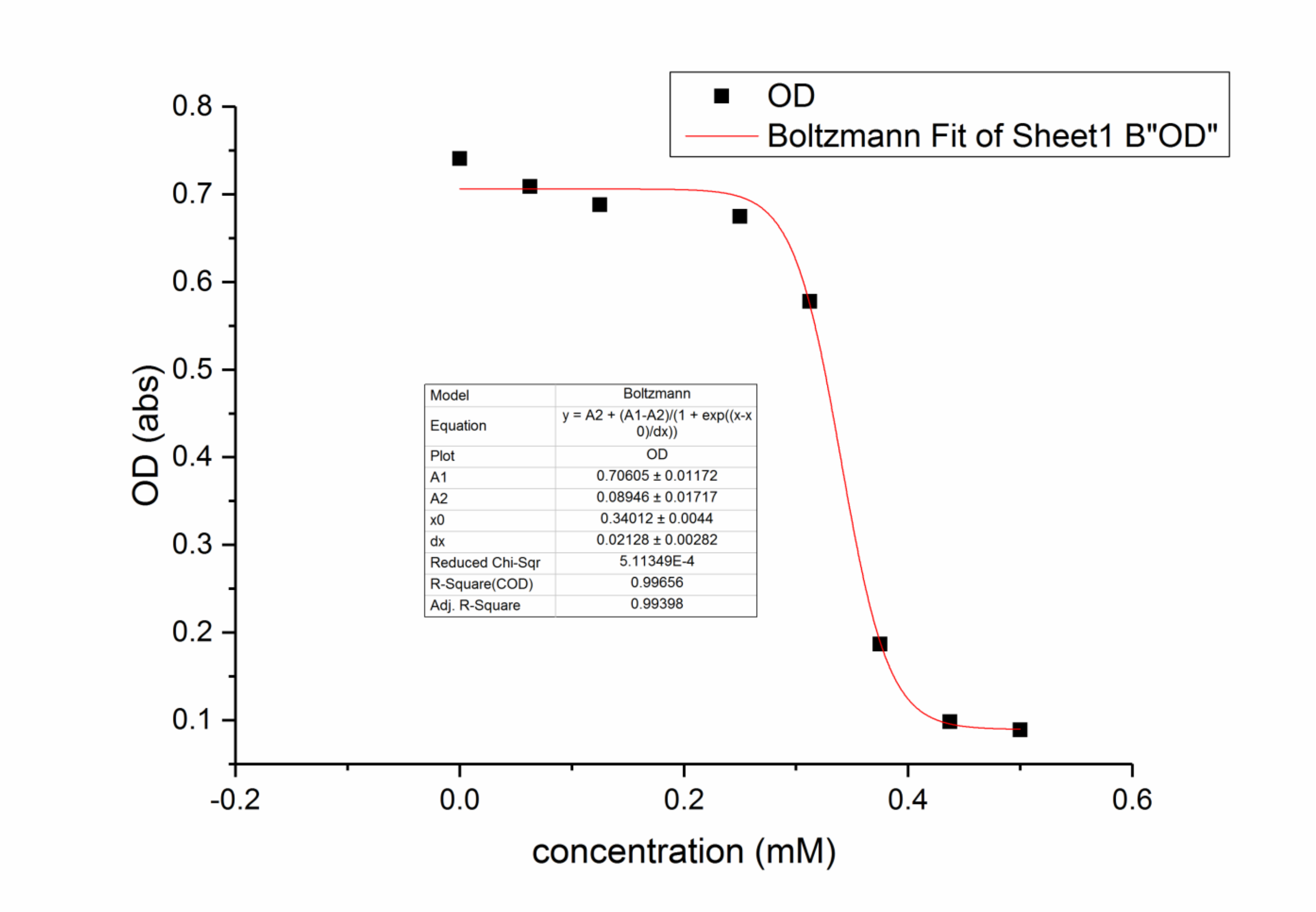

Figure S173. Data obtained from average growth curves with compounds **1** and **2** in a 1:1 mix at 900 mins and fitted using a Boltzmann fit. This fit was used to calculate the reported MIC_{50} value.

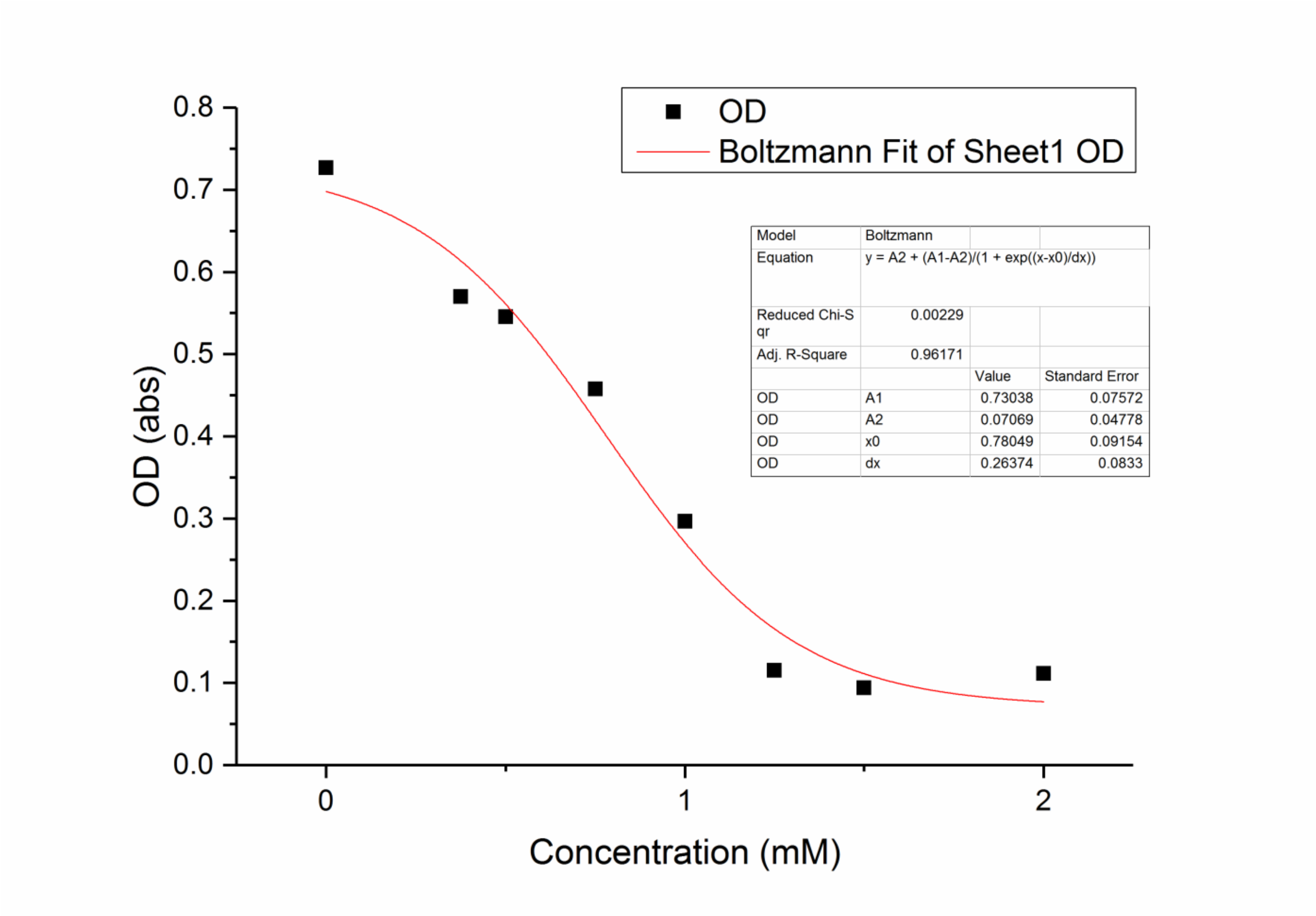

Figure S174. Data obtained from average growth curves with compounds **1** and **4** in a 1:1 mix at 900 mins and fitted using a Boltzmann fit. This fit was used to calculate the reported MIC_{50} value.

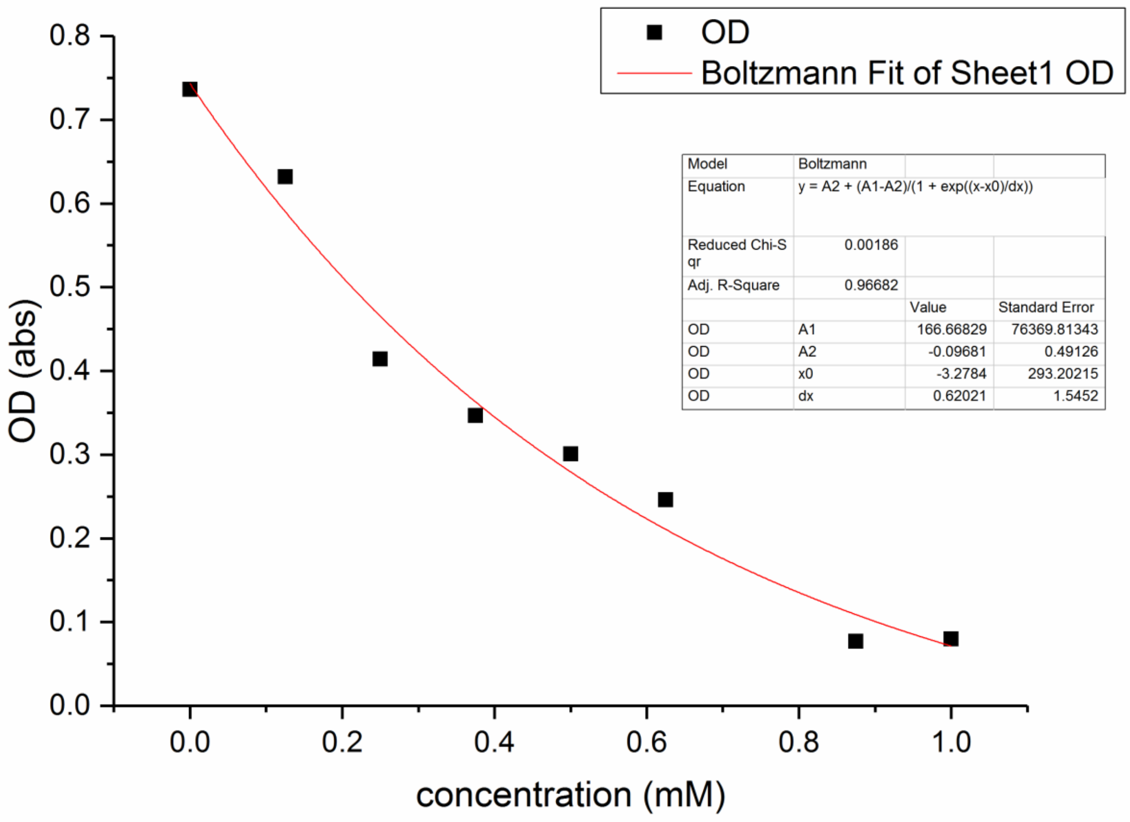

Figure S175. Data obtained from average growth curves with compounds **2** and **4** in a 1:1 mix at 900 mins and fitted using a Boltzmann fit. This fit was used to calculate the reported MIC_{50} value.

Table S9. MIC_{50} values calculated for compounds **1**, **2**, **4** and their 1:1 mixtures[a] against MRSA UAS300.

Compound	MIC_{50}	
	mM	μg/mL
**1**	0.7146	431.1539
**2**	0.4638	264.9921
**4**	0.6068	324.8200
**1** and **2**	0.3382	397.2835
**1** and **4**	0.3169	350.6974
**2** and **4**	0.7505	854.5568

[a] Values represent the total molar concentration or mass of compounds combined.

References

1 G. M. Sheldrick, *Acta Cryst.,* 2015, **A71**, 3-8.
2 G. M. Sheldrick, *Acta Cryst.,* 2015, **C71**, 3-8.
3 O. V. Dolomanov, L. J. Bourhis, R. J. Gildea, J. A. K. Howard, H. Puschmann, *J. Appl. Cryst.,* 2009, **42**, 339-341.
4 D. C. Palmer, *Z. Kristallogr. Cryst.,* 2015, **230**, 559-572.
5 J. M. Andrews, *J. Antimicrob. Chemother.,* 2001, **48**, 5-16.
6 L. J. White, N. J. Wells, L. R. Blackholly, H. J. Shepherd, B. Wilson, G. P. Bustone, T. J. Runacres, J. R. Hiscock, *Chem. Sci.,* 2017, **8**, 7620-7630.
